# Multicomponent Photochemical Assembly of C(sp^2^)–S Substituted Imidazoles via Exciplex Formation

**DOI:** 10.1021/acsorginorgau.6c00004

**Published:** 2026-02-20

**Authors:** Illán Tagarro, Helena F. Piedra, Daniel González-Pinardo, Israel Fernández, Irene Bosque, Manuel Plaza

**Affiliations:** † Departamento de Química Orgánica e Inorgánica and Instituto Universitario de Química Organometálica “Enrique Moles” and Centro de Innovación en Química Avanzada (ORFEO−CINQA), 16763 Universidad de Oviedo, Oviedo 33006, Spain; ‡ Departamento de Química Orgánica and Centro de Innovación en Química Avanzada (ORFEO−CINQA), Facultad de Ciencias Químicas, 16734 Universidad Complutense de Madrid, Madrid 28040, Spain; § Instituto de Síntesis Orgánica (ISO) and Departamento de Química Orgánica, Universidad de Alicante, Alicante 03080, Spain

**Keywords:** photochemistry, exciplex, CS bond formation, mechanistic studies, organic chemistry

## Abstract

We report a visible-light-driven multicomponent strategy for the synthesis of *S*-functionalized imidazoles from readily available organic halides, isothiocyanates, and isocyanides. The method operates under mild, photocatalyst-free conditions and exhibits broad substrate scope with excellent functional-group tolerance. Detailed mechanistic investigations support a reaction pathway involving direct photoexcitation of in situ generated thiophenolate intermediates and subsequent radical-based C–S bond formation. This process is likely governed by exciplex-type interactions rather than preassembled electron donor–acceptor complexes. Overall, this work introduces a distinct photochemical activation mode for C–S bond construction and expands the synthetic toolbox for accessing sulfur-containing heterocycles.

## Graphical abstract

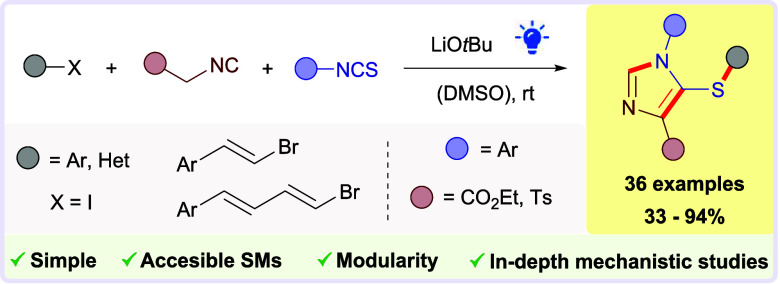

## 1 General Introduction

Carbon–sulfur bonds are fundamental structural motifs that permeate a broad range of chemical disciplines, appearing in pharmaceuticals, agrochemicals, functional materials, and numerous natural products.^1–4^ Because molecules bearing C–S linkages frequently display valuable biological and physicochemical properties, the development of reliable methods to construct these bonds remains a central objective in synthetic chemistry.^5–7^ Classical approaches including nucleophilic substitution,^8^ metal-catalyzed cross-couplings,^9^ and various radical-based protocols^10,11^ have enabled important progress in this area. However, these strategies can be limited by demanding reaction conditions, costly catalysts, or the need for stoichiometric activating agents, factors that may undermine sustainability and restrict the tolerance of sensitive functional groups.^12^ These challenges underscore the ongoing need for alternative, milder, and more versatile tactics for forging C–S bonds. For these reasons, the development of photochemical radical-based strategies for constructing C–S bonds has become an increasingly active and impactful area within modern organic synthesis^13–16^ Among the emerging approaches, the exploitation of the strong reducing ability of photoexcited sulfur anions has proven particularly powerful.^17,18^ Over the past few years, two conceptually distinct strategies have been established to enable C­(sp^2^)–S bond formation under visible-light irradiation.

The first relies on the photoexcitation of electron donor–acceptor (EDA) complexes,^19–21^ supramolecular assemblies formed through noncovalent interaction between an electron-deficient acceptor and an electron-rich donor.^22–24^ Upon visible-light excitation, these complexes undergo photoinduced electron transfer (PET), generating radical ions that can engage in diverse downstream processes including cross-couplings, functionalizations, and cyclizations. In the context of C­(sp^2^)–S bond construction,^25,26^ the most widely explored variant uses organic halides as electron acceptors and thiolate salts as electron donors (Scheme 1a). A seminal demonstration was reported in 2017 by Miyake and co-workers, who showed that EDA complexes formed between aryl bromides/iodides and thiophenolate anions undergo PET upon irradiation, leading to C–X bond cleavage and generation of an aryl radical that recombines with the corresponding sulfur-centered radical.^27^ More recently, our group expanded this activation mode to alkenyl and 1,3-dienyl halides, enabling a range of C­(sp^2^)–S bond-forming transformations including thioetherifications,^28,29^ carbothiophosphorylations,^30^ sulfonylations,^31^ and thiocyanations.^32^ It is worth noting that EDA-complex activation has become a broadly useful platform for C–S bond formation, with many groups contributing significantly to this rapidly advancing field.^33–38^

**Scheme 1:**
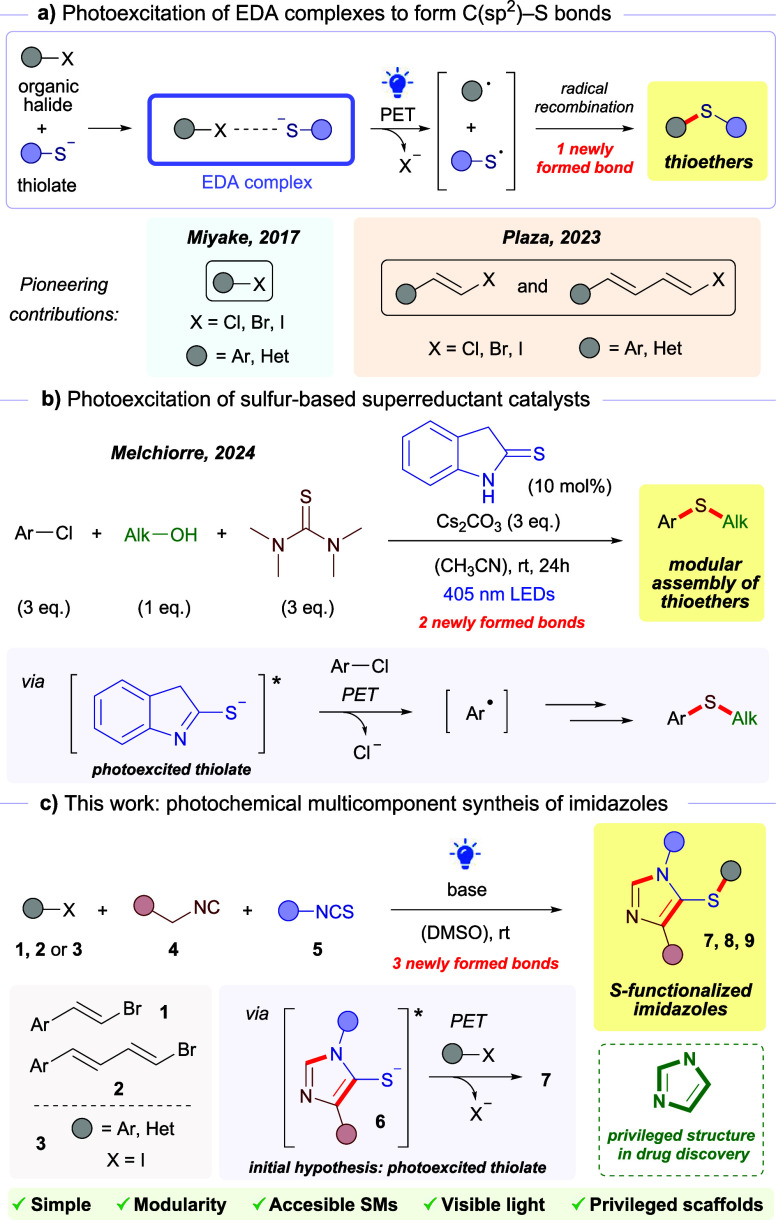
Photochemical Metal-Free Strategies to Form C­(sp^2^)–S Bonds from Organic Halides (a, b); This Work (c)

A second, mechanistically distinct approach involves direct photoexcitation of sulfur anions, without requiring preassembly of an EDA complex. A notable recent example was reported by Melchiorre and co-workers,^39,40^ who developed a metal-free method in which a photoexcited indole-thiolate catalyst acts as a potent reductant to generate both alkyl and aryl radicals, ultimately enabling diverse thioetherifications from aryl chlorides and alcohol-derived nucleophiles using tetramethylthiourea as the sulfur source (Scheme 1b).

Building on these precedents, we intended to develop a multicomponent strategy in which a thiophenolate anion is generated in situ from simple, modular precursors and subsequently directly photoexcited to promote reductive C–S bond formation with suitable organic halides. Such a concept would streamline access to sulfur-containing architectures while avoiding the need for preassembled ground-state donor–acceptor aggregates. Given the central role of the imidazole ring in pharmaceuticals and chemical biology,^41,42^ we were particularly motivated to apply this approach to the synthesis of *S*-functionalized imidazoles. A recent study by Yang and co-workers demonstrated that imidazole-substituted thiophenolate anions can participate in photochemical C–S bond formation when engaged in an EDA complex with thianthrenium salts.^43^ While this elegant work established the reactivity of these thiolates within an EDA framework, we wondered whether direct photoexcitation of the same thiolate species could unlock a distinct activation mode. We reasoned that such a strategy could enable a complementary and operationally simpler route to imidazole-based thioethers from readily available aryl and alkenyl halides (Scheme 1c).

## 2 Results and Discussion

We began our investigation by selecting β-bromostyrene (**1a**), *p*-toluenesulfonylmethyl isocyanide (**4a**), and phenyl isothiocyanate (**5a**) as the starting materials for assembling the targeted *S*-functionalized imidazole **7a**. Formation of the key deprotonated thiophenolate intermediate from **6a** requires a basic medium to promote the initial steps of the multicomponent sequence; therefore, LiO*t*Bu was chosen as the preliminary base. To ensure adequate solubility of all components and to stabilize the polar intermediates expected throughout the process, DMSO was employed as the solvent.

To guide the selection of an appropriate irradiation wavelength, we performed UV–vis spectroscopic analyses (Figure 1). For this purpose, we measured the absorbance profile of the sodium thiophenolate salt of **6b** (red trace), prepared independently (see Section 4 of the Supporting Information). We next evaluated whether β-bromostyrene (**1a**) might form an EDA complex with the thiophenolate salt **6b-Na**. A mixture containing all reaction components (dark blue trace) did not reveal any discernible charge-transfer band, suggesting that significant ground-state complexation is unlikely under these conditions. In any case, we evaluated if irradiation from a 450 nm LED source could enable energy-to-charge transfer (E_*n*_-CT) activation of a ground-state EDA complex,^44^ although we did not observe any representative bathochromic shift. At this point, we hypothesized that the direct photochemical excitation of the **6** salt could promote the in situ formation of a sulfur-superreductant. Based on these observations, we initiated our reaction-optimization studies using violet-to-blue LEDs (390–467 nm), which overlap well with the absorption profile of the thiophenolate salt. A detailed description of this study can be found in Section 6 of the Supporting Information.

**Figure 1:**
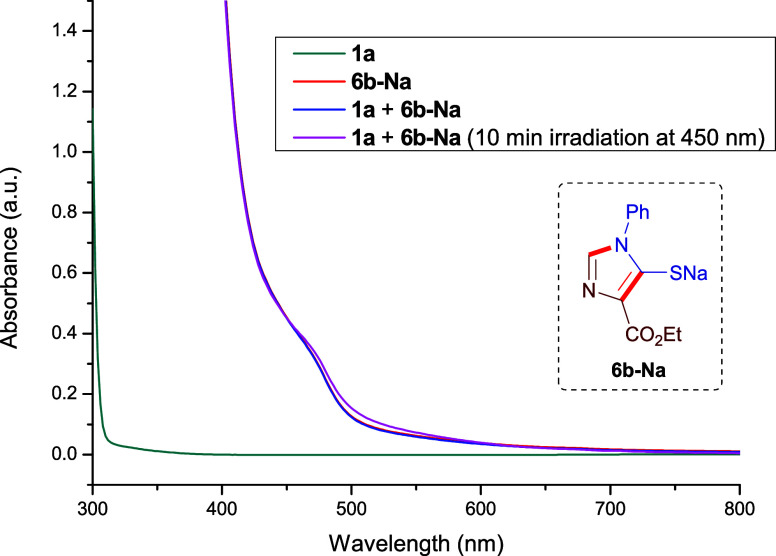
UV–vis spectra (in DMSO) of compounds **1a** (green line), thiophenolate salt **6b-Na** (red line), the mixture of **1a** and **6b-Na** (blue line), and the mixture of **1a** and **6b-Na** after 10 min of irradiation at 450 nm.

Guided by the initial UV–vis analyses, we next turned to the optimization of the reaction conditions (Table 1; for complete data, see Section 2 of the SI). As a starting point, we adopted the same conditions used during the spectroscopic studies: [**1a**] = 0.1 mM, a 2:1.2:1 molar ratio of **4a**:**5a**:**1a**, DMSO as the solvent, and LiO*t*Bu as the base (entry 1). Upon irradiation for 16 h with a 390 nm LED, the desired imidazole **7a** was obtained in 60% conversion, accompanied by a moderate diastereomeric ratio (d.r. = 2.1:1, *trans/cis*). These encouraging results prompted a systematic evaluation of key parameters to improve the efficiency of the transformation.

**Table 1:**
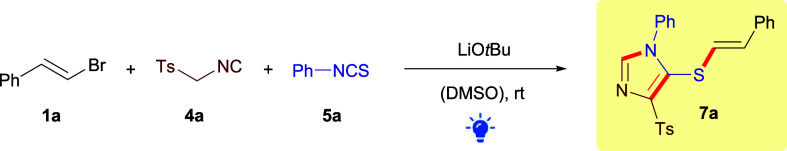
Summary of the Optimization Studies^a^

**entry**	**ratio 4a:5a:1a**	**LED lamp (nm)**	**[1a] (mM)**	**solvent**	**time (h)**	**conversion (%)** ^b^	**d.r. (*trans:cis*)** ^b^
1	2:1.2:1	390	0.1	DMSO	16	60	2.1:1
2	2:1.2:1	427	0.1	DMSO	16	69	1.3:1
3	2:1.2:1	440	0.1	DMSO	16	75	2.4:1
4	2:1.2:1	456	0.1	DMSO	16	73	0.9:1
5	2:1.2:1	467	0.1	DMSO	16	36	5.1:1
6	2:1.2:1	440	0.1	DMF	16	27	3.2:1
7	2:1.2:1	440	0.1	DCM	16	0	
8	2:1.2:1	440	0.1	CH_3_CN	16	0	
9	2:1.2:1	440	0.1	THF	16	0	
10^c^	2:1.2:1	440	0.1	DMSO	16	75	1.1:1
11	2:1.2:1	440	0.1	DMSO	3	14	0.8:1
12	2:1.2:1	440	0.1	DMSO	6	20	0.8:1
13	2:1.2:1	440	0.1	DMSO	72	90	0.3:1
14	2:1.2:1	456	0.2	DMSO	16	88	5.1:1
15^d^	2.5:1.5:1	440	0.1	DMSO	16	>99	0.7:1

a Optimized reaction conditions: **4a** (2.5 equiv), **5a** (1.5 equiv), **1a** (1.0 equiv), lithium *tert*-butoxide (3.0 equiv), DMSO as the solvent, [**1a**] = 0.1 mM, r.t., 16 h, 2 × 440 nm Kessil LEDs.

b The d.r. and conversion values were determined from the ^1^H NMR analysis of the crude reaction mixture.

c The reaction was carried out under an argon atmosphere.

d The reaction was performed using a dual-lamp setup.

We first examined the effect of irradiation wavelength using LEDs spanning the 390–467 nm range (entries 1–5). Among the tested sources, the 440 nm lamp afforded the highest conversion of **7a** (75%, entry 3), in agreement with the absorption profile of thiophenolate **6b**. Subsequently, we assessed the influence of different solvents (DMF, CH_2_Cl_2_, MeCN, THF; entries 6–9). None of these alternatives outperformed DMSO, which remained the solvent of choice. Notably, conducting the reaction under an argon atmosphere (entry 10) did not alter the outcome, indicating that oxygen does not significantly interfere with the process. We then explored the reaction time (entries 11–13). Short irradiation periods (<6 h) resulted in low conversion (<20%), whereas extending the exposure to 72 h increased the conversion to 90%, highlighting both the photostability of the reaction components and the product. Given the impracticality of such long reaction times, we investigated strategies to accelerate the process. Increasing the equivalents of the limiting reagent (entry 14) and employing a dual-lamp setup (entry 15) both improved performance, with the latter providing the most efficient conditions. Overall, the parameters identified in entry 15 were selected as the optimal conditions for this transformation. It is worth mentioning that the same reaction, but starting from β-iodostyrene, afforded the same results as in entry 15. The reaction with β-chlorostyrene, however, led to no conversion toward the desired imidazole **7a**.

With the optimized conditions in hand, we next explored the substrate scope of the *S*-alkenylation reaction leading to imidazoles **7** (Scheme 2a). Overall, the transformation proved to be general and robust, displaying broad functional-group tolerance and delivering the desired products in good to excellent yields. It is worth noting that the diastereoselectivity of the process is inherently challenging to control, largely due to E/Z isomerization of the alkenyl moiety under the irradiation conditions required for the reaction. For this reason, we prioritized reaction efficiency over stereocontrol, accepting moderate d.r. values in exchange for higher overall conversions. A wide range of β-bromostyrene derivatives **1** participated effectively in the reaction. Substrates bearing alkyl groups (**7c**, **7g**, **7k**), nitriles (**7d**), halogens (**7e**, **7i**, **7j**, **7m**, **7n**), nitro groups (**7f**), and thioethers (**7h**) were well tolerated, underscoring the mildness and compatibility of the photochemical conditions. Importantly, heteroaromatic alkenes such as thiophene- (**7p**) and benzofuran-derived (**7q**) β-bromostyrenes also reacted smoothly, enabling the preparation of *bis*-heterocyclic architectures. The modular nature of the multicomponent process allowed diverse combinations of substituents originating from both the isothiocyanate and β-bromostyrene partners, providing an expanded library of products (**7r**–**7z**). Furthermore, the reaction proved applicable to late-stage functionalization of complex, biologically relevant scaffolds. Derivatives of D-glucose (**7aa**), myrtenol (**7ab**), and (−)-menthol (**7ac**) underwent efficient *S*-alkenylation, highlighting the utility of this method for structural diversification of natural product frameworks.

**Scheme 2:**
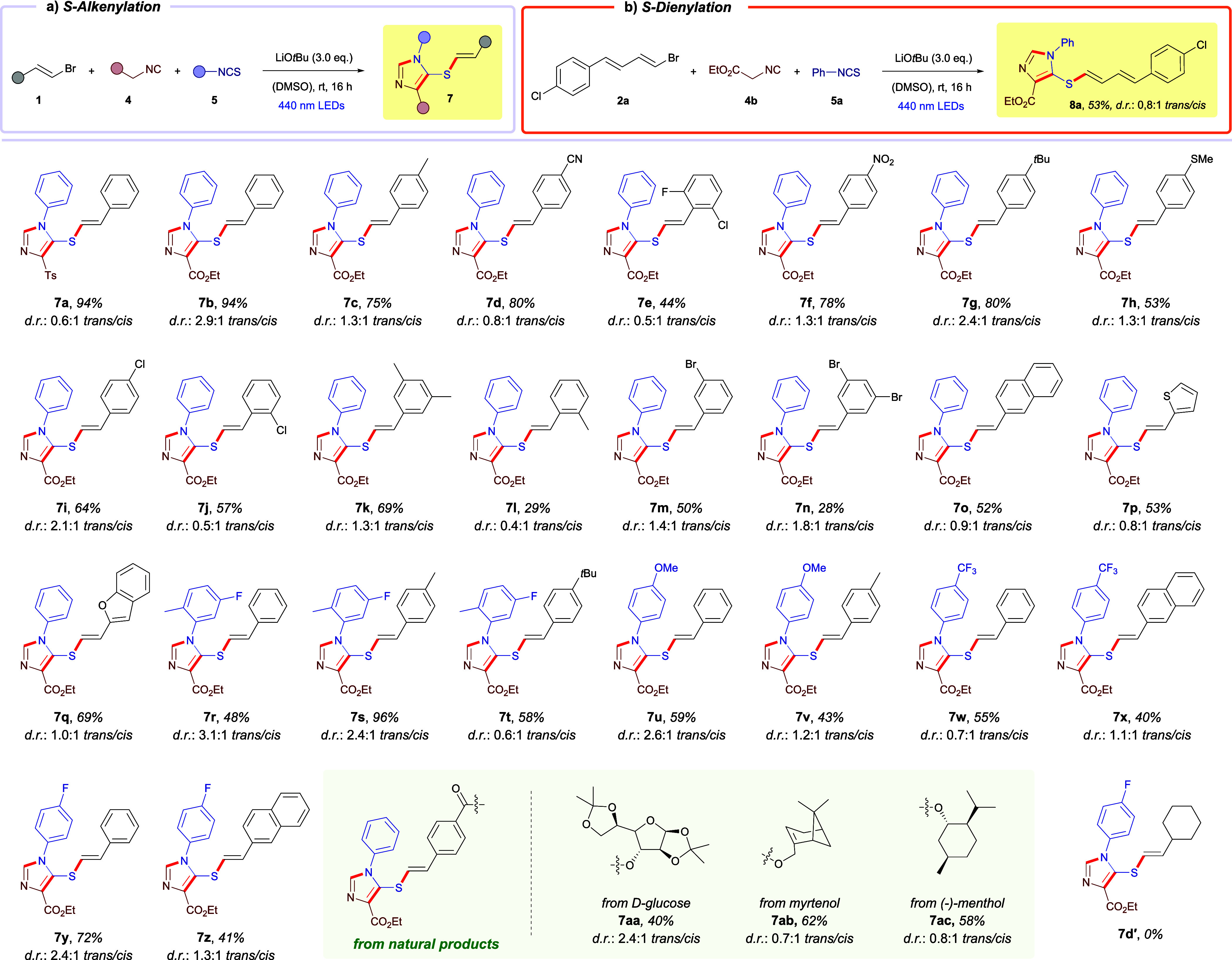
Reaction Scope for the Preparation of *S*-alkenyl and *S*-dienyl Substituted Imidazoles **7** and **8** ^a^

a Reaction conditions: **4** (2.5 equiv), **5** (1.5 equiv), **1** or **2** (1.0 equiv), lithium *tert*-butoxide (3.0 equiv), DMSO as the solvent, [**1** or **2**] = 0.1 mM, r.t., 16 h, irradiation from 2 × 440 nm Kessil LEDs.

Not all substrates were competent under the optimized conditions. For instance, β-haloalkenes bearing aliphatic substituents, such as the cyclohexyl-substituted β-iodoalkene and β-bromoalkene, failed to deliver the desired product (**7d′**). This lack of reactivity may arise from insufficiently favorable reduction potentials or the instability of the resulting alkenyl radical formed after C–X bond cleavage. Furthermore, as will be discussed later in the mechanistic section of this manuscript, the absence of a possible π–π stacking interaction (due to the lack of an aromatic core in the corresponding deprotonated thiol) is consistent with the reactivity observed in this system.

Interestingly, because the transformation generally favors formation of the *trans* isomer, we investigated whether postsynthetic photochemical E/Z isomerization could be employed to selectively enrich the corresponding *cis* isomer. To this end, compound **7b** was chosen as a representative model substrate. A range of photochemical and photocatalytic conditions was evaluated (see Section 5 of the Supporting Information for details). For this particular substrate, we found that irradiation for 24 h with a 390 nm LED effectively inverted the stereochemical outcome, affording *cis*-**7b** as the predominant isomer with a 1:2.6 *trans/cis* ratio.

Finally, we evaluated a substrate derived from a 1,3-diene (**2a**). Gratifyingly, the reaction furnished the corresponding *S*-dienyl imidazole **8a** in good yield (Scheme 2b), demonstrating that this activation mode is not limited to simple alkenyl fragments and can be extended to dienylation, thereby significantly broadening the overall scope of the methodology.

Encouraged by the broad substrate scope observed in the *S*-alkenylation manifold, we next investigated whether the protocol could be extended to C­(sp^2^)–S bond formation with aryl halides, thereby enabling incorporation of an aryl substituent onto the imidazole scaffold. As an initial assessment, we tested bromobenzene and chlorobenzene under the optimized conditions; however, no formation of the desired product was observed with either substrate, consistent with their significantly higher reduction potentials. We therefore turned our attention to the corresponding iodoarenes **3**, anticipating a more favorable reactivity. Our first successful experiment employed iodobenzene (**3a**) in combination with ethyl isocyanoacetate (**4b**), phenyl isothiocyanate (**5a**), and LiO*t*Bu, followed by irradiation for 16 h with two 440 nm LEDs (Scheme 3). Gratifyingly, the desired *S*-arylated imidazole **9a** was isolated in 50% yield, demonstrating that the photochemical thiolate activation mode can be extended beyond alkenyl and 1,3-dienyl halides. To assess the generality of this extension, we evaluated a range of aryl and heteroaryl iodides. Substrates bearing alkyl groups (**9b**, **9d**), trifluoromethyl substituents (**9c**) or aldehyde functionality (**9f**) all participated efficiently, underscoring the functional-group tolerance of the methodology. Importantly, the reaction also proved suitable for accessing *bis*-heterocyclic architectures, as illustrated by the incorporation of pyridine (**9e**) and thiophene (**9g**) motifs into the final imidazole products.

**Scheme 3:**
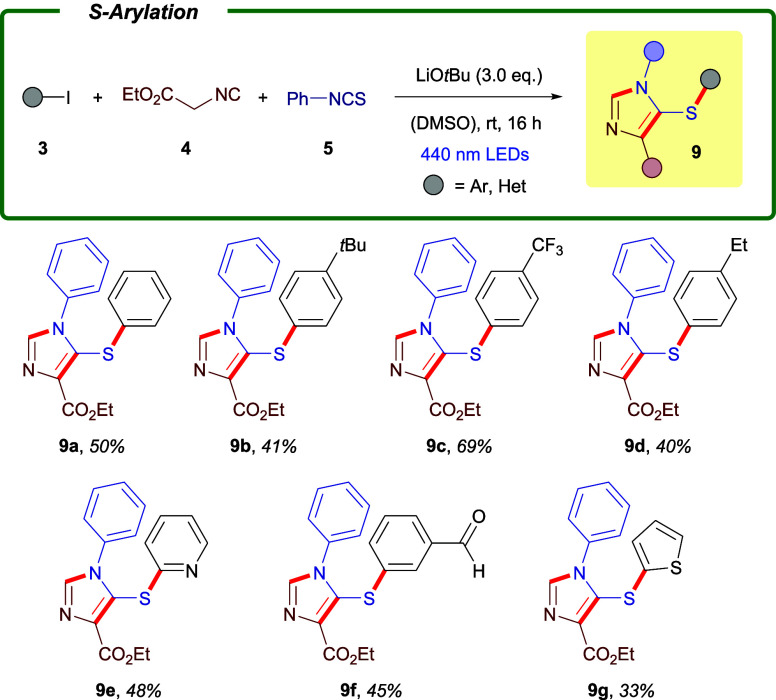
Reaction Scope for the Preparation of *S*-aryl Substituted Imidazoles **9** ^a^

a Reaction conditions: **4** (2.5 equiv), **5** (1.5 equiv), **3** (1.0 equiv), lithium *tert*-butoxide (3.0 equiv), DMSO as the solvent, [**3**] = 0.1 mM, r.t., 16 h, irradiation from 2 × 440 nm Kessil LEDs.

In addition, we carried out a complete set of experimental studies to elucidate the mechanistic nature of this transformation. Initially, we hypothesized that three different mechanistic pathways (I, II and III) could be operating (Scheme 4). Pathway I (Scheme 4a) would involve the formation of a ground-state EDA complex **10** between the thiophenolate salt of **6** and one of the organic halides (**1**, **2**, or **3**). Photochemical excitation of this complex would lead to its fragmentation through mesolytic C–X bond reduction to afford the corresponding carbon-centered radical **11** and the sulfur-centered radical **12**. A rapid radical recombination would eventually lead to the construction of the *S*-functionalized imidazoles. However, as previously stated, no detection of the EDA complex was possible through either the UV–vis analysis or NMR titration experiments (see Section 7 of the Supporting Information). Therefore, this mechanistic pathway was discarded.

**Scheme 4:**
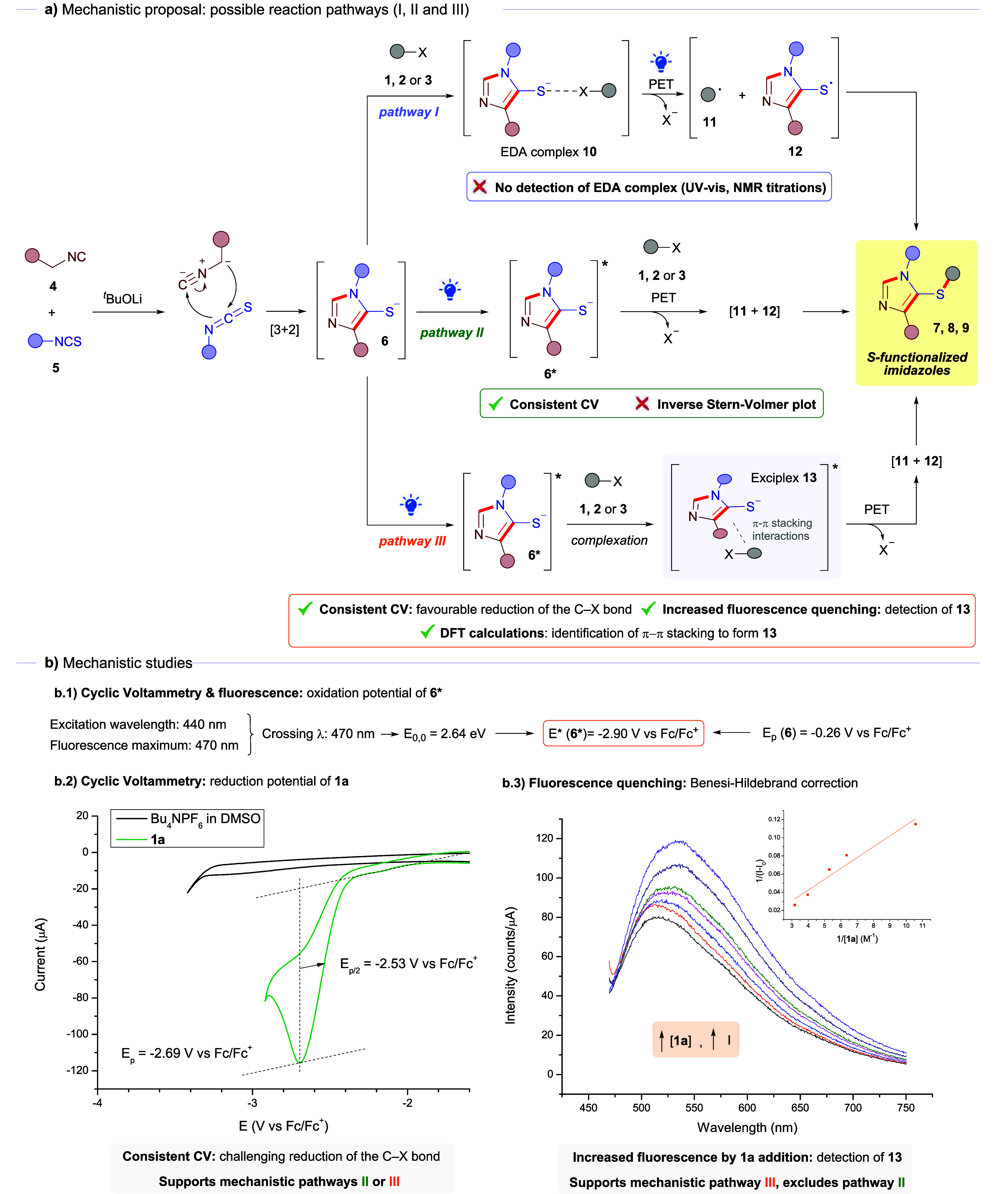
(a) Representation of the Possible Mechanistic Pathways (I, II, or II), (b) Mechanistic Studies: Calculation of the Oxidation Potential of 6*, Cyclic Voltammetry of **1a**, Fluorescence Quenching, and Benesi–Hildebrand Analysis (Inset)

Thus, we considered pathway II as a plausible alternative for the generation of the final products **7**, **8** or **9** via the photochemical excitation of the thiophenolate salt from **6**, which potentially behaved as a super-reducing agent in its excited state and therefore could also promote the heterolytic cleavage of the C–X bond in the organic halides to create the radical intermediates **11** and **12** (Scheme 4a). In this regard, the feasibility of the reaction between thiolate **6b-Na** and substrate **1a** was evaluated through electrochemical and spectroscopic analyses. Steady-state absorption and CV studies showed no evidence of interaction between thiolate **6b-Na** and **1a** in their ground states, suggesting that no EDA complex is formed prior to excitation. The reduction potential of substrate **1a** was determined by cyclic voltammetry (CV) to be −2.69 V vs Fc/Fc^+^ (Scheme 4b), representing a challenging target for single-electron transfer. In the ground state, thiolate **6b-Na** exhibits an oxidation potential (*E*_ox_) of – 0.26 V vs Fc/Fc^+^. Comparing these values, the SET from the ground-state thiolate to **1a** is highly endergonic (Δ*G*_ET_ ≫ 0), which is consistent with the lack of reactivity observed under dark conditions. Upon photoexcitation, the reducing power of **6b-Na** is significantly enhanced. The zero–zero excitation energy (*E*_0,0_) was estimated to be 2.64 eV from the intersection of the absorption and fluorescence spectra (λ_int_ = 470 nm) (see Section 9 of the Supporting Information for details). According to the Rehm–Weller equation (E*_ox_=*E*_ox_–*E*_0,0_), the oxidation potential of the excited state was calculated as −2.90 V vs Fc/Fc^+^. The drastic shift in the oxidation potential upon excitation provides a substantial thermodynamic driving force for the reaction. The excited-state potential of **6b-Na** is sufficiently more negative than the reduction potential of substrate **1a**, resulting in a favorable downhill electron transfer. These results confirm that the reaction is exclusively gated by light, as only the excited state of the thiolate possesses the requisite reducing strength to activate substrate **1a**.

Furthermore, quenching experiments revealed an interesting result (see Section 8 of the Supporting Information for details). Contrary to the expected quenching, the emission of 6b* was enhanced in the presence of **1a** (Scheme 4b), meaning that a classical Stern–Volmer approach is no longer operative. This behavior suggests the stabilization of an excited-state association that occurs exclusively upon excitation, potentially leading to the formation of a luminescent exciplex or an excited-state EDA assembly. Utilizing the Benesi–Hildebrand equation,^45–47^ which takes into account this phenomenon, the association constant of this complex was calculated to be *K*_*a*_ = 1.26 M^–1^, a modest association constant that reflects a weak-to-moderate interaction in the excited state, consistent with a transient and dynamic exciplex rather than a strongly bound ground-state complex. Therefore, at this point we considered a last possible mechanistic pathway, which would involve the formation of an exciplex **13** between the photoexcited thiophenolate anion 6* and the organic halides (pathway III; Scheme 4a).^48^ Reduction of the C–X bond of the latter through single electron transfer at the exciplex would furnish radical intermediates **11** and **12**, whose recombination leads to the formation of the final products. Since the reaction proceeds equally well when running the reaction under an argon atmosphere, we discarded oxygen-mediated oxidation of the starting thiophenol to generate sulfur-centered radicals upon photoexcitation.^49^ Given all the experimental studies, we consider this last mechanistic pathway (III) as the most likely.

Finally, preliminary Density Functional Theory (DFT) calculations at the dispersion-corrected CPCM­(DMSO)-PBE0-D3BJ/def2-TZVP level were carried out to gain more insight into the formation and nature of the above proposed exciplex **13** from the photoreaction involving **1a** and thiolate **6b**. By means of an initial conformational search using the CREST (Conformer-Rotamer Ensemble Sampling Tool) program followed by DFT optimization (see Computational Details in the SI), we could identify two potential aggregation minima (i.e., EDA complexes) on the ground-state surface, namely a species mainly stabilized by a π–π stacking interaction, **EDA-S**_**0**_, and a higher lying isomer, **EDA′-S**_**0**_, stabilized by a halogen bonding involving the sulfide atom (Figure 2). From the data in Figure 2, it becomes clear that the formation of both EDA complexes is endergonic (i.e., energetically disfavored with respect to the separate reactants), which is fully consistent with the lack of detection of any EDA complex through either the UV–vis analysis or NMR titration experiments (see above). Therefore, the formation of the exciplex from either **EDA-S**_**0**_ or **EDA′-S**_**0**_ seems unlikely, which further discards the pathway I discussed above.

**Figure 2:**
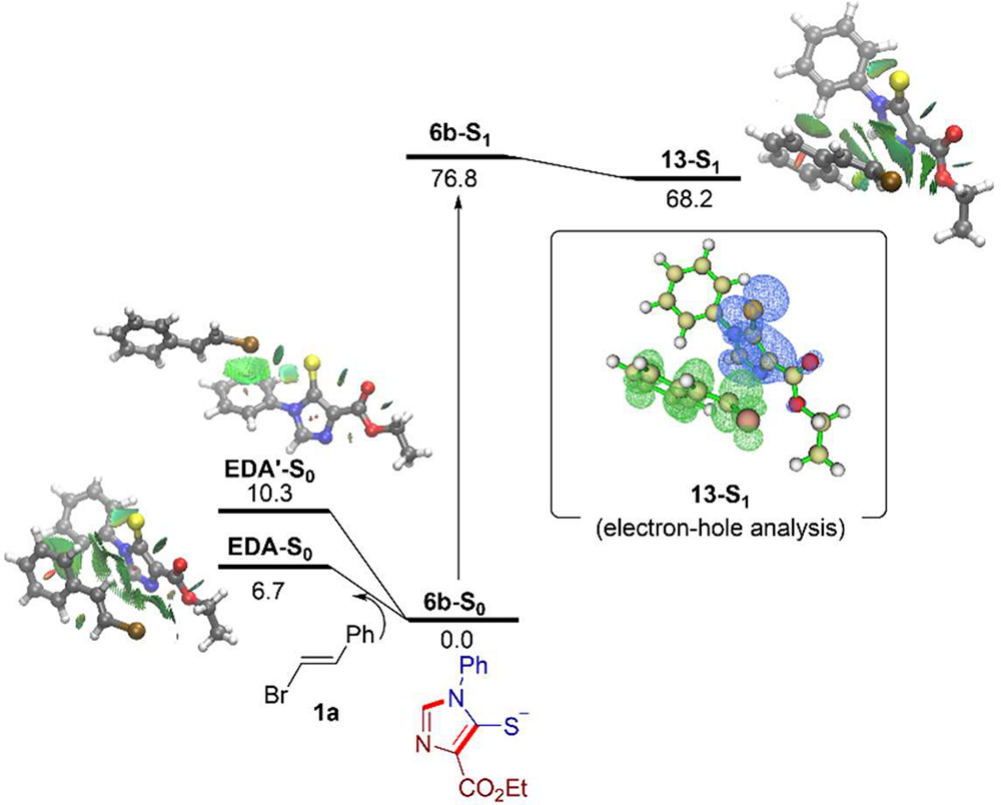
Computed reaction profile for the formation of the exciplex **13-S**_**1**_. Relative free energies (Δ*G*) are given in kcal/mol. Inset: electron–hole analysis for **13-S**_**1**_, where the charge-transfer takes place in the direction from blue to green. All data have been computed at the CPCM­(DMSO)-(TD)-PBE0-D3BJ/def2-TZVP level.

Instead, by means of the Time Dependent (TD)-DFT calculations, we found that upon excitation, thiolate **6b** is transformed into the excited **6b-S**_**1**_ species, which in the presence of the bromostyrene **1a** leads to the formation of the excited complex **13-S**_**1**_ in the S_1_-excited hypersurface. At variance with the results in the S_0_-ground state, the formation of this exciplex is exergonic (ΔG = −8.6 kcal/mol), which agrees with the experimental results and further supports the proposed pathway III. According to the NCIplot method (see inset in Figure 2), this exciplex is stabilized by two main noncovalent interactions, namely a π–π stacking between the heteroaromatic ring and the C=C of the styrene and a CH···π interaction involving the phenyl groups of both fragments. Furthermore, according to the electron–hole analysis (see Figure 2), this π–π interaction facilitates the charge-transfer from the thiolate to the styrene in the exciplex, which, in turn, weakens the C–Br bond (Mayer bond order of 0.93 vs 1.00 in **13-S**_**1**_ and **1a**, respectively), therefore facilitating subsequent bond cleavage.

## 3 Conclusions and Outlook

In conclusion, we have developed a photocatalyst-free, visible-light-driven multicomponent strategy for the synthesis of *S*-functionalized imidazoles from readily available organic halides, isothiocyanates, and isocyanides. Central to this approach is the in situ generation and direct photoexcitation of thiophenolate intermediates, which enables radical-based C–S bond formation under mild conditions without the need for preassembled electron donor–acceptor complexes. The method exhibits broad substrate scope, tolerating a wide range of functional groups and accommodating both alkenyl and aryl halides, including heteroaryl motifs and structurally complex, biologically relevant scaffolds. Mechanistic studies support a pathway involving excited thiolate species and exciplex-type interactions, providing insight into an alternative photochemical activation mode for sulfur anions. Overall, this work expands the conceptual framework for photochemical C–S bond formation and highlights the potential of direct thiolate photoexcitation as a versatile tool for assembling sulfur-containing heterocycles. We anticipate that this strategy will inspire further developments in multicomponent and photocatalyst-free radical transformations.

## 5 Experimental/Method Section

### General Procedure for the Synthesis of Compounds **7**, **8**, and **9**

In a 5 mL glass vial, isocyanide **4** (0.50 mmol, 2.5 equiv), isothiocyanate **5** (0.30 mmol, 1.5 equiv), and lithium *tert*-butoxide (0.60 mmol, 3.0 equiv) were dissolved in DMSO (2 mL). The resulting mixture was stirred under air for 30 min to generate the corresponding thiolate anion. Subsequently, the corresponding vinyl bromide **1**, dienyl bromide **2** or aryl iodide **3** (0.20 mmol, 1.0 equiv) was added. The vial was sealed and positioned between two 440 nm LED lamps at a distance of approximately 3 cm from each source. Both lamps were operated at full intensity under continuous ventilation, and the reaction was stirred for 16 h at room temperature. After completion, the reaction mixture was transferred to a separatory funnel and diluted with H_2_O (5 mL) and EtOAc (5 mL). The aqueous layer was extracted with EtOAc (2 × 5 mL), and the combined organic extracts were washed with brine (2 × 5 mL). The organic phase was dried over anhydrous Na_2_SO_4_, filtered, and concentrated under reduced pressure, followed by high vacuum. The crude product was analyzed to determine the E/Z diastereomeric ratio (d.r.) and subsequently purified by column chromatography to afford the desired products **7**, **8** and **9**.

## Supplementary Material



## Data Availability

The data underlying this study are available in the published article and its Supporting Information.

## Notes

General considerations; optimization of reaction conditions; experimental procedures and characterization data for compounds **7**–**9**; isolation of intermediate **6b**; photochemical isomerization of **7b**; UV–vis spectra; NMR titration experiments; quenching experiments; calculation of the excited-state oxidation potential of **6b–Na**; cyclic voltammetry data; computational details; and copies of NMR spectra (PDF)
