# Supplementary material for: Multicomponent Photochemical Assembly of C(sp2)–S Substituted Imidazoles via Exciplex Formation
Source: ACS Org Inorg Au. 2026 Feb 20;6(3):337–45. doi: 10.1021/acsorginorgau.6c00004 (PMC13237609; doi:10.1021/acsorginorgau.6c00004)

Supplementary Information for

Multicomponent Photochemical Assembly of C(sp²)–S Substituted Imidazoles via Exciplex Formation

Illán Tagarro,[a] Helena F. Piedra,[a] Daniel González-Pinardo,[b] Israel Fernández,[b]* Irene Bosque*,[c] and Manuel Plaza*[a]

[a] *Departamento de Química Orgánica e Inorgánica* and *Instituto Universitario de Química Organometálica "Enrique Moles"* and *Centro de Innovación en Química Avanzada (ORFEO-CINQA)*. Universidad de Oviedo. Julián Clavería, 8. 33006, Oviedo. Spain.

[b] *Departamento de Química Orgánica* and *Centro de Innovación en Química Avanzada (ORFEO-CINQA)*, Facultad de Ciencias Químicas, Universidad Complutense de Madrid, 28040, Madrid, Spain.

[c] *Instituto de Síntesis Orgánica (ISO) and Departamento de Química Orgánica.* Universidad de Alicante, Apdo. 99, 03080 Alicante, Spain.

CONTENTS

1. General considerations 2
2. Optimization of the reaction conditions 4
3. General procedure for the synthesis and characterization data for the compounds **7**, **8** and **9** 6
4. Isolation of the intermediate **6b**. 46
5. Photochemical isomerization of product **7b**. 47
6. UV-Vis spectra. 50
7. NMR titration experiments. 50
8. Quenching experiments 52
9. Calculation of the oxidation potential of the excited state of **6b-Na**. 54
10. Cyclic voltammetries. 57
11. Computational calculations. 60
12. Copies of the NMR spectra. 72

1. General considerations

All of the vinyl bromides **1** were prepared following previously reported methodologies.[1] The different reagents employed during the development of this work are commercially available from Sigma Aldrich Chemical co., Acros Organics Chemical co. and Alfa Aesar Chemical co. Dry DMSO stored with molecular sieves acquired from Sigma Aldrich Chemical co. was used for the photochemical reactions.

A Kessil® PR160 Rig equipped with different lamps (PR160-366nm, PR160-390nm, PR160-427nm, PR160-440nm and PR160-456nm) and a cooling fan was used as the photochemistry setup. A PR time controller was additionally used to select the irradiation time. 5 ml glass vials purchased in VWR® were used to run the photochemical reactions. The vials were sealed with a cap after adding the chemicals and solvent and placed at a distance of approximately 5 cm away from the lamp prior to irradiation at maximum intensity (100% power) of the Kessil lamp. Figures SI-1 to SI-3 illustrate relevant photophysical properties of the lamps.

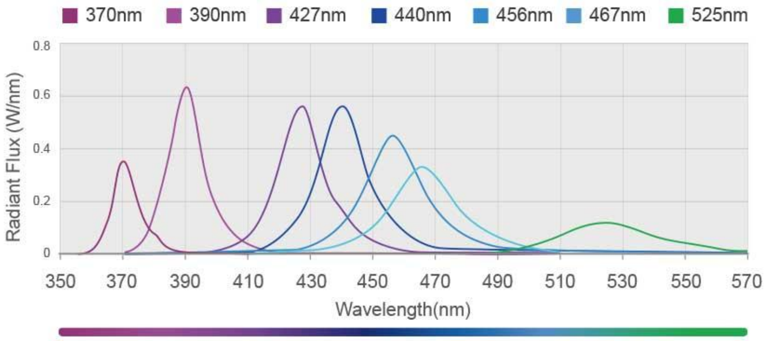

Figure SI-1. Emission spectrums of the different Kessil® lamps.

Power Consumption	370nm (max 43W), 390nm (max 52W), 427nm & 440nm (max 45W), 456nm (max 50W), 467nm (max 44W), 525nm (max 44W)
Input Voltage	100-240 VAC
Operating Temperature	0 - 40°C / 32 - 104°F
Beam Angle	56°
Wavelength Options	370nm, 390nm, 427nm, 440nm, 456nm, 467nm, 525nm
Average Intensity of PR160 series	352mW/cm2 (measured from 1 cm distance)
Dimensions (H x D)	4.49" x 2.48" / 11.4cm x 6.3cm

Figure SI- 2. Technical specifications of the Kessil® lamps.

[1] For the synthesis of the vinyl bromides **1**, we followed a two-step sequence based on a dibromoolefination reaction of the corresponding aldehyde precursor (A. R. Silva, E. C. Polo, N. C. Martins, Correia, D. C. Roque. *Adv. Synth. Catal.* **2018**, *360*, 346.) followed by a dehalogenation reaction (Y. Ye, H. Chen, K. Yao, H. Gong. *Org. Lett.* **2020**, *22*, 2070.).

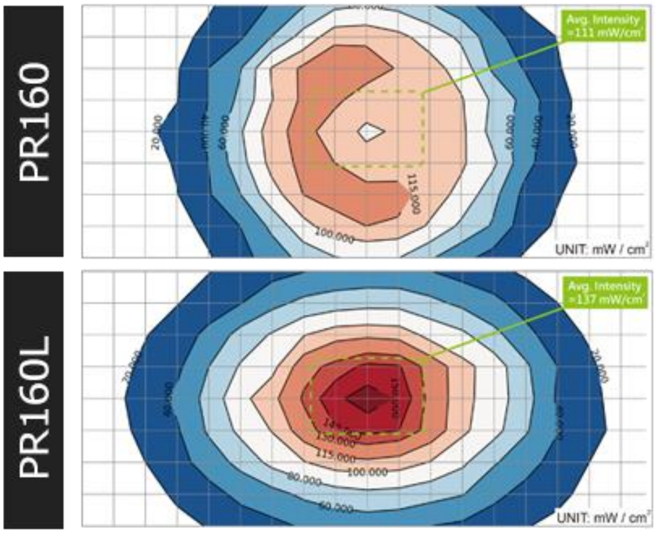

Figure SI-3. Intensity map of the Kessil® lamps.

NMR spectra were recorded in $CDCl_3$ at 300 MHz for ^1H, 75 MHz, 100 MHz for ^{13}C and 282 MHz for ^{19}F, with tetramethylsilane as internal standard for ^1H and the residual solvent signals as standard for ^{13}C. The data is being reported as s = singlet, bs = broad singlet, d = doublet, dd = double doublet, t = triplet, dt = double triplet, td = triple doublet, q = quatriplet, p = quintuplet and m = multiplet or unresolved, chemical shifts in ppm and coupling constant(s) in Hz. HRMS were measured in ESI or EI mode, and the mass analyser of the HRMS was TOF (Bruker model Impact II).

UV-visible spectra and fluorescence measurements were performed in a Duetta spectrofluorimeter.

Cyclic voltammograms were recorded using an EmStatblue+ potentiostat with a standard three-electrode setup: a 3 mm diameter vitreous carbon electrode as the working electrode, a platinum wire as the counter electrode, and an Ag/AgCl electrode (0.10 M aqueous KCl) as the reference electrode. Data was analyzed using OriginLab software.

The cyclic voltammetry (CV) plots follow the IUPAC-recommended plotting conventions. In all cases, anodic currents are shown as positive and cathodic currents as negative, with potentials reported according to standard electrochemical notation.

2. Optimization of the reaction conditions.

Table S1. Screening for the optimal conditions for the reaction.[a]

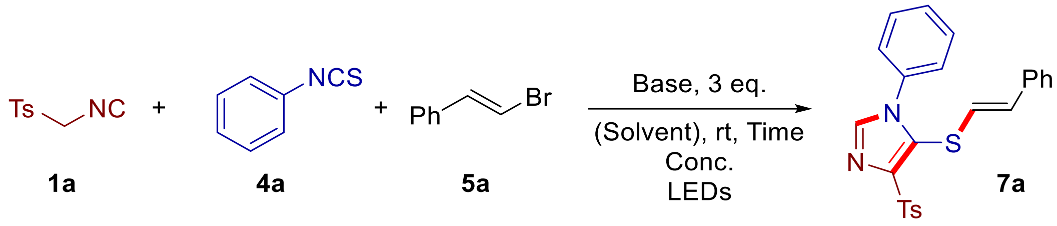

Entry	**4a**:**5a**:**1a**	Lamp (nm)	[**1a**] (M)	Solvent	Time (h)	Base	Conv.[b]	d.r. (*E*:*Z*)[b]
1	2:1.2:1	390	0.1	DMSO	16	LiO*t*Bu	60%	2.1:1
2	2:1.2:1	427	0.1	DMSO	16	LiO*t*Bu	69%	1.3:1
3	2:1.2:1	440	0.1	DMSO	16	LiO*t*Bu	75%	2.4:1
4	2:1.2:1	456	0.1	DMSO	16	LiO*t*Bu	73%	0.9:1
5	2:1.2:1	467	0.1	DMSO	16	LiO*t*Bu	36%	5.1:1
6	2:1.2:1	440	0.1	DMF	16	LiO*t*Bu	27%	3.2:1
7	2:1.2:1	440	0.1	CH_2Cl_2	16	LiO*t*Bu	0%	-
8	2:1.2:1	440	0.1	CH_3CN	16	LiO*t*Bu	0%	-
9	2:1.2:1	440	0.1	THF	16	LiO*t*Bu	0%	-
10[c]	2:1.2:1	440	0.1	DMSO	16	LiO*t*Bu	75%	1.1:1
11	2:1.2:1	440	0.1	DMSO	3	LiO*t*Bu	14%	0.8:1
12	2:1.2:1	440	0.1	DMSO	6	LiO*t*Bu	20%	0.8:1
13	2:1.2:1	440	0.1	DMSO	72	LiO*t*Bu	90%	0.3:1
14	2:1.2:1	456	0.2	DMSO	16	LiO*t*Bu	88%	5.1:1
15	2:1.2:1	456	0.2	DMSO	16	NaOH	75%	1.4:1

16	2:1.2:1	456	0.2	DMSO	16	DBU	0%	-
17	2:1.2:1	456	0.2	DMSO	16	LiOH	79%	3.4:1
18	2:1.2:1	456	0.2	DMSO	16	KO*t*Bu	41%	1.7:1
19[d]	2.5:1.5:1	440	0.1	DMSO	16	LiO*t*Bu	>99%	0.7:1

[a] Optimized reaction conditions: **4a** (2.5 eq.), **5a** (1.5 eq.), **1a** (1.0 eq.), lithium tert-butoxide (3.0 eq.), DMSO, 0.1 mM, r.t., 16 hours, 440 nm LEDs. [b] The d.r. and conversion values were determined from the ^{1}H NMR analysis of the crude reaction mixture. [c] The reaction was carried out under an argon atmosphere. [d] The reaction was performed using a dual-lamp setup, which was ultimately selected as the optimal set of conditions.

3. General procedure for the synthesis and characterization data for the compounds 7, 8 and 9.

General procedure A

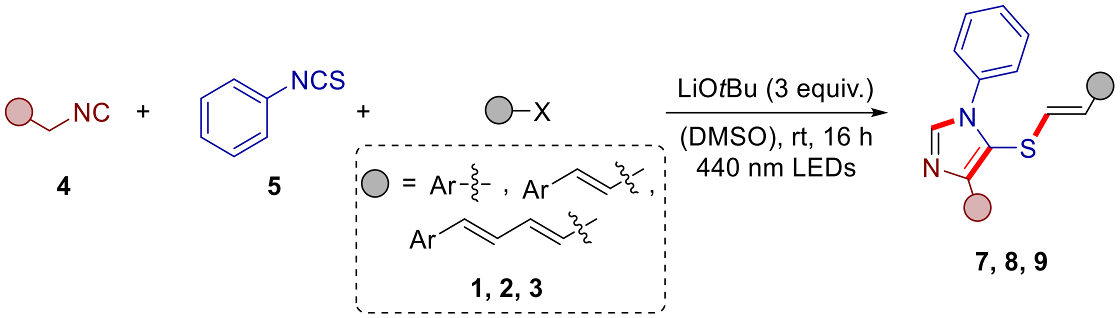

In a 5 mL glass vial, isocyanide **4** (0.50 mmol, 2.5 equiv.), isothiocyanate **5** (0.30 mmol, 1.5 equiv.), and lithium *tert*-butoxide (0.60 mmol, 3.0 equiv.) were dissolved in DMSO (2 ml). The resulting mixture was stirred under air for 30 min to generate the thiolate anion. Subsequently, the corresponding vinyl bromide **1,** dienyl bromide **2** or aryl iodide **3** (0.20 mmol, 1.0 equiv.) was added. The vial was sealed and positioned between two 440 nm Kessil® lamps at a distance of approximately 3 cm from each source. Both lamps were operated at full intensity under continuous ventilation, and the reaction was stirred for 16 h at room temperature. After completion, the reaction mixture was transferred to a separatory funnel and diluted with H_2O (5 ml) and EtOAc (5 ml). The aqueous layer was extracted with EtOAc (2 × 5 ml), and the combined organic extracts were washed with saturated NaCl solution (2 × 5 ml). The organic phase was dried over anhydrous Na_2SO_4, filtered, and concentrated under reduced pressure, followed by high vacuum. The crude product was analyzed to determine the *E/Z* diastereomeric ratio (*d.r.*) and subsequently purified by column chromatography to afford the desired products **7, 8** and **9**. The set up for the photochemical reaction is shown in Figure SI-4.

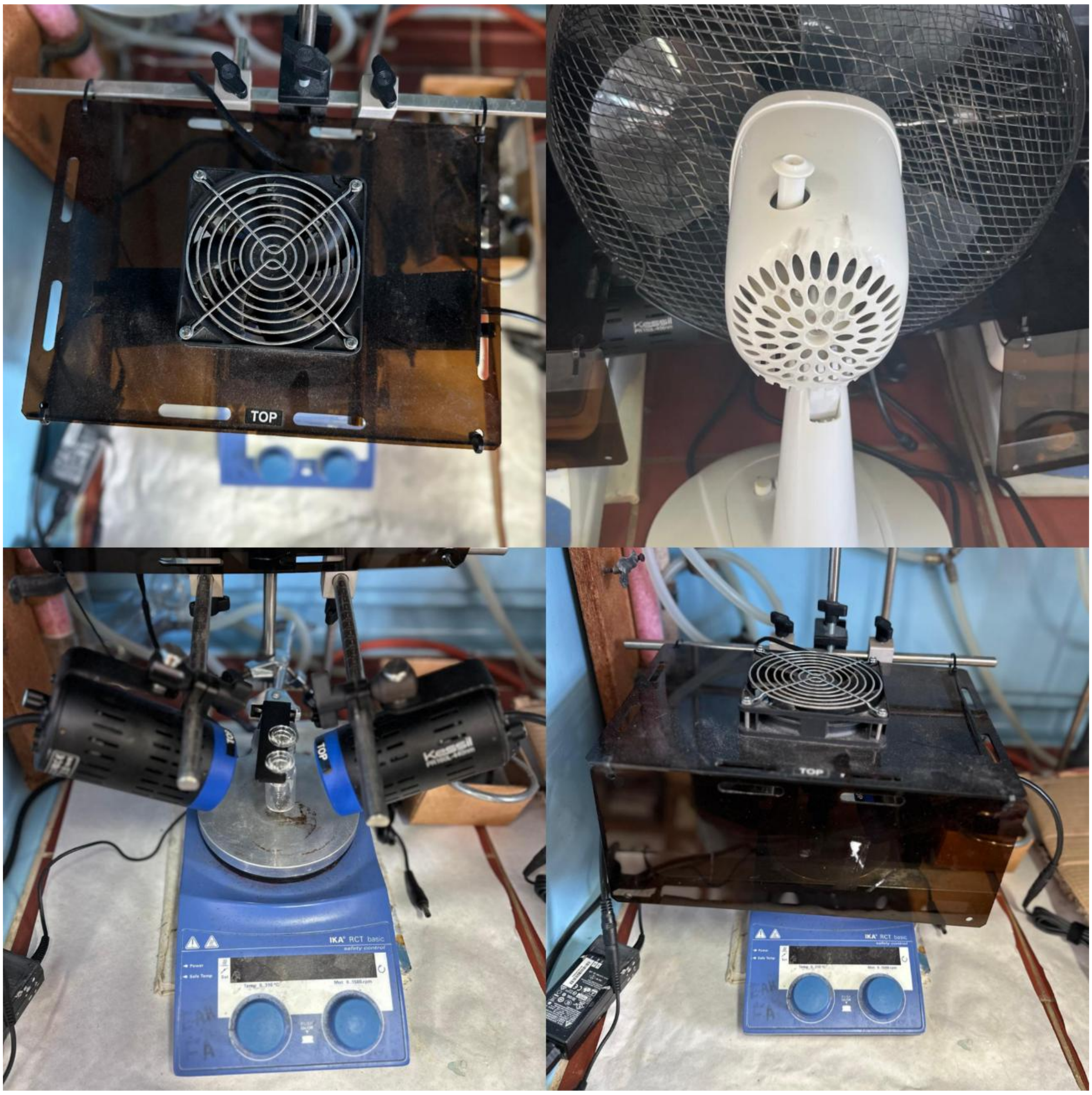

Figure SI-4. Set-up for the photochemical reaction.

Characterization data for the compounds 7, 8 and 9

1-phenyl-5-(styrylthio)-4-tosyl-1H-imidazole (7a)

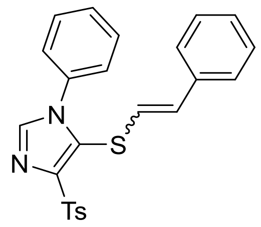

Following **General Procedure A**, isocyanide **4a** (98 mg, 0.50 mmol), isothiocyanate **5a** (36 μl, 0.30 mmol), and lithium *tert*-butoxide (48 mg, 0.60 mmol) were dissolved in DMSO. Subsequently, vinyl bromide **1a** (26 μl, 0.20 mmol) was added. After purification by column chromatography (Hex/EtOAc, 1:1), compound **7a** was obtained as an orange-yellow solid (81 mg, 94% isolated yield, *d.r.* = 0.6:1, *E/Z*).

Rf = 0.53 (*E isom.*), 0.47 (*Z isom.*). (1:1 Hex/EtOAc) [UV] [KMnO4].

In the ^{1}H RMN spectrum, both isomers are present, and the integrals of their olefinic signals were adjusted to one proton to determine the diastereomeric ratio. Consequently, all remaining integrals were normalized accordingly, and their combined values correspond to the total number of hydrogens in the molecule.

^{1}H RMN (300 MHz, $CDCl_3$, 300K): δ 8.03 – 8.00 (m, 2H), 7.80 (s, 1H, *E isom.*), 0.56 (s, 1H, *Z isom.*), 7.45 (dd, *J* = 5.1, 1.9 Hz, 1H), 7.39 – 7.35 (m, 2H), 7.31 – 7.20 (m, 7H), 7.06 – 7.00 (m, 1H), 6.41 (d, *J* = 10.4 Hz, 1H, *Z isom.*), 6.27 (d, *J* = 15.3 Hz, 1H, *E isom.*), 6.08 (d, *J* = 15.3 Hz, 1H, *E isom.*), 5.93 (d, *J* = 10.4 Hz, 1H, *Z isom.*), 2.39 (s, 3H, *Z isom.*), 2.31 (s, 3H, *E isom.*).

^{13}C{^{1}H} NMR (75 MHz, $CDCl_3$, 300K) δ 146.4 (C), 145.7 (C), 144.5 (C), 139.8 (CH), 139.5 (CH), 138.1 (C), 138.0 (C), 137.4 (C), 135.6 (C), 135.4 (C), 134.4 (C), 134.3 (C), 132.0(CH), 130.3 (CH), 129.9 (CH), 129.8 (CH), 129.8 (CH), 129.6 (CH), 129.5 (CH), 129.3 (CH), 128.9 (CH), 128.7 (CH), 128.6 (CH), 128.3 (CH), 128.0 (CH), 128.0 (CH), 127.6 (CH), 126.5 (CH), 126.4 (CH), 126.1 (CH), 124.4 (C), 123.7 (CH), 122.3 (CH), 121.9 (CH), 120.9 (CH), 21.7 (CH_3), 21.6 (CH_3).

HRMS [EI]: calcd. for $[C_{24}H_{20}N_2O_2S_2]^{+}$: 432.0961, found: 432.0971.

Ethyl 1-phenyl-5-(styrylthio)-1H-imidazole-4-carboxylate (7b)

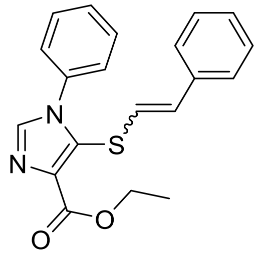

Following **General Procedure A**, isocyanide **4b** (55 μl, 0.50 mmol), isothiocyanate **5a** (36 μl, 0.30 mmol), and lithium *tert*-butoxide (48 mg, 0.60 mmol) were dissolved in DMSO. Subsequently, vinyl bromide **1a** (26 μl, 0.20 mmol) was added. After purification by column chromatography (Hex/EtOAc, 1:1), compound **7b** was obtained as a pale yellow solid (49 mg *E isom.* + 17 mg *Z isom.,* 94% isolated yield, *d.r.* = 2,9:1, *E/Z*).

Rf = 0.52 (*E isom.*), 0.40 (*Z isom.*). (1:1 Hex/EtOAc) [UV] [KMnO4].

In this case, both isomers were isolated separately, and their NMR analyses were performed independently.

- ***E isomer.***

^{1}H RMN (300 MHz, $CDCl_3$, 300K): δ 7.83 (s, 1H), 7.51 – 7.46 (m, 3H), 7.36 – 7.32 (m, 2H), 7.26 – 7.19 (m, 3H), 7.14 (dd, *J* = 8.0, 1.7 Hz, 2H), 6.47 (d, *J* = 15.4 Hz, 1H), 6.31 (d, *J* = 15.4 Hz, 1H), 4.43 (q, *J* = 7.1 Hz, 2H), 1.41 (t, *J* = 7.1 Hz, 3H).

^{13}C{^{1}H} NMR (75 MHz, $CDCl_3$, 300K) δ 162.1 (C), 139.6 (CH), 137.3 (C), 135.9 (C), 134.9 (C), 130.8 (CH), 129.6 (CH), 129.5 (CH), 128.6 (CH), 127.7 (CH), 127.0 (C), 126.5 (CH), 126.0 (CH), 121.8 (CH), 61.1 (CH_2), 14.4 (CH_3).

HRMS (EI): Calcd. for $[C_{20}H_{18}N_2O_2S]^+$: 350.1084, found: 350.1107.

- ***Z isomer.***

^{1}H RMN (300 MHz, $CDCl_3$, 300K): δ 7.78 (s, 1H), 7.44 – 7.40 (m, 3H), 7.38 – 7.20 (m, 7H), 6.43 (d, *J* = 10.5 Hz, 1H), 6.03 (d, *J* = 10.5 Hz, 1H), 4.42 (q, *J* = 7.1 Hz, 2H), 1.39 (t, *J* = 7.1 Hz, 3H).

$^{13}C\{^1H\}$ **NMR** (75 MHz, $CDCl_3$, 300K) δ 162.2 (C), 139.2 (CH), 136.5 (C), 135.7 (C), 134.8 (C), 129.5 (CH), 129.3 (CH), 129.1 (C), 128.7 (CH), 128.3 (C), 128.1 (CH), 127.4 (CH), 126.4 (CH), 124.6 (CH), 61.1 (CH_2), 14.4 (CH_3).

HRMS (EI): Calcd. for $[C_{20}H_{18}N_2O_2S]^+$: 350.1084, found: 350.1107.

Ethyl 5-((4-methylstyryl)thio)-1-phenyl-1H-imidazole-4-carboxylate (7c)

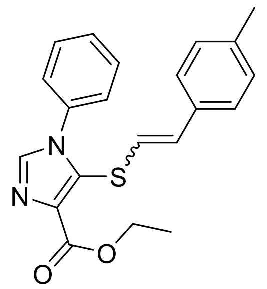

Following **General Procedure A**, isocyanide **4b** (55 μl, 0.50 mmol), isothiocyanate **5a** (36 μl, 0.30 mmol), and lithium *tert*-butoxide (48 mg, 0.60 mmol) were dissolved in DMSO. Subsequently, vinyl bromide **1b** (39 mg, 0.20 mmol) was added. After purification by column chromatography (Hex/EtOAc, 1:1), compound **7c** was obtained as a yellow solid (55 mg, 75% isolated yield, *d.r.* = 1,3:1, *E/Z*).

Rf = 0.46 (*E isom.*), 0.40 (*Z isom.*). (1:1 Hex/EtOAc) [UV] [KMnO4].

In the ^1H RMN spectrum, both isomers are present, and the integrals of their diagnostic signals were adjusted to one proton to determine the diastereomeric ratio. Consequently, all remaining integrals were normalized accordingly, and their combined values correspond to the total number of hydrogens in the molecule.

^1H **RMN** (300 MHz, $CDCl_3$, 300K): δ 7.80 (s, 1H, *E isom.*), 7.77 (s, 1H, *Z isom.*), 7.51 – 7.37 (m, 3H), 7.37 – 7.25 (m, 2H), 7.24 (d, *J* = 8.9 Hz, 1H), 7.09 (d, *J* = 7.9 Hz, 1H), 7.03 (s, 2H), 6.40 (d, *J* = 15.3 Hz, 1H, *E isom.*), 6.38 (d, *J* = 10.4 Hz, 1H, *Z isom.*), 6.29 (d, *J* = 15.4 Hz, 1H, *E isom.*), 5.96 (d, *J* = 10.5 Hz, 1H, *Z isom.*), 4.42 (q, *J* = 7.2 Hz, 2H, *E isom.*), 4.41 (q, *J* = 7.1 Hz, 1H, *Z isom.*), 2.31 (s, 3H, *Z isom.*), 2.28 (s, 3H, *E isom.*), 1.40 (t, *J* = 7.2 Hz, 3H, *E isom.*), 1.38 (t, *J* = 7.2 Hz, 3H, *Z isom.*).

$^{13}C\{^1H\}$ **NMR** (75 MHz, $CDCl_3$, 300K) δ 162.1 (C), 139.5 (CH), 139.2 (CH), 137.7 (C), 137.2 (C), 137.1 (C), 136.5 (C), 135.0 (C), 133.2 (C), 132.9 (C), 131.4 (CH), 129.5 (CH),

129.4 (CH), 129.3 (CH), 129.3 (CH), 128.9 (CH), 128.7 (CH), 128.1 (CH), 127.3 (C), 126.5 (CH), 126.4 (CH), 125.9 (CH), 123.5 (CH), 120.4 (CH), 61.0 (CH_2), 21.3 (CH_3), 21.2 (CH_3), 14.4 (CH_3), 14.4 (CH_3).

HRMS (EI): Calcd. for $[C_{21}H_{20}N_2O_2S]^+$: 364.1245, found: 364.1249.

Ethyl 5-((4-cyanostyryl)thio)-1-phenyl-1H-imidazole-4-carboxylate (7d)

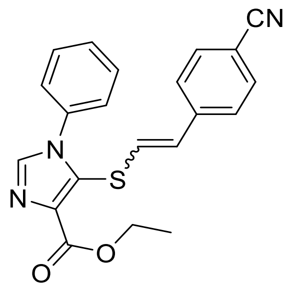

Following **General Procedure A**, isocyanide **4b** (55 µl mg, 0.50 mmol), isothiocyanate **5a** (36 µl, 0.30 mmol), and lithium *tert*-butoxide (48 mg, 0.60 mmol) were dissolved in DMSO. Subsequently, vinyl bromide **1c** (41 mg, 0.20 mmol) was added. After purification by column chromatography (Hex/EtOAc, 1:1), compound **$** was obtained as a pale yellow solid (60 mg, 80% isolated yield, *d.r.* = 0,8:1, *E/Z*).

Rf = 0.28 (*E isom.*), 0.22 (*Z isom.*). (1:1 Hex/EtOAc) [UV] [KMnO4].

In the ^{1}H RMN spectrum, both isomers are present, and the integrals of their diagnostic signals were adjusted to one proton to determine the diastereomeric ratio. Consequently, all remaining integrals were normalized accordingly, and their combined values correspond to the total number of hydrogens in the molecule.

^{1}H RMN (300 MHz, $CDCl_3$, 300K): δ 7.85 (s, 1H, *E isom.*), 7.80 (s, 1H, *Z isom*), 7.60 – 7.39 (m, 6H), 7.34 – 7.25 (m, 2H), 7.20 (d, *J* = 8.4 Hz, 1H), 6.65 (d, *J* = 15.5 Hz, 1H, *E isom.*), 6.40 (d, *J* = 10.7 Hz, 1H, *Z isom*), 6.24 (d, *J* = 10.8 Hz, 1H, *Z isom*), 6.23 (d, *J* = 15.3 Hz, 1H, *E isom.*), 4.40 (q, *J* = 7.1 Hz, 2H), 1.38 (t, *J* = 7.1 Hz, 3H, *E isom.*), 1.37 (t, *J* = 7.1 Hz, 3H, *Z isom.*).

$^{13}C\{^1H\}$ NMR (75 MHz, $CDCl_3$, 300K) δ 162.0 (C), 161.9 (C), 140.2 (C), 140.1 (C), 139.9 (CH), 139.5 (CH), 137.7 (C), 136.8 (C), 134.7 (C), 132.5 (CH), 132.1 (CH), 130.1 (CH), 129.7 (CH), 129.6 (CH), 129.5 (CH), 129.2 (CH), 129.1 (CH), 127.9 (C), 127.3 (CH),

127.2 (CH), 126.5 (CH), 126.4 (CH), 126.2 (CH), 126.0 (CH), 121.7 (C), 118.8 (C), 110.6 (CN), 110.5 (CN), 61.1 (CH_2), 14.4 (CH_3).

HRMS (EI): Calcd. for $[C_{21}H_{17}N_3O_2S]^+$: 375.1036, found: 375.1070.

Ethyl 5-((2-chloro-6-fluorostyryl)thio)-1-phenyl-1H-imidazole-4-carboxylate (7e)

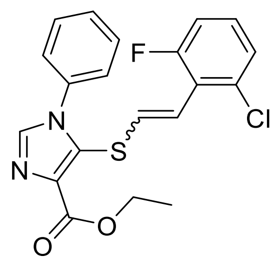

Following **General Procedure A**, isocyanide **4b** (55 µl mg, 0.50 mmol), isothiocyanate **5a** (36 µl, 0.30 mmol), and lithium *tert*-butoxide (48 mg, 0.60 mmol) were dissolved in DMSO. Subsequently, vinyl bromide **1d** (47 mg, 0.20 mmol) was added. After purification by column chromatography (Hex/EtOAc, 1:1), compound **7e** was obtained as a yellow oil (60 mg, 44% isolated yield, *d.r.* = 0,5:1, *E/Z*).

Rf = 0.52 (*E isom.*), 0.45 (*Z isom.*). (1:1 Hex/EtOAc) [UV] [KMnO4].

In the ^{1}H RMN spectrum, both isomers are present, and the integrals of their diagnostic signals were adjusted to one proton to determine the diastereomeric ratio. Consequently, all remaining integrals were normalized accordingly, and their combined values correspond to the total number of hydrogens in the molecule.

^{1}H RMN (300 MHz, $CDCl_3$, 300K): δ 7.86 (s, 1H, *E isom.*) , 7.76 (s, 1H, *Z isom.*), 7.48 (dd, *J* = 5.1, 2.0 Hz, 1H), 7.43 – 6.89 (m, 7H), 6.84 (d, *J* = 15.7 Hz, 1H, *E isom.*), 6.46 – 6.34 (m, 3H, *Z isom* X2 + *E isom.*), 4.43 (q, *J* = 7.1 Hz, 2H, *Z isom.*), 4.43 (q, *J* = 7.1 Hz, 2H, *E isom.*), 1.41 (t, *J* = 7.1 Hz, 3H, *Z isom.*), 1.41 (t, *J* = 7.1 Hz, 3H, *E isom.*).

^{13}C{^{1}H} NMR (75 MHz, $CDCl_3$, 300K) δ 162.2 (C), 160.5 (d, *J* = 250.2 Hz, C), 159.7 (d, *J* = 250.2 Hz), 139.9 (CH), 139.3 (CH), 137.8 (C), 136.9 (C), 134.8 (C), 134.4 (d, *J* = 6 Hz, C), 133.5 (d, *J* = 6 Hz, C), 130.8 (CH), 130.6 (CH), 129.6 (CH), 129.6 (CH), 129.5 (CH), 129.3 (CH), 129.2 (CH), 128.1 (CH), 127.9 (CH), 126.5 (CH), 126.3 (CH), 125.6 (d, *J* = 3.4 Hz, CH), 124.9 (d, *J* = 3.4 Hz, CH), 124.9 (CH), 122.7 (C), 122.4 (C), 120.2

(CH), 119.4 (CH), 114.5 (d, J = 23.4 Hz, CH), 114.1 (d, J = 22.9 Hz, CH), 61.1 (CH_2), 61.0 (CH_2), 14.4 (CH_3).

^{19}F RMN (282 MHz, $CDCl_3$, 300K): δ -107.43 (*Z isom*), -111.32 (*E isom*).

HRMS (EI): Calcd. for $[C_{20}H_{16}ClFN_2O_2S]^+$: 402.0600, found: 402.0632.

Ethyl 5-((4-nitrostyryl)thio)-1-phenyl-1H-imidazole-4-carboxylate (7f)

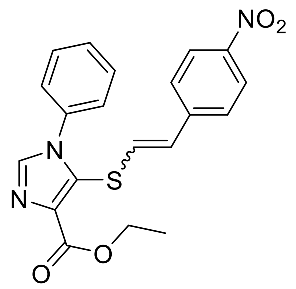

Following **General Procedure A**, isocyanide 4**b** (55 µl mg, 0.50 mmol), isothiocyanate **5a** (36 µl, 0.30 mmol), and lithium *tert*-butoxide (48 mg, 0.60 mmol) were dissolved in DMSO. Subsequently, vinyl bromide **1e** (46 mg, 0.20 mmol) was added. After purification by column chromatography (Hex/EtOAc, 1:1), compound **7f** was obtained as a orange oil (60 mg, 78% isolated yield, *d.r.* = 1,3:1, *E/Z*).

Rf = 0.52 (*E isom.*), 0.45 (*Z isom.*). (1:1 Hex/EtOAc) [UV] [KMnO4].

In the ^{1}H RMN spectrum, both isomers are present, and the integrals of their diagnostic signals were adjusted to one proton to determine the diastereomeric ratio. Consequently, all remaining integrals were normalized accordingly, and their combined values correspond to the total number of hydrogens in the molecule.

^{1}H RMN (300 MHz, $CDCl_3$, 300K): δ 8.16 (d, J = 8.8 Hz, 2H, *Z isom.*), 8.11 (d, J = 8.8 Hz, 2H, *E isom.*), 7.88 (s, 1H, *E isom.*), 7.82 (s, 1H, *Z isom.*), 7.49 (td, J = 7.0, 2.8 Hz, 3H), 7.38 – 7.22 (m, 4H), 6.74 (d, J = 15.5 Hz, 1H, *E isom.*), 6.47 (d, J = 10.7 Hz, 1H, *Z isom.*), 6.32 (d, J = 10.7 Hz, 1H, *Z isom.*), 6.29 (d, J = 15.5 Hz, 1H, *E isom.*), 4.43 (q, J = 7.1 Hz, 2H), 1.41 (t, J = 7.1 Hz, 3H, *E isom.*), 1.40 (t, J = 7.1 Hz, 3H, *Z isom.*).

^{13}C{^{1}H} NMR (75 MHz, $CDCl_3$, 300K) δ 161.9 (C), 146.6 (C), 146.3 (C), 142.2 (C), 142.1 (C), 139.9 (CH), 139.5 (CH), 137.8 (C), 136.9 (C), 134.7 (C), 130.2 (CH), 129.8 (CH), 129.6 (CH), 129.6 (CH), 129.2 (CH), 128.4 (CH), 127.8 (C), 126.6 (CH), 126.5 (CH),

126.4 (CH), 126.2 (CH), 125.6 (CH), 124.1 (CH), 123.7 (CH), 121.7 (CH), 61.2 (CH_2), 14.4 (CH_3).

HRMS (EI): Calcd. for $[C_{20}H_{17}N_3O_4S]^+$: 395.0940, found: 395.0972.

Ethyl 5-((4-(tert-butyl)styryl)thio)-1-phenyl-1H-imidazole-4-carboxylate (7g)

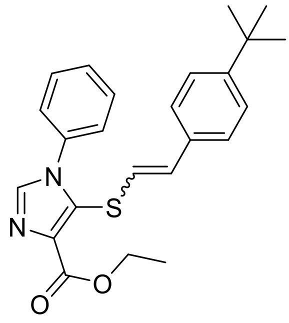

Following **General Procedure A**, isocyanide **4b** (55 µl mg, 0.50 mmol), isothiocyanate **5a** (36 µl, 0.30 mmol), and lithium *tert*-butoxide (48 mg, 0.60 mmol) were dissolved in DMSO. Subsequently, vinyl bromide **1f** (48 mg, 0.20 mmol) was added. After purification by column chromatography (Hex/EtOAc, 1:1), compound **7g** was obtained as a yellow oil (65 mg, 80% isolated yield, *d.r.* = 2,4:1, *E/Z*).

In the ^{1}H RMN spectrum, both isomers are present, and the integrals of their diagnostic signals were adjusted to one proton to determine the diastereomeric ratio. Consequently, all remaining integrals were normalized accordingly, and their combined values correspond to the total number of hydrogens in the molecule.

Rf = 0.42 (*E isom.*), 0.37 (*Z isom.*). (1:1 Hex/EtOAc) [UV] [KMnO4].

^{1}H RMN (300 MHz, $CDCl_3$, 300K): δ 7.82 (s, 1H, *E isom.*), 7.79 (s, 1H, *Z isom.*), 7.51 – 7.45 (m, 2H), 7.45 – 7.40 (m, 1H), 7.38 – 7.25 (m, 5H), 6.43 (d, *J* = 15.3 Hz, 1H, *E isom.*), 6.40 (d, *J* = 10.5 Hz, 1H, *Z isom.*), 6.31 (d, *J* = 15.3 Hz, 1H, *E isom.*), 5.98 (d, *J* = 10.5 Hz, 1H, *Z isom.*), 4.43 (q, *J* = 7.1 Hz, 2H, *E isom.*), 4.42 (q, *J* = 7.0 Hz, 2H, *Z isom.*), 1.41 (t, *J* = 7.2 Hz, 3H), 1.30 (s, 9H, *Z isom.*), 1.28 (s, 9H, *E isom.*).

^{13}C{^{1}H} NMR (75 MHz, $CDCl_3$, 300K) δ 162.1 (C), 150.9 (C), 150.4 (C), 139.5 (CH), 139.2 (CH), 137.3 (C), 136.5 (C), 135.0 (C), 133.2 (C), 132.9 (C), 131.1 (CH), 129.5 (CH), 129.5 (CH), 129.4 (CH), 129.3 (CH), 128.5 (CH), 128.0 (CH), 127.2 (C), 126.5 (CH),

126.4 (CH), 125.7 (CH), 125.5 (CH), 125.2 (CH), 123.6 (CH), 120.8 (CH), 61.0 (CH_2), 34.6 (C), 31.2 (CH_3), 14.4 (CH_3).

HRMS (EI): Calcd. for $[C_{24}H_{26}N_2O_2S]^+$: 406.1715, found: 406.1721.

Ethyl 5-((4-(methylthio)styryl)thio)-1-phenyl-1H-imidazole-4-carboxylate (7h)

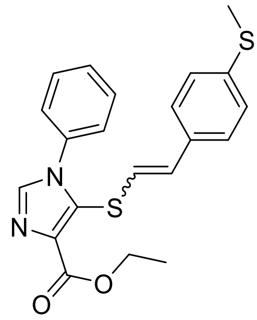

Following **General Procedure A**, isocyanide **4b** (55 µl mg, 0.50 mmol), isothiocyanate **5a** (36 µl, 0.30 mmol), and lithium *tert*-butoxide (48 mg, 0.60 mmol) were dissolved in DMSO. Subsequently, vinyl bromide **1g** (46 mg, 0.20 mmol) was added. After purification by column chromatography (Hex/EtOAc, 1:2), compound **7h** was obtained as a yellow oil (42 mg, 53% isolated yield, *d.r.* = 1,3:1, *E/Z*).

In the ^{1}H RMN spectrum, both isomers are present, and the integrals of their diagnostic signals were adjusted to one proton to determine the diastereomeric ratio. Consequently, all remaining integrals were normalized accordingly, and their combined values correspond to the total number of hydrogens in the molecule.

Rf = 0.41 (*E isom.*), 0.38 (*Z isom.*). (1:2 Hex/EtOAc) [UV] [KMnO4].

^{1}H RMN (300 MHz, $CDCl_3$, 300K): δ 7.81 (s, 1H, *E isom.*), 7.78 (s, 1H, *Z isom.*), 7.50 – 7.40 (m, 3H), 7.37 – 7.25 (m, 3H), 7.19 – 7.03 (m, 3H), 6.41 (d, *J* = 15.3 Hz, 1H, *E isom.*), 6.36 (d, *J* = 10.5 Hz, 1H, *Z isom.*), 6.26 (d, *J* = 15.3 Hz, 1H, *E isom.*), 5.99 (d, *J* = 10.5 Hz, 1H *Z isom.*), 4.42 (q, *J* = 7.1 Hz, 2H, *E isom.*), 4.41 (q, *J* = 7.1 Hz, 2H, *Z isom.*), 2.46 (s, 3H, *Z isom.*), 2.44 (s, 3H, *E isom.*), 1.41 (t, *J* = 6.8 Hz, 3H, *E isom.*), 1.38 (t, *J* = 6.8 Hz, 3H, *Z isom.*),

^{13}C{^{1}H} NMR (75 MHz, $CDCl_3$, 300K) δ 162.2 (C), 162.1 (C), 139.5 (CH), 139.2 (CH), 138.2 (C), 137.8 (C), 137.2 (C), 136.5 (C), 134.9 (C), 134.9 (C), 132.9 (C), 132.5 (C),

130.5 (CH), 129.5 (CH), 129.5 (CH), 129.4 (CH), 129.3 (CH), 129.1 (CH), 128.9 (C), 127.6 (CH), 127.1 (C), 126.5 (CH), 126.4 (CH), 126.4 (CH), 126.1 (CH), 124.0 (CH), 121.0 (CH), 61.0 (CH_2), 15.7 (CH_3), 15.6 (CH_3), 14.4 (CH_3), 14.4 (CH_3).

HRMS (EI): Calcd. for $[C_{21}H_{20}N_2O_2S_2]^{+}$: 396.0966, found: 396.0977.

Ethyl 5-((4-chlorostyryl)thio)-1-phenyl-1H-imidazole-4-carboxylate (7i)

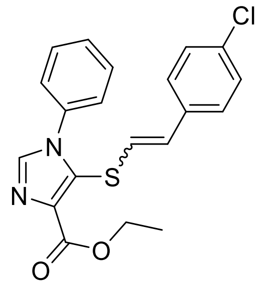

Following **General Procedure A**, isocyanide **4b** (55 µl mg, 0.50 mmol), isothiocyanate **5a** (36 µl, 0.30 mmol), and lithium *tert*-butoxide (48 mg, 0.60 mmol) were dissolved in DMSO. Subsequently, vinyl bromide **1h** (44 mg, 0.20 mmol) was added. After purification by column chromatography (Hex/EtOAc, 1:2), compound **7i** was obtained as a yellow oil (49 mg, 64% isolated yield, *d.r.* = 2,1:1, *E/Z*).

In the ^{1}H RMN spectrum, both isomers are present, and the integrals of their diagnostic signals were adjusted to one proton to determine the diastereomeric ratio. Consequently, all remaining integrals were normalized accordingly, and their combined values correspond to the total number of hydrogens in the molecule.

Rf = 0.64 (*E isom.*), 0.59 (*Z isom.*). (1:3 Hex/EtOAc) [UV] [KMnO4].

^{1}H RMN (300 MHz, $CDCl_3$, 300K): δ 7.82 (s, 1H, *E isom.*), 7.79 (s, 1H, isómero cis), 7.50 – 7.41 (m, 3H), 7.34 – 7.29 (m, 3H), 7.21 (d, *J* = 8.5 Hz, 1H + 1H *E isom.*), 7.06 (d, *J* = 8.5 Hz, 1H + 1H *Z isom.*), 6.45 (d, *J* = 15.4 Hz, 1H, *E isom.*), 6.37 (d, *J* = 10.6 Hz, 1H, *Z isom.*), 6.25 (d, *J* = 15.4 Hz, 1H, *E isom.*), 6.06 (d, *J* = 10.5 Hz, 1H, *Z isom.*), 4.42 (q, *J* = 7.1 Hz, 2H, *E isom.*), 4.41 (q, *J* = 7.1 Hz, 2H, *Z isom.*), 1.39 (t, *J* = 7.1 Hz, 3H, *E isom.*), 1.40 (t, *J* = 7.1 Hz, 3H, *Z isom.*).

^{13}C{^{1}H} NMR (75 MHz, $CDCl_3$, 300K) δ 162.7 (C), 140.3 (CH), 140.0 (CH), 138.0 (C), 137.3 (C), 135.5 (C), 135.1 (C), 134.8 (C), 134.0 (C), 133.7 (C), 130.6 (CH), 130.2 (CH),

130.2 (CH), 130.1 (CH), 130.1 (CH), 130.0 (CH), 129.5 (CH), 129.1 (CH), 127.8 (CH), 127.6 (CH), 127.4 (C), 127.1 (CH), 126.1 (CH), 123.4 (CH), 61.7 (CH_2), 15.0 (CH_3).

HRMS (EI): Calcd. for $[C_{20}H_{17}ClN_2O_2S]^+$: 384.0699, found: 384.0712.

Ethyl 5-((2-chlorostyryl)thio)-1-phenyl-1H-imidazole-4-carboxylate (7j)

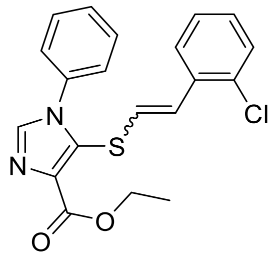

Following **General Procedure A**, isocyanide **4b** (55 µl mg, 0.50 mmol), isothiocyanate **5a** (36 µl, 0.30 mmol), and lithium *tert*-butoxide (48 mg, 0.60 mmol) were dissolved in DMSO. Subsequently, vinyl bromide **1i** (44 mg, 0.20 mmol) was added. After purification by column chromatography (Hex/EtOAc, 1:1), compound **7j** was obtained as a yellow oil (44 mg, 57% isolated yield, *d.r.* = 0,5:1, *E/Z*).

In the ^{1}H RMN spectrum, both isomers are present, and the integrals of their diagnostic signals were adjusted to one proton to determine the diastereomeric ratio. Consequently, all remaining integrals were normalized accordingly, and their combined values correspond to the total number of hydrogens in the molecule.

Rf = 0.75 (*E isom.*), 0.75 (*Z isom.*). (1:2 Hex/EtOAc) [UV] [KMnO4].

^{1}H RMN (300 MHz, $CDCl_3$, 300K): δ 7.83 (s, 1H, *E isom.*), 7.78 (s, 1H, *Z isom.*), 7.54 – 7.44 (m, 1H + 1H *Z isom.*), 7.47 – 7.37 (m, 2H), 7.40 – 7.29 (m, 1H + 1H *E isom.*), 7.31 – 7.22 (m, 2H), 7.22 – 7.08 (m, 2H), 6.66 (d, *J* = 15.4 Hz, 1H, *E isom.*), 6.65 (d, *J* = 10.4 Hz, 1H, *Z isom.*), 6.49 (d, *J* = 15.4 Hz, 1H, *E isom.*), 6.18 (d, *J* = 10.4 Hz, 1H, *Z isom.*), 4.43 (q, *J* = 7.1 Hz, 2H), 1.41 (t, *J* = 7.1 Hz, 3H, *E isom.*), 1.40 (t, *J* = 7.1 Hz, 3H, *Z isom.*).

^{13}C{^{1}H} NMR (75 MHz, $CDCl_3$, 300K) δ 162.2 (C), 162.1 (C), 139.7 (CH), 139.3 (CH), 137.4 (C), 136.7 (C), 134.8 (C), 134.8 (C), 134.1 (C), 133.6 (C), 133.3 (C), 132.3 (C), 129.8 (CH), 129.7 (CH), 129.6 (CH), 129.5 (CH), 129.4 (CH), 129.4 (CH), 128.8 (CH),

128.6 (CH), 128.4 (C), 127.0 (CH), 126.9 (CH), 126.5 (CH), 126.4 (CH), 126.4 (CH), 126.3 (CH), 125.4 (CH), 125.3 (CH), 61.1 (CH_2), 14.4 (CH_3).

HRMS (EI): Calcd. for $[C_{20}H_{17}ClN_2O_2S]^+$: 384.0699, found: 384.0723.

Ethyl 5-((3,5-dimethylstyryl)thio)-1-phenyl-1H-imidazole-4-carboxylate (7k)

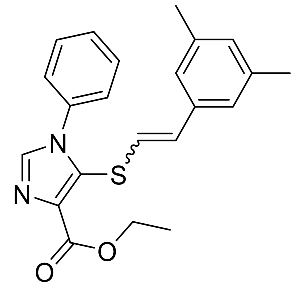

Following **General Procedure A**, isocyanide **4b** (55 μl mg, 0.50 mmol), isothiocyanate **5a** (36 μl, 0.30 mmol), and lithium *tert*-butoxide (48 mg, 0.60 mmol) were dissolved in DMSO. Subsequently, vinyl bromide **1j** (42 mg, 0.20 mmol) was added. After purification by column chromatography (Hex/EtOAc, 1:1), compound **7k** was obtained as a yellow oil (52 mg, 69% isolated yield, *d.r.* = 1,3:1, *E/Z*).

In the ^{1}H RMN spectrum, both isomers are present, and the integrals of their diagnostic signals were adjusted to one proton to determine the diastereomeric ratio. Consequently, all remaining integrals were normalized accordingly, and their combined values correspond to the total number of hydrogens in the molecule.

Rf = 0.80 (*E isom.*), 0.74 (*Z isom.*). (1:2 Hex/EtOAc) [UV] [KMnO4].

^{1}H RMN (300 MHz, $CDCl_3$, 300K): δ 7.82 (s, 1H, *E isom.*), 7.78 (s, 1H, *Z isom.*), 7.53 – 7.41 (m, 3H), 7.32 (td, *J* = 7.4, 3.4 Hz, 2H), 6.97 (s, 2H, *Z isom.*), 6.85 (d, *J* = 5.1 Hz, 1H), 6.77 (s, 2H, *E isom.*) 6.44 (d, *J* = 15.3 Hz, 1H, *E isom.*), 6.35 (d, *J* = 10.5 Hz, 1H, *Z isom.*), 6.26 (d, *J* = 15.4 Hz, 1H, *E isom.*), 5.98 (d, *J* = 10.5 Hz, 1H, *Z isom.*), 4.43 (q, *J* = 7.1 Hz, 2H, *E isom.*), 4.42 (q, *J* = 7.1 Hz, 2H, isómero cis), 2.27 (s, 6H, *Z isom.*), 2.25 (s, 6H, *E isom.*), 1.41 (t, *J* = 7.1 Hz, 3H, *E isom.*), 1.39 (t, *J* = 7.1 Hz, 3H, *Z isom.*).

^{13}C{^{1}H} NMR (75 MHz, $CDCl_3$, 300K) δ 162.2 (C), 162.1 (C), 139.5 (CH), 139.2 (CH), 138.1 (C), 137.7 (C), 137.3 (C), 136.5 (C), 135.9 (C), 135.6 (C), 135.0 (C), 131.2 (CH), 129.5 (CH), 129.4 (CH), 129.3 (CH), 129.1 (CH), 128.3 (CH), 127.2 (C), 126.5 (CH),

126.4 (CH), 124.1 (CH), 123.9 (CH), 121.3 (CH), 61.0 (CH), 21.3 (CH_2), 21.2 (CH_3), 14.4 (CH_3), 14.4 (CH_3).

HRMS (EI): Calcd. for $[C_{22}H_{22}N_2O_2S]^+$: 378.1402, found: 378.1417.

Ethyl 5-((2-methylstyryl)thio)-1-phenyl-1H-imidazole-4-carboxylate (7l)

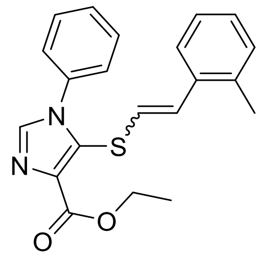

Following **General Procedure A**, isocyanide **4b** (55 µl mg, 0.50 mmol), isothiocyanate **5a** (36 µl, 0.30 mmol), and lithium *tert*-butoxide (48 mg, 0.60 mmol) were dissolved in DMSO. Subsequently, vinyl bromide **1k** (39 mg, 0.20 mmol) was added. After purification by column chromatography (Hex/EtOAc, 1:1), compound **7l** was obtained as a pale yellow oil (21 mg, 29% isolated yield, *d.r.* = 0,4:1, *E/Z*).

In the ^{1}H RMN spectrum, both isomers are present, and the integrals of their diagnostic signals were adjusted to one proton to determine the diastereomeric ratio. Consequently, all remaining integrals were normalized accordingly, and their combined values correspond to the total number of hydrogens in the molecule.

Rf = 0.73 (*E isom.*), 0.69 (*Z isom.*). (1:2 Hex/EtOAc) [UV] [KMnO4].

^{1}H RMN (300 MHz, $CDCl_3$, 300K): δ 7.81 (s, 1H, *E isom.*), 7.77 (s, 1H, *Z isom.*), 7.53 – 7.25 (m, 6H), 7.17 – 7.07 (m, 3H), 6.58 (d, *J* = 15.2 Hz, 1H, *E isom.*), 6.50 (d, *J* = 10.2 Hz, 1H, *Z isom.*), 6.34 (d, *J* = 15.2 Hz, 1H, *E isom.*), 6.08 (d, *J* = 10.2 Hz, 1H, *Z isom.*), 4.44 (q, *J* = 7.1 Hz, 2H), 2.18 (s, 3H, *E isom.*), 2.15 (s, 3H, *Z isom.*), 1.42 (t, *J* = 7.1 Hz, 3H, *E isom.*), 1.41 (t, *J* = 7.1 Hz, 3H, *Z isom.*).

^{13}C{^{1}H} NMR (75 MHz, $CDCl_3$, 300K) δ 162.2 (C), 139.5 (C), 139.2 (CH), 136.4 (C), 136.0 (C), 134.9 (C), 134.5 (C), 130.3 (CH), 130.0 (CH), 129.5 (CH), 129.4 (CH), 129.3 (CH), 129.2 (CH), 129.1 (C), 128.4 (CH), 127.7 (CH), 127.2 (CH), 126.5 (CH), 126.4 (CH),

126.1 (C), 125.5 (CH), 125.4 (CH), 125.3 (CH), 122.8 (CH), 61.0 (CH_2), 61.0 (CH_2), 19.7 (CH_3), 19.7 (CH_3), 14.4 (CH_3).

HRMS (EI): Calcd. for $[C_{21}H_{20}N_2O_2S]^+$: 364.1245, found: 364.1254.

Ethyl 5-((3-bromostyryl)thio)-1-phenyl-1H-imidazole-4-carboxylate (7m)

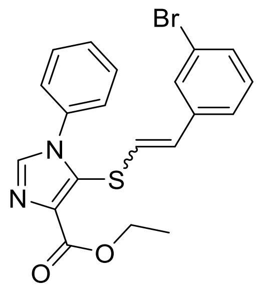

Following **General Procedure A**, isocyanide **4b** (55 μl mg, 0.50 mmol), isothiocyanate **5a** (36 μl, 0.30 mmol), and lithium *tert*-butoxide (48 mg, 0.60 mmol) were dissolved in DMSO. Subsequently, vinyl bromide **1l** (52 mg, 0.20 mmol) was added. After purification by column chromatography (Hex/EtOAc, 1:2), compound **7m** was obtained as a yellow oil (43 mg, 50% isolated yield, *d.r.* = 1,4:1, *E/Z*).

In the ^{1}H RMN spectrum, both isomers are present, and the integrals of their diagnostic signals were adjusted to one proton to determine the diastereomeric ratio. Consequently, all remaining integrals were normalized accordingly, and their combined values correspond to the total number of hydrogens in the molecule.

Rf = 0.71 (*E isom.*), 0.68 (*Z isom.*). (1:2 Hex/EtOAc) [UV] [KMnO4].

^{1}H RMN (300 MHz, $CDCl_3$, 300K): δ 7.83 (s, 1H, *E isom.*), 7.79 (s, 1H, *Z isom.*), 7.53 – 7.40 (m, 3H + 1H *E isom.*), 7.37 – 7.23 (m, 3H + 1H *Z isom.*), 7.21 – 7.00 (m, 2H), 6.48 (d, *J* = 15.4 Hz, 1H, *E isom.*), 6.33 (d, *J* = 10.5 Hz, 1H, *Z isom.*), 6.20 (d, *J* = 15.4 Hz, 1H, *E isom.*), 6.09 (d, *J* = 10.5 Hz, 1H, *Z isom.*), 4.42 (q, *J* = 7.1 Hz, 2H, *E isom.*), 4.42 (q, *J* = 7.1 Hz, 2H, *Z isom.*), 1.40 (t, *J* = 7.2 Hz, 3H, *E isom.*), 1.39 (t, *J* = 7.1 Hz, 3H, *Z isom.*).

$^{13}C\{^1H\}$ NMR (75 MHz, $CDCl_3$, 300K) δ 162.1 (C), 162.0 (C), 139.7 (CH), 139.3 (CH), 138.0 (C), 137.7 (C), 137.4 (C), 136.6 (C), 134.8 (C), 134.7 (C), 131.4 (CH), 130.4 (CH), 130.3 (CH), 130.1 (CH), 129.8 (CH), 129.6 (CH), 129.5 (CH), 129.5 (CH), 128.7 (CH),

128.5 (CH), 127.2 (CH), 126.6 (CH), 126.5 (CH), 126.4 (CH), 124.5 (CH), 124.0 (CH), 122.8 (C), 122.4 (C), 61.1 (CH_2), 14.4 (CH_3).

HRMS (EI): Calcd. for $[C_{20}H_{17}BrN_2O_2S]^{+}$: 428.0194, found: 428.0211.

Ethyl 5-((3,5-dibromostyryl)thio)-1-phenyl-1H-imidazole-4-carboxylate (7n)

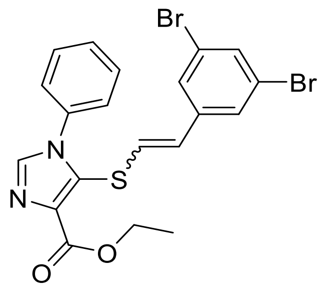

Following **General Procedure A**, isocyanide **4b** (55 µl mg, 0.50 mmol), isothiocyanate **5a** (36 µl, 0.30 mmol), and lithium *tert*-butoxide (48 mg, 0.60 mmol) were dissolved in DMSO. Subsequently, vinyl bromide **1m** (68 mg, 0.20 mmol) was added. After purification by column chromatography (Hex/EtOAc, 1:1), compound **7n** was obtained as a orange oil (28 mg, 28% isolated yield, *d.r.* = 1,8:1, *E/Z*).

In the ^{1}H RMN spectrum, both isomers are present, and the integrals of their diagnostic signals were adjusted to one proton to determine the diastereomeric ratio. Consequently, all remaining integrals were normalized accordingly, and their combined values correspond to the total number of hydrogens in the molecule.

Rf = 0.81 (*E isom.*), 0.75 (*Z isom.*). (1:3 Hex/EtOAc) [UV] [KMnO4].

^{1}H RMN (300 MHz, $CDCl_3$, 300K): δ 7.85 (s, 1H, *E isom.*), 7.81 (s, 1H, *Z isom.*), 7.55 – 7.42 (m, 4H), 7.41 (d, *J* = 1.7 Hz, 2H, *Z isom.*), 7.37 – 7.25 (m, 2H), 7.19 (d, *J* = 1.7 Hz, 2H, *E isom.*), 6.52 (d, *J* = 15.4 Hz, 1H, *E isom.*), 6.27 (d, *J* = 10.6 Hz, 1H, *Z isom.*), 6.15 (d, *J* = 11.0 Hz, 1H, *Z isom.*), 6.11 (d, *J* = 15.6 Hz, 1H, *E isom.*), 4.43 (q, *J* = 7.1 Hz, 2H), 1.42 (t, *J* = 7.1 Hz, 3H, *E isom.*), 1.41 (t, *J* = 7.1 Hz, 3H, *Z isom.*).

^{13}C{^{1}H} NMR (75 MHz, $CDCl_3$, 300K) δ 162.7 (C), 140.6 (CH), 140.2 (C), 140.1 (C), 139.7 (C), 138.3 (C), 135.4 (C), 133.4 (CH), 130.8 (CH), 130.5 (CH), 130.4 (C), 130.3 (CH), 129.0 (CH), 128.2 (CH), 127.2 (C), 127.1 (CH), 126.8 (CH), 126.6 (C), 126.0 (CH), 123.9 (C), 123.5 (C), 61.9 (CH_2), 15.1 (CH_3).

HRMS (EI): Calcd. for $[C_{20}H_{16}Br_2N_2O_2S]^+$: 505.9299, found: 505.9306.

Ethyl 5-((2-(naphthalen-2-yl)vinyl)thio)-1-phenyl-1H-imidazole-4-carboxylate (7o)

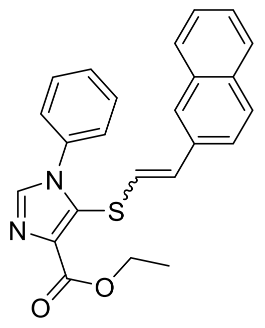

Following **General Procedure A**, isocyanide **4b** (55 μl mg, 0.50 mmol), isothiocyanate **5a** (36 μl, 0.30 mmol), and lithium *tert*-butoxide (48 mg, 0.60 mmol) were dissolved in DMSO. Subsequently, vinyl bromide **1n** (47 mg, 0.20 mmol) was added. After purification by column chromatography (Hex/EtOAc, 1:2), compound **7o** was obtained as a orange oil (42 mg, 52% isolated yield, *d.r.* = 0.9:1, *E/Z*).

In the ^{1}H RMN spectrum, both isomers are present, and the integrals of their diagnostic signals were adjusted to one proton to determine the diastereomeric ratio. Consequently, all remaining integrals were normalized accordingly, and their combined values correspond to the total number of hydrogens in the molecule.

Rf = 0.68 (*E isom.*), 0.64 (*Z isom.*). (1:3 Hex/EtOAc) [UV] [KMnO4].

^{1}H RMN (300 MHz, $CDCl_3$, 300K): δ 7.88 – 7.67 (m, 4H), 7.53 – 7.28 (m, 8H), 6.60 (d, *J* = 15.4 Hz, 1H, *E isom.*), 6.57 (d, *J* = 10.5 Hz, 1H, *Z isom.*), 6.47 (d, *J* = 15.3 Hz, 1H, *E isom.*), 6.12 (d, *J* = 10.5 Hz, 1H, *Z isom.*), 4.45 (q, *J* = 7.1 Hz, 2H, *E isom.*), 4.44 (q, *J* = 7.1 Hz, 2H, *Z isom.*), 1.42 (t, *J* = 6.8 Hz, 3H, *E isom.*), 1.40 (t, *J* = 6.9 Hz, 3H, *Z isom.*).

$^{13}C\{^1H\}$ NMR (75 MHz, $CDCl_3$, 300K) δ 162.2 (C), 162.1 (C), 139.6 (CH), 139.3 (CH), 137.3 (C), 136.6 (C), 135.0 (C), 134.9 (C), 133.5 (C), 133.4 (C), 133.3 (C), 133.2 (C), 132.9 (C), 132.5 (C), 130.9 (CH), 129.5 (CH), 129.5 (CH), 129.3 (CH), 129.0 (C) (CH), 128.3 (CH), 128.2 (CH), 128.2 (CH), 128.0 (C), 127.9 (CH), 127.8 (CH), 127.7 (CH), 127.6 (CH), 126.6 (CH), 126.5 (CH), 126.4 (CH), 126.4 (CH), 126.2 (CH), 126.1 (CH), 125.9 (CH), 125.0 (CH), 123.0 (CH), 122.2 (CH), 61.1 (CH_2), 14.4 (CH_3), 14.4 (CH_3).

HRMS (EI): Calcd. for $[C_{24}H_{20}N_2O_2S]^+$: 400.1245, found: 400.1249.

Ethyl 1-phenyl-5-((2-(thiophen-2-yl)vinyl)thio)-1H-imidazole-4-carboxylate (7p)

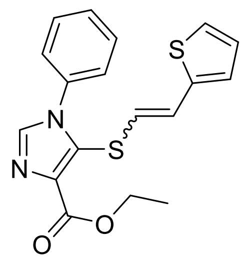

Following **General Procedure A**, isocyanide **4b** (55 μl mg, 0.50 mmol), isothiocyanate **5a** (36 μl, 0.30 mmol), and lithium *tert*-butoxide (48 mg, 0.60 mmol) were dissolved in DMSO. Subsequently, vinyl bromide **1o** (47 mg, 0.20 mmol) was added. After purification by column chromatography (Hex/EtOAc, 1:2), compound **7p** was obtained as a yellow oil (38 mg, 53% isolated yield, *d.r.* = 0.8:1, *E/Z*).

In the ^{1}H RMN spectrum, both isomers are present, and the integrals of their diagnostic signals were adjusted to one proton to determine the diastereomeric ratio. Consequently, all remaining integrals were normalized accordingly, and their combined values correspond to the total number of hydrogens in the molecule.

Rf = 0.52 (*E isom.*), 0.48 (*Z isom.*). (1:2 Hex/EtOAc) [UV] [KMnO4].

^{1}H RMN (300 MHz, $CDCl_3$, 300K): δ 7.81 (s, 1H, *E isom.*), 7.79 (s, 1H, *Z isom.*), 7.50 – 7.45 (m, 1H + 1H *E isom.*), 7.42 – 7.39 (m, 1H + 1H *Z isom.*), 7.35 – 7.25 (m, 2H + 1H *Z isom.*), 7.10 (d, *J* = 5.1 Hz, 1H, *E isom.*), 7.04 (d, *J* = 3.2 Hz, 1H, *Z isom.*), 6.97 (dd, *J* = 5.1, 3.6 Hz, 1H, *Z isom.*), 6.89 (dd, *J* = 5.1, 3.5 Hz, 1H, *E isom.*), 6.77 (d, *J* = 3.6 Hz, 1H, *E isom.*), 6.60 (d, *J* = 10.2 Hz, 1H, *Z isom.*), 6.44 (d, *J* = 15.2 Hz, 1H, *E isom.*), 6.27 (d, *J* = 15.1 Hz, 1H, *E isom.*), 5.94 (d, *J* = 10.2 Hz, 1H, *Z isom.*), 4.43 (q, *J* = 7.2 Hz, 2H, *E isom.*), 4.41 (q, *J* = 7.1 Hz, 2H, *Z isom.*), 1.41 (t, *J* = 7.1 Hz, 3H), 1.38 (t, *J* = 7.1 Hz, 1H).

$^{13}C\{^1H\}$ NMR (75 MHz, $CDCl_3$, 300K) δ 162.2 (C), 162.1 (C), 140.7 (C), 139.6 (CH), 139.3 (CH), 139.1 (C), 137.2 (C), 136.5 (C), 134.9 (C), 129.6 (CH), 129.5 (CH), 129.3 (CH), 128.4 (CH), 127.4 (CH), 126.8 (CH), 126.5 (CH), 126.4 (CH), 125.5 (CH), 124.8 (CH), 124.6 (CH), 121.9 (CH), 121.6 (CH), 120.6 (CH), 61.1 (CH_2), 14.4 (CH_3), 14.3 (CH_3).

HRMS (EI): Calcd. for $[C_{18}H_{16}N_2O_2S_2]^+$: 356.0653, found: 356.0667.

Ethyl 5-((2-(benzofuran-2-yl)vinyl)thio)-1-phenyl-1H-imidazole-4-carboxylate (7q)

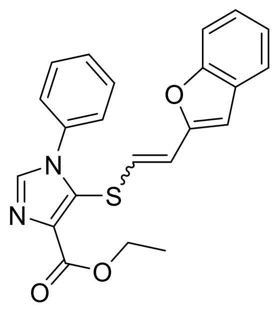

Following **General Procedure A**, isocyanide **4b** (55 μl mg, 0.50 mmol), isothiocyanate **5a** (36 μl, 0.30 mmol), and lithium *tert*-butoxide (48 mg, 0.60 mmol) were dissolved in DMSO. Subsequently, vinyl bromide **1p** (45 mg, 0.20 mmol) was added. After purification by column chromatography (Hex/EtOAc, 1:2), compound **7q** was obtained as a brown oil (58 mg, 69% isolated yield, *d.r.* = 1:1, *E/Z*).

In the ^{1}H RMN spectrum, both isomers are present, and the integrals of their diagnostic signals were adjusted to one proton to determine the diastereomeric ratio. Consequently, all remaining integrals were normalized accordingly, and their combined values correspond to the total number of hydrogens in the molecule.

Rf = 0.31 (*E isom.*), 0.28 (*Z isom.*). (1:2 Hex/EtOAc) [UV] [KMnO4].

^{1}H RMN (300 MHz, $CDCl_3$, 300K): δ 7.87 (s, 1H, *E isom.*), 7.81 (s, 1H, *Z isom.*), 7.51 – 7.40 (m, 5H), 7.36 – 7.31 (m, 2H), 7.28 – 7.13 (m, 2H), 6.74 (d, *J* = 15.8 Hz, 1H, *E isom.*), 6.71 (s, 1H, *Z isom.*), 6.39 (s, 1H, *E isom.*), 6.38 (d, *J* = 10.8 Hz, 1H, is *Z isom.*), 6.15 (d, *J* = 15.1 Hz, 1H, *E isom.*), 6.15 (d, *J* = 10.8 Hz, 1H), 4.42 (q, *J* = 7.1 Hz, 2H), 1.40 (t, *J* = 7.1 Hz, 3H, *Z isom.*), 1.38 (t, *J* = 7.2 Hz, 3H, *E isom.*).

$^{13}C\{^1H\}$ NMR (75 MHz, $CDCl_3$, 300K) δ 162.1 (C), 161.9 (C), 154.7 (C), 154.4 (C), 153.2 (C), 153.1 (C), 139.9 (CH), 139.4 (CH), 138.0 (C), 136.6 (C), 136.3 (C), 134.8 (C), 134.7 (C), 130.1 (CH), 129.6 (CH), 129.6 (CH), 129.5 (CH), 128.8 (C), 128.7 (C), 127.4 (CH), 126.5 (CH), 126.4 (CH), 125.7 (C), 124.8 (CH), 124.6 (CH), 122.9 (CH), 121.7 (CH), 121.0 (CH), 120.9 (CH), 117.4 (CH), 115.4 (CH), 111.2 (CH), 110.8 (CH), 106.3 (CH), 103.9 (CH), 61.1 (CH_2), 61.1 (CH_2), 14.4 (CH_3).

HRMS (EI): Calcd. for $[C_{22}H_{18}N_2O_3S]^+$: 390.1038, found: 390.1049.

Ethyl 1-(5-fluoro-2-methylphenyl)-5-(styrylthio)-1H-imidazole-4-carboxylate (7r)

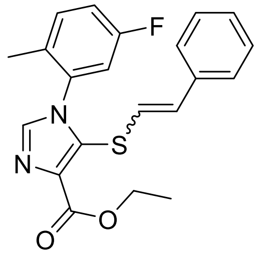

Following **General Procedure A**, isocyanide **4b** (55 μl mg, 0.50 mmol), isothiocyanate **5b** (42 μl, 0.30 mmol), and lithium *tert*-butoxide (48 mg, 0.60 mmol) were dissolved in DMSO. Subsequently, vinyl bromide **1a** (25 μl, 0.20 mmol) was added. After purification by column chromatography (Hex/EtOAc, 1:1), compound **7r** was obtained as a yellow oil (37 mg, 48% isolated yield, *d.r.* = 3:1, *E/Z*).

In the ^{1}H RMN spectrum, both isomers are present, and the integrals of their diagnostic signals were adjusted to one proton to determine the diastereomeric ratio. Consequently, all remaining integrals were normalized accordingly, and their combined values correspond to the total number of hydrogens in the molecule.

Rf = 0.28 (*E isom.*), 0.24 (*Z isom.*). (1:1 Hex/EtOAc) [UV] [KMnO4].

^{1}H RMN (300 MHz, $CDCl_3$, 300K): δ 7.67 (s, 1H, *E isom.*), 7.65 (s, 1H, *Z isom.*), 7.31 – 7.19 (m, 4H + 1H *Z isom.*), 7.17 – 7.05 (m, 2H + 1H *E isom.*), 6.92 (td, *J* = 9.1, 2.7 Hz, 1H), 6.46 (d, *J* = 15.3 Hz, 1H, *E isom.*), 6.44 (d, *J* = 10.6 Hz, 1H, *Z isom.*), 6.35 (d, *J* = 15.4 Hz, 1H, *E isom.*), 6.04 (d, *J* = 10.5 Hz, 1H, *Z isom.*), 4.43 (q, *J* = 7.2 Hz, 2H, *E isom.*), 4.42 (q, *J* = 7.0 Hz, 2H, *Z isom.*), 2.00 (s, 3H, *E isom.*), 1.99 (s, 3H, *Z isom.*), 1.42 (t, *J* = 7.1 Hz, 3H, *E isom.*), 1.39 (t, *J* = 7.1 Hz, 3H, *Z isom.*).

$^{13}C\{^1H\}$ NMR (75 MHz, $CDCl_3$, 300K) δ 162.1 (C), 160.7 (d, *J* = 247.8 Hz, C), 139.2 (CH), 139.0 (CH), 136.6 (C), 135.7 (C), 135.5 (C), 134.7 (d, *J* = 9.4 Hz, C), 132.3 (CH), 132.1 (d, *J* = 8.5 Hz, CH), 131.6 (d, *J* = 3.9 Hz, C), 128.7 (C), 128.2 (C), 128.0 (CH), 127.9 (CH), 127.4 (CH), 126.0 (CH), 123.8 (CH), 120.8 (CH), 117.3 (d, *J* = 20.6 Hz, CH), 115.6 (d, *J* = 23.3 Hz, CH), 61.1 (CH_2), 16.9 (CH_3), 14.4 (CH_3), 14.3 (CH_3).

^{19}**F RMN** (282 MHz, $CDCl_3$, 300K): δ -114.80.

HRMS (EI): Calcd. for $[C_{21}H_{19}FN_2O_2S]^+$: 382.1151, found: 382.1173.

Ethyl 1-(5-fluoro-2-methylphenyl)-5-((4-methylstyryl)thio)-1H-imidazole-4-carboxylate (7s)

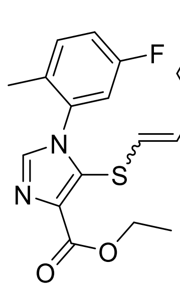

Following **General Procedure A**, isocyanide **4b** (55 µl mg, 0.50 mmol), isothiocyanate **5b** (42 µl, 0.30 mmol), and lithium *tert*-butoxide (48 mg, 0.60 mmol) were dissolved in DMSO. Subsequently, vinyl bromide **1b** (39 mg, 0.20 mmol) was added. After purification by column chromatography (Hex/EtOAc, 1:1), compound **7s** was obtained as a yellow oil (54 mg *E isom.* + 22 mg *Z isom.,* 96% isolated yield, *d.r.* = 2,4:1, *E/Z*).

In this case, both isomers were isolated separately, and their NMR analyses were performed independently.

Rf = 0.50 (*E isom.*), 0.42 (*Z isom.*). (1:1 Hex/EtOAc) [UV] [KMnO4].

- ***E isomer.***

^{1}**H RMN** (300 MHz, $CDCl_3$, 300K): δ 7.66 (s, 1H), 7.31 – 7.24 (m, 1H), 7.16 – 7.01 (m, 5H), 6.94 (dd, *J* = 8.4, 2.7 Hz, 1H), 6.40 (d, *J* = 15.3 Hz, 1H), 6.33 (d, *J* = 15.3 Hz, 1H), 4.44 (q, *J* = 7.1 Hz, 2H), 2.31 (s, 3H), 2.01 (s, 3H), 1.43 (t, *J* = 7.1 Hz, 3H).

^{13}**C{^{1}H} NMR** (75 MHz, $CDCl_3$, 300K) δ 162.0 (C), 160.7 (d, *J* = 247.8 Hz, C), 139.1 (CH), 138.0 (C), 136.4 (C), 134.7 (C), 133.0 (C), 132.9 (CH), 132.1 (d, *J* = 8.4 Hz, CH), 131.6 d, *J* = 3.4 Hz, C), 129.3 (CH), 128.3 (C), 126.0 (CH), 119.4 (CH), 117.3 (d, *J* = 20.6 Hz, CH), 115.6 (d, *J* = 23.4 Hz, CH), 61.1 (CH_2), 21.2 (CH_3), 16.9 (CH_3), 14.4 (CH_3).

^{19}**F RMN** (282 MHz, $CDCl_3$, 300K): δ -114.86.

HRMS (EI): Calcd. for $[C_{22}H_{21}FN_2O_2S]^+$: 396.1308, found: 396.1325.

- ***Z isomer.***

^{1}H RMN (300 MHz, $CDCl_3$, 300K): δ 7.65 (s, 1H), 7.29 – 7.16 (m, 3H), 7.14 – 7.03 (m, 3H), 6.91 (dd, *J* = 8.4, 2.7 Hz, 1H), 6.41 (d, *J* = 10.4 Hz, 1H), 5.98 (d, *J* = 10.4 Hz, 1H), 4.43 (q, *J* = 7.2 Hz, 2H), 2.32 (s, 3H), 2.00 (s, 3H), 1.40 (t, *J* = 7.1 Hz, 3H).

^{13}C{^{1}H} NMR (75 MHz, $CDCl_3$, 300K) δ 162.1 (C), 160.7 (d, *J* = 247.4 Hz, C), 138.9 (CH), 137.3 (C), 136.0 (C), 132.8 (C), 132.2 (CH), 132.1 (d, *J* = 8.5 Hz, CH), 131.5 (d, *J* = 3.4 Hz, C), 129.9 (C), 128.9 (CH), 128.6 (CH), 122.7 (CH), 117.2 (d, *J* = 20.4 Hz, CH), 115.6 (d, *J* = 23.3 Hz, CH), 61.1 (CH_2), 21.2 (CH_3), 16.9 (CH_3), 14.3 (CH_3).

^{19}F RMN (282 MHz, $CDCl_3$, 300K): δ -114.88.

HRMS (EI): Calcd. for $[C_{22}H_{21}FN_2O_2S]^+$: 396.1308, found: 396.1325.

Ethyl 5-((4-(tert-butyl)styryl)thio)-1-(5-fluoro-2-methylphenyl)-1H-imidazole-4-carboxylate (7t)

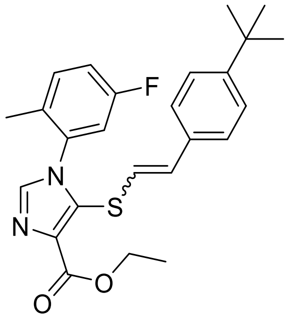

Following **General Procedure A**, isocyanide **4b** (55 μl mg, 0.50 mmol), isothiocyanate **5b** (42 μl, 0.30 mmol), and lithium *tert*-butoxide (48 mg, 0.60 mmol) were dissolved in DMSO. Subsequently, vinyl bromide **1f** (48 mg, 0.20 mmol) was added. After purification by column chromatography (Hex/EtOAc, 1:1), compound **7t** was obtained as a yellow oil (51 mg, 58% isolated yield, *d.r.* = 0,6:1, *E/Z*).

In the ^{1}H RMN spectrum, both isomers are present, and the integrals of their diagnostic signals were adjusted to one proton to determine the diastereomeric ratio. Consequently,

all remaining integrals were normalized accordingly, and their combined values correspond to the total number of hydrogens in the molecule.

Rf = 0.50 (*E isom.*), 0.47 (*Z isom.*). (1:1 Hex/EtOAc) [UV] [KMnO4].

^{1}H RMN (300 MHz, $CDCl_3$, 300K): δ 7.67 (s, 1H, *E isom.*), 7.65 (s, 1H, *Z isom.*), 7.33 – 7.21 (m, 4H), 7.16 – 7.03 (m, 2H), 6.97 – 6.88 (m, 1H), 6.42 (d, *J* = 10.6 Hz, 1H, *Z isom.*), 6.42 (d, *J* = 15.3 Hz, 1H, *E isom.*), 6.35 (d, *J* = 15.3 Hz, 1H, E isom.), 5.99 (d, *J* = 10.4 Hz, 1H, *Z isom.*), 4.49 – 4.38 (m, 2H), 2.01 (s, 3H, *E isom.*), 2.00 (s, 3H, *Z isom.*), 1.43 (t, *J* = 7.1 Hz, 3H, *E isom.*), 1.39 (t, *J* = 7.1 Hz, 3H, *Z isom.*), 1.30 (s, 9H, *Z isom.*), 1.29 (s, 9H, *E isom.*).

^{13}C{^{1}H} NMR (75 MHz, $CDCl_3$, 300K) δ 162.1 (C), 160.7 (d, *J* = 248.0 Hz, C), 151.2 (C), 150.5 (C), 139.2 (CH), 138.9 (CH), 136.5 (C), 136.0 (C), 134.7 (C), 133.0 (C), 132.8 (C), 132.6 (CH), 132.1 (d, *J* = 8.6 Hz, CH), 131.5 (d, *J* = 3.4 Hz, C), 129.9 (C), 128.5 (CH), 128.5 (CH), 125.8 (CH), 125.6 (CH), 125.1 (CH), 122.8 (CH), 119.7 (CH), 117.2 (d, *J* = 20.7 Hz, CH), 115.6 (d, *J* = 23.5 Hz, CH), 61.1 (CH_2), 34.6 (C), 31.2 (CH_3), 16.9 (CH_3), 14.3 (CH_3).

^{19}F RMN (282 MHz, $CDCl_3$, 300K): δ -114.78, -114.82.

HRMS (EI): Calcd. for $[C_{25}H_{27}FN_2O_2S]^+$: 438.1777, found: 438.1791.

Ethyl 1-(4-methoxyphenyl)-5-(styrylthio)-1H-imidazole-4-carboxylate (7u)

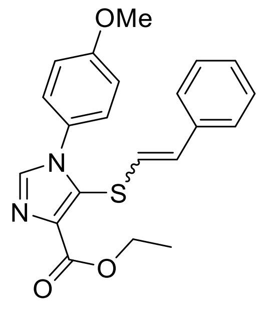

Following **General Procedure A**, isocyanide **4b** (55 μl mg, 0.50 mmol), isothiocyanate **5c** (41 μl, 0.30 mmol), and lithium *tert*-butoxide (48 mg, 0.60 mmol) were dissolved in DMSO. Subsequently, vinyl bromide **1a** (25 μl, 0.20 mmol) was added. After purification

by column chromatography (Hex/EtOAc, 1:2), compound **7u** was obtained as a yellow oil (45 mg, 59% isolated yield, *d.r.* = 2,6:1, *E/Z*).

In the ^{1}H RMN spectrum, both isomers are present, and the integrals of their diagnostic signals were adjusted to one proton to determine the diastereomeric ratio. Consequently, all remaining integrals were normalized accordingly, and their combined values correspond to the total number of hydrogens in the molecule.

Rf = 0.53 (*E isom.*), 0.49 (*Z isom.*). (1:2 Hex/EtOAc) [UV] [KMnO4].

^{1}H RMN (300 MHz, $CDCl_3$, 300K): δ 7.77 (s, 1H, *E isom.*), 7.74 (s, 1H, *Z isom.*), 7.40 – 7.09 (m, 7H), 6.98 – 6.86 (m, 2H), 6.46 (d, *J* = 15.4 Hz, 1H, *E isom.*), 6.42 (d, *J* = 10.7 Hz, 1H, *Z isom.*), 6.29 (d, *J* = 15.3 Hz, 1H, *E isom.*), 6.02 (d, *J* = 10.5 Hz, 1H, *Z isom.*), 4.42 (q, *J* = 7.1 Hz, 2H, *E isom.*), 4.40 (q, *J* = 7.1 Hz, 2H, *Z isom.*), 3.80 (s, 3H), 1.40 (t, *J* = 7.2 Hz, 3H, *E isom.*), 1.39 (t, *J* = 7.3 Hz, 3H, *Z isom.*)

^{13}C{^{1}H} NMR (75 MHz, $CDCl_3$, 300K) δ 162.2 (C), 162.2 (C), 160.3 (C), 160.1 (C), 139.8 (CH), 139.4 (CH), 136.9 (C), 136.2 (C), 136.0 (C), 135.7 (C), 130.7 (CH), 129.3 (C), 128.7 (CH), 128.6 (CH), 128.2 (CH), 128.0 (CH), 127.8 (CH), 127.7 (CH), 127.6 (C), 127.3 (CH), 126.0 (CH), 124.7 (CH), 121.9 (CH), 114.5 (CH), 114.5 (CH), 61.0 (CH_2), 55.6 (CH_3), 14.4 (CH_3), 14.4 (CH_3).

HRMS (EI): Calcd. for $[C_{21}H_{20}N_2O_3S]^+$: 380.1195, found: 380.1210.

Ethyl 1-(4-methoxyphenyl)-5-((4-methylstyryl)thio)-1H-imidazole-4-carboxylate (7v)

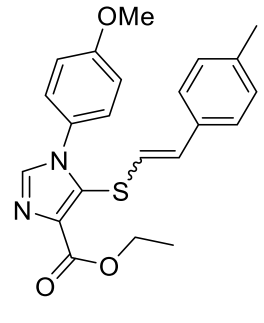

Following **General Procedure A**, isocyanide **4b** (55 μl mg, 0.50 mmol), isothiocyanate **5c** (41 μl, 0.30 mmol), and lithium *tert*-butoxide (48 mg, 0.60 mmol) were dissolved in

DMSO. Subsequently, vinyl bromide **1c** 39 mg, 0.20 mmol) was added. After purification by column chromatography (Hex/EtOAc, 1:2), compound **7v** was obtained as a pale yellow oil (19 mg *E isom.* + 15 mg *Z isom.*, 43% isolated yield, *d.r.* = 1,2:1, *E/Z*).

In this case, both isomers were isolated separately, and their NMR analyses were performed independently.

Rf = 0.33 (*E isom.*), 0.26 (*Z isom.*). (1:2 Hex/EtOAc) [UV] [KMnO4].

- ***E isomer.***

^{1}H RMN (300 MHz, $CDCl_3$, 300K): δ 7.77 (s, 1H), 7.29 – 7.19 (m, 2H), 7.06 (s, 4H), 6.95 (d, *J* = 8.9 Hz, 2H), 6.41 (d, *J* = 15.3 Hz, 1H), 6.29 (d, *J* = 15.3 Hz, 1H), 4.43 (q, *J* = 7.1 Hz, 2H), 3.82 (s, 3H), 2.30 (s, 3H), 1.41 (t, *J* = 7.1 Hz, 3H).

^{13}C{^{1}H} NMR (75 MHz, $CDCl_3$, 300K) δ 162.2 (C), 160.2 (C), 139.7 (CH), 137.7 (C), 136.8 (C), 133.3 (C), 131.2 (CH), 129.3 (CH), 127.8 (CH), 127.7 (C), 127.6 (C), 125.9 (CH), 120.5 (CH), 114.5 (CH), 61.0 (CH_2), 55.6 (CH_3), 21.2 (CH_3), 14.4 (CH_3).

HRMS (EI): Calcd. for $[C_{22}H_{22}N_2O_3S]^+$: 394.1351, found: 394.1361.

- ***Z isomer.***

^{1}H RMN (300 MHz, $CDCl_3$, 300K): δ 7.74 (s, 1H), 7.30 – 7.24 (m, 3H), 7.23 – 7.18 (m, 2H), 7.15 – 7.05 (m, 2H), 6.94 – 6.87 (m, 2H), 6.40 (d, *J* = 10.5 Hz, 1H), 5.97 (d, *J* = 10.5 Hz, 1H), 4.42 (q, *J* = 7.1 Hz, 2H), 3.82 (s, 3H), 2.33 (s, 3H), 1.39 (t, *J* = 7.1 Hz, 3H).

^{13}C{^{1}H} NMR (75 MHz, $CDCl_3$, 300K) δ 162.3 (C), 160.1 (C), 139.4 (CH), 137.2 (C), 136.2 (C), 133.0 (C), 129.5 (C), 128.9 (CH), 128.7 (CH), 128.0 (CH), 127.7 (CH), 123.6 (CH), 114.5 (CH), 61.0 (CH_2), 55.6 (CH_3), 21.3 (CH_3), 14.4 (CH_3).

HRMS (EI): Calcd. for $[C_{22}H_{22}N_2O_3S]^+$: 394.1351, found: 394.1361.

Ethyl 5-(styrylthio)-1-(4-(trifluoromethyl)phenyl)-1H-imidazole-4-carboxylate (7w)

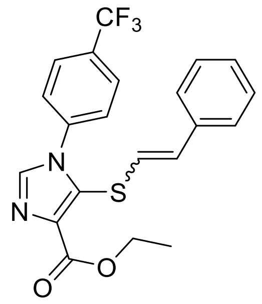

Following **General Procedure A**, isocyanide **4b** (55 μl mg, 0.50 mmol), isothiocyanate **5d** (61 mg, 0.30 mmol), and lithium *tert*-butoxide (48 mg, 0.60 mmol) were dissolved in DMSO. Subsequently, vinyl bromide **1a** (25 μl, 0.20 mmol) was added. After purification by column chromatography (Hex/EtOAc, 1:2), compound **7w** was obtained as a yellow oil (46 mg, 55% isolated yield, *d.r.* = 0,7:1, *E/Z*).

In the ^{1}H RMN spectrum, both isomers are present, and the integrals of their diagnostic signals were adjusted to one proton to determine the diastereomeric ratio. Consequently, all remaining integrals were normalized accordingly, and their combined values correspond to the total number of hydrogens in the molecule.

Rf = 0.67 (*E isom.*), 0.64 (*Z isom.*). (1:2 Hex/EtOAc) [UV] [KMnO4].

^{1}H RMN (300 MHz, $CDCl_3$, 300K): δ 7.85 (s, 1H, *E isom.*), 7.80 (s, 1H, *Z isom.*), 7.76 (d, *J* = 8.2 Hz, 1H), 7.64 (d, *J* = 8.3 Hz, 1H), 7.50 (d, *J* = 8.2 Hz, 1H), 7.43 (d, *J* = 8.3 Hz, 1H), 7.35 – 7.19 (m, 4H), 7.17 – 7.11 (m, 1H), 6.48 (d, *J* = 10.4 Hz, 1H, *Z isom.*), 6.46 (d, *J* = 15.3 Hz, 1H, *E isom.*), 6.32 (d, *J* = 15.4 Hz, 1H, *E isom.*), 6.02 (d, *J* = 10.4 Hz, 1H, *Z isom.*), 4.44 (q, *J* = 7.2 Hz, 2H, *E isom.*), 4.43 (q, *J* = 7.1 Hz, 2H, *Z isom.*), 1.41 (t, *J* = 7.2 Hz, 3H, *E isom.*), 1.40 (t, *J* = 7.1 Hz, 3H, *Z isom.*)

$^{13}C\{^{1}H\}$ NMR (75 MHz, $CDCl_3$, 300K) δ 162.0 (C), 161.9 (C), 139.3 (CH), 139.0 (CH), 137.9 (C), 137.8 (C), 137.0 (C), 135.7 (C), 135.4 (C), 131.8 (CH), 131.2 (C), 129.6 (CH), 128.9 (CH), 128.8 (CH), 128.7 (C), 128.3 (CH), 128.0 (CH), 127.7 (CH), 127.0 (CH), 126.8 (CH), 126.7 (CH), 126.6 (CH), 126.0 (CH), 123.6 (CH), 121.2 (CH), 61.3 (CH_2), 14.4 (CH_3).

^{19}F RMN (282 MHz, $CDCl_3$, 300K): δ -62.74, -62.75.

HRMS (EI): Calcd. for $[C_{21}H_{17}F_3N_2O_2S]^+$: 418.0963, found: 418.0970.

Ethyl 5-((2-(naphthalen-2-yl)vinyl)thio)-1-(4-(trifluoromethyl)phenyl)-1H-imidazole-4-carboxylate (7x)

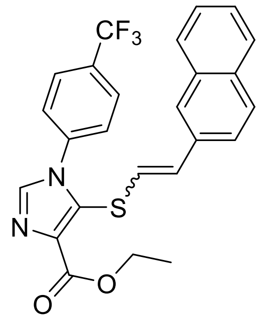

Following **General Procedure A**, isocyanide **4b** (55 µl mg, 0.50 mmol), isothiocyanate **5d** (61 mg, 0.30 mmol), and lithium *tert*-butoxide (48 mg, 0.60 mmol) were dissolved in DMSO. Subsequently, vinyl bromide **1n** (47 mg, 0.20 mmol) was added. After purification by column chromatography (Hex/EtOAc, 1:2), compound **7x** was obtained as a yellow oil (37 mg, 40% isolated yield, *d.r.* = 1,1:1 *E/Z*).

In the ^{1}H RMN spectrum, both isomers are present, and the integrals of their diagnostic signals were adjusted to one proton to determine the diastereomeric ratio. Consequently, all remaining integrals were normalized accordingly, and their combined values correspond to the total number of hydrogens in the molecule.

Rf = 0.75 (*E isom.*), 0.72 (*Z isom.*). (1:2 Hex/EtOAc) [UV] [KMnO4].

^{1}H RMN (300 MHz, $CDCl_3$, 300K): δ 7.87 (s, 1H, *Z isom.*), 7.85 – 7.68 (m, 4H + 1H *E isom.*), 7.62 (d, *J* = 8.3 Hz, 1H), 7.55 – 7.39 (m, 5H + 1H *E isom.*), 7.34 (dd, *J* = 8.6, 1.8 Hz, 1H, *Z isom.*), 6.62 (d, *J* = 10.4 Hz, 1H, *Z isom.*), 6.59 (d, *J* = 15.4 Hz, 1H, *E isom.*), 6.47 (d, *J* = 15.4 Hz, 1H, *E isom.*), 6.10 (d, *J* = 10.4 Hz, 1H, *Z isom.*), 4.45 (q, *J* = 7.1 Hz, 2H), 1.43 (t, *J* = 7.1 Hz, 3H, *E isom.*), 1.41 (t, *J* = 7.1 Hz, 3H, *Z isom.*).

$^{13}C\{^{1}H\}$ NMR (75 MHz, $CDCl_3$, 300K) δ 162.0 (C), 161.9 (C), 139.3 (CH), 139.0 (CH), 137.9 (C), 137.8 (C), 137.7 (C), 137.0 (C), 133.4 (C), 133.1 (C), 133.0 (C), 132.9 (C), 132.6 (C), 131.8 (CH), 129.6 (CH), 128.9 (C), 128.4 (CH), 128.2 (CH), 128.1 (CH), 128.0 (CH), 127.9 (CH), 127.7 (CH), 127.6 (CH), 127.0 (CH), 126.7 (CH), 126.6 (CH), 126.5 (CH), 126.5 (CH), 126.4 (CH), 126.2 (CH), 126.1 (CH), 124.0 (CH), 122.9 (CH), 121.5 (CH), 61.3 (CH_2), 14.4 (CH_3).

^{19}F RMN (282 MHz, $CDCl_3$, 300K): δ -62.72, -62.75.

HRMS (EI): Calcd. for $[C_{25}H_{19}F_3N_2O_2S]^+$: 468.1119, found: 468.1148.

Ethyl 1-(4-fluorophenyl)-5-(styrylthio)-1H-imidazole-4-carboxylate (7y)

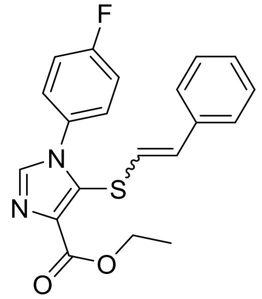

Following **General Procedure A**, isocyanide **4b** (55 μl mg, 0.50 mmol), isothiocyanate **5e** (37 μl, 0.30 mmol), and lithium *tert*-butoxide (48 mg, 0.60 mmol) were dissolved in DMSO. Subsequently, vinyl bromide **1a** (25 μl, 0.20 mmol) was added. After purification by column chromatography (Hex/EtOAc, 1:1), compound **7y** was obtained as a yellow oil (38 mg *E isom.* + 15 mg *Z isom.,* 72% isolated yield, *d.r.* = 2,4:1, *E/Z*).

In this case, both isomers were isolated separately, and their NMR analyses were performed independently.

Rf = 0.36 (*E isom.*), 0.27 (*Z isom.*). (1:1 Hex/EtOAc) [UV] [KMnO4].

- ***E isomer.***

^{1}H RMN (300 MHz, $CDCl_3$, 300K): δ 7.80 (s, 1H), 7.36 – 7.11 (m, 9H), 6.46 (d, *J* = 15.3 Hz, 1H), 6.30 (d, *J* = 15.4 Hz, 1H), 4.43 (q, *J* = 7.1 Hz, 2H), 1.41 (t, *J* = 7.1 Hz, 3H).

$^{13}C\{^1H\}$ NMR (75 MHz, $CDCl_3$, 300K) δ 162.9 (d, *J* = 250.6 Hz, C), 162.0 (C), 139.6 (CH), 137.4 (C), 135.8 (C), 131.1 (CH), 130.9 (d, *J* = 3.2 Hz, C), 128.7 (CH), 128.5 (d, *J* = 8.9 Hz, CH), 127.8 (CH), 127.1 (C), 126.0 (CH), 121.6 (CH), 116.5 (d, *J* = 23.0 Hz, CH), 61.1 (CH_2), 14.4 (CH_3).

^{19}F RMN (282 MHz, $CDCl_3$, 300K): δ -110.61.

HRMS (EI): Calcd. for $[C_{20}H_{17}FN_2O_2S]^+$: 368.0995, found: 368.1007.

- ***Z isomer.***

^{1}H RMN (300 MHz, $CDCl_3$, 300K): δ 7.76 (s, 1H), 7.38 – 7.22 (m, 7H), 7.09 (t, *J* = 8.5 Hz, 2H), 6.46 (d, *J* = 10.5 Hz, 1H), 6.02 (d, *J* = 10.5 Hz, 1H), 4.43 (q, *J* = 7.1 Hz, 2H), 1.39 (t, *J* = 7.1 Hz, 3H).

$^{13}C\{^1H\}$ NMR (75 MHz, $CDCl_3$, 300K) δ 162.7 (d, *J* = 250.5 Hz, C), 162.1 (C), 139.2 (CH), 136.6 (C), 135.5 (C), 130.9 (d, *J* = 250.5 Hz, C), 129.2 (C), 128.7 (CH), 128.4 (CH), 128.3 (CH), 127.5 (CH), 124.2 (CH), 116.5 (d, *J* = 23.1 Hz), 61.1 (CH_2), 14.4 (CH_3).

^{19}F RMN (282 MHz, $CDCl_3$, 300K): δ -110.90.

HRMS (EI): Calcd. for $[C_{20}H_{17}FN_2O_2S]^+$: 368.0995, found: 368.1007.

Ethyl 1-(4-fluorophenyl)-5-((2-(naphthalen-2-yl)vinyl)thio)-1H-imidazole-4-carboxylate (7z)

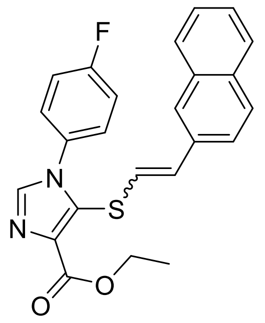

Following **General Procedure A**, isocyanide **4b** (55 μl mg, 0.50 mmol), isothiocyanate **5e** (37 μl, 0.30 mmol), and lithium *tert*-butoxide (48 mg, 0.60 mmol) were dissolved in DMSO. Subsequently, vinyl bromide **1n** (47 mg, 0.20 mmol) was added. After purification by column chromatography (Hex/EtOAc, 1:1), compound **7z** was obtained as a yellow oil (19 mg *E isom.* + 15 mg *Z isom.,* 41% isolated yield, *d.r.* = 1,3:1, *E/Z*).

In this case, both isomers were isolated separately, and their NMR analyses were performed independently.

Rf = 0.42 (*E isom.*), 0.36 (*Z isom.*). (1:1 Hex/EtOAc) [UV] [KMnO4].

- ***E isomer.***

^{1}H RMN (300 MHz, $CDCl_3$, 300K): δ 7.83 (s, 1H), 7.81 – 7.68 (m, 3H), 7.53 (d, *J* = 1.7 Hz, 1H), 7.49 – 7.39 (m, 2H), 7.40 – 7.29 (m, 3H), 7.23 – 7.11 (m, 2H), 6.59 (d, *J* = 15.3 Hz, 1H), 6.46 (d, *J* = 15.4 Hz, 1H), 4.45 (q, *J* = 7.1 Hz, 2H), 1.42 (t, *J* = 7.1 Hz, 3H).

^{13}C{^{1}H} NMR (75 MHz, $CDCl_3$, 300K) δ 162.9 (d, *J* = 250.7 Hz, C), 162.1 (C), 139.6 (CH), 137.5 (C), 133.5 (C), 133.3 (C), 132.9 (C), 131.1 (CH), 130.9 (d, *J* = 3.3 Hz, C), 128.5 (CH), 128.4 (CH), 128.0 (CH), 127.7 (CH), 127.1 (C), 126.5 (CH), 126.1 (d, *J* = 13.5 Hz, CH), 122.9 (CH), 121.9 (CH), 116.5 (d, *J* = 23.1 Hz, CH), 61.2 (CH_2), 14.4 (CH_3).

^{19}F RMN (282 MHz, $CDCl_3$, 300K): δ - 110.54.

HRMS (EI): Calcd. for $[C_{24}H_{19}FN_2O_2S]^+$: 418.1151, found: 418.1162.

- ***Z isomer.***

^{1}H RMN (300 MHz, $CDCl_3$, 300K): δ 7.84 – 7.73 (m, 5H), 7.52 – 7.43 (m, 3H), 7.32 – 7.24 (m, 2H), 7.12 – 7.01 (m, 2H), 6.60 (d, *J* = 10.4 Hz, 1H), 6.10 (d, *J* = 10.4 Hz, 1H), 4.44 (q, *J* = 7.1 Hz, 2H), 1.41 (t, *J* = 7.1 Hz, 3H).

^{13}C{^{1}H} NMR (75 MHz, $CDCl_3$, 300K) δ 162.1 (C), 162.7 (d, *J* = 250.2 Hz, C), 139.3 (CH), 136.6 (C), 133.1 (d, *J* = 4.8 Hz, C), 132.5 (C), 130.9 (d, *J* = 4.8 Hz, C), 129.2 (C), 128.8 (CH), 128.4 (CH), 128.2 (d, *J* = 8.1 Hz, CH), 128.0 (CH), 127.9 (CH), 127.6 (CH), 126.5 (CH), 126.3 (CH), 126.3 (CH), 124.6 (CH), 116.6 (d, *J* = 23.2 Hz, CH), 61.2 (CH_2), 14.4 (CH_3).

^{19}F RMN (282 MHz, $CDCl_3$, 300K): δ - 110.83.

HRMS (EI): Calcd. for $[C_{24}H_{19}FN_2O_2S]^+$: 418.1151, found: 418.1162.

Ethyl 5-((4-((((3aS,5S,6R,6aS)-5-((R)-2,2-dimethyl-1,3-dioxolan-4-yl)-2,2-dimethyltetrahydrofuro[2,3-d][1,3]dioxol-6-yl)oxy)carbonyl)styryl)thio)-1-phenyl-1H-imidazole-4-carboxylate (7aa)

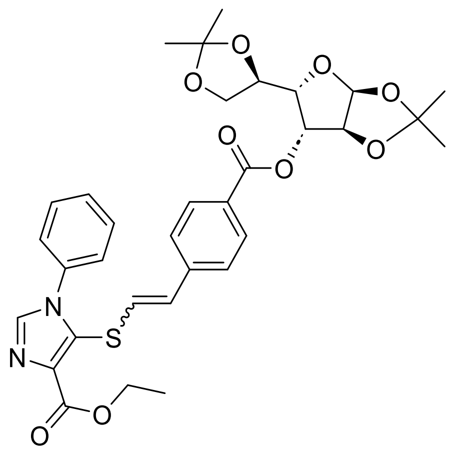

Following **General Procedure A**, isocyanide **4b** (55 μl mg, 0.50 mmol), isothiocyanate **5a** (36 μl, 0.30 mmol), and lithium *tert*-butoxide (48 mg, 0.60 mmol) were dissolved in DMSO. Subsequently, vinyl bromide **1q** (94 mg, 0.20 mmol) was added. After purification by column chromatography (Hex/EtOAc, 1:3), compound **7a'** was obtained as a yellow oil (51 mg, 40% isolated yield, *d.r.* = 2,4:1, *E/Z*).

In the ^{1}H RMN spectrum, both isomers are present, and the integrals of their diagnostic signals were adjusted to one proton to determine the diastereomeric ratio. Consequently, all remaining integrals were normalized accordingly, and their combined values correspond to the total number of hydrogens in the molecule. In addition, in this case, both sugar epimers are present, resulting in a total of four diastereoisomers instead of two.

Rf = 0.8 (*E isom.*), 0.74 (*Z isom.*). (1:3 Hex/EtOAc) [UV] [KMnO4].

^{1}H RMN (300 MHz, $CDCl_3$, 300K): δ 8.01 – 7.77 (m, 3H), 7.53 – 7.38 (m, 4H), 7.37 – 7.23 (m, 2H), 7.24 – 7.13 (m, 1H), 6.64 (d, *J* = 15.4 Hz, 1H, *E isom.*), 6.60 (d, *J* = 15.4 Hz, 1H, *E isom.*), 6.44 (d, *J* = 10.7 Hz, 1H, *Z isom.*), 6.28 (d, *J* = 15.0 Hz, 1H, *E isom.*), 6.20 (d, *J* = 10.6 Hz, 1H, *Z isom.*), 6.17 (d, *J* = 10.6 Hz, 1H, *Z isom.*), 5.97 – 5.89 (m, 1H), 5.54 – 5.42 (m, 1H), 4.60 (t, *J* = 4.0 Hz, 1H), 4.48 – 4.26 (m, 4H), 4.07 (t, *J* = 4.0 Hz, 2H), 1.53 (s, 3H), 1.43 – 1.23 (m, 12H).

^{13}C{^{1}H} NMR (75 MHz, $CDCl_3$, 300K) δ 166.3 (C), 164.8 (C), 162.0 (C), 140.8 (C), 140.1 (C), 139.8 (CH), 139.4 (CH), 137.5 (C), 136.6 (C), 134.7 (C), 130.1 (CH), 129.9 (CH), 129.8 (CH), 129.7 (CH), 129.6 (CH), 129.5 (CH), 129.2 (C), 128.7 (CH), 128.5 (CH), 128.3 (C), 128.1 (CH), 127.8 (C), 127.6 (CH), 126.9 (CH), 126.5 (C), 126.4 (CH), 126.1 (CH), 126.0 (CH), 125.8 (CH), 125.6 (CH), 125.3 (CH), 112.4 (C), 109.4 (C), 105.1 (CH),

83.3 (CH), 79.9 (CH), 77.2 (CH), 72.6 (CH), 67.2 (CH_2), 61.2 (CH_3), 26.8 (CH_3), 26.7 (CH_3), 26.2 (CH_3), 25.2 (CH_3), 14.4 (CH_3).

HRMS (EI): Calcd. for $[C_{33}H_{36}N_2O_9S]^+$: 636.2142, found: 636.2147.

Ethyl 5-((4-((((1S,5R)-6,6-dimethylbicyclo[3.1.1]hept-2-en-2-yl)methoxy)carbonyl)styryl)thio)-1-phenyl-1H-imidazole-4-carboxylate (7ab)

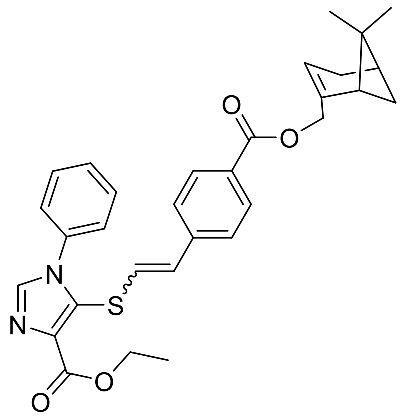

Following **General Procedure A**, isocyanide **4b** (55 µl mg, 0.50 mmol), isothiocyanate **5a** (36 µl, 0.30 mmol), and lithium *tert*-butoxide (48 mg, 0.60 mmol) were dissolved in DMSO. Subsequently, vinyl bromide **1r** (69 mg, 0.20 mmol) was added. After purification by column chromatography (Hex/EtOAc, 1:1), compound **7b'** was obtained as a yellow oil (27 mg *E isom.* + 39 mg *Z isom.,* 62% isolated yield, *d.r.* = 0,7:1, *E/Z*).

In this case, both isomers were isolated separately, and their NMR analyses were performed independently.

Rf = 0.49 (*E isom.*), 0.37 (*Z isom.*). (1:2 Hex/EtOAc) [UV] [KMnO4].

- ***E isomer.***

^{1}H RMN (300 MHz, $CDCl_3$, 300K): δ 7.93 – 7.82 (m, 3H), 7.53 – 7.41 (m, 3H), 7.36 – 7.29 (m, 2H), 7.19 (d, *J* = 8.3 Hz, 2H), 6.61 (d, *J* = 15.4 Hz, 1H), 6.29 (d, *J* = 15.4 Hz, 1H), 5.63 (td, *J* = 3.0, 1.5 Hz, 1H), 4.66 (q, *J* = 1.5 Hz, 2H), 4.42 (q, *J* = 7.1 Hz, 2H), 2.47 – 2.36 (m, 1H), 2.29 (dd, *J* = 7.5, 4.6 Hz, 2H), 2.18 (td, *J* = 5.6, 1.5 Hz, 1H), 2.16 – 2.07 (m, 1H), 1.40 (t, *J* = 7.1 Hz, 3H), 1.28 (s, 3H), 1.21 (d, *J* = 8.7 Hz, 1H), 0.84 (s, 3H).

$^{13}C\{^1H\}$ NMR (75 MHz, $CDCl_3$, 300K) δ 166.1 (C), 162.0 (C), 142.9 (C), 140.2 (C), 139.8 (CH), 137.5 (C), 134.8 (C), 130.0 (CH), 129.7 (CH), 129.6 (C), 129.5 (CH), 129.2 (C), 128.6 (CH), 126.4 (CH), 126.2 (C), 125.7 (CH), 125.3 (CH), 121.7 (CH), 67.5 (CH_2), 61.2 (CH_2), 43.6 (CH), 40.7 (CH), 38.1 (C), 31.5 (CH_2), 31.3 (CH_2), 26.2 (CH_3), 21.2 (CH_3), 14.4 (CH_3).

HRMS (EI): Calcd. for $[C_{31}H_{32}N_2O_4S]^+$: 528.2083, found: 528.2090.

- ***Z isomer.***

^1H RMN (300 MHz, $CDCl_3$, 300K): δ 7.95 (d, *J* = 8.2 Hz, 2H), 7.80 (s, 1H), 7.49 – 7.38 (m, 5H), 7.34 – 7.24 (m, 2H), 6.44 (d, *J* = 10.7 Hz, 1H), 6.17 (d, *J* = 10.6 Hz, 1H), 5.69 – 5.58 (m, 1H), 4.67 (q, *J* = 1.7 Hz, 2H), 4.42 (q, *J* = 7.1 Hz, 2H), 2.53 – 2.36 (m, 1H), 2.34 – 2.25 (m, 2H), 2.22 – 2.16 (m, 1H), 2.13 – 2.06 (m, 1H), 1.38 (t, *J* = 7.1 Hz, 3H), 1.29 (s, 3H), 1.22 (d, *J* = 8.8 Hz, 1H), 0.86 (s, 3H).

$^{13}C\{^1H\}$ NMR (75 MHz, $CDCl_3$, 300K) δ 166.1 (C), 162.1 (C), 143.0 (C), 140.1 (C), 139.4 (CH), 136.6 (C), 134.7 (C), 129.6 (CH), 129.5 (CH), 128.9 (C), 128.5 (C), 128.5 (CH), 127.6 (CH), 126.9 (CH), 126.4 (CH), 121.8 (CH), 67.6 (CH_2), 61.1 (CH_2), 43.6 (CH), 40.7 (CH), 38.1 (C), 31.5 (CH_2), 31.3 (CH_2), 26.2 (CH_3), 21.2 (CH_3), 14.4 (CH_3).

HRMS (EI): Calcd. for $[C_{31}H_{32}N_2O_4S]^+$: 528.2083, found: 528.2090.

Ethyl 5-((4-((((1S,2R,5R)-2-isopropyl-5-methylcyclohexyl)oxy)carbonyl)styryl)thio)-1-phenyl-1H-imidazole-4-carboxylate (7ac)

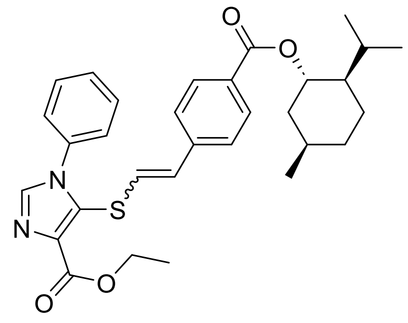

Following **General Procedure A**, isocyanide **4b** (55 µl mg, 0.50 mmol), isothiocyanate **5a** (36 µl, 0.30 mmol), and lithium *tert*-butoxide (48 mg, 0.60 mmol) were dissolved in

DMSO. Subsequently, vinyl bromide **1s** (73 mg, 0.20 mmol) was added. After purification by column chromatography (Hex/EtOAc, 1:1), compound **7c'** was obtained as a yellow oil (27 mg *E isom.* + 35 mg *Z isom.,* 58% isolated yield, *d.r.* = 0,8:1, *E/Z*).

In this case, both isomers were isolated separately, and their NMR analyses were performed independently.

Rf = 0.50 (*E isom.*), 0.36 (*Z isom.*). (1:2 Hex/EtOAc) [UV] [KMnO4].

- ***E isomer.***

^{1}H RMN (300 MHz, $CDCl_3$, 300K): δ 7.92 (d, *J* = 8.2 Hz, 2H), 7.85 (s, 1H), 7.48 (dd, *J* = 5.0, 1.9 Hz, 3H), 7.33 (dd, *J* = 6.9, 2.8 Hz, 2H), 7.18 (d, *J* = 8.2 Hz, 2H), 6.60 (d, *J* = 15.4 Hz, 1H), 6.29 (d, *J* = 15.4 Hz, 1H), 5.37 – 5.17 (m, 1H), 4.43 (q, *J* = 7.1 Hz, 2H), 1.99 – 1.88 (m, 1H), 1.85 – 1.62 (m, 4H), 1.65 – 1.42 (m, 3H), 1.41 (t, *J* = 7.1 Hz, 3H), 1.32 – 1.16 (m, 1H), 0.96 (t, *J* = 6.7 Hz, 6H), 0.87 (d, *J* = 6.7 Hz, 3H).

^{13}C{^{1}H} NMR (75 MHz, $CDCl_3$, 300K) δ 165.6 (C) , 162.0 (C), 140.0 (C), 139.8 (CH), 137.5 (C), 134.8 (C), 129.9 (CH), 129.8 (C), 129.7 (CH), 129.6 (C), 129.5 (CH), 128.8 (CH), 126.4 (CH), 126.2 (C), 125.6 (CH), 125.1 (CH), 72.5 (CH), 61.2 (CH_2), 45.7 (CH), 35.7 (CH_2), 29.9 (CH_2), 27.7 (CH_3), 26.4 (CH_3), 21.3 (CH_2), 21.0 (CH_3), 20.8 (CH_3), 19.4 (CH_3), 14.4 (CH_3).

HRMS (EI): Calcd. for $[C_{31}H_{36}N_2O_4S]^+$: 532.2396, found: 532.2404.

- ***Z isomer.***

^{1}H RMN (300 MHz, $CDCl_3$, 300K): δ 8.02 – 7.93 (m, 2H), 7.80 (s, 1H), 7.48 – 7.36 (m, 5H), 7.34 – 7.23 (m, 2H), 6.45 (d, *J* = 10.6 Hz, 1H), 6.17 (d, *J* = 10.6 Hz, 1H), 5.33 – 5.22 (m, 1H), 4.42 (q, *J* = 7.1 Hz, 1H), 2.00 – 1.89 (m, 1H), 1.87 – 1.64 (m, 4H), 1.60 – 1.45 (m, 3H), 1.38 (t, *J* = 7.1 Hz, 3H), 1.30 – 1.18 (m, 1H), 0.96 (t, *J* = 7.1 Hz, 6H), 0.88 (d, *J* = 6.7 Hz, 3H)

^{13}C{^{1}H} NMR (75 MHz, $CDCl_3$, 300K) δ 165.6 (C), 162.1 (C), 139.9 (C), 139.4 (CH), 136.6 (C), 134.7 (C), 129.9 (C), 129.6 (CH), 129.5 (CH), 129.5 (CH), 128.8 (C), 128.5 (CH), 127.5 (CH), 127.0 (CH), 126.4 (CH), 125.6 (C), 72.5 (CH), 61.1 (CH_2), 45.7 (CH), 35.7 (CH_2), 29.9 (CH_2), 27.7 (CH), 26.4 (CH), 21.3 (CH_2), 20.9 (CH_3), 20.8 (CH_3), 19.4 (CH_3), 14.4 (CH_3).

HRMS (EI): Calcd. for $[C_{31}H_{36}N_2O_4S]^+$: 532.2396, found: 532.2404.

Ethyl 5-((4-(4-chlorophenyl)buta-1,3-dien-1-yl)thio)-1-phenyl-1H-imidazole-4-carboxylate (8a)

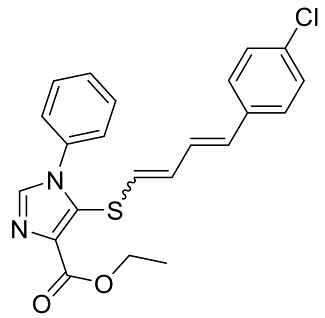

Following **General Procedure A**, isocyanide **4b** (55 µl mg, 0.50 mmol), isothiocyanate **5a** (36 µl, 0.30 mmol), and lithium *tert*-butoxide (48 mg, 0.60 mmol) were dissolved in DMSO. Subsequently, dienyl bromide **2a** (49 mg, 0.20 mmol) was added. After purification by column chromatography (Hex/EtOAc, 1:1), compound **8a** was obtained as a yellow oil (35 mg, 43% isolated yield, *d.r.* = 1,4:1, *E/Z*).

In the ^{1}H RMN spectrum, both isomers are present, and the integrals of their diagnostic signals were adjusted to one proton to determine the diastereomeric ratio. Consequently, all remaining integrals were normalized accordingly, and their combined values correspond to the total number of hydrogens in the molecule.

Rf = 0.45 (*E isom.*), 0.41 (*Z isom.*). (1:1 Hex/EtOAc) [UV] [KMnO4].

^{1}H RMN (300 MHz, $CDCl_3$, 300K): δ 7.83 (s, 1H, *E isom.*), 7.77 (s, 1H, *Z isom.*), 7.55 – 7.41 (m, 3H), 7.37 – 7.27 (m, 2H), 7.24 (d, *J* = 9.0 Hz, 4H), 6.91 (dd, *J* = 15.5, 10.6 Hz, 1H, *Z isom.*), 6.58 (ddd, *J* = 15.6, 8.2, 1.8 Hz, 1H, *E isom.*), 6.45 (d, *J* = 15.6 Hz, 1H, *E isom.*), 6.29 (d, *J* = 15.8 Hz, 1H), 6.10 (d, *J* = 8.5 Hz, 1H), 5.91 (d, *J* = 9.1 Hz, 1H, *Z isom.*), 4.43 (q, *J* = 7.1 Hz, 2H), 1.41 (t, *J* = 7.1 Hz, 3H).

^{13}C{^{1}H} NMR (75 MHz, $CDCl_3$, 300K) d 162.2 (C), 162.0 (C), 139.6 (CH), 139.2 (CH), 137.4 (C), 136.5 (C), 135.4 (C), 135.3 (C), 134.9 (C), 134.8 (C), 133.6 (C), 133.2 (C), 133.1 (CH), 130.6 (CH), 130.5 (CH), 130.4 (CH), 129.6 (CH), 129.5 (CH), 128.8 (CH), 128.3 (C), 127.8 (CH), 127.4 (CH), 127.4 (CH), 126.6 (CH), 126.5 (C), 126.4 (CH), 125.7 (CH), 124.3 (CH), 123.8 (CH), 61.1 (CH_2), 61.0 (CH_2), 14.4 (CH_3).

HRMS (EI): Calcd. for $[C_{22}H_{19}ClN_2O_2S]^+$: 410.0856, found: 410.0875.

Ethyl 1-phenyl-5-(phenylthio)-1H-imidazole-4-carboxylate (9a)

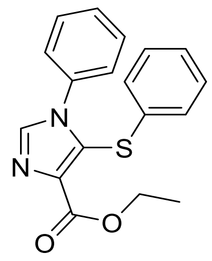

Following **General Procedure A**, isocyanide **4b** (55 μl mg, 0.50 mmol), isothiocyanate **5a** (36 μl, 0.30 mmol), and lithium *tert*-butoxide (48 mg, 0.60 mmol) were dissolved in DMSO. Subsequently, aryl iodide **3a** (41 mg, 0.20 mmol) was added. After purification by column chromatography (Hex/EtOAc, 1:2), compound **9a** was obtained as a yellow oil (32 mg, 50% isolated yield).

Rf = 0.50 (1:2 Hex/EtOAc) [UV] [KMnO4].

^{1}H RMN (300 MHz, $CDCl_3$, 300K): δ 7.79 (s, 1H), 7.47 – 7.31 (m, 3H), 7.22 – 7.04 (m, 5H), 7.02 – 6.85 (m, 2H), 4.39 (q, *J* = 7.1 Hz, 2H), 1.35 (t, *J* = 7.1 Hz, 3H).

^{13}C{^{1}H} NMR (75 MHz, $CDCl_3$, 300K) δ 162.1 (C), 139.7 (CH), 137.9 (C), 134.8 (C), 129.3 (CH), 129.2 (CH), 129.0 (CH), 128.5 (CH), 127.7 (C), 126.7 (CH), 126.6 (CH), 61.0 (CH_2), 14.3 (CH_3).

HRMS (EI): Calcd. for $[C_{18}H_{16}N_2O_2S]^+$: 324.0932, found: 324.0937.

Ethyl 5-((4-(tert-butyl)phenyl)thio)-1-phenyl-1H-imidazole-4-carboxylate (9b)

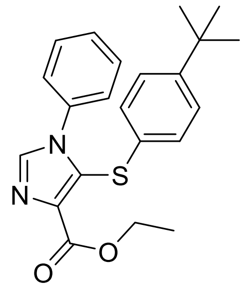

Following **General Procedure A**, isocyanide **4b** (55 μl mg, 0.50 mmol), isothiocyanate **5a** (36 μl, 0.30 mmol), and lithium *tert*-butoxide (48 mg, 0.60 mmol) were dissolved in

DMSO. Subsequently, aryl iodide **3b** (52 mg, 0.20 mmol) was added. After purification by column chromatography (Hex/EtOAc, 1:1), compound **9b** was obtained as a yellow oil (31 mg, 41% isolated yield).

Rf = 0.33 (1:1 Hex/EtOAc) [UV] [KMnO4].

^{1}H RMN (300 MHz, $CDCl_3$, 300K): δ 7.76 (s, 1H), 7.52 – 7.32 (m, 3H), 7.21 – 7.05 (m, 4H), 6.96 – 6.74 (m, 2H), 4.40 (q, *J* = 7.1 Hz, 2H), 1.36 (t, *J* = 7.1 Hz, 3H), 1.23 (s, 9H).

^{13}C{^{1}H} NMR (75 MHz, $CDCl_3$, 300K) δ 162.2 (C), 150.1 (C), 139.5 (CH), 137.4 (C), 135.0 (C), 131.0 (C), 129.2 (CH), 129.2 (CH), 129.0 (CH), 128.6 (C), 126.7 (CH), 126.0 (CH), 60.9 (CH_2), 34.4 (C), 31.2 (CH_3), 14.3 (CH_3).

HRMS (EI): Calcd. for $[C_{22}H_{24}N_2O_2S]^+$: 380.1558, found: 380.1567.

Ethyl 1-phenyl-5-((4-(trifluoromethyl)phenyl)thio)-1H-imidazole-4-carboxylate (9c)

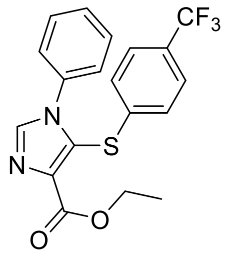

Following **General Procedure A**, isocyanide **4b** (55 μl mg, 0.50 mmol), isothiocyanate **5a** (36 μl, 0.30 mmol), and lithium *tert*-butoxide (48 mg, 0.60 mmol) were dissolved in DMSO. Subsequently, aryl iodide **3c** (54 mg, 0.20 mmol) was added. After purification by column chromatography (Hex/EtOAc, 1:1), compound **9c** was obtained as a yellow solid (54 mg, 69% isolated yield).

Rf = 0.23 (1:1 Hex/EtOAc) [UV] [KMnO4].

^{1}H RMN (300 MHz, $CDCl_3$, 300K): δ 7.86 (s, 1H), 7.51 – 7.31 (m, 5H), 7.18 – 7.10 (m, 2H), 7.05 (d, *J* = 8.2 Hz, 2H), 4.37 (q, *J* = 7.1 Hz, 2H), 1.33 (t, *J* = 7.1 Hz, 3H).

^{13}C{^{1}H} NMR (75 MHz, $CDCl_3$, 300K) δ 161.9 (C), 140.2 (CH), 138.8 (C), 134.6 (C), 129.6 (CH), 129.4 (CH), 128.5 (q, *J* = 32.9 Hz, C), 127.3 (CH), 126.4 (CH), 125.9 (q, *J* = 3.8 Hz, CH), 125.7 (C), 125.5 (C), 122.1 (C), 118.5 (C), 61.2 (CH_2), 14.2 (CH_3).

^{19}**F RMN** (282 MHz, $CDCl_3$, 300K): δ - 62.58

HRMS (EI): Calcd. for $[C_{19}H_{15}F_3N_2O_2S]^+$: 392.0835, found: 392.0806.

Ethyl 5-((4-ethylphenyl)thio)-1-phenyl-1H-imidazole-4-carboxylate (9d)

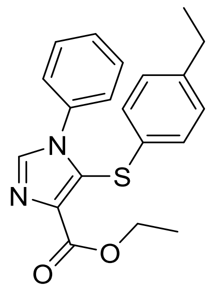

Following **General Procedure A**, isocyanide **4b** (55 μl mg, 0.50 mmol), isothiocyanate **5a** (36 μl, 0.30 mmol), and lithium *tert*-butoxide (48 mg, 0.60 mmol) were dissolved in DMSO. Subsequently, aryl iodide **3d** (46 mg, 0.20 mmol) was added. After purification by column chromatography (Hex/EtOAc, 1:1), compound **9d** was obtained as a pale yellow solid (28 mg, 40% isolated yield).

Rf = 0.35 (1:1 Hex/EtOAc) [UV] [KMnO4].

^{1}**H RMN** (300 MHz, $CDCl_3$, 300K): δ 7.76 (s, 1H), 7.48 – 7.33 (m, 3H), 7.23 – 7.07 (m, 2H), 6.98 – 6.86 (m, 4H), 4.40 (q, *J* = 7.1 Hz, 2H), 2.53 (q, *J* = 7.6 Hz, 2H), 1.37 (t, *J* = 7.1 Hz, 3H), 1.15 (t, *J* = 7.6 Hz, 3H).

^{13}**C{^{1}H} NMR** (75 MHz, $CDCl_3$, 300K) δ 162.2 (C), 143.3 (C), 139.5 (CH), 137.5 (C), 135.0 (C), 131.2 (C), 129.2 (CH), 129.2 (CH), 128.5 (CH), 126.7 (CH), 60.9 (CH_2), 28.4 (CH_2), 15.5 (CH_3), 14.3 (CH_3).

HRMS (EI): Calcd. for $[C_{20}H_{20}N_2O_2S]^+$: 352.1245, found: 352.1269.

Ethyl 1-phenyl-5-(pyridin-2-ylthio)-1H-imidazole-4-carboxylate (9e)

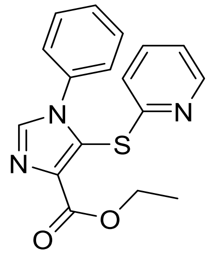

Following **General Procedure A**, isocyanide **4b** (55 μl mg, 0.50 mmol), isothiocyanate **5a** (36 μl, 0.30 mmol), and lithium *tert*-butoxide (48 mg, 0.60 mmol) were dissolved in DMSO. Subsequently, aryl iodide **3e** (41 mg, 0.20 mmol) was added. After purification by column chromatography (Hex/EtOAc, 1:2), compound **9e** was obtained as a pale yellow oil (31 mg, 48% isolated yield).

Rf = 0.10 (1:2 Hex/EtOAc) [UV] [KMnO4].

^{1}H RMN (300 MHz, $CDCl_3$, 300K): δ 8.29 (d, *J* = 3.9 Hz, 1H), 7.90 (s, 1H), 7.49 – 7.35 (m, 4H), 7.33 – 7.22 (m, 2H), 7.02 – 6.86 (m, 2H), 4.33 (q, *J* = 7.1 Hz, 2H), 1.28 (t, *J* = 7.1 Hz, 3H).

^{13}C{^{1}H} NMR (75 MHz, $CDCl_3$, 300K) δ 162.0 (C), 158.1 (C), 149.7 (CH), 140.0 (CH), 138.5 (C), 136.7 (CH), 134.9 (C), 129.4 (CH), 129.3 (CH), 126.4 (CH), 121.1 (CH), 120.5 (CH), 60.9 (CH_2), 14.2 (CH_3).

HRMS (EI): Calcd. for $[C_{17}H_{15}N_3O_2S]^+$: 325.0885, found: 325.0895.

Ethyl 5-((3-formylphenyl)thio)-1-phenyl-1H-imidazole-4-carboxylate (9f)

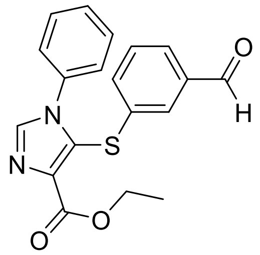

Following **General Procedure A**, isocyanide **4b** (55 μl mg, 0.50 mmol), isothiocyanate **5a** (36 μl, 0.30 mmol), and lithium *tert*-butoxide (48 mg, 0.60 mmol) were dissolved in DMSO. Subsequently, aryl iodide **3f** (46 mg, 0.20 mmol) was added. After purification by column chromatography (EtOAc), compound **9f** was obtained as a yellow oil (32 mg, 45% isolated yield).

Rf = 0.50 (EtOAc) [UV] [KMnO4].

^{1}H RMN (300 MHz, $CDCl_3$, 300K): δ 9.83 (s, 1H), 7.83 (s, 1H), 7.62 (dt, *J* = 7.4, 1.5 Hz, 1H), 7.47 – 7.35 (m, 4H), 7.33 – 7.23 (m, 2H), 7.18 – 7.12 (m, 2H), 4.40 (q, *J* = 7.1 Hz, 2H), 1.36 (t, *J* = 7.1 Hz, 3H).

^{13}C{^{1}H} NMR (75 MHz, $CDCl_3$, 300K) δ 191.2 (C), 162.0 (C), 140.0 (CH), 138.3 (C), 136.9 (C), 136.6 (C), 134.7 (C), 134.0 (CH), 129.7 (CH), 129.6 (CH), 129.5 (C), 129.4 (CH), 129.2 (CH), 127.9 (CH), 126.5 (CH), 126.4 (C), 61.1 (CH_2), 14.3 (CH_3).

HRMS (EI): Calcd. for $[C_{19}H_{16}N_2O_3S]^+$: 352.0882, found: 352.0893.

Ethyl 1-phenyl-5-(thiophen-2-ylthio)-1H-imidazole-4-carboxylate (9g)

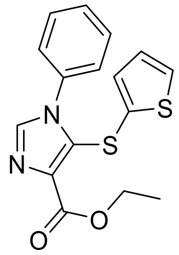

Following **General Procedure A**, isocyanide **4b** (55 μl mg, 0.50 mmol), isothiocyanate **5a** (36 μl, 0.30 mmol), and lithium *tert*-butoxide (48 mg, 0.60 mmol) were dissolved in DMSO. Subsequently, aryl iodide **3g** (42 mg, 0.20 mmol) was added. After purification by column chromatography (Hex/EtOAc, 1:2), compound **9g** was obtained as a orange oil (22 mg, 33% isolated yield).

Rf = 0.41 (1:2 Hex/EtOAc) [UV] [KMnO4].

^{1}H RMN (300 MHz, $CDCl_3$, 300K): δ 7.69 (s, 1H), 7.55 – 7.44 (m, 3H), 7.28 – 7.23 (m, 2H), 7.21 (dd, *J* = 4.6, 2.0 Hz, 1H), 6.78 – 6.73 (m, 2H), 4.47 (q, *J* = 7.1 Hz, 2H), 1.45 (t, *J* = 7.1 Hz, 3H).

^{13}C{^{1}H} NMR (75 MHz, $CDCl_3$, 300K) d 162.2 (C), 139.3 (CH), 136.7 (C), 134.9 (C), 133.8 (CH), 131.2 (C), 130.0 (CH), 129.5 (CH), 129.3 (CH), 127.0 (CH), 61.0 (CH_2), 14.5 (CH_3).

HRMS (EI): Calcd. for $[C_{16}H_{14}N_2O_2S_2]^+$: 330.0497, found: 330.0515.

4. Isolation of the intermediate 6b.

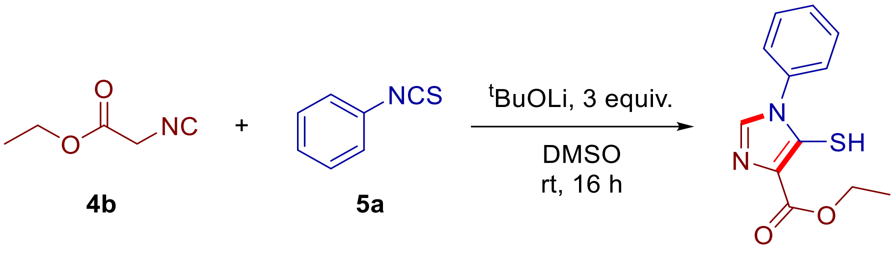

In a 100 mL single-neck flask, isocyanide **4b** (329 μl, 3 mmol, 1.5 equiv.), isothiocyanate **5a** (237 μl, 2 mmol, 1.0 equiv.), lithium *tert*-butoxide (480 mg, 6 mmol, 3.0 equiv.), and DMSO (15 mL) were added. The reaction mixture was stirred for 16 hours at room temperature. The mixture was then acidified with 1 M HCl to pH 5. The aqueous phase was extracted with EtOAc (2 × 20 ml), and the combined organic layers were washed with H_2O (20 ml). The organic phase was dried over anhydrous Na_2SO_4, filtered, and concentrated first by rotary evaporation and then under high vacuum to afford the crude product. Purification by column chromatography (EtOAc) yielded **6b** as a yellow solid (112 mg, 23%). The spectral data match those reported in the literature.[2]

^{1}H RMN (300 MHz, $CDCl_3$, 300K): δ 7.72 (m, 2H), 7.47 – 7.45 (m, 3H), 7.34 – 7.31 (m, 2H), 4.26 (q, *J* = 7.1 Hz, 2H), 1.34 (t, *J* = 7.1 Hz, 3H).

Rf = 0.16 (EtOAc) [UV] [KMnO4].

Starting from thiol **6b**, the corresponding sodium thiolate **Na–6b** was generated by deprotonation with NaH (1.0 equiv.), dissolving compound **6b** in acetonitrile (1mL/1mmol **6b**). This species was subsequently employed in a wide range of mechanistic studies.

[2] J. Zuo, X. Li, Y. Shi, J. Lv, D. Yang. *Org. Lett.* **2024**, 17, 3541–3546.

5. Photochemical isomerization of product 7b.

Because the reaction does not display a clear stereoselective preference for either diastereoisomer, a post-reaction photochemical isomerization was explored to increase the proportion of the cis isomer, following the established principles of olefin *E*/*Z* photoisomerization.[3] The reaction was first conducted according to **General Procedure A**, and after the standard 16 h reaction time, two in situ photochemical isomerization strategies were examined.

The first approach involved direct irradiation, aiming to promote excitation of the *E* isomer to its triplet state, from which isomerization can occur. As an alternative, a sensitized isomerization was evaluated using a photosensitizer (5% mol) capable of transferring triplet energy to the target molecule—in this case, the *E* isomer—to induce *E*→*Z* conversion.

For the direct-irradiation approach, several wavelengths were evaluated. In parallel, a screening of photocatalysts was carried out to identify conditions under which efficient interconversion of the *E* isomer into the *Z* isomer could be achieved. As shown in **Table S2**, the best outcome was obtained under direct irradiation at 390 nm, which afforded the highest stereoselectivity toward the *Z* isomer (**Entry 2**).

---

[3] For a recent review on photochemical E/Z isomerizations, see: T. Neveselý, M. Wienhold, J. J. Molloy, R. Gilmour. Chem. Rev. **2022**, *122*, 2650.

Table 2. Screening of photosensitizers and conditions of direct irradiation for the photochemical E/Z isomerization of **7b**.

Entry	Photocatalyst	ET ($KJ \cdot mol^{-1}$)	λ_{max}	Lamp (nm)	Time (h)	d.r. (*E*:*Z*)[b]
1	-	-	-	370	24	1:1.6
2	-	-	-	390	24	1:2.6
3	-	-	-	427	24	1:2.2
4	Xanthone	310	350	370	24	1:1.9
5	Thioxanthone	265	390	427	24	1.1:1
6	$Ir[dF(CF_3)ppy]_2(dtbpy))PF_6$	251	389	427	24	1.6:1
7	9-Fluorenone	211	230	370	24	2.2:1
8	$[Ru(bpy)_3][PF_6]_2$	205	451	427	24	1:0.8
9	$[Ru(bpy)_3]Cl_2$	194	452	427	24	1:0.5
10	Eosin Y	190	539	525	24	1:0.4

6. UV-Vis spectra.

All measurements were carried out by dissolving the reagents in DMSO.

- Sample of **1a**: 5.8 µL (0.045 mmol) of bromostyrene (**1a**) were dissolved in 3 mL of DMSO, to achieve a final concentration of 15 mM.
- Sample of **6b-Na**: 18.2 mg (0.067 mmol) of the sodium salt of thiol **6b** were dissolved in 3 mL of DMSO, to achieve a final concentration of 22.5 mM.
- Mixed sample: 5.8 µL (0.045 mmol) of bromostyrene (**1a**) were added to a solution of 18.2 mg (0.067 mmol) of the sodium salt of thiol **6b** in 3 mL of DMSO.

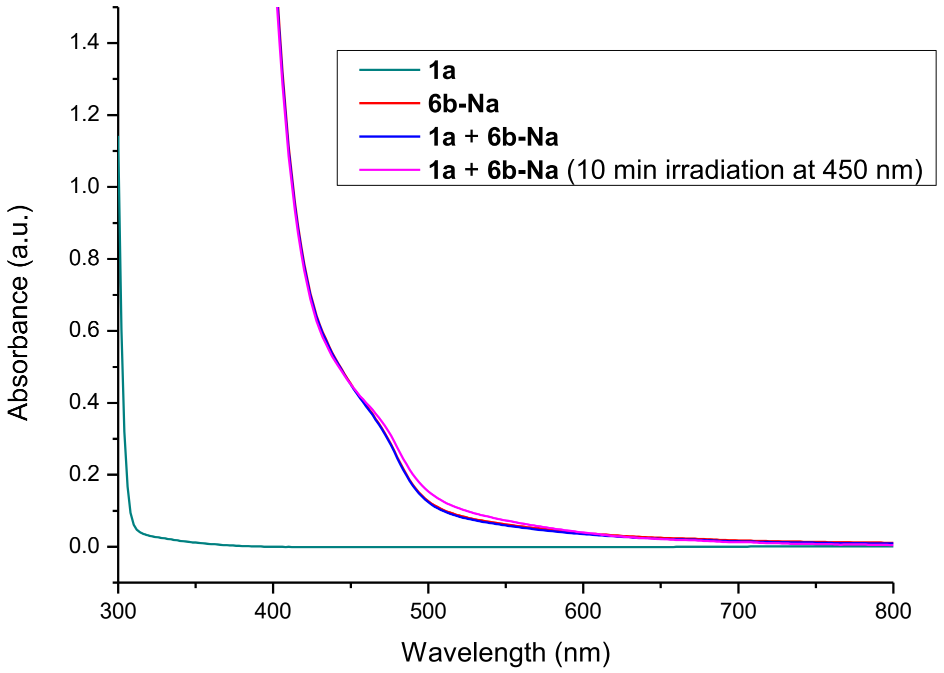

Figure SI- 5. UV-Vis spectra of compounds 1a, thiolate 6b-Na, and the mixture of both before and after 10 min of irradiation at 450 nm.

Upon mixting thiolate (**6b-Na**) and bromostyrene (**1a**) there is no CT band observed. Only after irradiating the sample at 450 nm for 10 min, a small red-shift is observed (Figure SI-5).

7. NMR titration experiments.

For these experiments, solutions with increasing concentrations of one of the two reactants forming the HaB were prepared, and the chemical shift variations of their NMR signals were monitored. To enable direct NMR analysis, the samples were prepared using d⁶-DMSO as the solvent. Details regarding the composition of each sample are provided below.

- Sample 1: In the empty vial, bromostyrene **1a** (0.10 mmol, 13 μl) was added. The mixture was then stirred for 30 minutes prior to preparing the NMR sample.

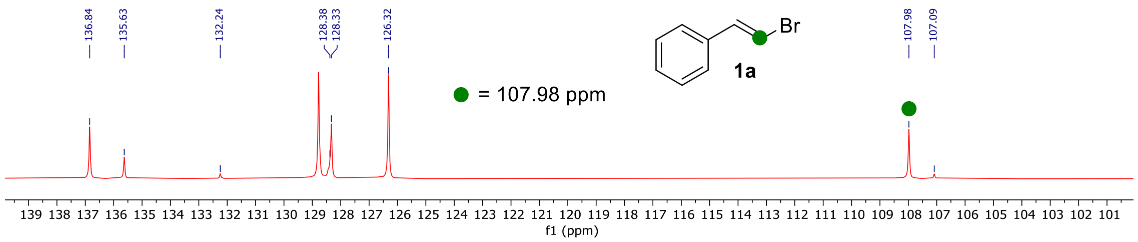

Figure SI-6. ^{13}C-NMR spectra recorded for the Sample 1 in d⁶-DMSO.

Chemical shift (●): 107.98 ppm.

- Sample 2: **1a** (65 μl, 0.50 mmol) was added to the mixture of thiol **6b** (0.25 mg, 0.1 mmol) and NaOH (6 mg, 0.15 mmol).

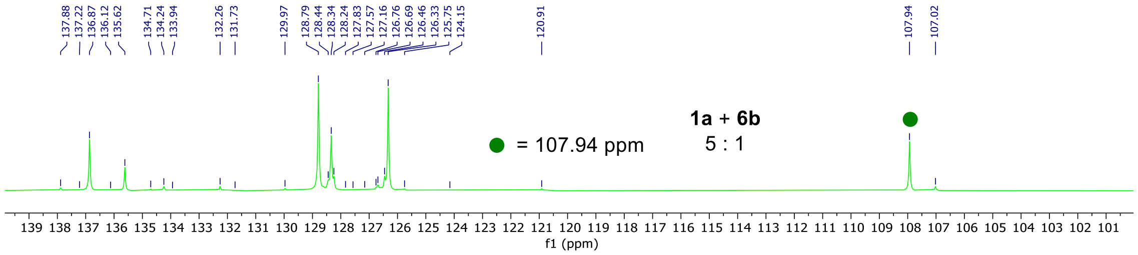

Figure SI-7. ^{13}C-NMR spectra recorded for the Sample 2 in d⁶-DMSO.

Chemical shift (●): 107.94 ppm.

- Sample 3: **1a** (129 μl, 1.00 mmol) was added to the mixture of thiol **6b** (0.25 mg, 0.10 mmol) and NaOH (6 mg, 0.15 mmol).

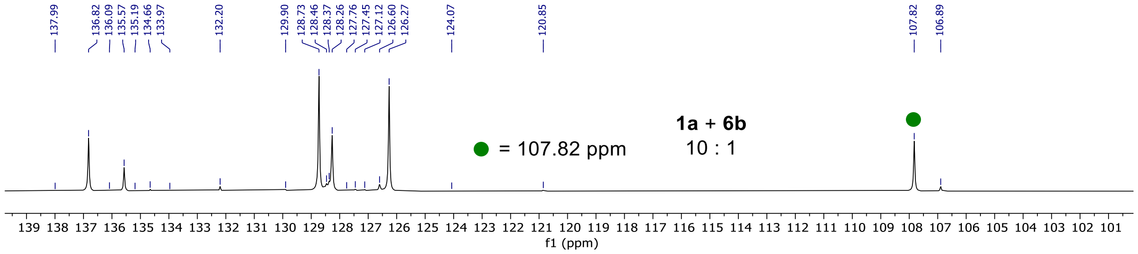

Figure SI-8. ^{13}C-NMR spectra recorded for the Sample 3 in d^{6}-DMSO.

Chemical shift (●): 107.82 ppm.

In this study, the amount of **1a** was increased while keeping **6b** constant, using 5:1 and 10:1 (**1a:6b**) ratios. As shown in Figures SI-6 to SI-8, the stacked ^{13}C NMR spectra display just a slight upfield shift (0.16 ppm) of the marked resonances as the concentration of **1a** increases.

8. Quenching experiments.

The following experiments were performed exciting at 440 nm (slits 20 nm, 0.05 s integration time) and recording the fluorescence from 470 to 750 nm. to a solution of the sodium salt of thiol **6b** (**6b-Na**, 18.2 mg, 0.067 mmol) in 3 mL of DMSO, sequential additions of bromostyrene (**1a**) were performed, to achieve final concentrations ranging from 0.03 to 0.30 M (Figures SI-9 and SI-10).

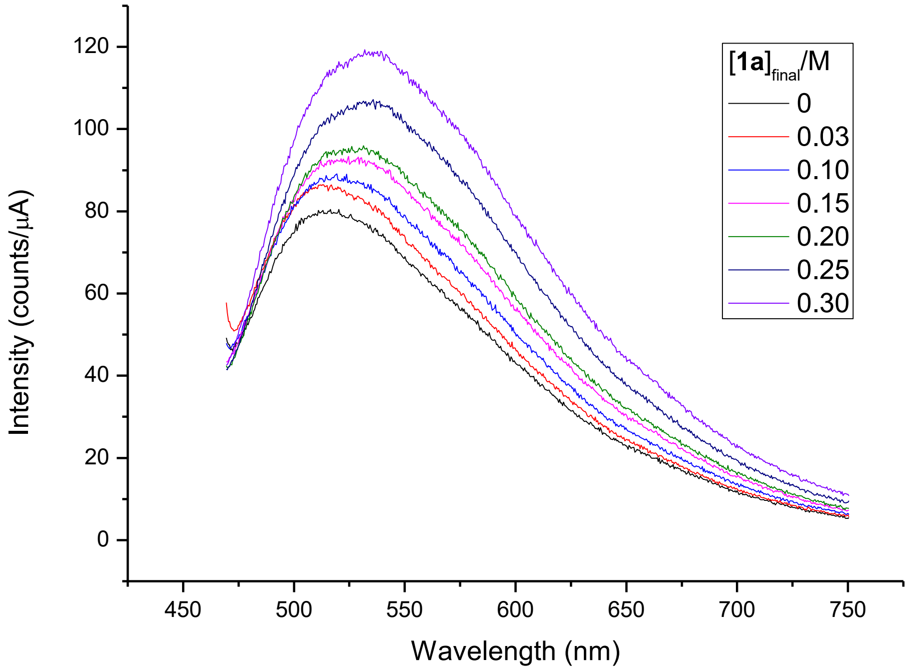

Figure SI-9. Fluorescence spectra of the sodium salt of thiol **6b** (**6b-Na**), and after addition of different equivalents of bromostyrene (**1a**) in DMSO.

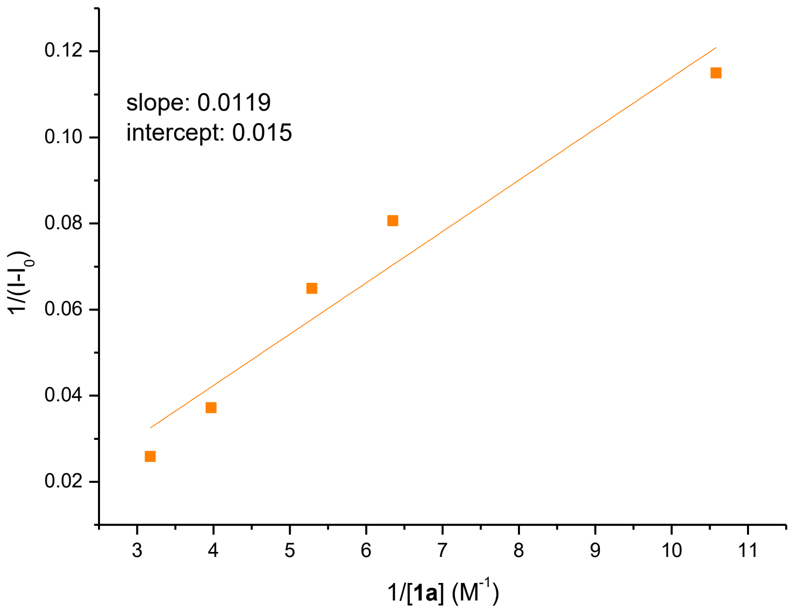

Figure SI-10. Representation of $1/(I-I_0)$ vs 1/[**1a**] for the sodium salt of thiol **6b** (**6b-Na**) using bromostyrene (**1a**) as quencher.

Interestingly, fluorescence titration experiments revealed a progressive increase in emission intensity upon the addition of **1a** to thiolate **6b-Na**. Therefore, the classical Stern–Volmer treatment is not applicable and requires correction. Accordingly, the Benesi Hidebrand equation (valid for high quencher concentrations) was used instead:

$$\frac{1}{I - I_0} = \frac{1}{(I_{max} - I_0)K_a[Q]} + \frac{1}{I_{max} - I_0}$$

With [Q] representing the quencher concentration (**1a**), I_0 the absorbance of receptor in the absence of guest, I the absorbance recorded in the presence of added guest, I_{max} the maximum fluorescence intensity when the fluorophore (**6b-Na**) is fully complexed as an exciplex, and K_a, the association constant of the complex using the Benesi Hidebrand approach, where a 1:1 stoichiometry is assumed. From this representation, the calculated values are I_{max} = 146.67 and K_a = 1.26 M^{-1}. The relatively low association constant indicates a weak-to-moderate interaction in the excited state, consistent with a transient and dynamic exciplex rather than a strongly bound ground-state complex. Moreover, the I_{max} value reflects a limited but measurable enhancement of fluorescence upon full exciplex formation, supporting the notion that emissive exciplexes are formed only under high quencher concentrations.

9. Calculation of the oxidation potential of the excited state of 6b-Na.

The calculation of the oxidation potential of the excited state of thiolate 6b-Na was calculated determining $E_{0,0}$ from the absorbance and fluorescence spectra and the oxidation potential of the ground state of this compound.

The UV-visible spectra of sodium salt of thiol **6b** was recorded by adding the thiolate (**6b-Na**, 18.2 mg, 0.067 mmol) in 3 mL of DMSO. Excitation at 440 nm. The fluorescence curve was normalized so that the maximum fluorescence (510 nm) equals the absorbance at 440 nm. The crossing point was determined at 470 nm → $E_{0,0}$ = 2.64 eV. (Figure SI-11).

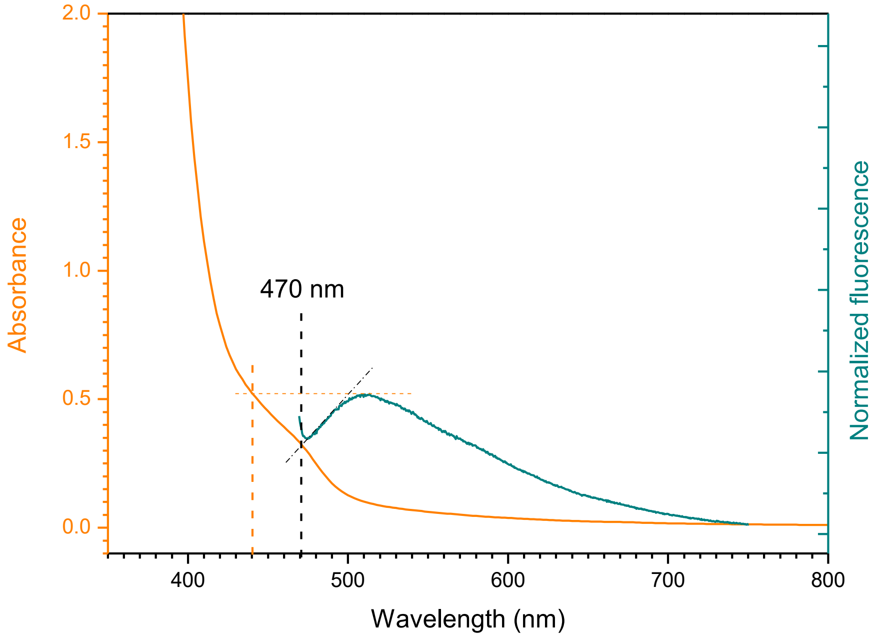

Figure SI-11. Absorbance spectra of **1a** and **6b-Na** and flourescence spectra upon excitation at 440 nm. Crossing point of both curves observed at 470 nm, for the the $E_{0,0}$ determination in DMSO.

The oxidation potential of thiolate **6b-Na** was determined measuring the cyclic voltammetry in DMSO. The value for the peak potential was determined to be E_p = +0.16 V vs Ag/AgCl (Figure SI-12).

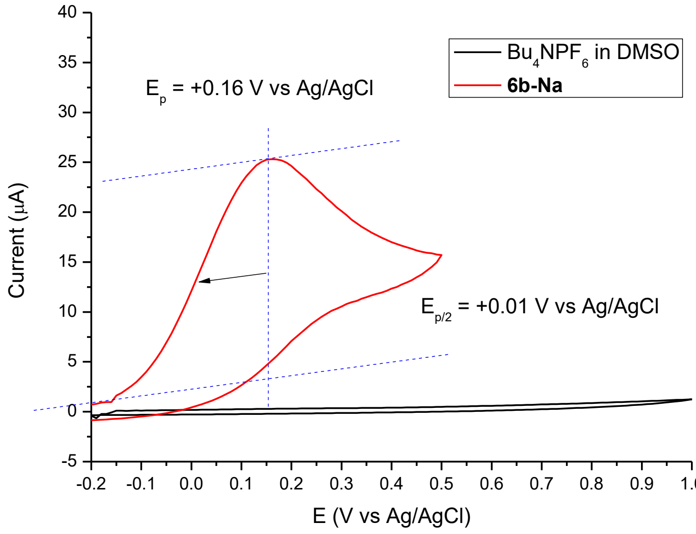

Figure SI-12. E_p and $E_{p/2}$ of **6b-Na** vs Ag/AgCl in DMSO for the calculations.

Calculation:

Excitation wavelength: 440 nm; Fluorescence maximum: 470 nm

Crossing wavelength: 470 nm → $E_{0,0}$ = 2.64 eV

From voltametric experiments: E_p = +0.16 V vs Ag/AgCl (0.1 M) in DMSO

Therefore, the potential of the excited state, according to the Rehm-Weller equation, would be:

$E^*_{\text{in DMSO}}$= + 0.16 – 2.64 = –2.48 V vs Ag/AgCl

Considering that the potential of ferrocene *vs.* SCE is +0.40 V in DMSO and that our Fc measurement is +0.420 V vs Ag/AgCl (0.1 M) in DMSO, there is a reference correction factor of 0.420-0.400 = 0.020 V.

The reduction potential of the excited state of **6b-Na** would be:

$E^*_{\text{in DMSO}}$= –2.48 V vs Ag/AgCl

$E^*_{\text{in DMSO}}$= –2.48 – 0.42 = **–2.90 V vs Fc**

$E^*_{\text{in DMSO}}$= –2.48 – 0.02 = **–2.50 V vs SCE**

10. Cyclic voltammetries.

The measurements were carried out in DMSO (3 mL each), using Bu_4NPF_6 as the supporting electrolyte (0.1 M). Solutions were bubbled with Ar for 5 min before each measurement. The scanning speed was set at 100 mV s^{-1}. The glassy carbon electrode was polished between every scan to ensure cleanliness. Potentials were corrected vs. ferrocene (Fc) using the correction factor obtained for Fc in DMSO vs Ag/AgCl (0.1 M) (Figure SI-13).

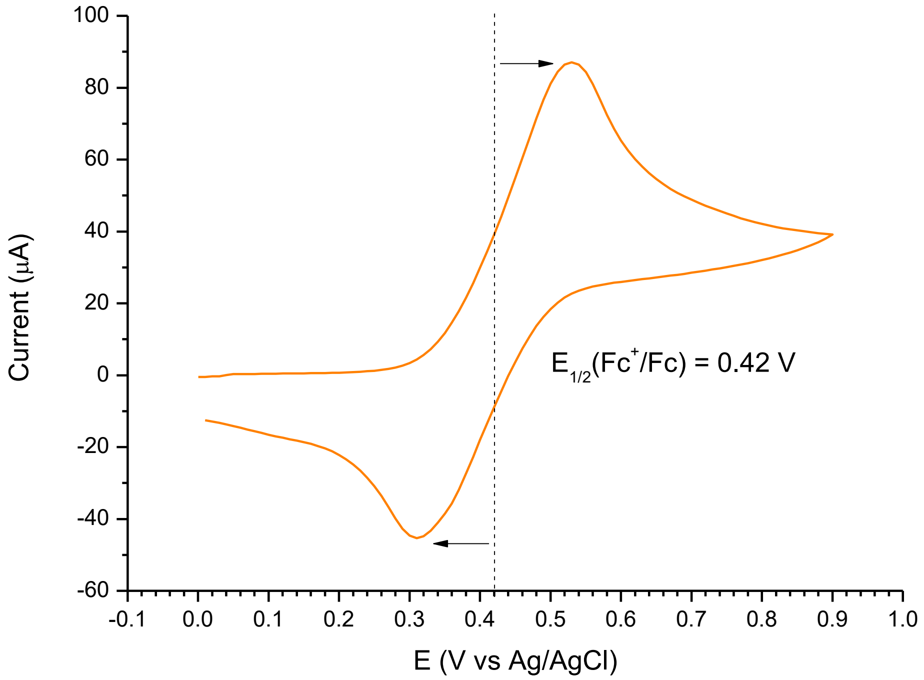

Figure SI-13. CV of ferrocene in DMSO for Ag/AgCl to Fc^+/Fc correction.

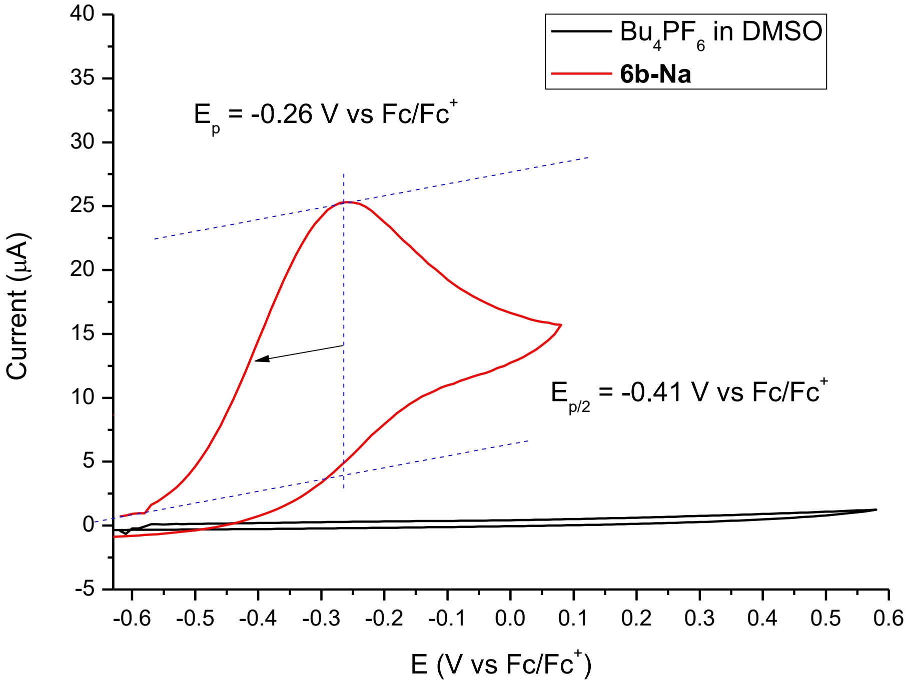

Figure SI-14. E_p and $E_{p/2}$ of thiolate **6b-Na** in DMSO.

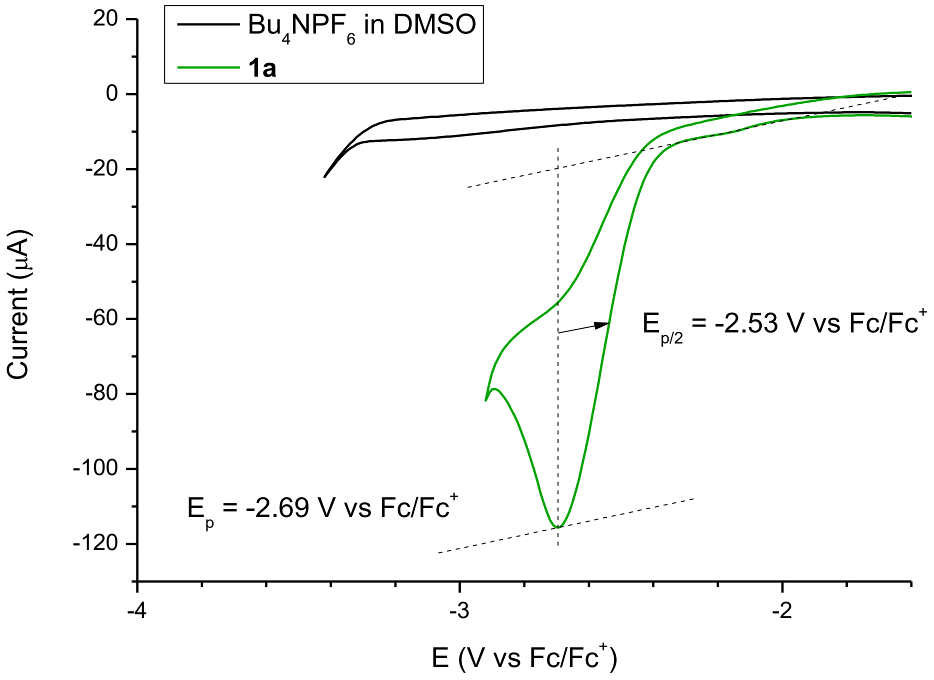

Figure SI-15. E_p and $E_{p/2}$ of bromostyrene (**1a**) in DMSO.

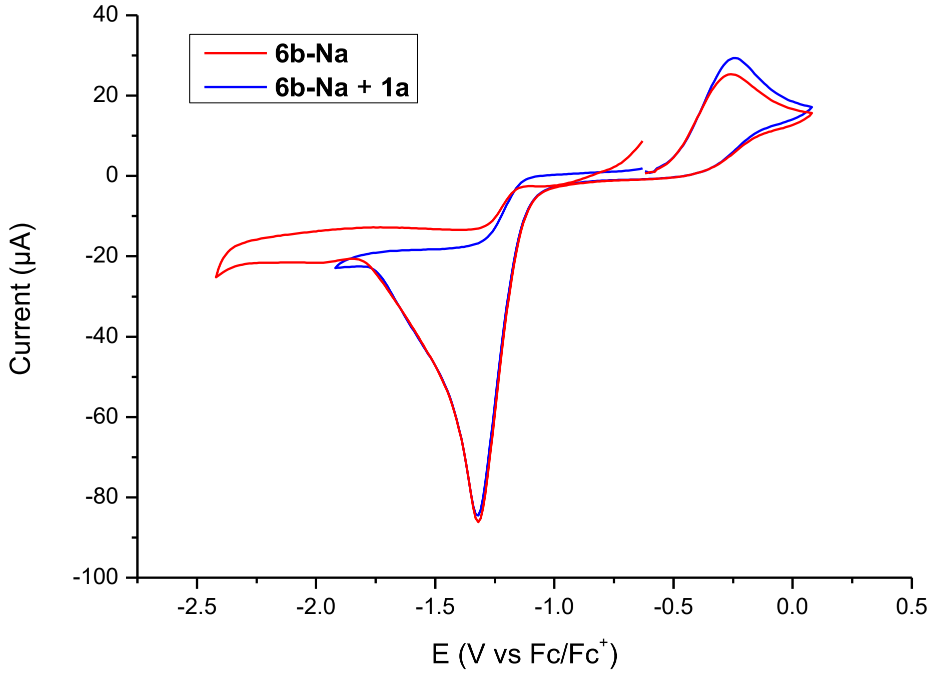

Figure SI-16. CV of thiolate 6b-Na in DMSO and upon addition of bromostyrene (**1a**).

The lack of observable changes when mixing **6b-Na** and **1a** indicates that both components remain independent in the ground state, precluding the existence of a ground-state EDA complex.

11. Computational calculations.

Geometry optimizations of the molecules used in the main manuscript were performed without symmetry constraints using the Orca 6.1.0[1] suite of program at the PBE0[2]-D3[3]BJ[4]/def2-TZVP[5] level, in combination with the keywords DEFGRID3 and VERYTIGHTOPT. Reactants and products were characterized by frequency calculations and have positive definite Hessian matrices. Solvent effects were also considered during both the geometry optimizations and the single-point calculations by means of the conductor-like polarizable continuum (CPCM)[6] for dimethysulfoxide, as implemented in ORCA. This level is denoted CPCM(dimethylsulfoxide)-PBE0-D3BJ/def2-TZVP. The computed thermochemistry data were computed at 298.15 K.The Resolution of Identity approximation for Coulomb and HF Exchange with the Chain of Spheres algorithm (RIJCOSX)[7] was used whenever possible to speed up the calculations. Time Dependent Density Functional Theory, as implemented in ORCA, was used to study the presented singlet excited states. During the calculation of vertical transitions and optimization of the S1 states, the lowest 10 roots were computed to avoid root flipping. The Electron-Hole analysis, as implemented and proposed in Multiwfn[8] was used to understand the nature of the excitations. The Conformer-Rotamer Ensemble Sampling Tool (CREST)[9] was used to assess different supramolecular conformations of the EDA complexes, generating and initial ensemble from which selected geometries were optimized with DFT.

References

[1] Neese F. Software update: the ORCA program system -- Version 6.0. *WIREs Comput Mol Sci*. **2025**, *15*, e70019. https://doi.org/10.1002/wcms.70019.

[2] Adamo. C.; Barone, V. Toward reliable density functional methods without adjustable parameters: The PBE0 model. *J. Chem. Phys.* **1999**, *110*, 6158–6170. https://doi.org/10.1063/1.478522.

[3] Grimme. S.; Antony J.; Ehrlich. S.; Krieg H. A consistent and accurate ab initio parametrization of density functional dispersion correction (DFT-D) for the 94 elements H-Pu. *J. Chem. Phys*. **2010**, *132*, 154104–154122. https://doi.org/10.1063/1.3382344.

[4] Becke A. D.; Johnson E. R A density-functional model of the dispersion interaction. *J. Chem. Phys.* **2025**, *123*, 154101–154109. https://doi.org/10.1063/1.2065267.

[5] Weigend F.; Alhrichs R. Balanced basis sets of split valence, triple zeta valence and quadruple zeta valence quality for H to Rn: Design and assessment of accuracy. *Phys. Chem. Chem. Phy*s. **2005**, *7*, 3297–3305. https://doi.org/10.1039/B508541A.

[6] Barone V.; Cossi M. Quantum Calculation of Molecular Energies and Energy Gradients in Solution by a Conductor Solvent Model. *J. Phys. Chem. A,* **1998**, *102*, 1995–2001. https://doi.org/10.1021/jp9716997.

[7] Helmich-Paris B.; de Souza B.; Neese F.; Izsák R. An improved chain of spheres for exchange algorithm. *J. Chem. Phys.* **2021**, *155*, 104109–104122. https://doi.org/10.1063/5.0058766.

[8] Lu, T.; Chen, F. Multiwfn: A multifunctional wavefunction analyzer. *J. Comput. Chem.* **2012**, *33* 580-592. https://doi.org/10.1002/jcc.22885.

[9] Grimme S. Exploration of Chemical Compound, Conformer, and Reaction Space with Meta-Dynamics Simulations Based on Tight-Binding Quantum Chemical Calculations, *Chem. Theory Comput.* **2019**, *15*, 2847–2862. https://doi.org/10.1021/acs.jctc.9b00143

Cartesian coordinates

Cartesian coordinates (in Å) and total energies (in Hartree) of all the stationary points discussed in the text. E_{el}, E_{el+ZPE} and G represent total electronic energies, electronic energies with Zero Point Correction included and Gibbs free energies, respectively.

1a

N_{imag} = 0

E_{el} = -2882.662233

E_{el+ZPE} = -2882.538154

G = -2882.572436

C	4.370615000000	-1.670351000000	1.952150000000
C	4.122282000000	-0.375176000000	2.110957000000
H	4.649707000000	0.289522000000	2.780937000000
C	5.401918000000	-2.431505000000	2.656389000000
C	6.245099000000	-1.873949000000	3.623648000000
C	5.551097000000	-3.785267000000	2.345574000000
C	7.203567000000	-2.648416000000	4.252501000000
C	6.512315000000	-4.560892000000	2.976512000000
C	7.342974000000	-3.995266000000	3.932590000000
H	6.152799000000	-0.827562000000	3.890543000000
H	4.902257000000	-4.229410000000	1.597878000000
H	7.848275000000	-2.199886000000	4.999914000000
H	6.611191000000	-5.609507000000	2.719721000000
H	8.095443000000	-4.597670000000	4.428418000000
H	3.772859000000	-2.235606000000	1.242556000000
Br	2.766229000000	0.509384000000	1.149450000000

6b-S_0

***N_{imag}* =** 0

***E_{el}* =** -1121.566523

E_{el+ZPE} = -1121.353265

***G* =** -1121.3949719

S	-0.233092000000	0.081102000000	1.689209000000
N	-3.128901000000	0.697868000000	-0.976092000000
C	-2.925066000000	-0.580965000000	-1.017659000000
C	-2.229068000000	1.189773000000	-0.044867000000
C	-1.449326000000	0.152773000000	0.494683000000
N	-1.927258000000	-0.972153000000	-0.166993000000
H	-3.440785000000	-1.294079000000	-1.645172000000
C	-1.503216000000	-2.312994000000	-0.008990000000
C	-2.418899000000	-3.269018000000	0.407745000000
C	-0.194065000000	-2.672416000000	-0.303071000000
C	-2.023020000000	-4.594455000000	0.527522000000
C	0.197233000000	-3.995336000000	-0.171954000000
C	-0.714951000000	-4.958638000000	0.242790000000
H	-3.434511000000	-2.970402000000	0.640844000000
H	0.505052000000	-1.915010000000	-0.633208000000
H	-2.739476000000	-5.340737000000	0.850780000000
H	1.218485000000	-4.276863000000	-0.401722000000
H	-0.405114000000	-5.992665000000	0.341233000000
C	-2.171875000000	2.600341000000	0.275137000000
O	-1.416402000000	3.130334000000	1.070419000000
O	-3.076469000000	3.313930000000	-0.423592000000

C	-3.094693000000	4.722038000000	-0.177780000000
H	-3.294950000000	4.899055000000	0.882082000000
H	-2.110227000000	5.139096000000	-0.405067000000
C	-4.164086000000	5.324486000000	-1.049511000000
H	-3.954047000000	5.145973000000	-2.106179000000
H	-5.144027000000	4.904542000000	-0.813026000000
H	-4.204839000000	6.403301000000	-0.885467000000

6b-S$_1$

***N*$_{imag}$ =** 0

***E*$_{el}$ =** -1121.438276

E$_{el+ZPE}$ = -1121.230035

***G* =** -1121.272493

S	-0.358446000000	0.197804000000	1.726149000000
N	-3.074067000000	0.704341000000	-1.041487000000
C	-2.874868000000	-0.612275000000	-1.010605000000
C	-2.170774000000	1.219677000000	-0.192965000000
C	-1.421093000000	0.184050000000	0.437577000000
N	-1.915693000000	-0.987532000000	-0.145461000000
H	-3.403869000000	-1.329899000000	-1.620070000000
C	-1.511796000000	-2.305899000000	0.127555000000
C	-2.487921000000	-3.334835000000	0.215922000000
C	-0.129372000000	-2.673004000000	0.015963000000
C	-2.106133000000	-4.650401000000	0.287330000000
C	0.224482000000	-3.998839000000	0.093912000000
C	-0.733827000000	-5.019744000000	0.248735000000

H	-3.539210000000	-3.067196000000	0.262222000000
H	0.623717000000	-1.907111000000	-0.119899000000
H	-2.869284000000	-5.415615000000	0.388805000000
H	1.276529000000	-4.261214000000	0.023626000000
H	-0.436310000000	-6.059413000000	0.290888000000
C	-2.020089000000	2.669949000000	-0.007103000000
O	-1.040064000000	3.208181000000	0.460790000000
O	-3.093565000000	3.335640000000	-0.431327000000
C	-3.030058000000	4.766972000000	-0.336859000000
H	-2.880953000000	5.042545000000	0.709672000000
H	-2.165642000000	5.117403000000	-0.905569000000
C	-4.318839000000	5.318087000000	-0.882019000000
H	-4.454883000000	5.033476000000	-1.927225000000
H	-5.173449000000	4.957822000000	-0.306003000000
H	-4.300007000000	6.408097000000	-0.822653000000

EDA-S_0

N_{imag} = 0

E_{el} = -4004.241308

E_{el+ZPE} = -4003.903225

G = -4003.957081

S	1.309876000000	0.894320000000	3.041756000000
N	-0.056879000000	-0.035517000000	-0.581940000000
C	0.738372000000	-1.014075000000	-0.283533000000
C	-0.031635000000	0.813656000000	0.511215000000
C	0.818536000000	0.315986000000	1.512666000000

N	1.286786000000	-0.871905000000	0.962338000000
H	0.959272000000	-1.873644000000	-0.900222000000
C	2.152026000000	-1.825317000000	1.549284000000
C	1.822085000000	-2.418421000000	2.762096000000
C	3.306811000000	-2.196313000000	0.876201000000
C	2.659448000000	-3.381702000000	3.301454000000
C	4.134632000000	-3.170563000000	1.416529000000
C	3.816182000000	-3.761649000000	2.630174000000
H	0.914107000000	-2.122553000000	3.270798000000
H	3.562922000000	-1.709923000000	-0.057358000000
H	2.402671000000	-3.844906000000	4.247293000000
H	5.037762000000	-3.455327000000	0.889369000000
H	4.466732000000	-4.517737000000	3.054581000000
C	-0.807686000000	2.035554000000	0.530322000000
O	-0.791782000000	2.887426000000	1.401029000000
O	-1.594129000000	2.151627000000	-0.556456000000
C	-2.349690000000	3.359609000000	-0.659753000000
H	-3.057818000000	3.413741000000	0.171490000000
H	-1.672554000000	4.213455000000	-0.576600000000
C	-3.056510000000	3.344121000000	-1.988721000000
H	-2.338442000000	3.300114000000	-2.810443000000
H	-3.728029000000	2.486421000000	-2.065970000000
H	-3.649140000000	4.254636000000	-2.098987000000
C	3.179130000000	2.127003000000	-0.592070000000
C	2.746999000000	2.551413000000	-1.773842000000
H	3.153555000000	2.256495000000	-2.731361000000

C	4.284900000000	1.189933000000	-0.395973000000
C	4.821120000000	0.412861000000	-1.427814000000
C	4.837008000000	1.071796000000	0.881367000000
C	5.888406000000	-0.435517000000	-1.188537000000
C	5.907283000000	0.223472000000	1.119101000000
C	6.440337000000	-0.530590000000	0.084359000000
H	4.393739000000	0.459823000000	-2.422798000000
H	4.414832000000	1.653602000000	1.693666000000
H	6.288392000000	-1.034507000000	-1.998915000000
H	6.319624000000	0.145427000000	2.118689000000
H	7.272093000000	-1.200764000000	0.268583000000
H	2.711708000000	2.505260000000	0.312505000000
Br	1.332758000000	3.781405000000	-1.945856000000

EDA'-S0

N N_{imag} = 1, -3 cm^{-1}

E_{el} = -4004.235048

E_{el+ZPE} = -4003.897250

G = -4003.951290

S	0.378089000000	1.865266000000	-1.001399000000
N	-3.256157000000	0.374035000000	-0.339029000000
C	-2.509776000000	-0.673227000000	-0.492772000000
C	-2.406751000000	1.460279000000	-0.466843000000
C	-1.087242000000	1.040382000000	-0.707645000000
N	-1.198430000000	-0.346680000000	-0.709837000000
H	-2.831314000000	-1.703915000000	-0.443269000000

C	-0.180182000000	-1.307115000000	-0.916015000000
C	-0.328468000000	-2.239959000000	-1.933765000000
C	0.932665000000	-1.343249000000	-0.085611000000
C	0.639926000000	-3.216689000000	-2.119507000000
C	1.902871000000	-2.312707000000	-0.285649000000
C	1.758993000000	-3.251705000000	-1.300172000000
H	-1.196956000000	-2.191893000000	-2.580476000000
H	1.029987000000	-0.613737000000	0.707524000000
H	0.522820000000	-3.943450000000	-2.915085000000
H	2.772623000000	-2.340367000000	0.360662000000
H	2.519437000000	-4.009524000000	-1.450975000000
C	-2.902793000000	2.816347000000	-0.357048000000
O	-2.240170000000	3.836143000000	-0.423263000000
O	-4.235894000000	2.851543000000	-0.163220000000
C	-4.819988000000	4.150040000000	-0.034916000000
H	-4.359757000000	4.668814000000	0.809990000000
H	-4.608633000000	4.729825000000	-0.937030000000
C	-6.300338000000	3.967582000000	0.169311000000
H	-6.751816000000	3.452301000000	-0.681088000000
H	-6.502344000000	3.390405000000	1.074068000000
H	-6.778302000000	4.944040000000	0.271734000000
C	3.608239000000	-2.047788000000	-4.560523000000
C	3.390962000000	-0.758749000000	-4.799899000000
H	3.769878000000	-0.207424000000	-5.649852000000
C	4.394369000000	-2.955567000000	-5.393864000000
C	5.051423000000	-2.555636000000	-6.562381000000

C	4.493135000000	-4.292493000000	-5.000355000000
C	5.779401000000	-3.466288000000	-7.307402000000
C	5.222630000000	-5.204883000000	-5.747987000000
C	5.869012000000	-4.795237000000	-6.905112000000
H	4.995294000000	-1.524746000000	-6.892629000000
H	3.987638000000	-4.613550000000	-4.095286000000
H	6.283033000000	-3.138841000000	-8.210024000000
H	5.285767000000	-6.237786000000	-5.424672000000
H	6.440832000000	-5.504629000000	-7.492291000000
H	3.169470000000	-2.491868000000	-3.671383000000
Br	2.350062000000	0.296523000000	-3.635603000000

13-S$_1$

***N*$_{imag}$** = 0

***E*$_{el}$** = -4004.140787

E$_{el+ZPE}$ = -4003.805445

G = -4003.859043

S	0.756686000000	0.626910000000	3.041936000000
N	0.211194000000	-0.389695000000	-0.688394000000
C	1.059110000000	-1.297502000000	-0.228404000000
C	-0.071152000000	0.397362000000	0.357694000000
C	0.625132000000	-0.025273000000	1.525914000000
N	1.332334000000	-1.138887000000	1.074816000000
H	1.483116000000	-2.104678000000	-0.806512000000
C	2.234070000000	-1.936626000000	1.828695000000
C	1.772541000000	-2.644730000000	2.928406000000

C	3.560858000000	-1.995086000000	1.435859000000
C	2.663466000000	-3.425850000000	3.646765000000
C	4.442217000000	-2.786230000000	2.158131000000
C	3.997047000000	-3.498180000000	3.262703000000
H	0.728221000000	-2.587080000000	3.208892000000
H	3.900487000000	-1.419125000000	0.582933000000
H	2.313042000000	-3.984591000000	4.506509000000
H	5.482126000000	-2.836320000000	1.856795000000
H	4.689356000000	-4.111989000000	3.827246000000
C	-0.969613000000	1.562194000000	0.245825000000
O	-1.512204000000	2.097605000000	1.183279000000
O	-1.119128000000	1.937284000000	-1.016993000000
C	-1.958323000000	3.083135000000	-1.242566000000
H	-2.975472000000	2.834433000000	-0.931228000000
H	-1.598409000000	3.902546000000	-0.617522000000
C	-1.882596000000	3.420943000000	-2.705295000000
H	-0.859204000000	3.668745000000	-2.994130000000
H	-2.230168000000	2.588230000000	-3.319735000000
H	-2.516119000000	4.286378000000	-2.908989000000
C	3.298689000000	2.565659000000	0.330164000000
C	2.032677000000	2.952619000000	-0.077247000000
H	1.567732000000	2.598147000000	-0.987489000000
C	4.042145000000	1.473670000000	-0.181456000000
C	3.622272000000	0.678531000000	-1.289540000000
C	5.282631000000	1.102852000000	0.410944000000
C	4.399119000000	-0.358886000000	-1.767205000000

C	6.045337000000	0.065625000000	-0.075098000000
C	5.620323000000	-0.690512000000	-1.178498000000
H	2.680882000000	0.900848000000	-1.778181000000
H	5.634472000000	1.670800000000	1.267704000000
H	4.041331000000	-0.933664000000	-2.616722000000
H	6.987321000000	-0.172074000000	0.409798000000
H	6.216788000000	-1.512187000000	-1.555100000000
H	3.748423000000	3.099099000000	1.164183000000
Br	1.467055000000	4.783774000000	0.260829000000

12. Copies of the NMR spectra.

1-phenyl-5-(styrylthio)-4-tosyl-1H-imidazole (7a)

^{1}H RMN (300 MHz, $CDCl_3$, 300K)

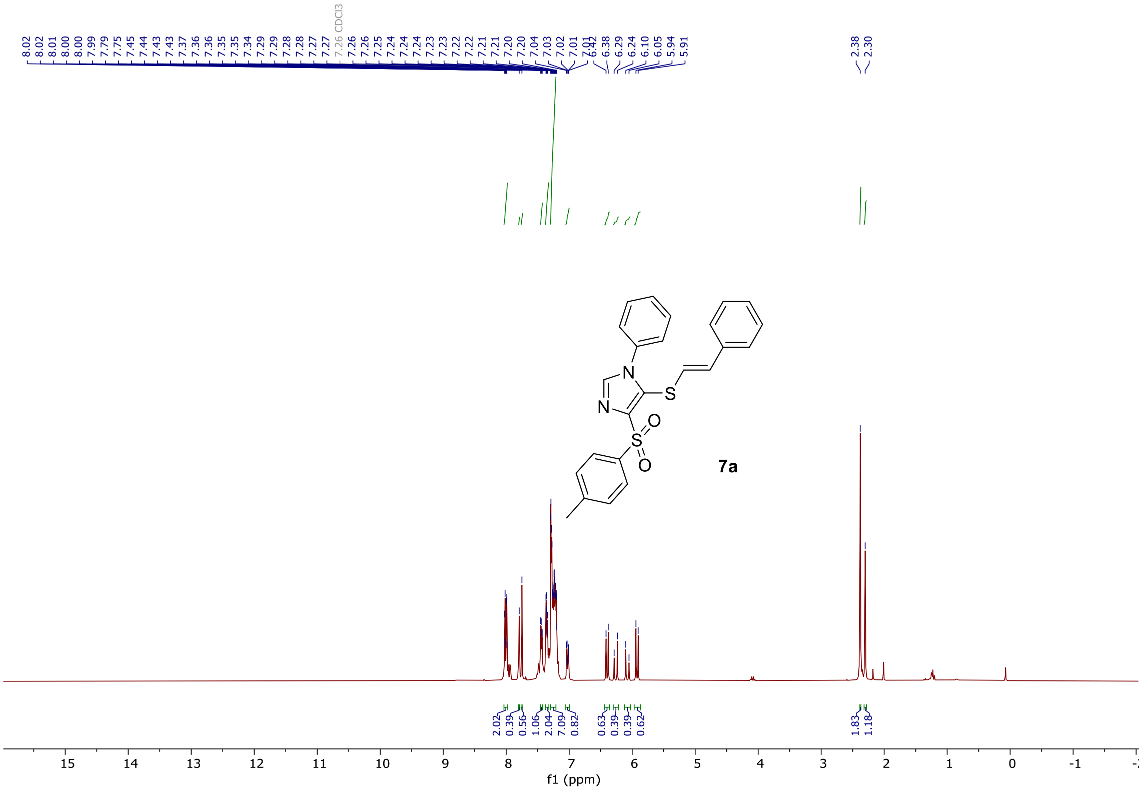

$^{13}C\{^{1}H\}$ NMR (75 MHz, $CDCl_3$, 300K)

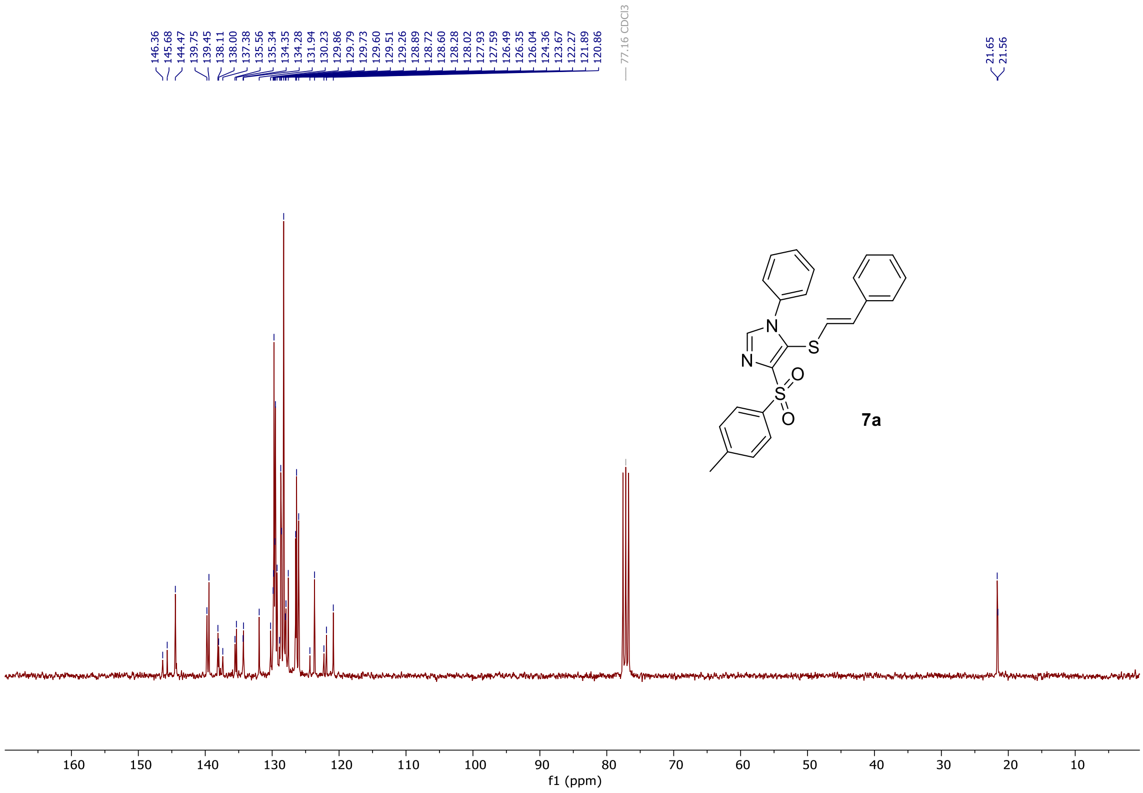

(*E*)-Ethyl 1-phenyl-5-(styrylthio)-1H-imidazole-4-carboxylate (7b)

^{1}H RMN (300 MHz, $CDCl_3$, 300K)

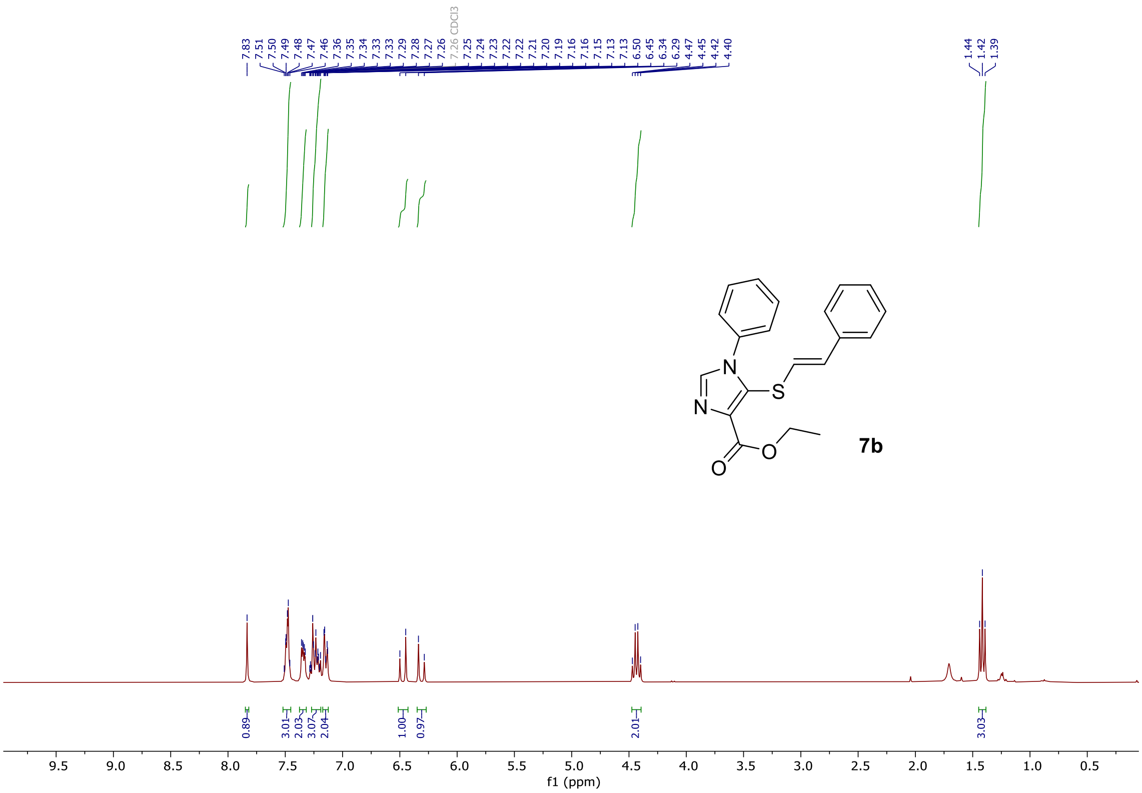

^{13}C{^{1}H} NMR (75 MHz, $CDCl_3$, 300K)

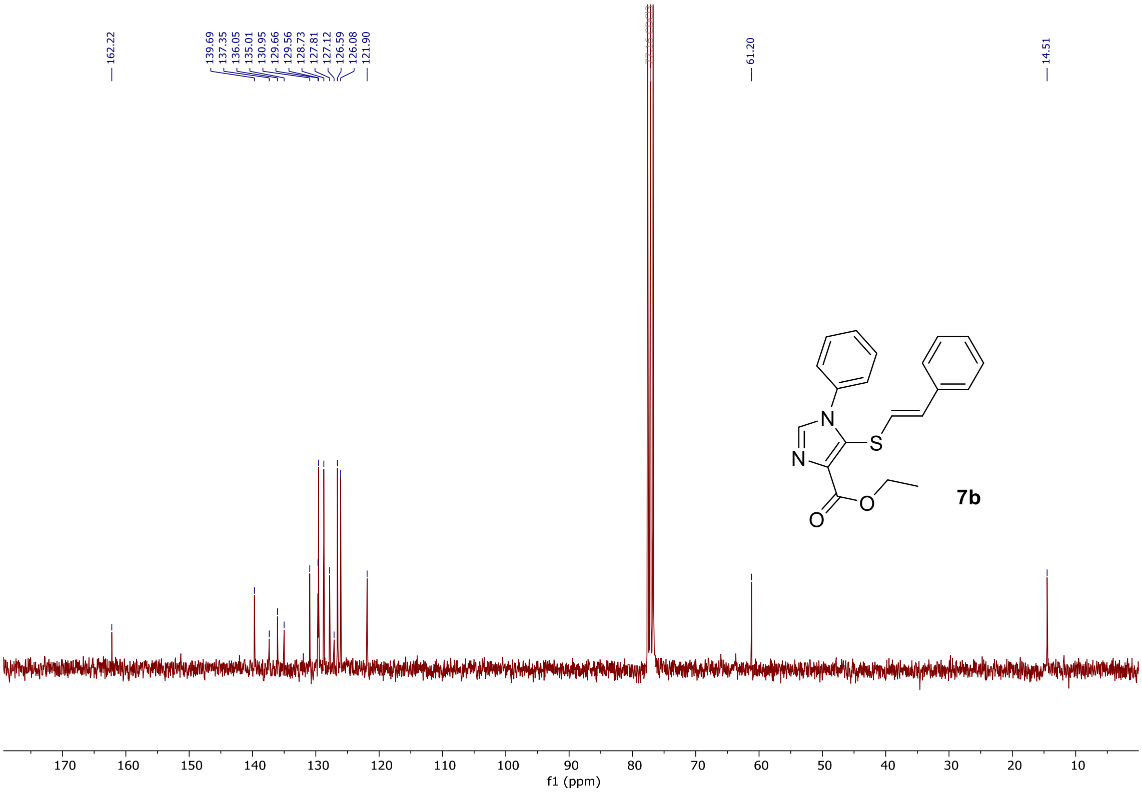

(*Z*)-Ethyl 1-phenyl-5-(styrylthio)-1H-imidazole-4-carboxylate (7b')

^{1}H RMN (300 MHz, $CDCl_3$, 300K)

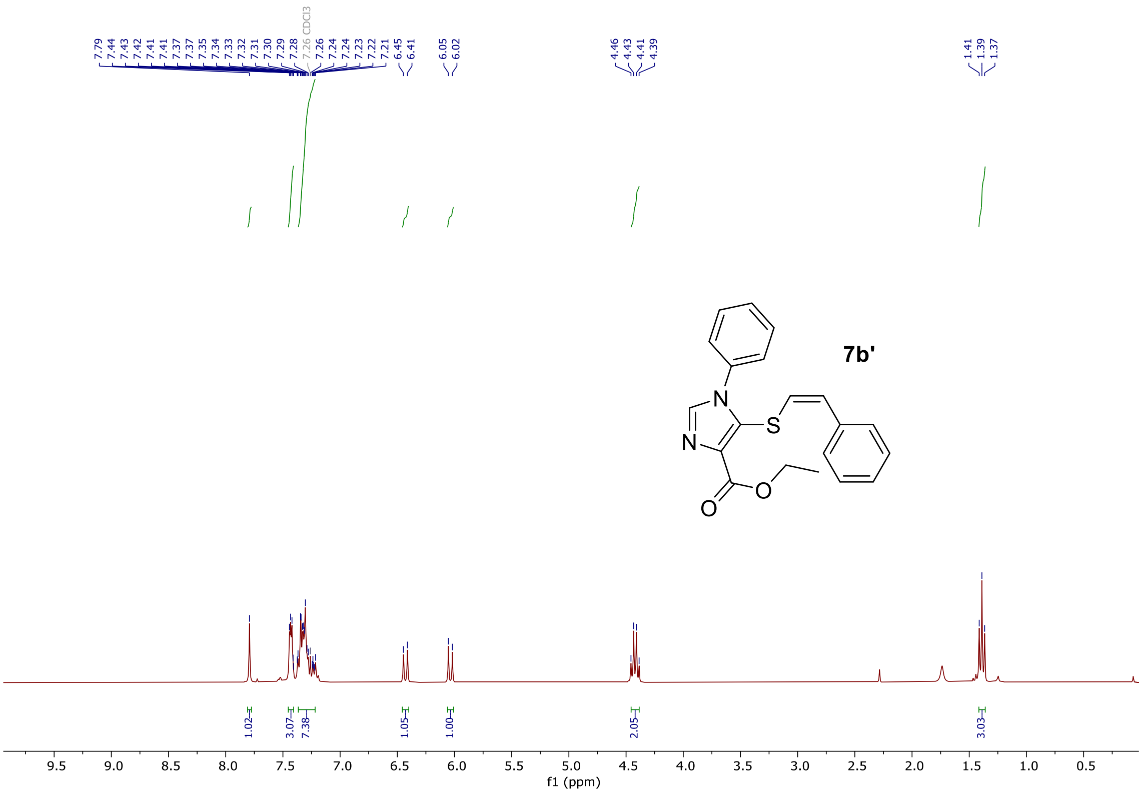

^{13}C{^{1}H} NMR (75 MHz, $CDCl_3$, 300K)

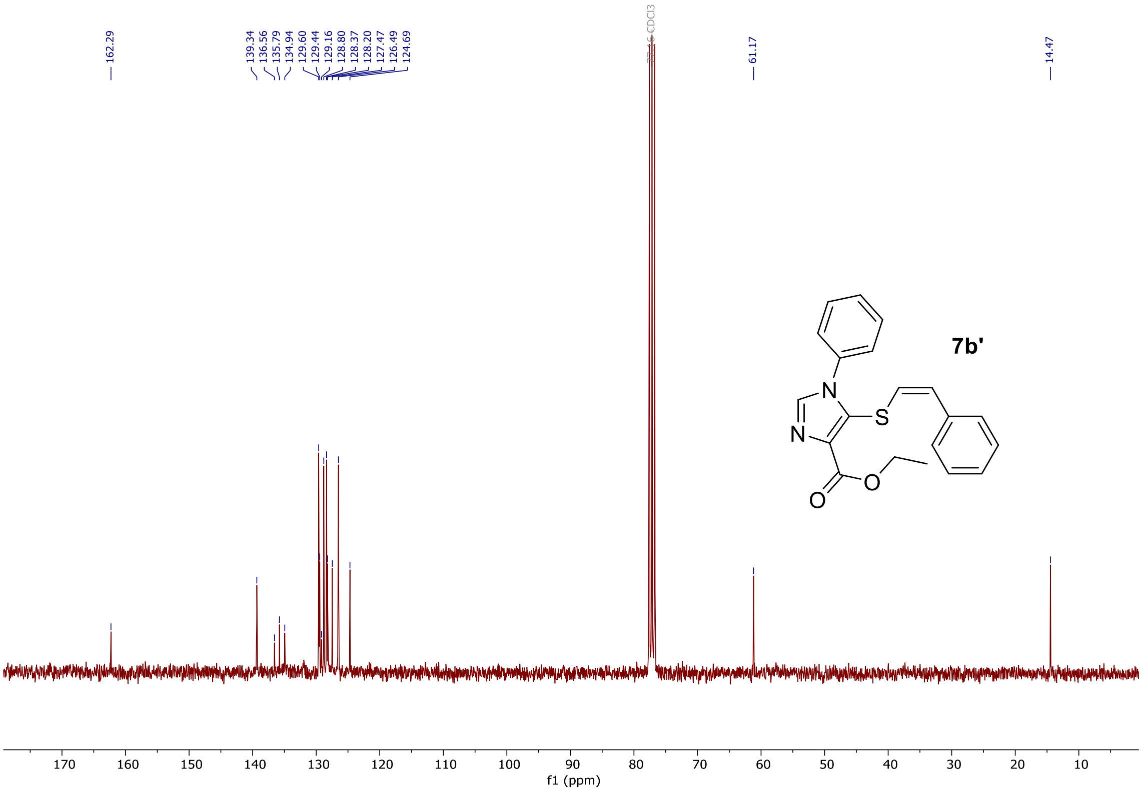

Ethyl 5-((4-methylstyryl)thio)-1-phenyl-1H-imidazole-4-carboxylate (7c)

^{1}H RMN (300 MHz, $CDCl_3$, 300K)

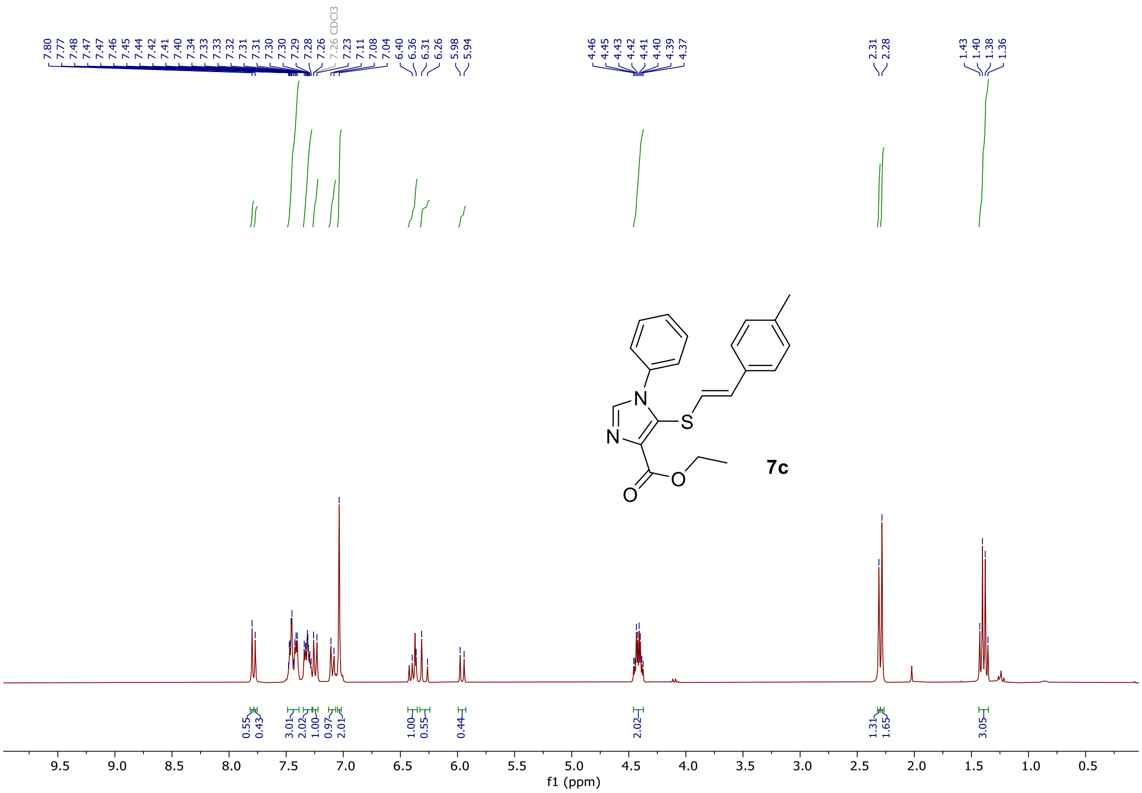

^{13}C{^{1}H} NMR (75 MHz, $CDCl_3$, 300K)

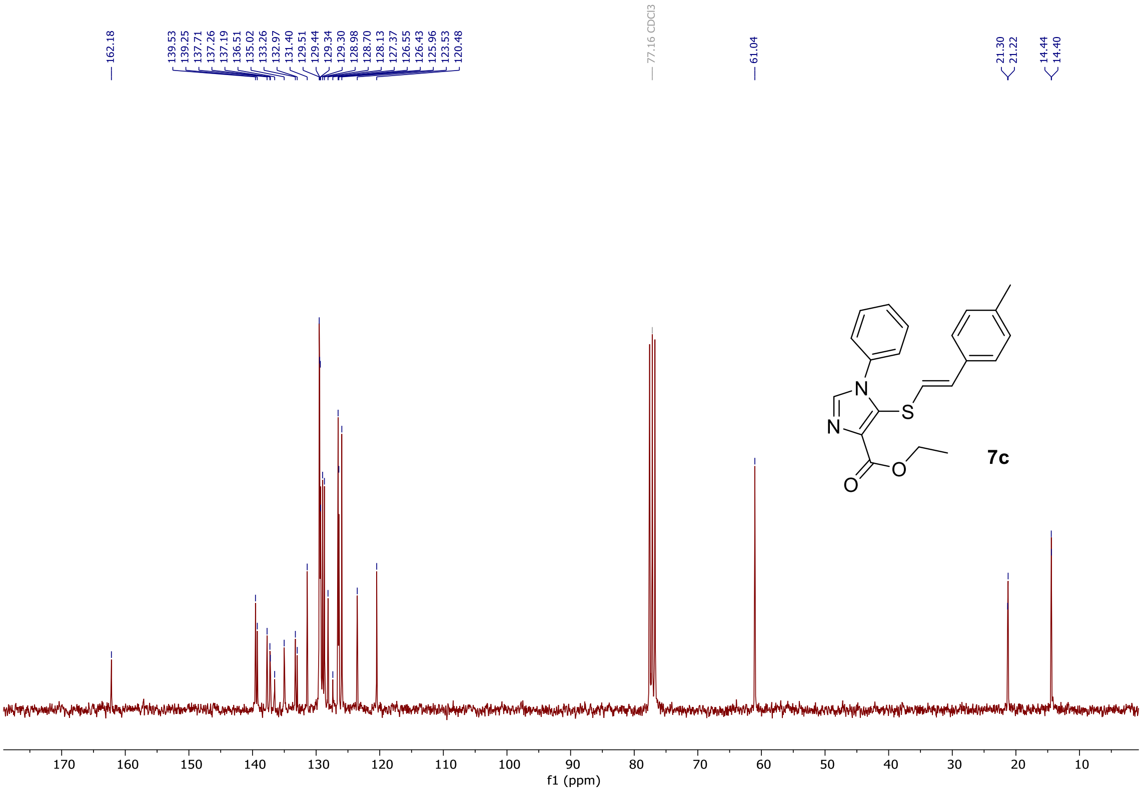

Ethyl 5-((4-cyanostyryl)thio)-1-phenyl-1H-imidazole-4-carboxylate (7d)

^{1}H RMN (300 MHz, $CDCl_3$, 300K)

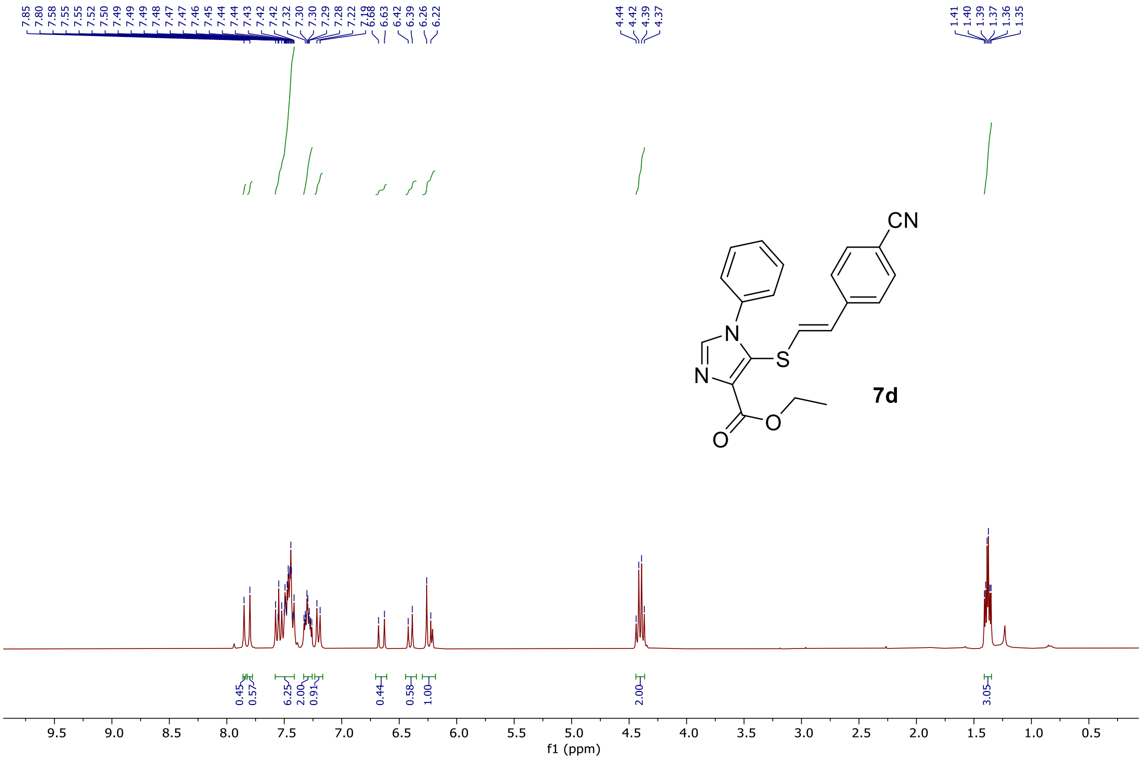

^{13}C{^{1}H} NMR (75 MHz, $CDCl_3$, 300K)

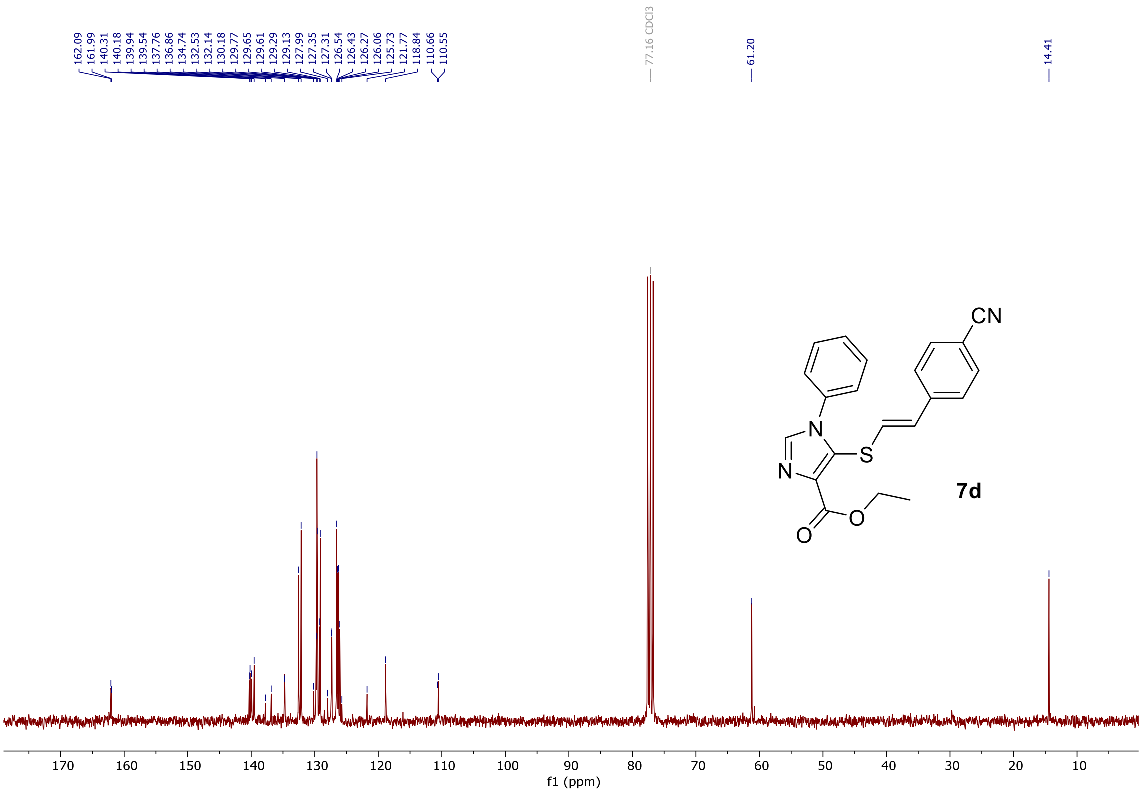

Ethyl 5-((2-chloro-6-fluorostyryl)thio)-1-phenyl-1H-imidazole-4-carboxylate (7e)

^{1}H RMN (300 MHz, $CDCl_3$, 300K)

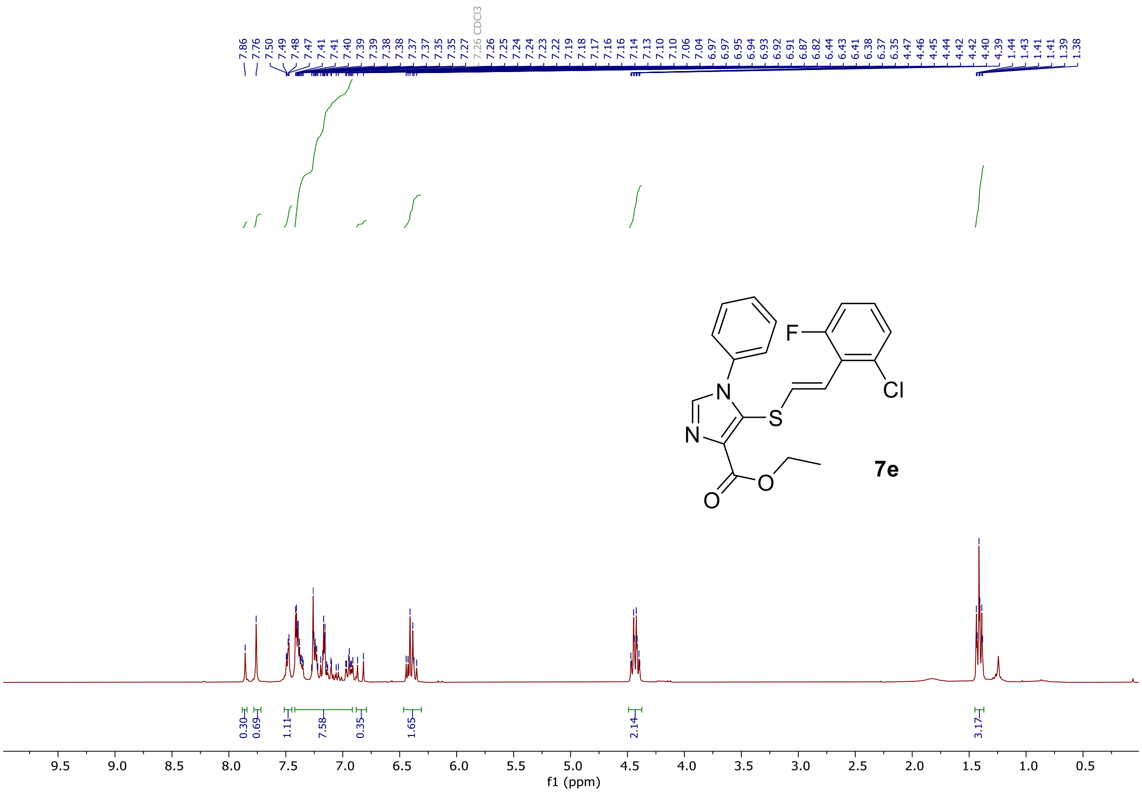

^{13}C{^{1}H} NMR (75 MHz, $CDCl_3$, 300K)

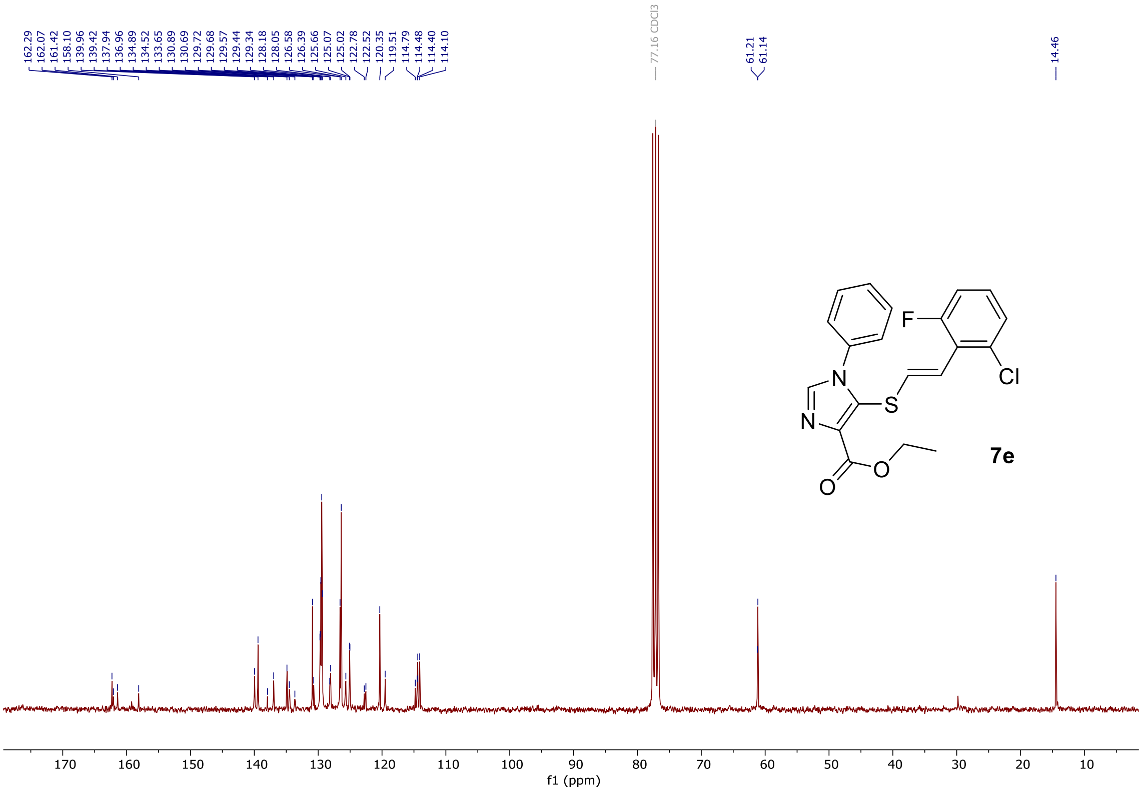

^{19}F RMN (282 MHz, $CDCl_3$, 300K)

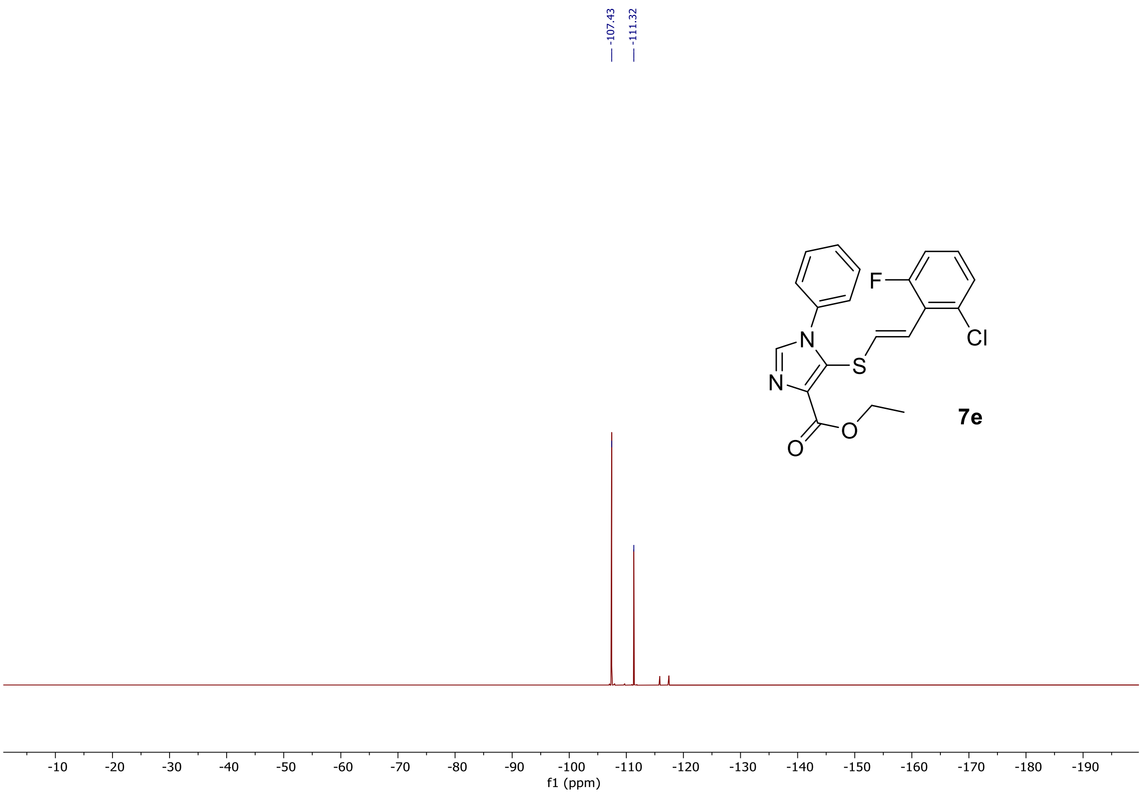

Ethyl 5-((4-nitrostyryl)thio)-1-phenyl-1H-imidazole-4-carboxylate (7f)

^{1}H RMN (300 MHz, $CDCl_3$, 300K)

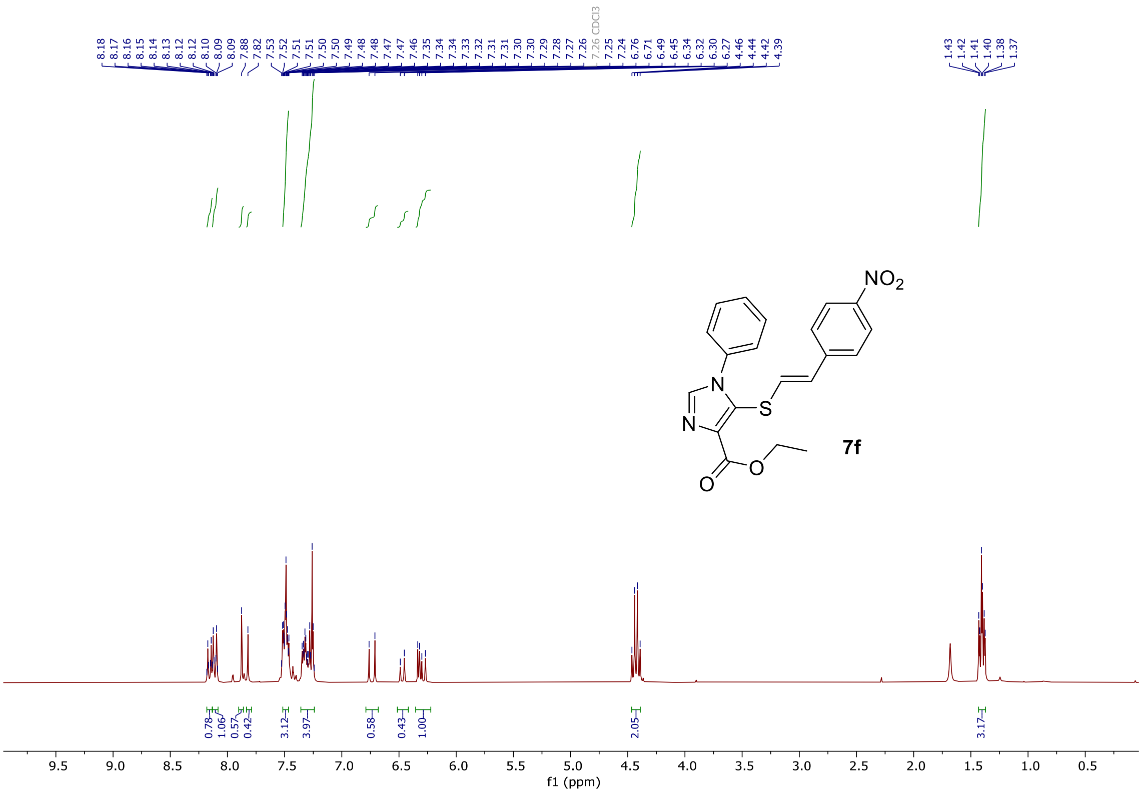

^{13}C{^{1}H} NMR (75 MHz, $CDCl_3$, 300K)

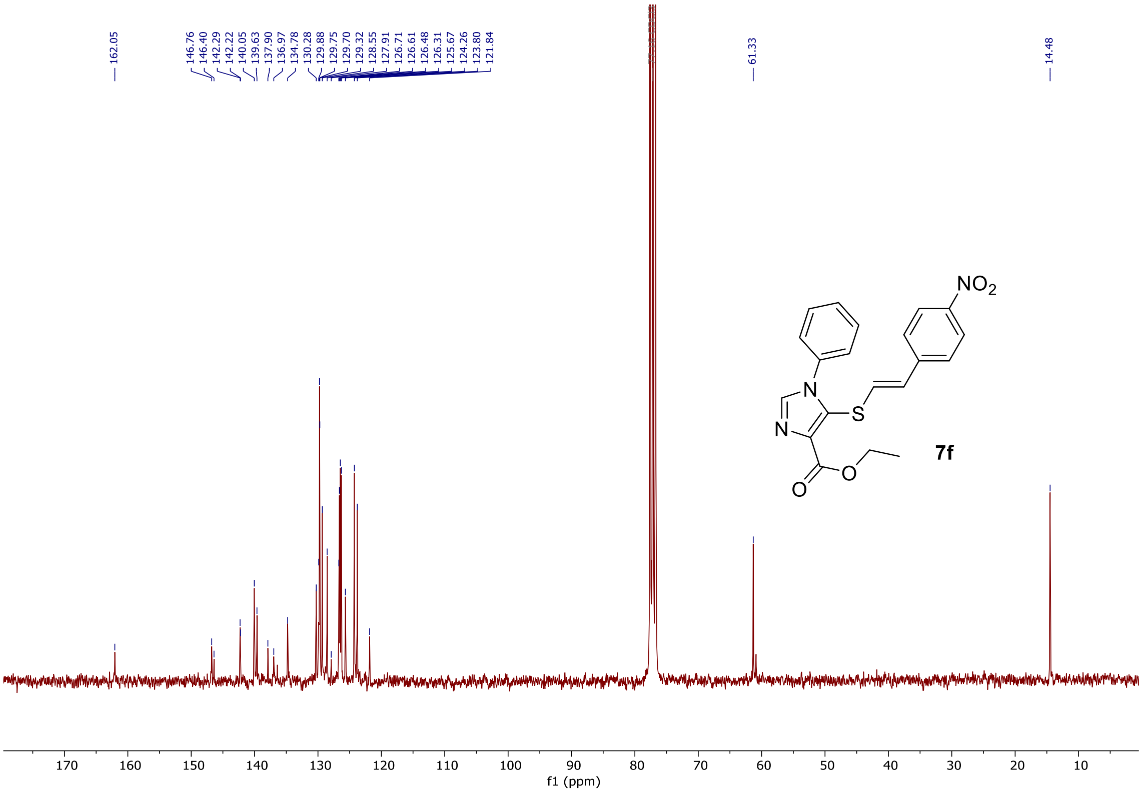

Ethyl 5-((4-(tert-butyl)styryl)thio)-1-phenyl-1H-imidazole-4-carboxylate (7g)

^{1}H RMN (300 MHz, $CDCl_3$, 300K)

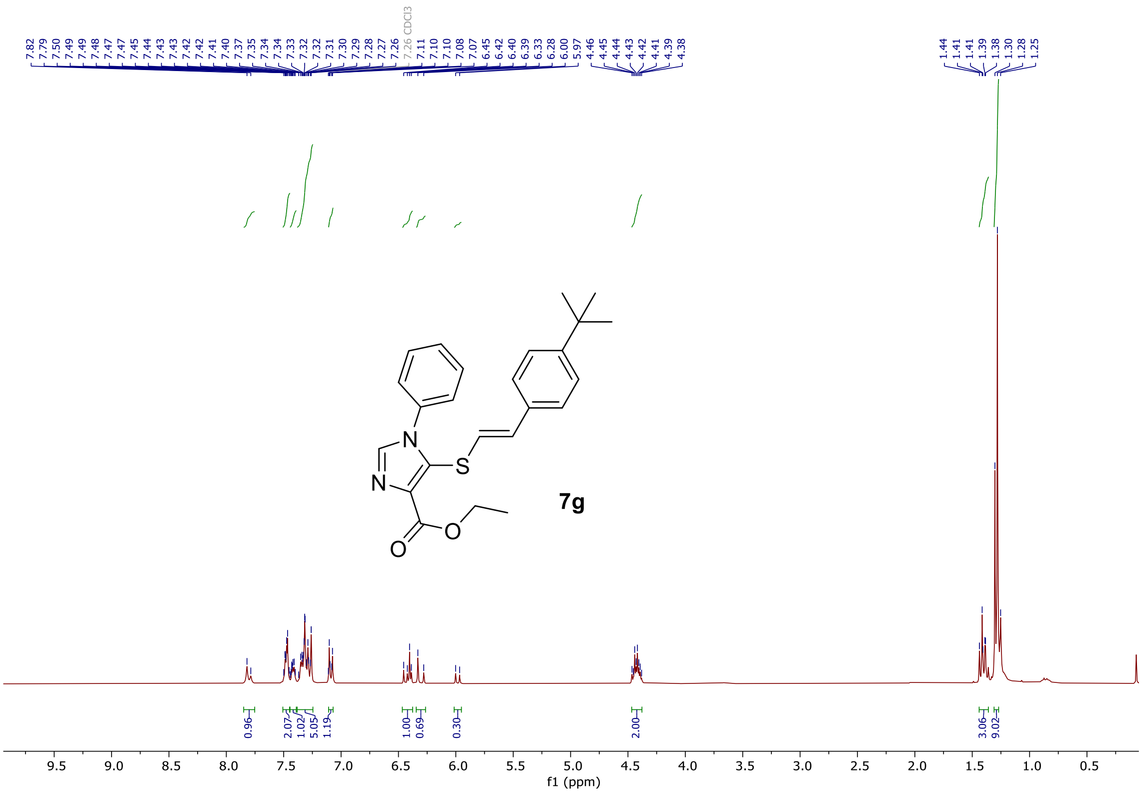

^{13}C{^{1}H} NMR (75 MHz, $CDCl_3$, 300K)

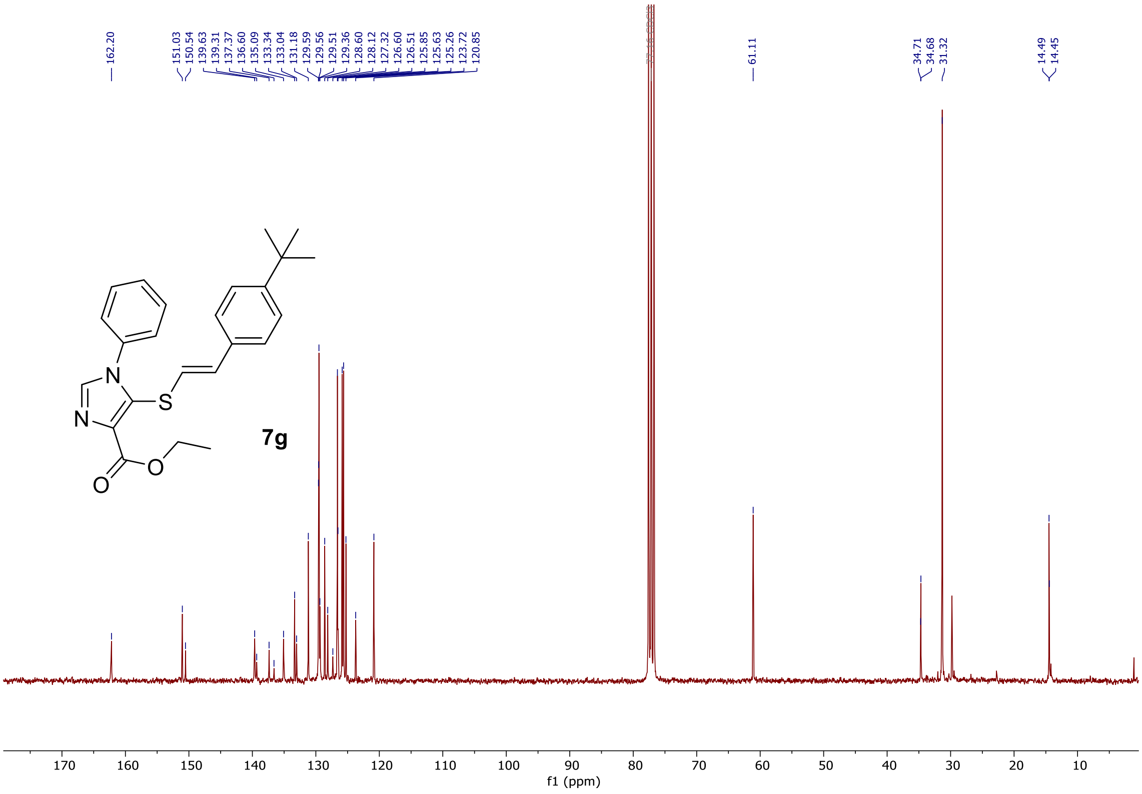

Ethyl 5-((4-(methylthio)styryl)thio)-1-phenyl-1H-imidazole-4-carboxylate (7h)

^{1}H RMN (300 MHz, $CDCl_3$, 300K)

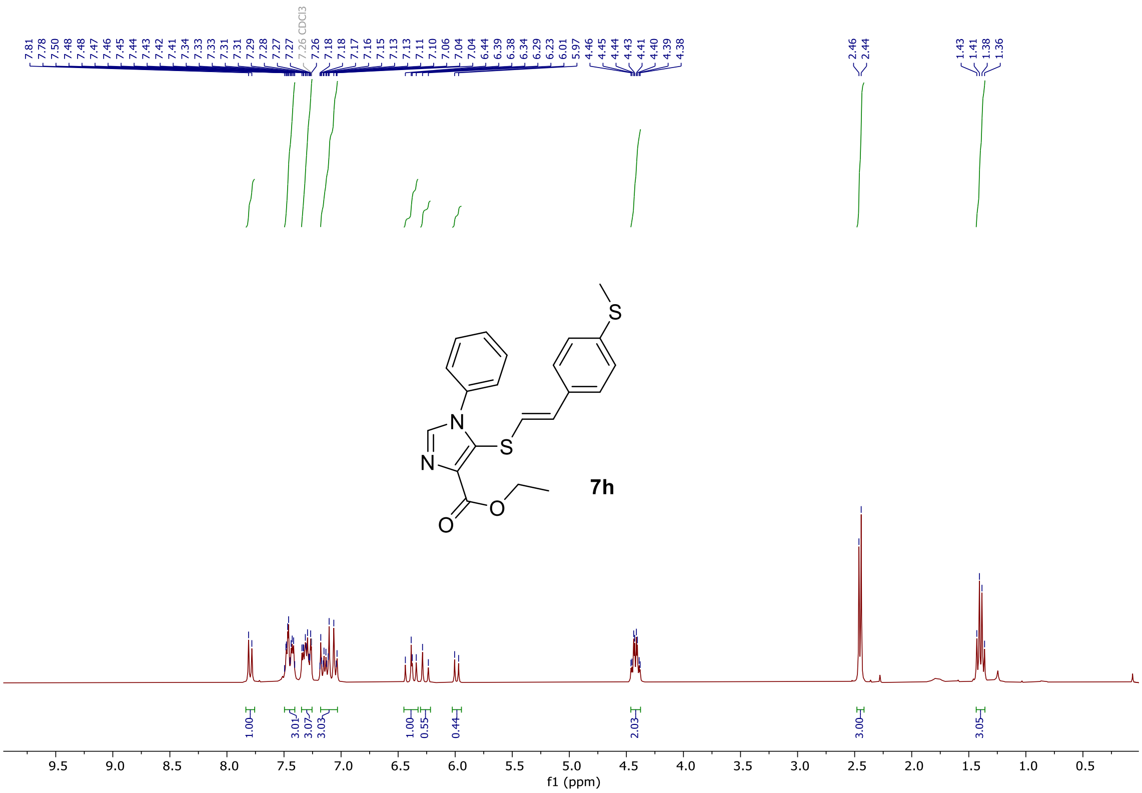

$^{13}C\{^{1}H\}$ NMR (75 MHz, $CDCl_3$, 300K)

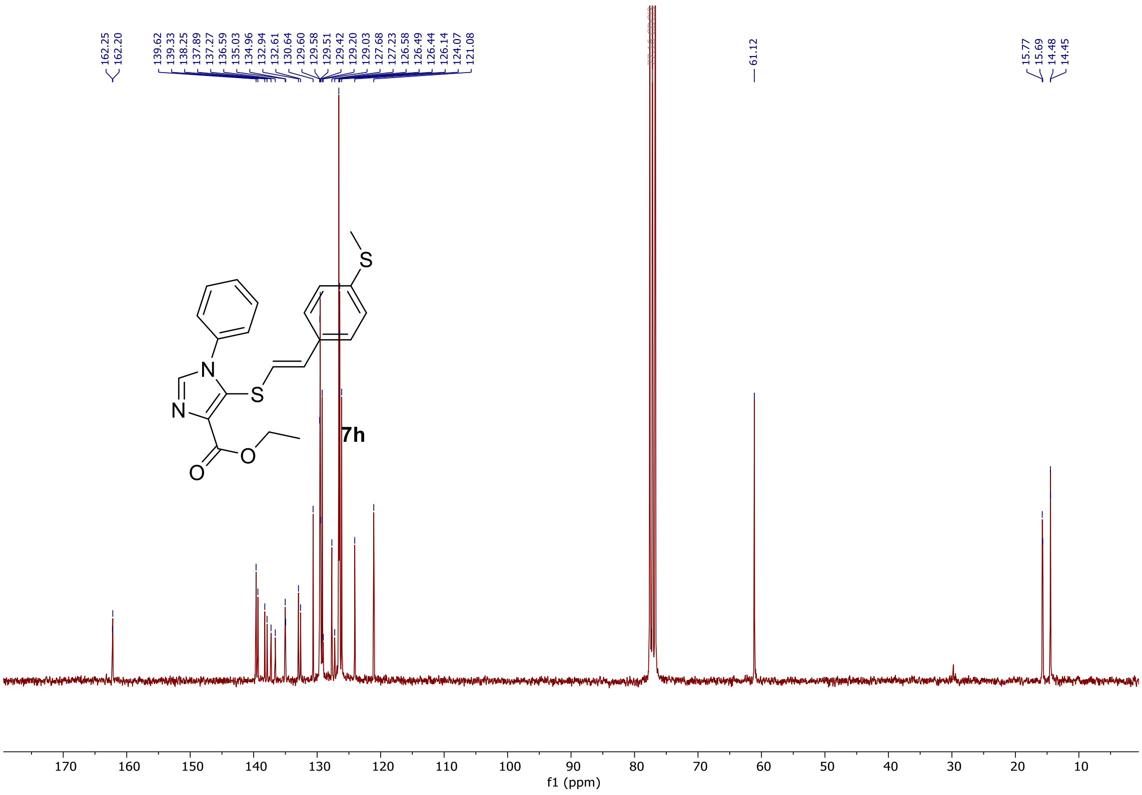

Ethyl 5-((4-chlorostyryl)thio)-1-phenyl-1H-imidazole-4-carboxylate (7i)

^{1}H RMN (300 MHz, $CDCl_3$, 300K)

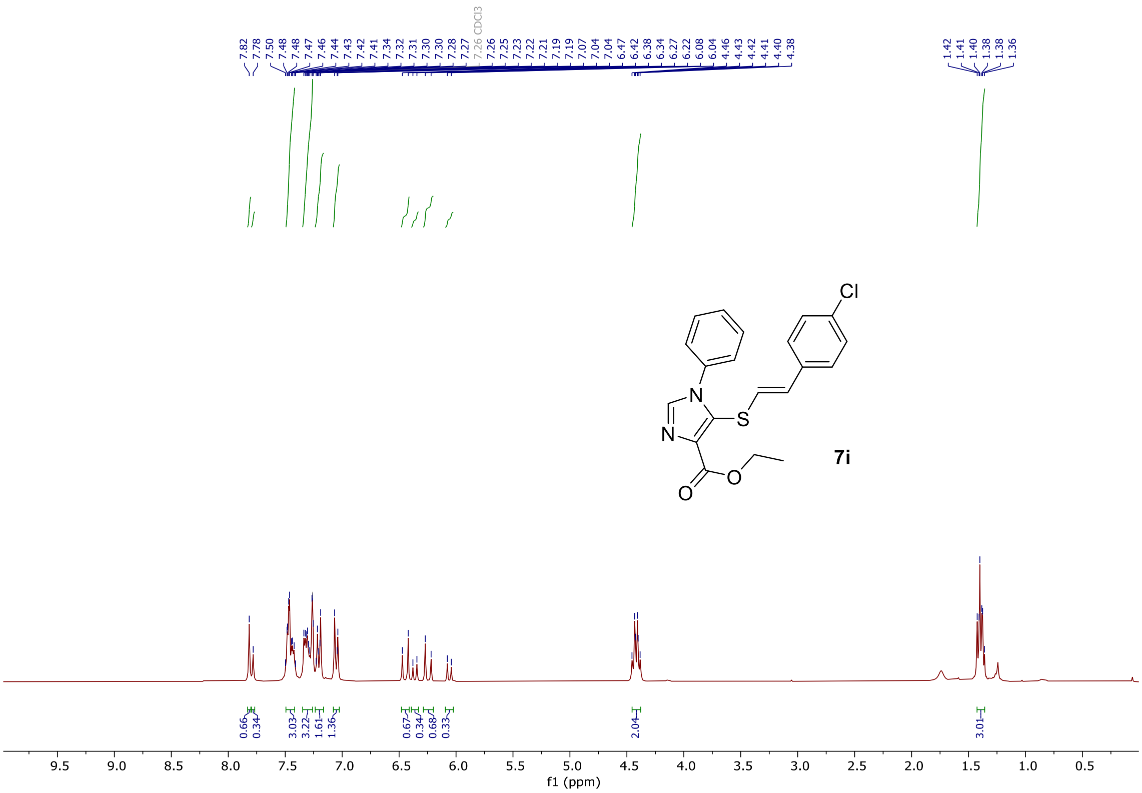

^{13}C{^{1}H} NMR (75 MHz, $CDCl_3$, 300K)

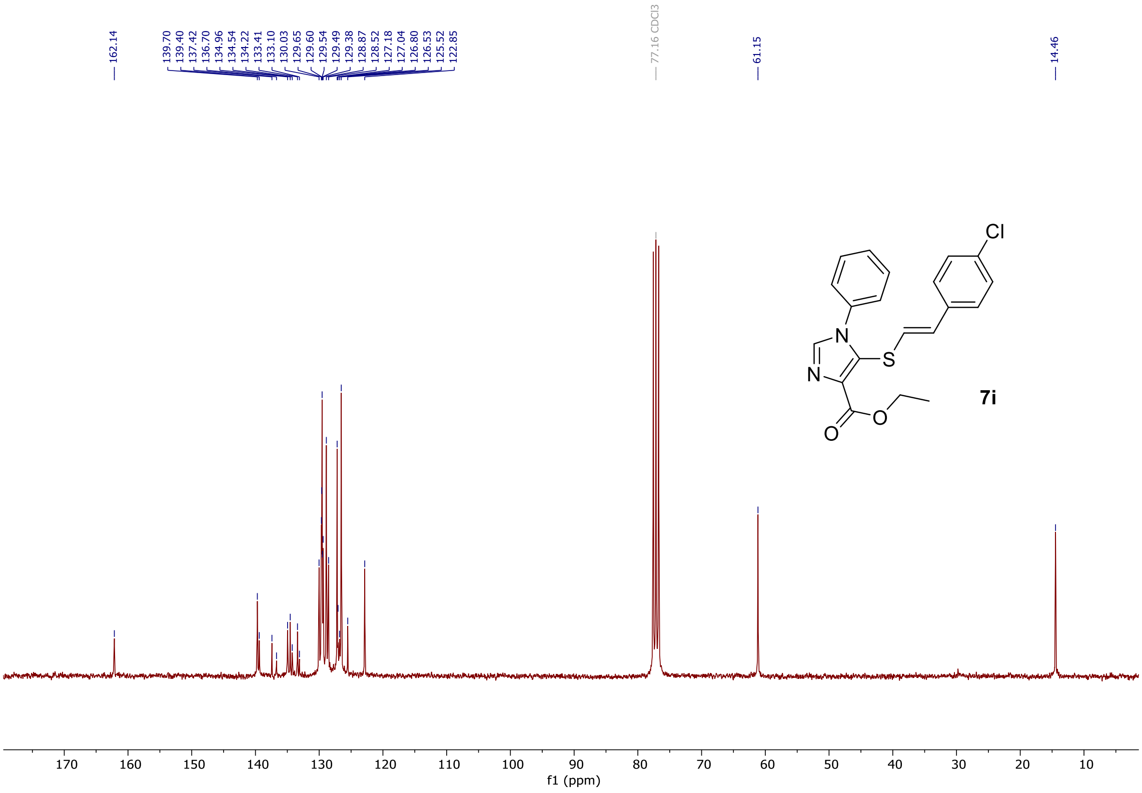

Ethyl 5-((2-chlorostyryl)thio)-1-phenyl-1H-imidazole-4-carboxylate (7j)

^{1}H RMN (300 MHz, $CDCl_3$, 300K)

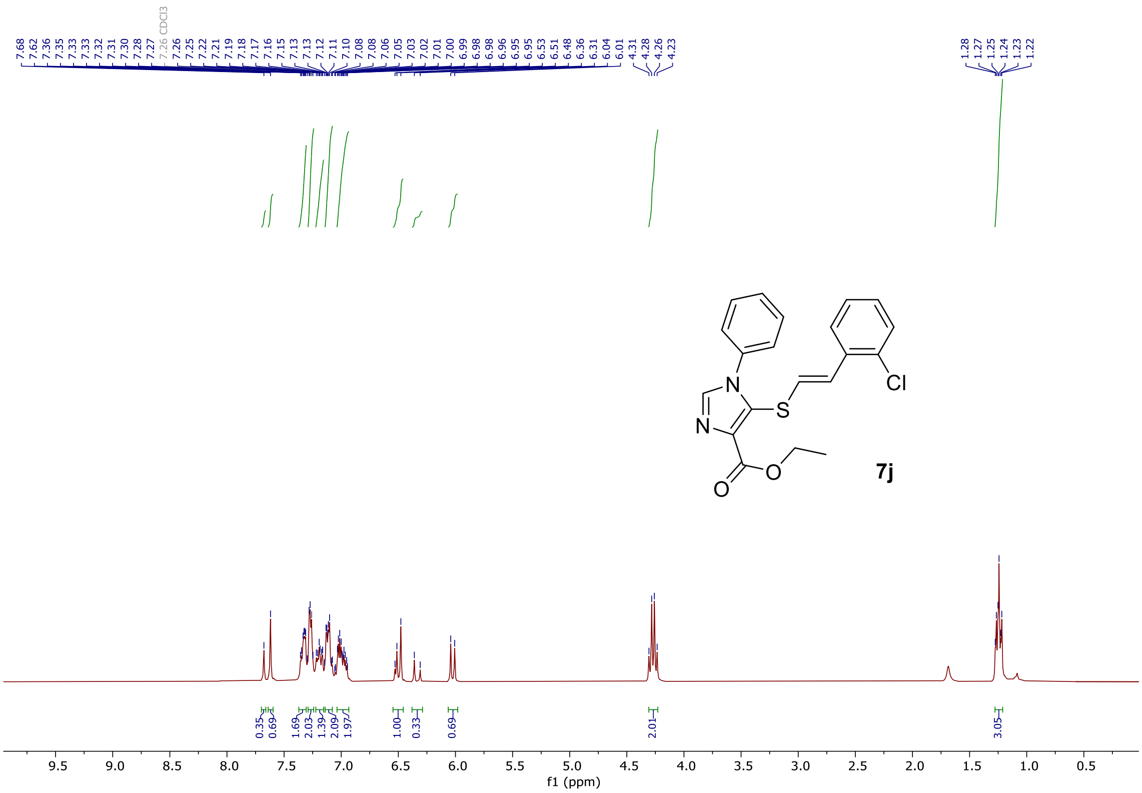

^{13}C{^{1}H} NMR (75 MHz, $CDCl_3$, 300K)

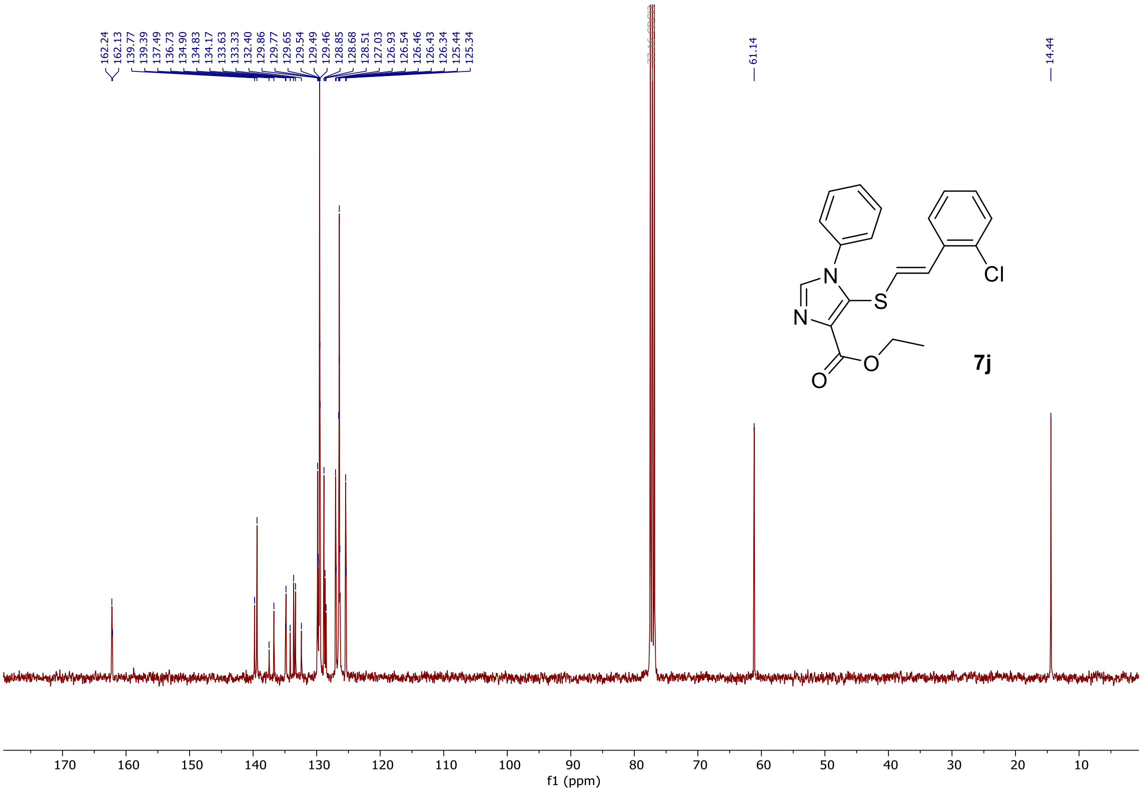

Ethyl 5-((3,5-dimethylstyryl)thio)-1-phenyl-1H-imidazole-4-carboxylate (7k)

^{1}H RMN (300 MHz, $CDCl_3$, 300K)

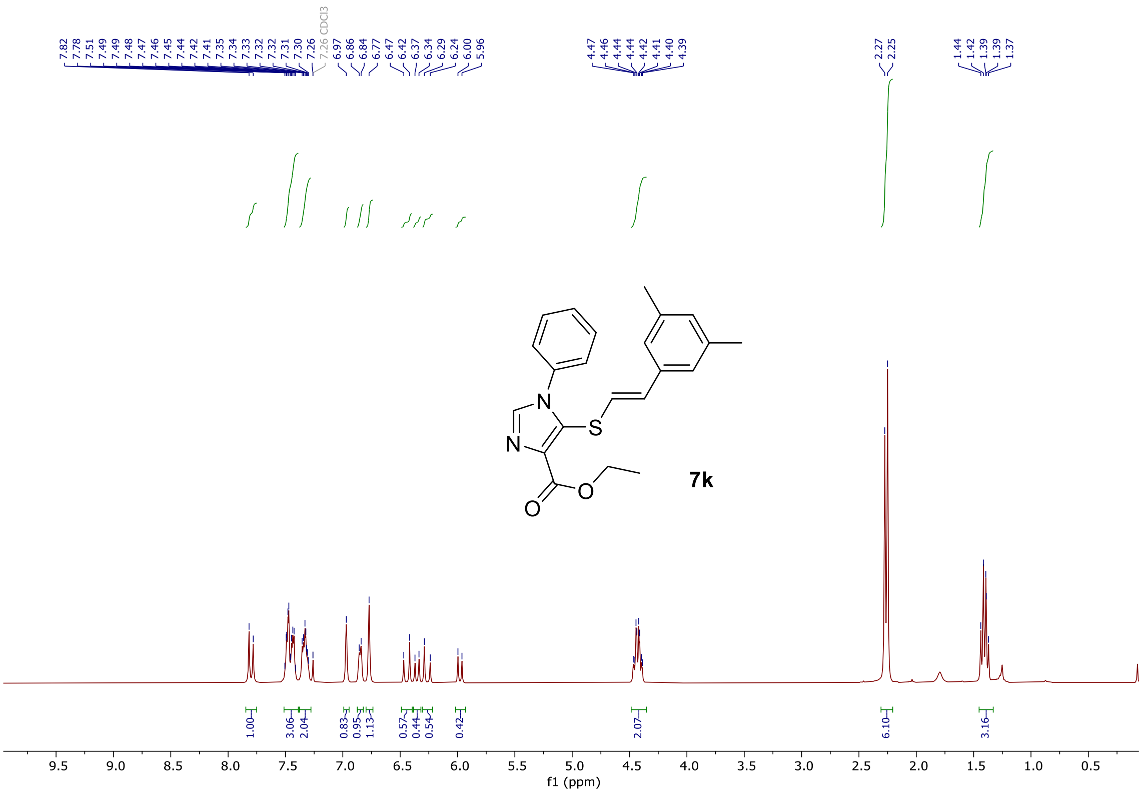

^{13}C{^{1}H} NMR (75 MHz, $CDCl_3$, 300K)

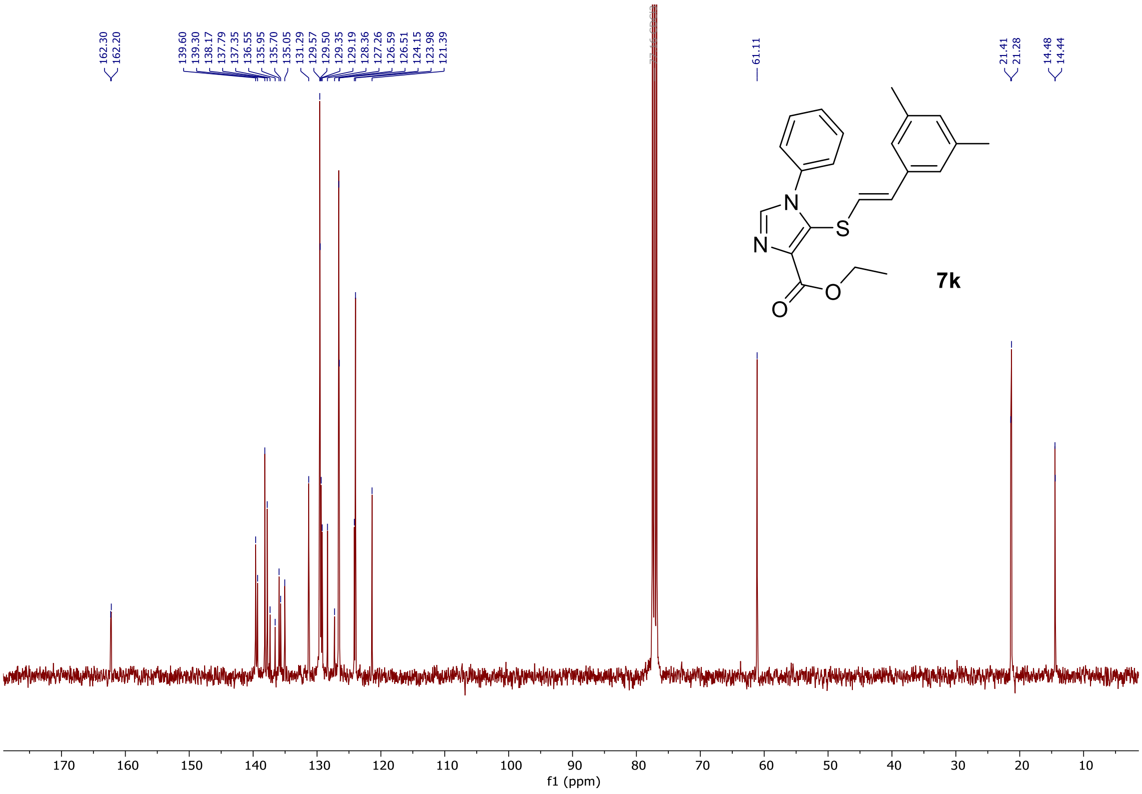

Ethyl 5-((2-methylstyryl)thio)-1-phenyl-1H-imidazole-4-carboxylate (7l)

^{1}H RMN (300 MHz, $CDCl_3$, 300K)

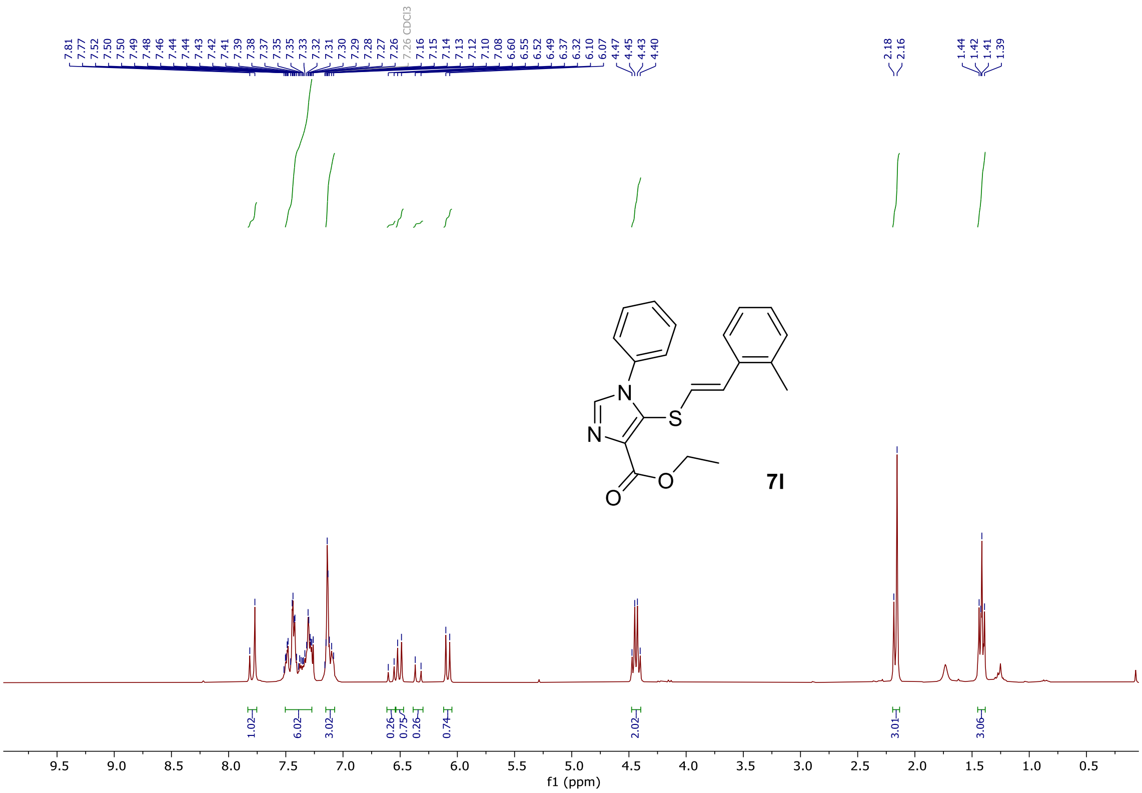

^{13}C{^{1}H} NMR (75 MHz, $CDCl_3$, 300K)

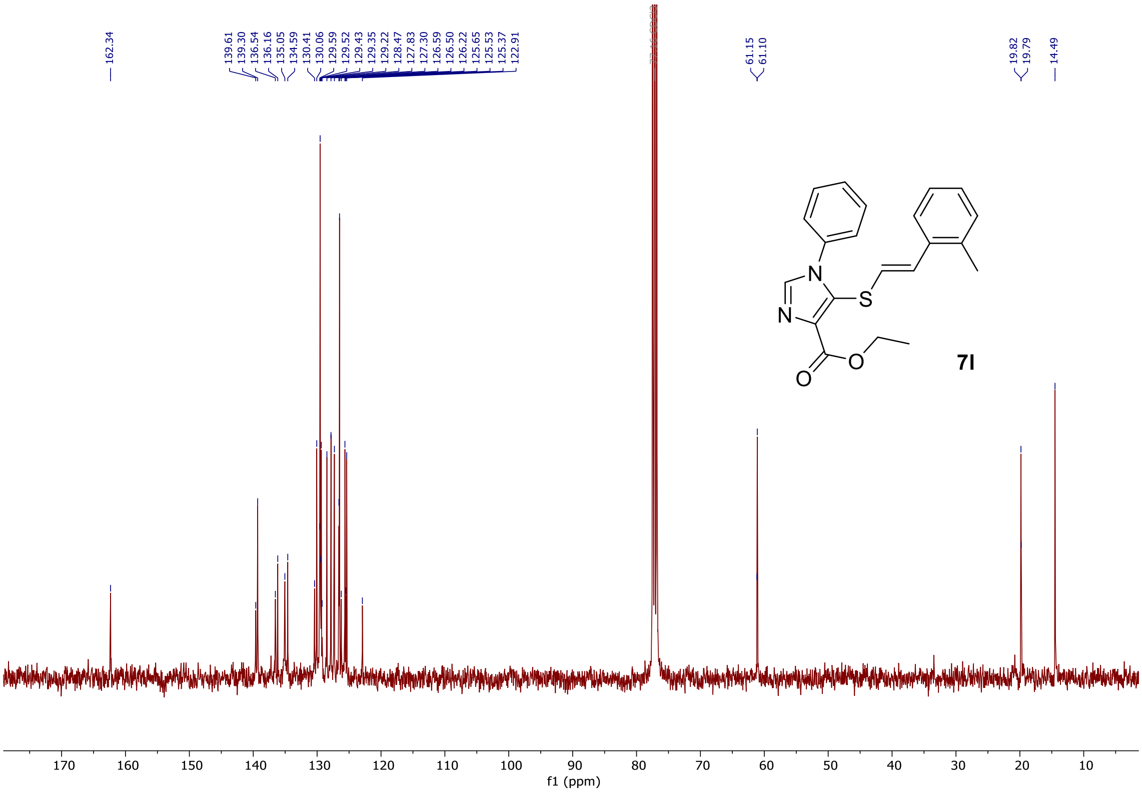

Ethyl 5-((3-bromostyryl)thio)-1-phenyl-1H-imidazole-4-carboxylate (7m)

^{1}H RMN (300 MHz, $CDCl_3$, 300K)

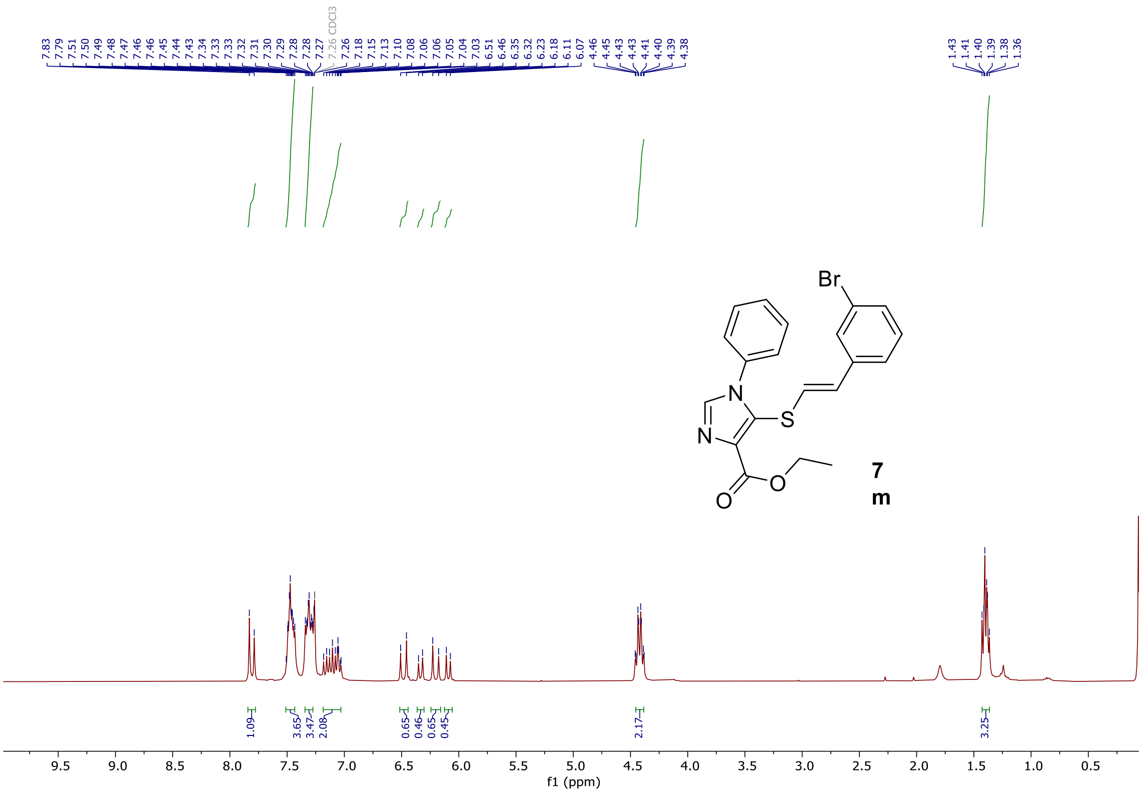

^{13}C{^{1}H} NMR (75 MHz, $CDCl_3$, 300K)

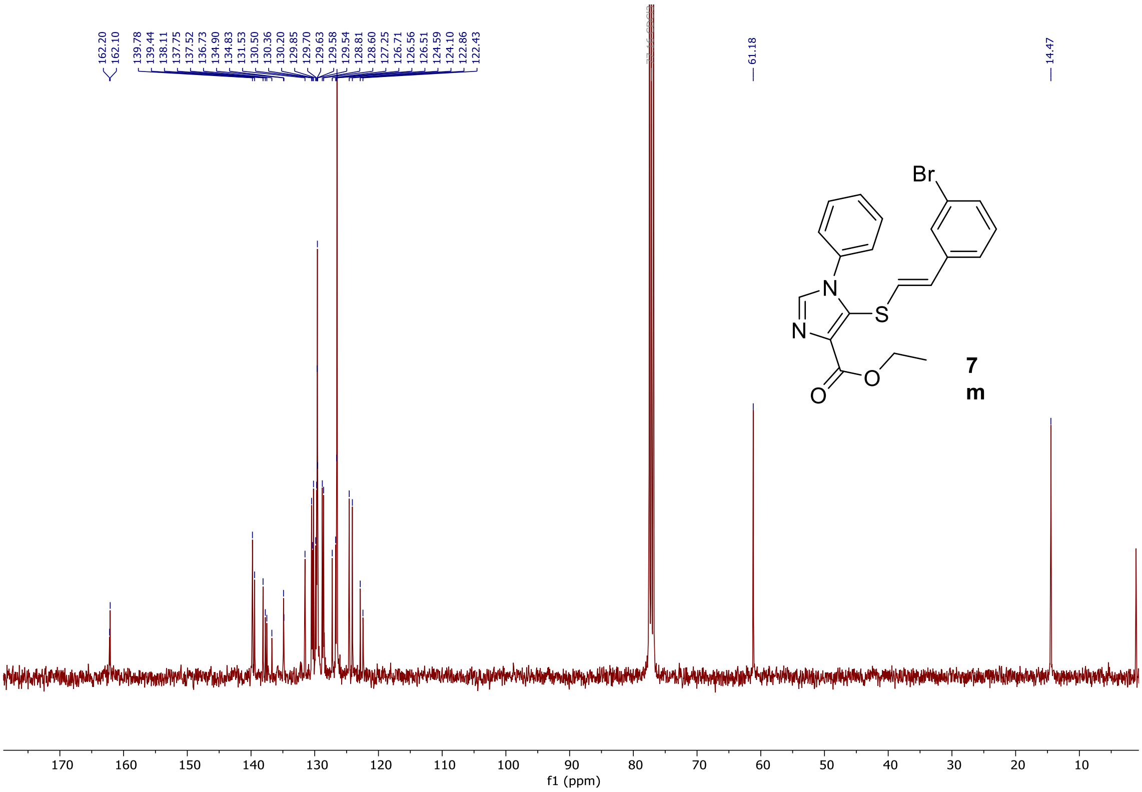

Ethyl 5-((3,5-dibromostyryl)thio)-1-phenyl-1H-imidazole-4-carboxylate (7n)

^{1}H RMN (300 MHz, $CDCl_3$, 300K)

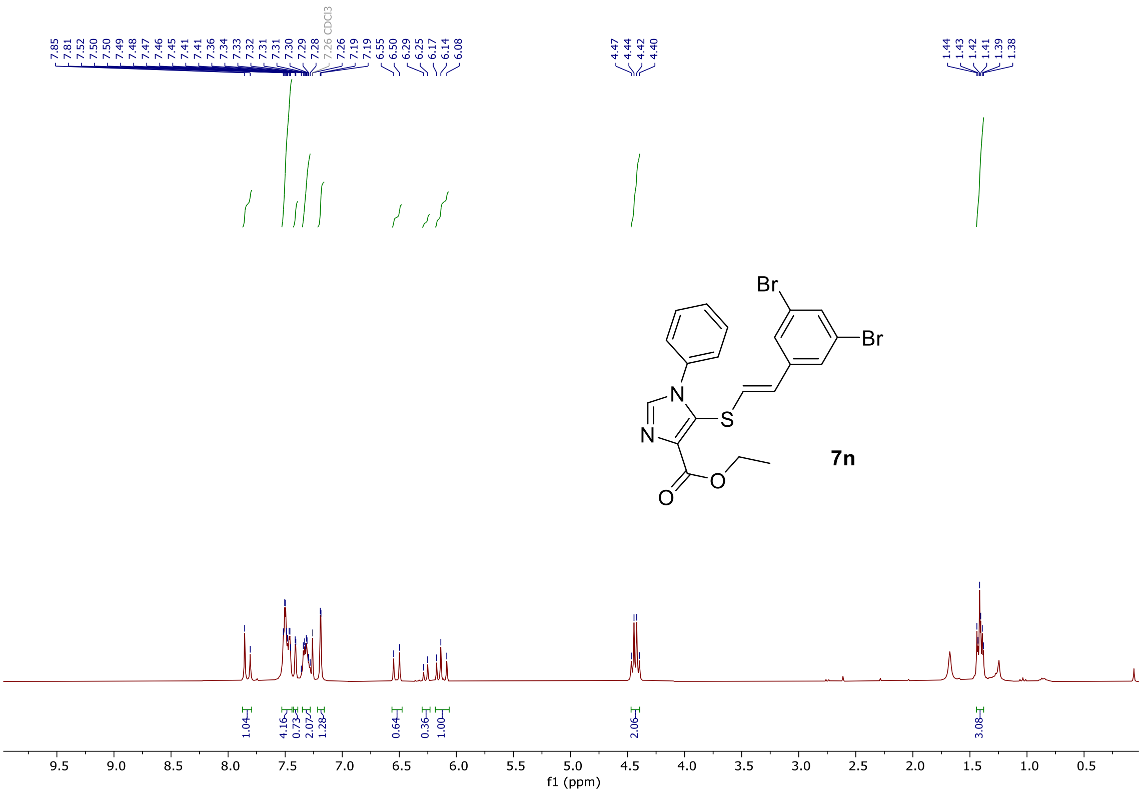

^{13}C{^{1}H} NMR (75 MHz, $CDCl_3$, 300K)

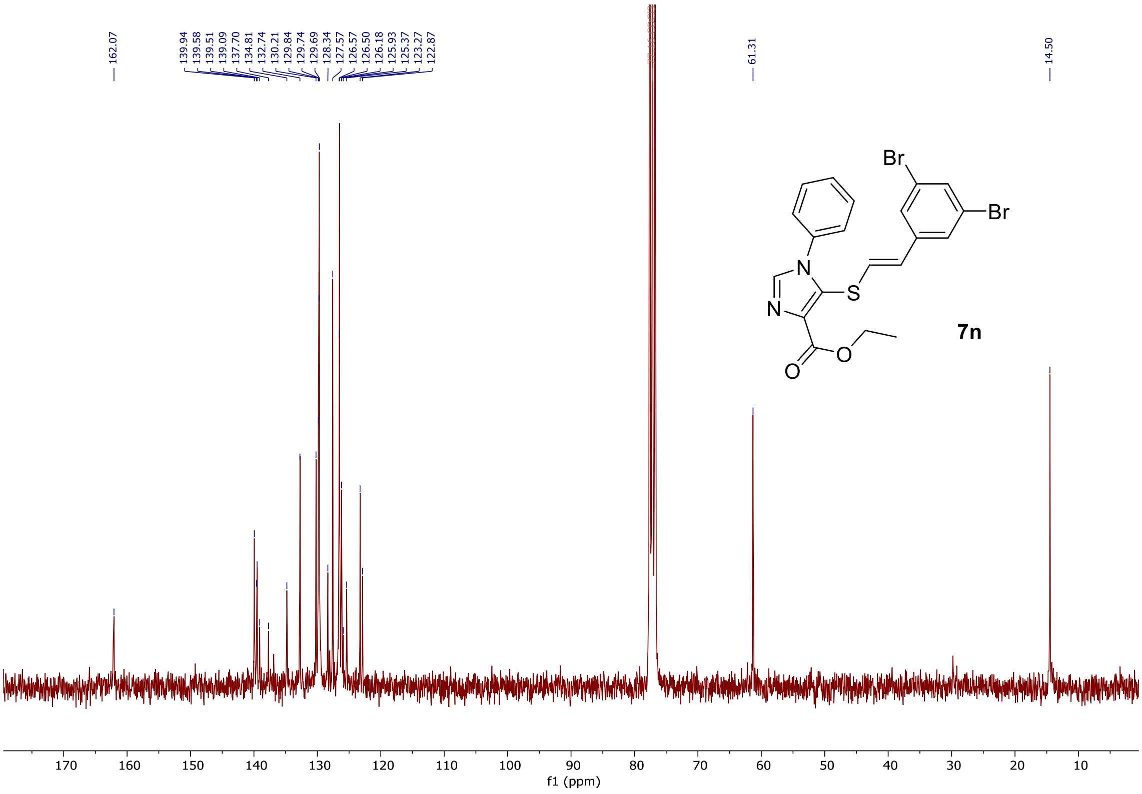

Ethyl 5-((2-(naphthalen-2-yl)vinyl)thio)-1-phenyl-1H-imidazole-4-carboxylate (7o)

^{1}H RMN (300 MHz, $CDCl_3$, 300K)

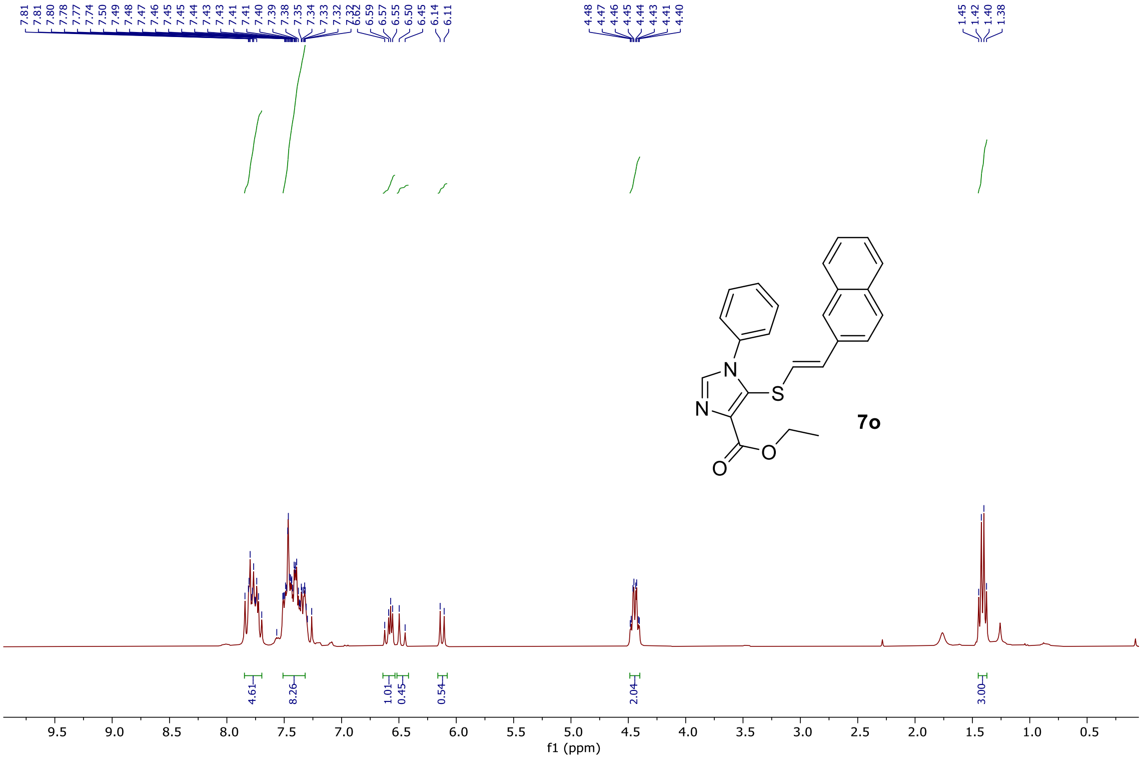

^{13}C{^{1}H} NMR (75 MHz, $CDCl_3$, 300K)

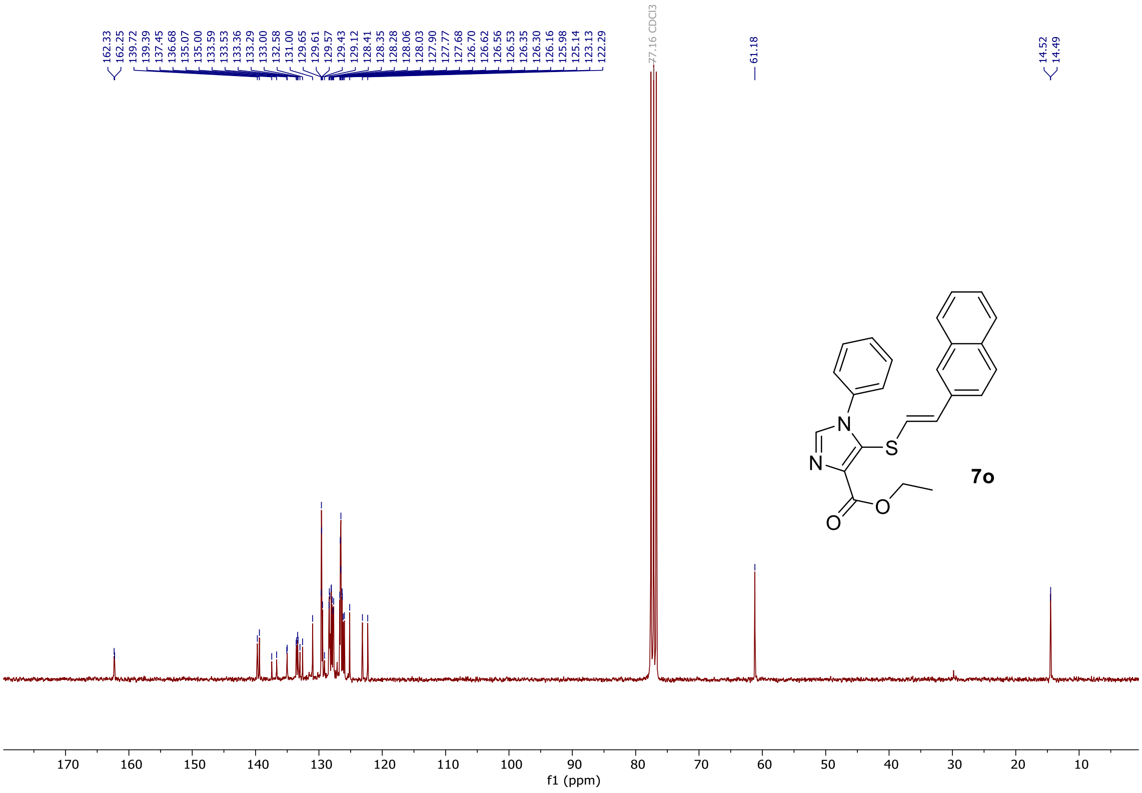

Ethyl 1-phenyl-5-((2-(thiophen-2-yl)vinyl)thio)-1H-imidazole-4-carboxylate (7p)

^{1}H RMN (300 MHz, $CDCl_3$, 300K)

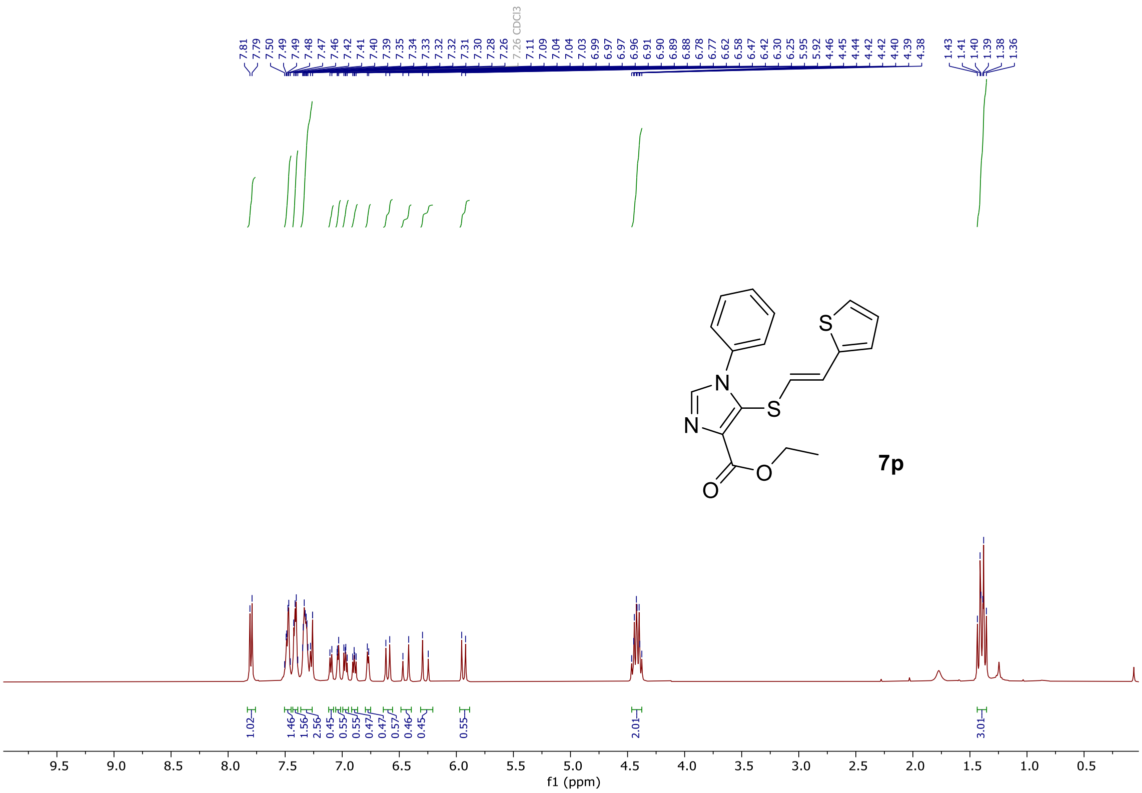

^{13}C{^{1}H} NMR (75 MHz, $CDCl_3$, 300K)

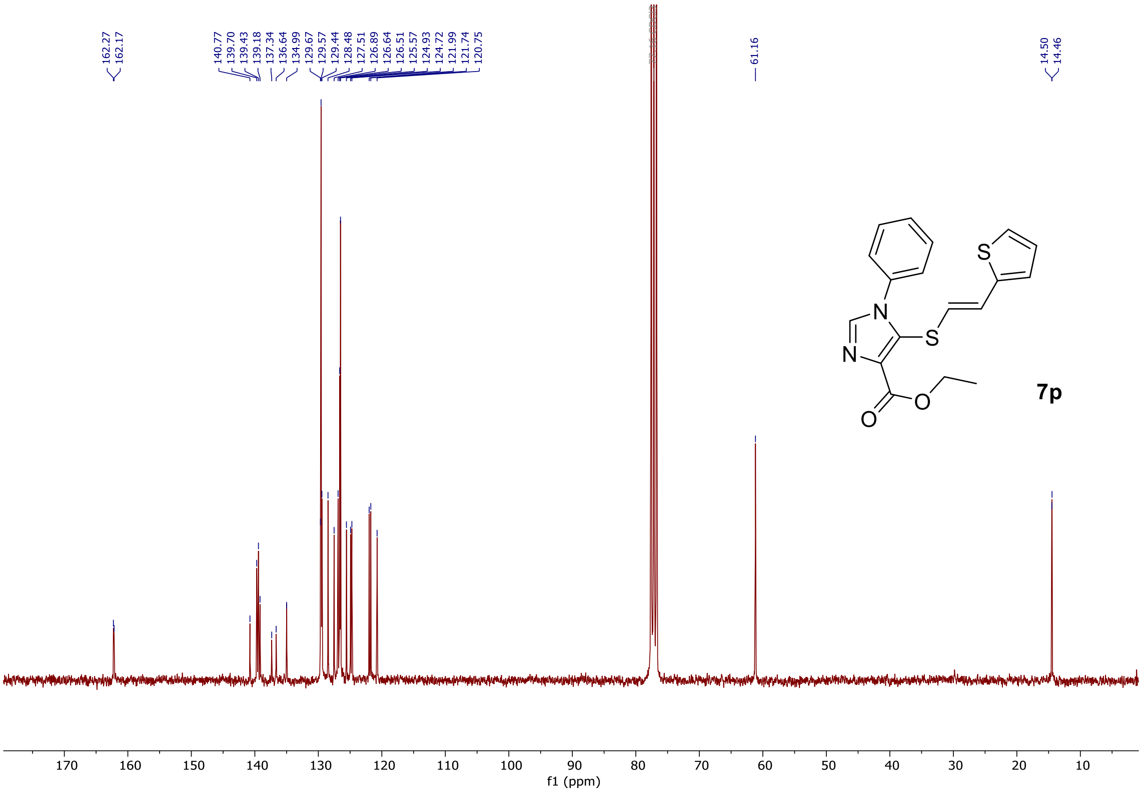

Ethyl 5-((2-(benzofuran-2-yl)vinyl)thio)-1-phenyl-1H-imidazole-4-carboxylate (7q)

^{1}H RMN (300 MHz, $CDCl_3$, 300K)

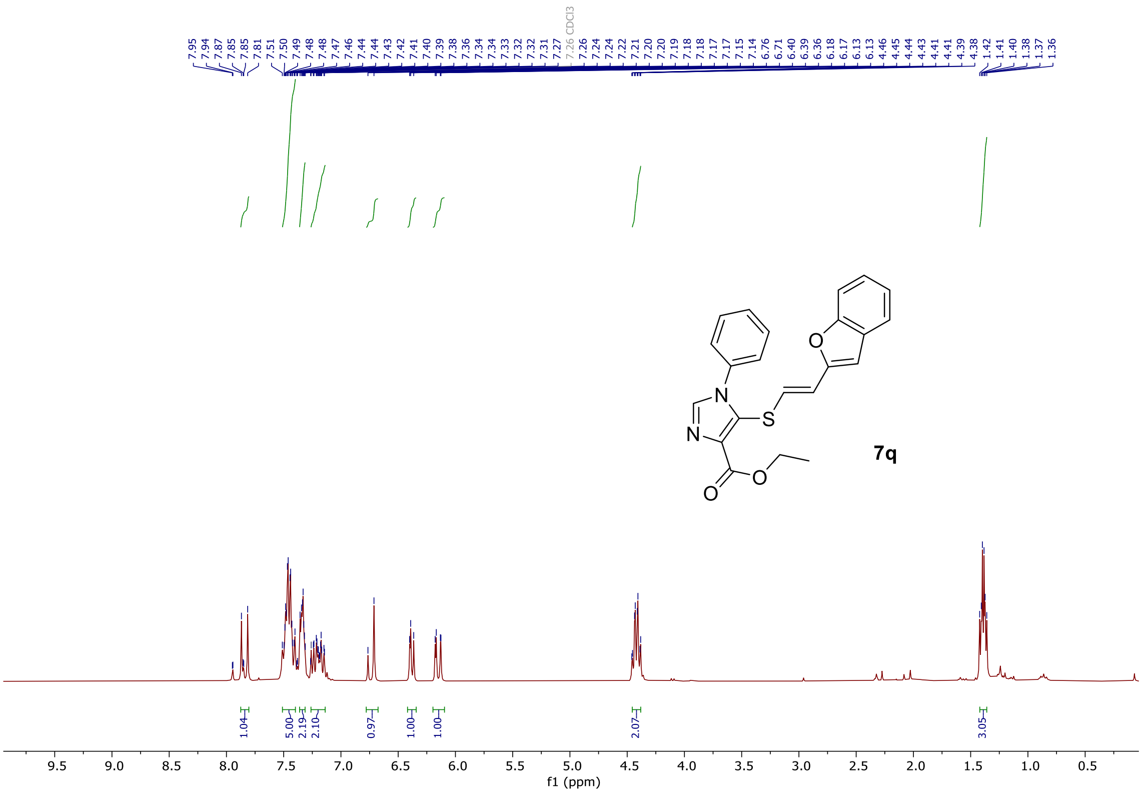

^{13}C{^{1}H} NMR (75 MHz, $CDCl_3$, 300K)

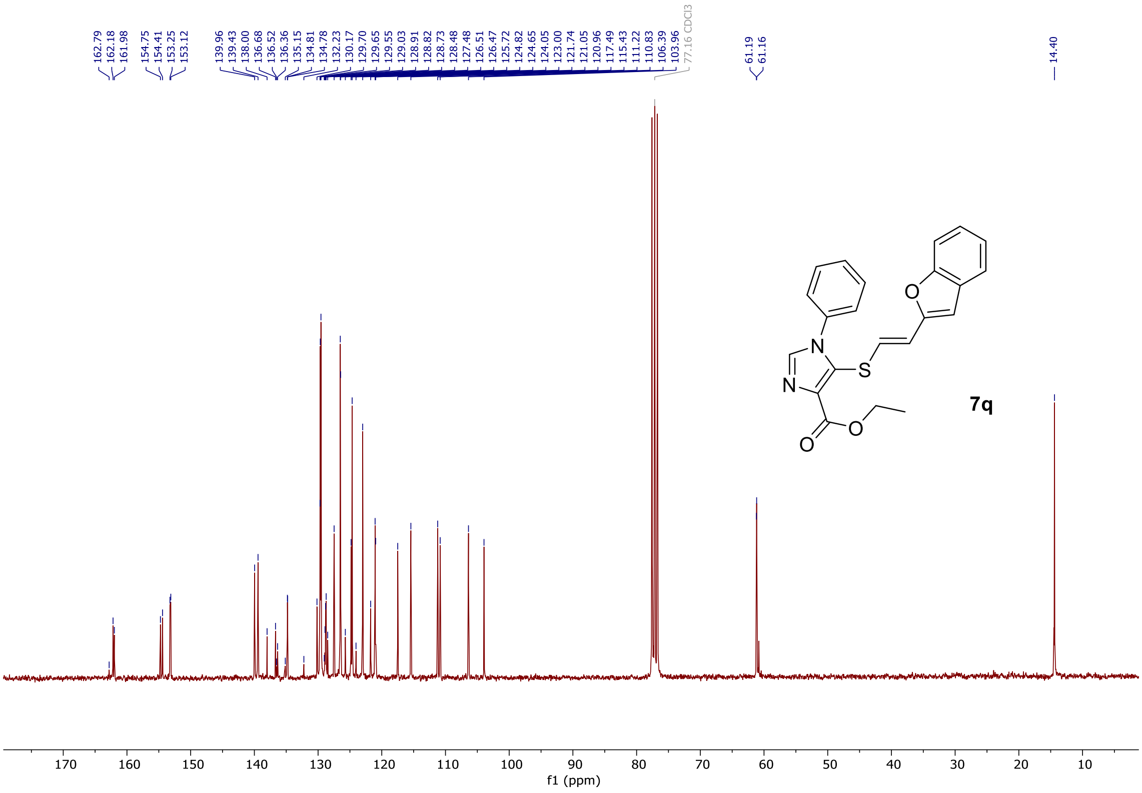

Ethyl 1-(5-fluoro-2-methylphenyl)-5-(styrylthio)-1H-imidazole-4-carboxylate (7r)

^{1}H RMN (300 MHz, $CDCl_3$, 300K)

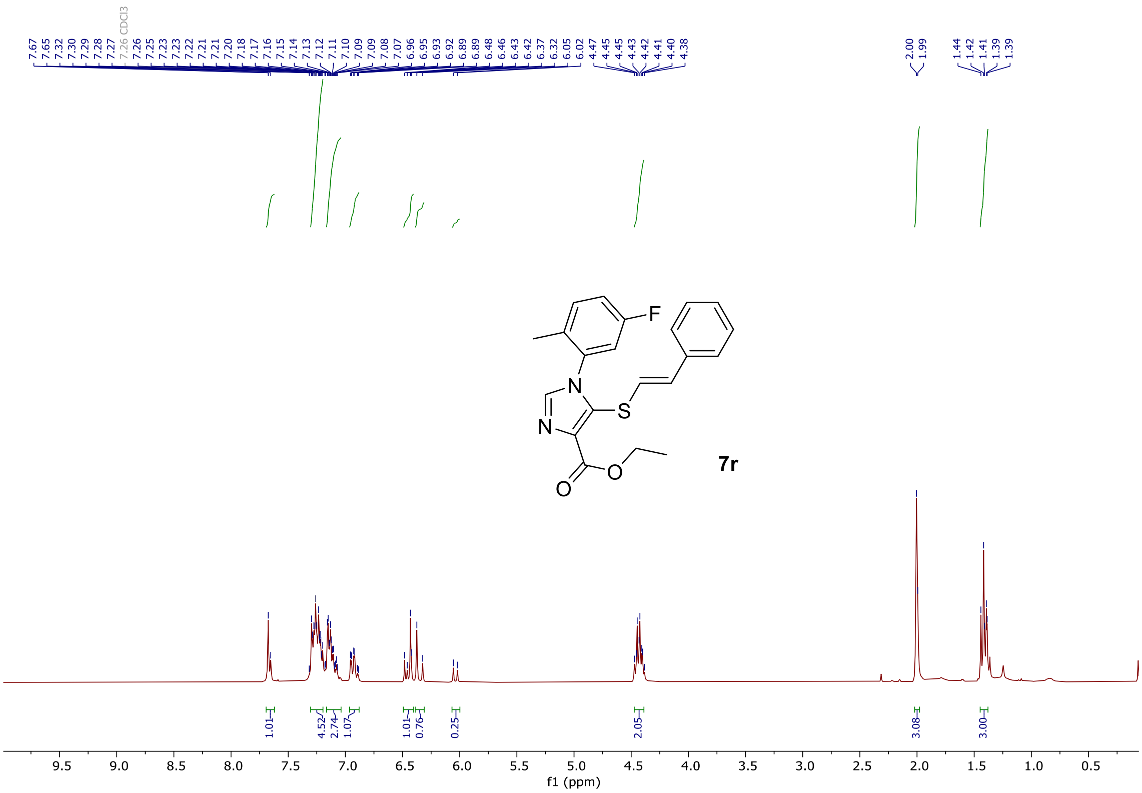

^{13}C{^{1}H} NMR (75 MHz, $CDCl_3$, 300K)

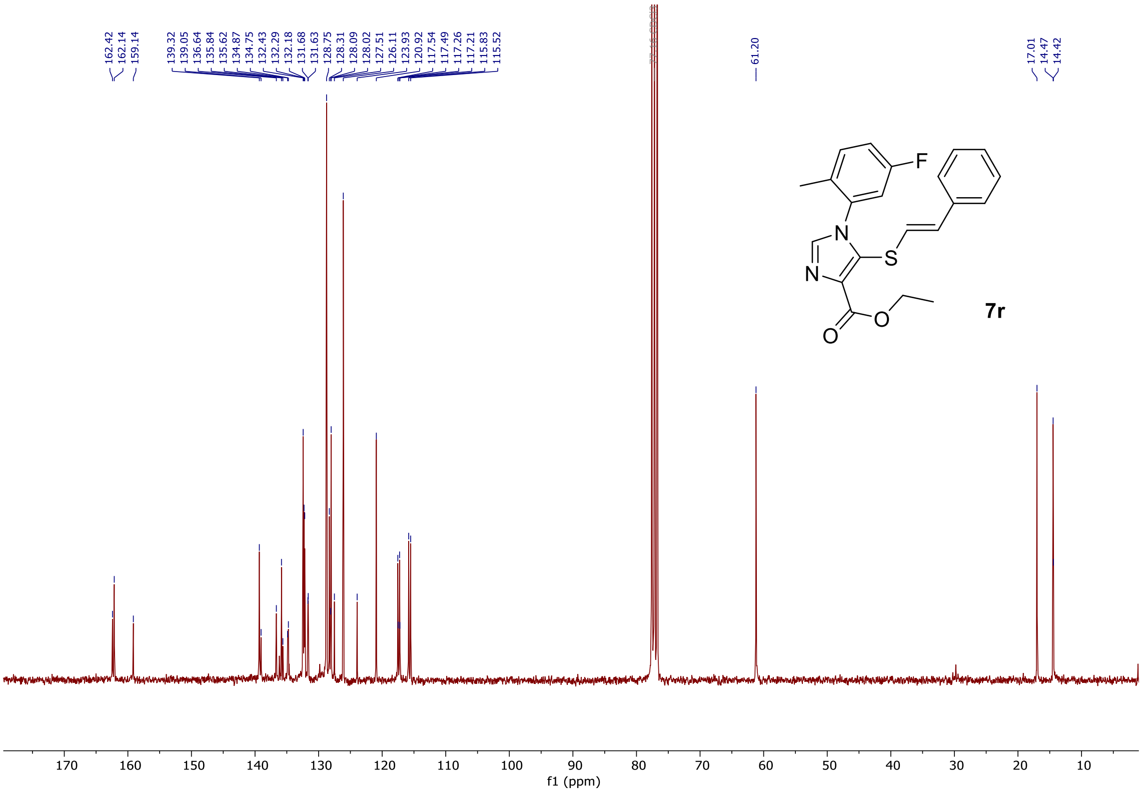

^{19}F RMN (282 MHz, $CDCl_3$, 300K)

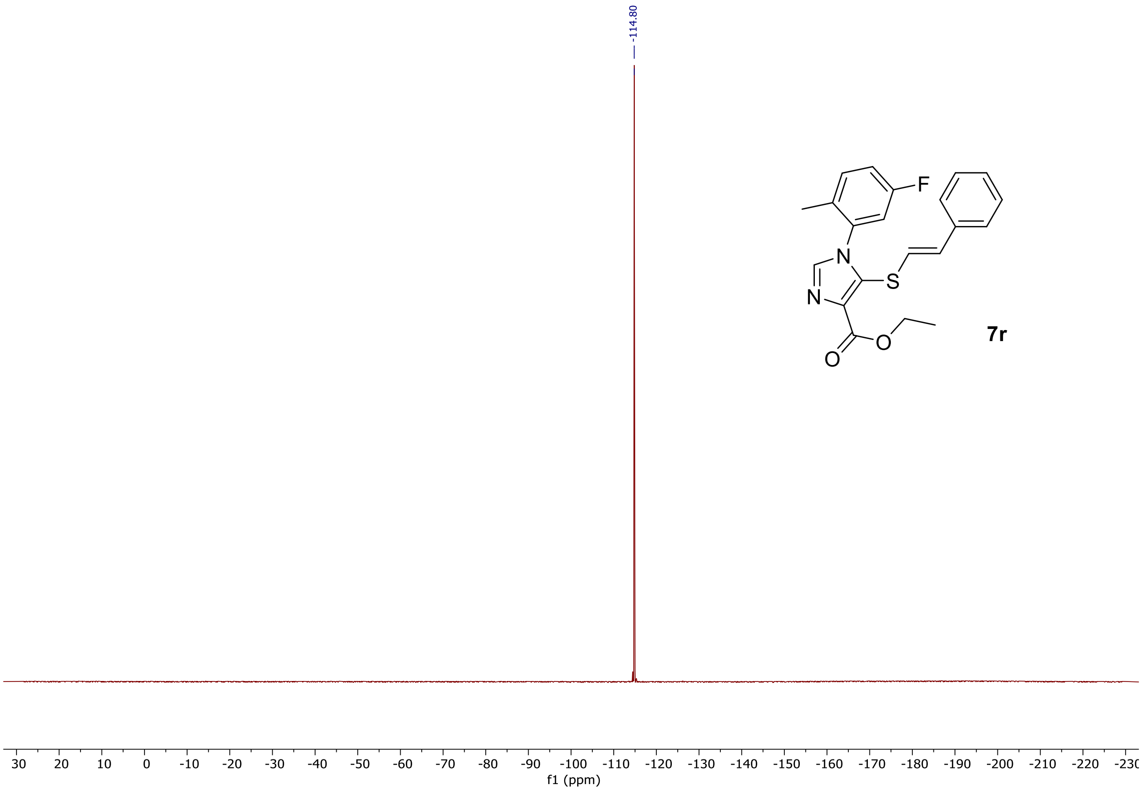

(*E*)-Ethyl 1-(5-fluoro-2-methylphenyl)-5-((4-methylstyryl)thio)-1H-imidazole-4-carboxylate (7s)

^{1}H RMN (300 MHz, $CDCl_3$, 300K)

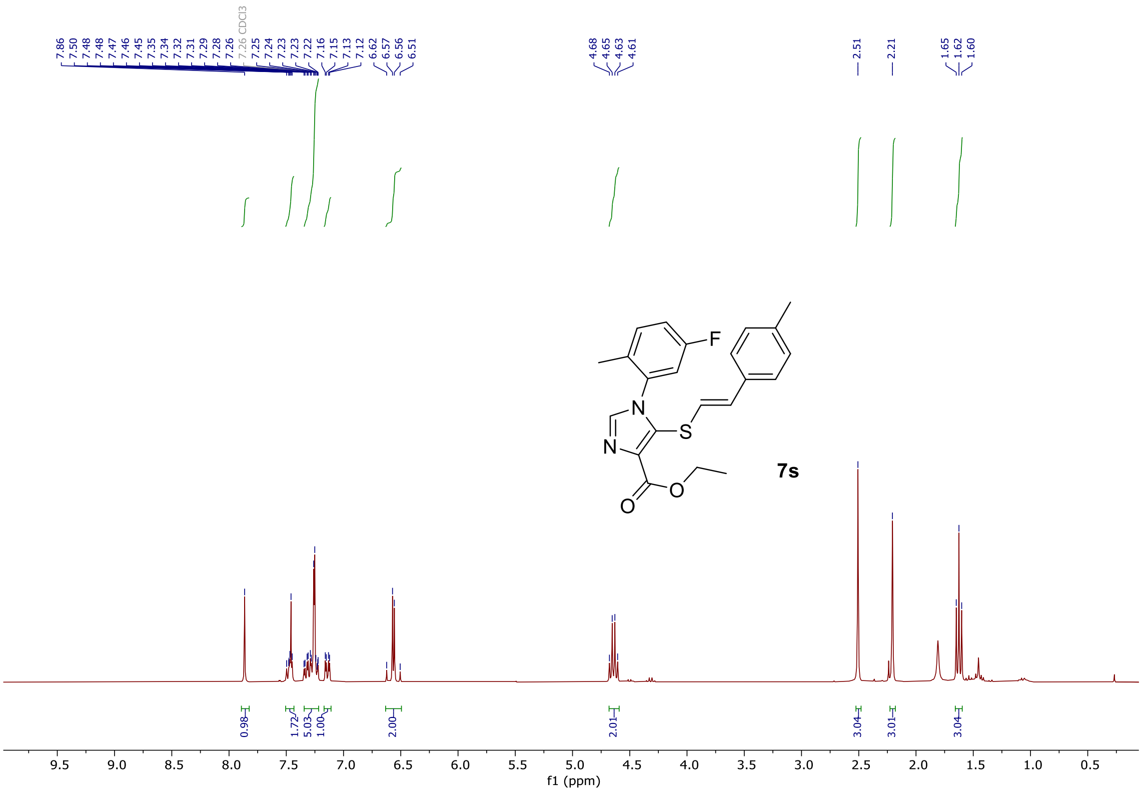

$^{13}C\{^1H\}$ **NMR** (75 MHz, $CDCl_3$, 300K)

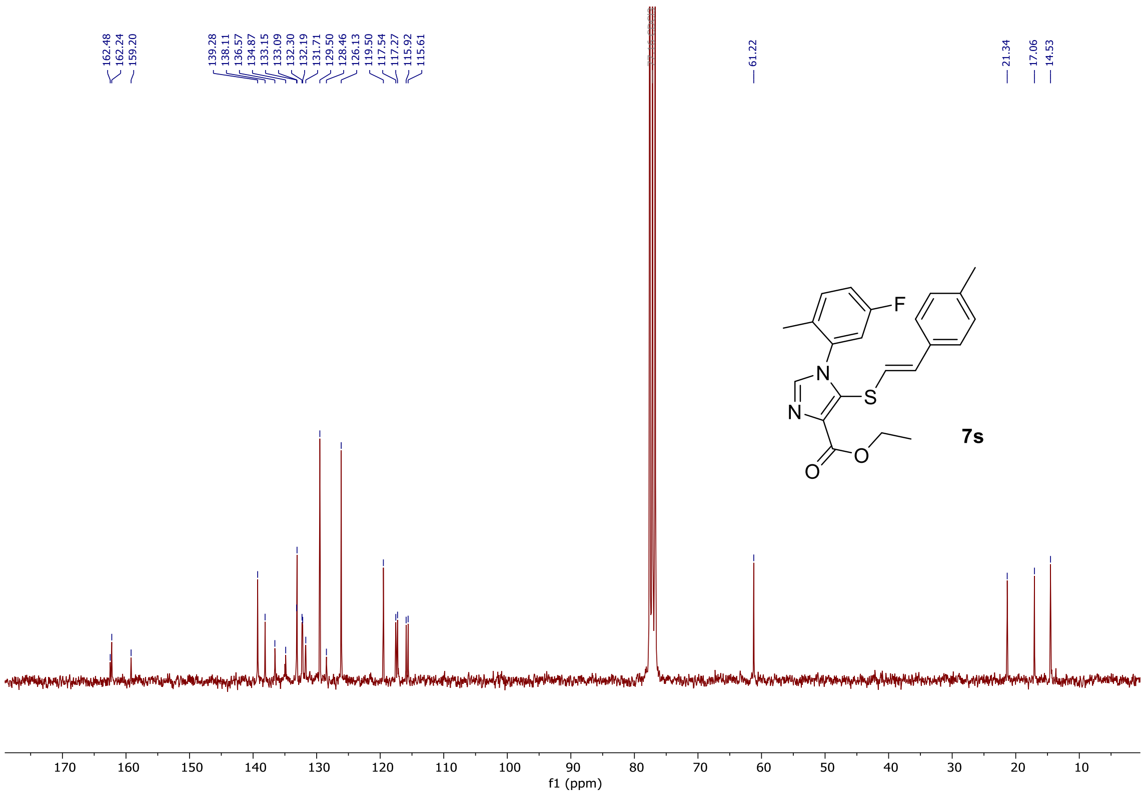

^{19}F **RMN** (282 MHz, $CDCl_3$, 300K)

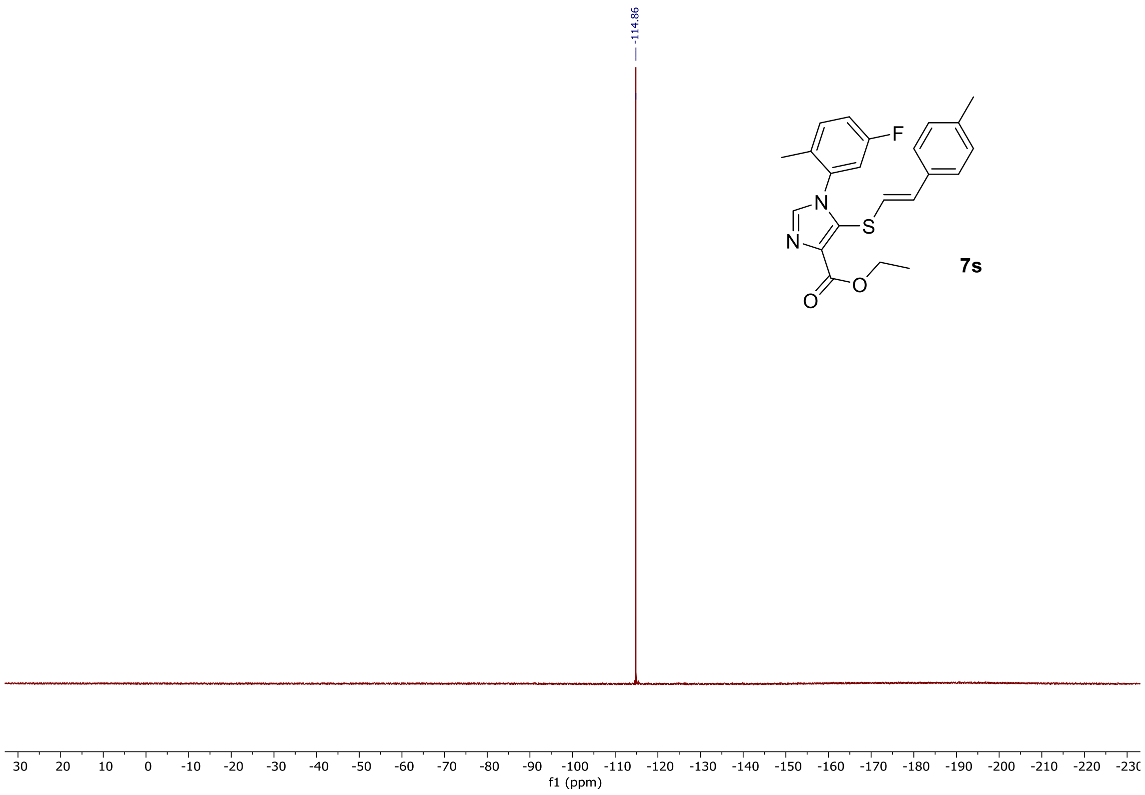

(*Z*)-Ethyl 1-(5-fluoro-2-methylphenyl)-5-((4-methylstyryl)thio)-1H-imidazole-4-carboxylate (7s’)

^{1}H RMN (300 MHz, $CDCl_3$, 300K)

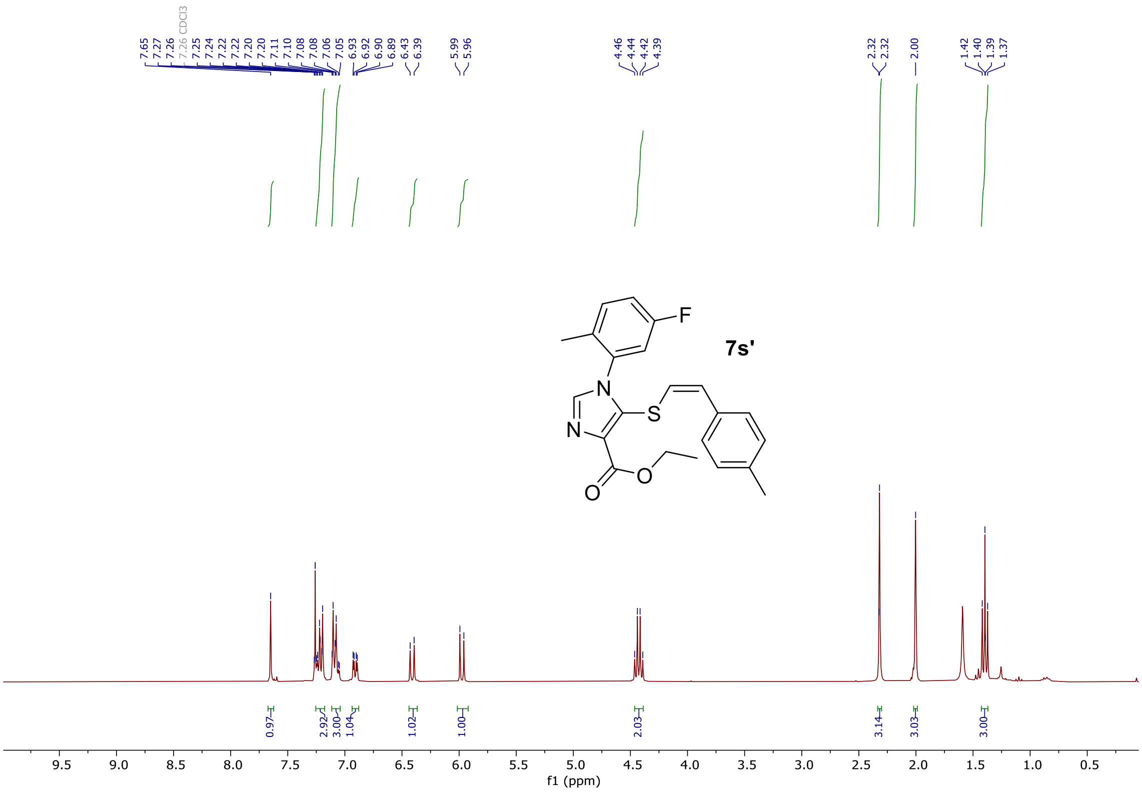

$^{13}C\{^{1}H\}$ NMR (75 MHz, $CDCl_3$, 300K)

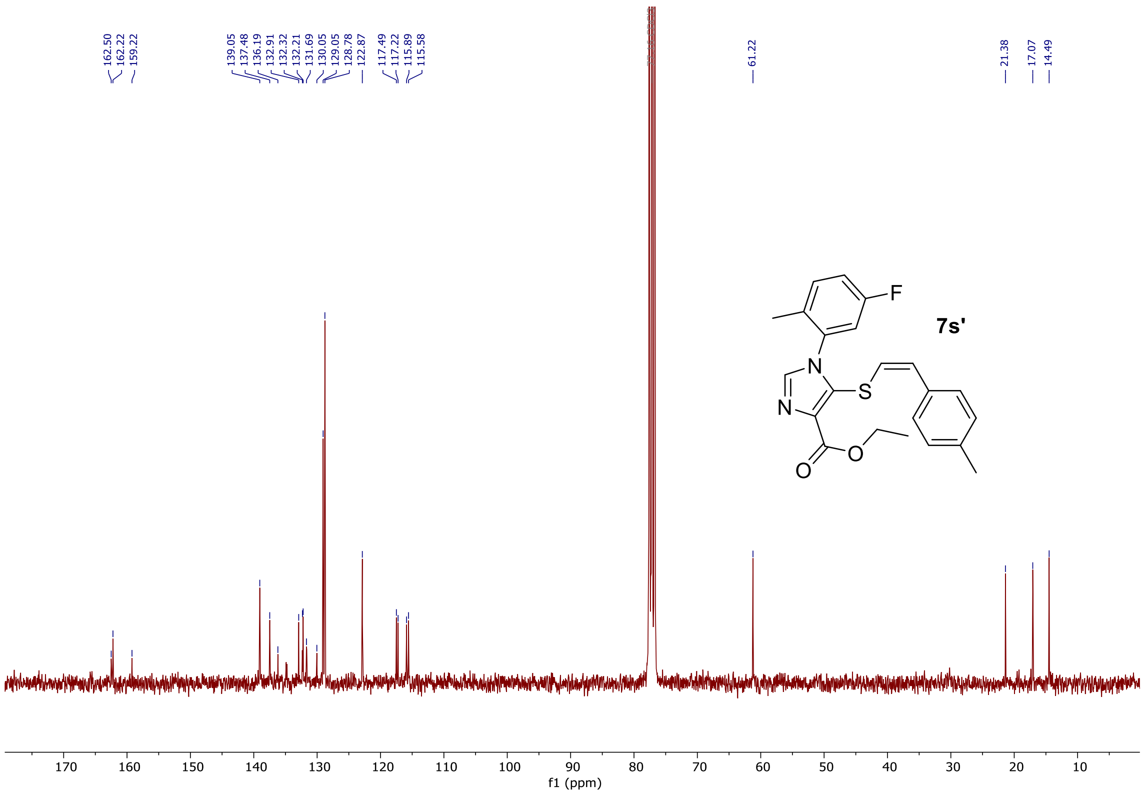

^{19}F RMN (282 MHz, $CDCl_3$, 300K)

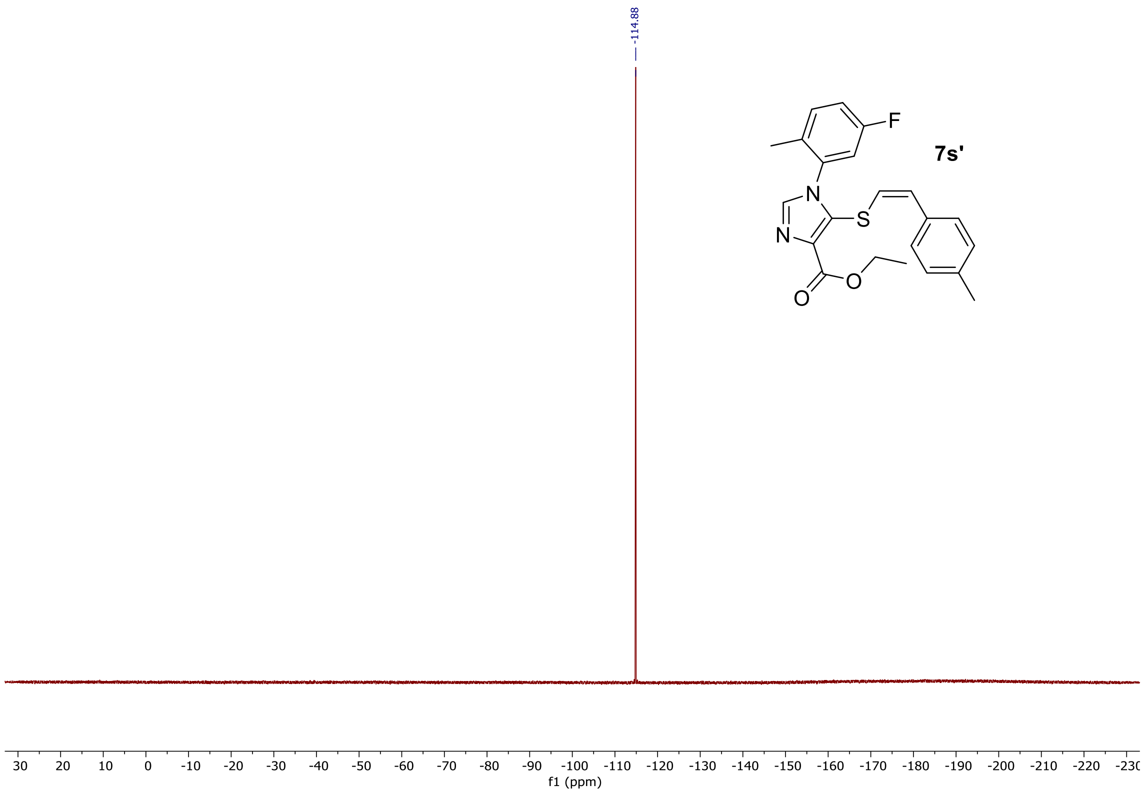

Ethyl 5-((4-(tert-butyl)styryl)thio)-1-(5-fluoro-2-methylphenyl)-1H-imidazole-4-carboxylate (7t)

^{1}H RMN (300 MHz, $CDCl_3$, 300K)

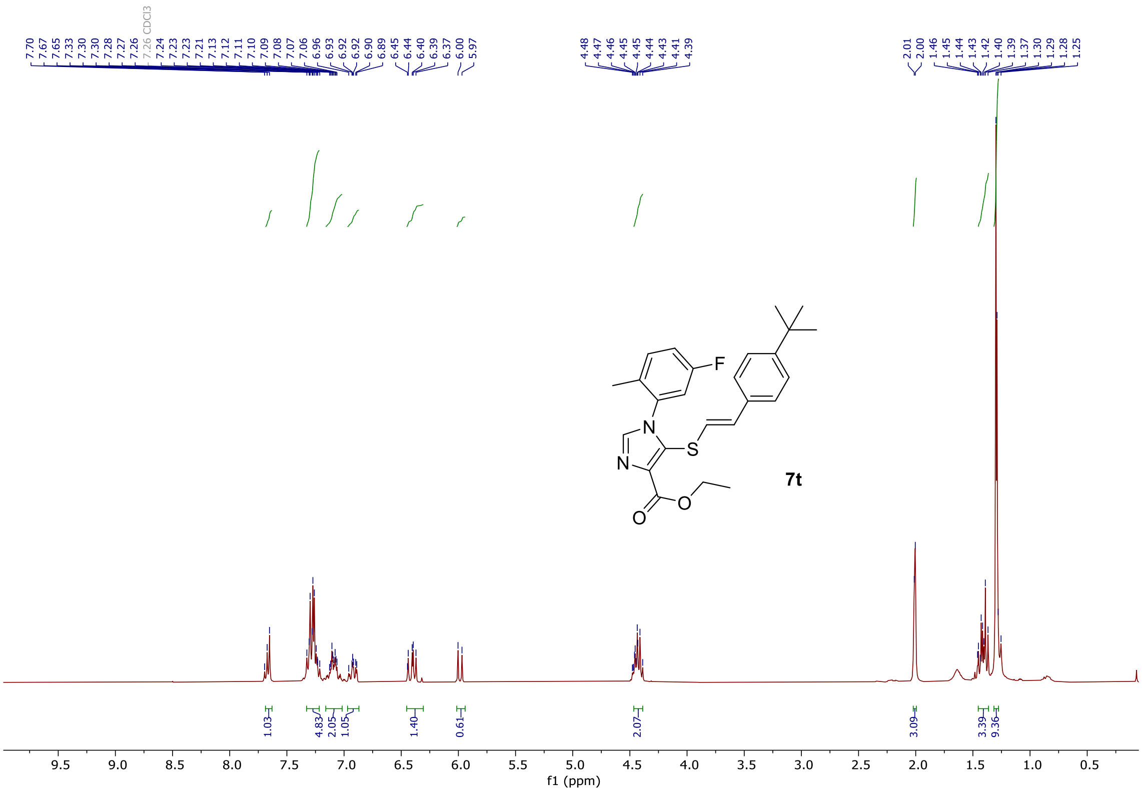

$^{13}C\{^{1}H\}$ NMR (75 MHz, $CDCl_3$, 300K)

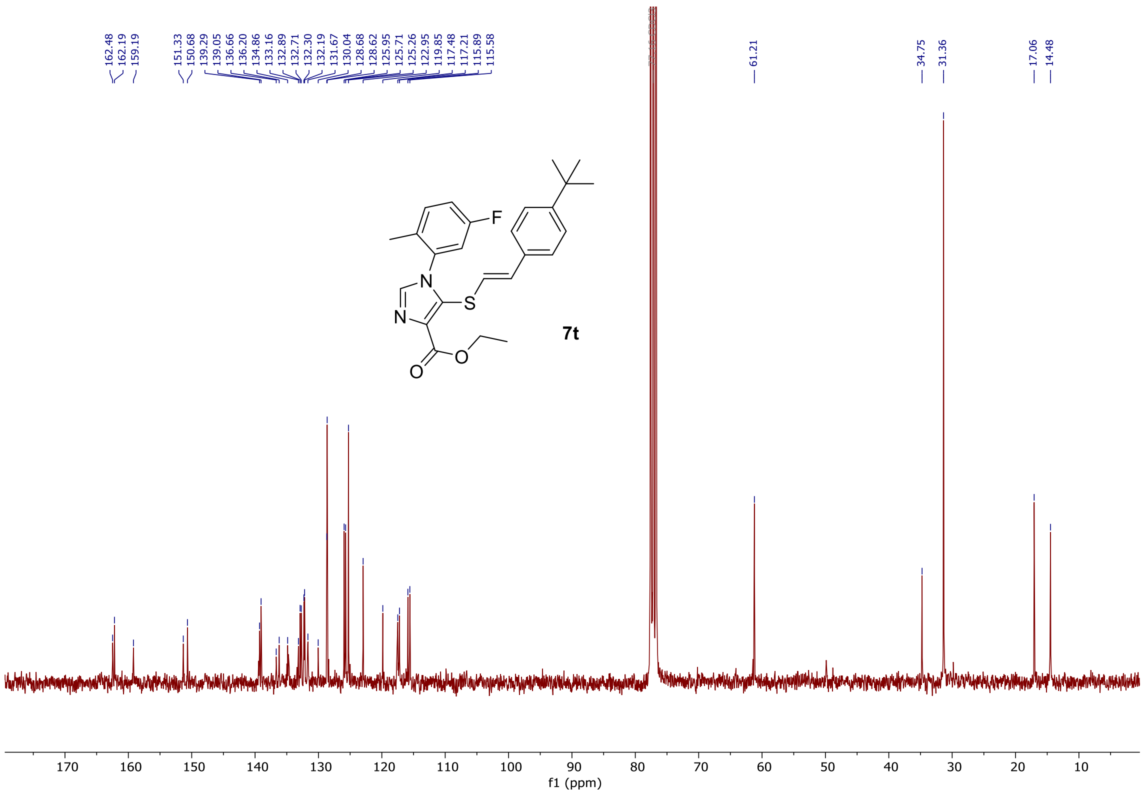

^{19}F RMN (282 MHz, $CDCl_3$, 300K)

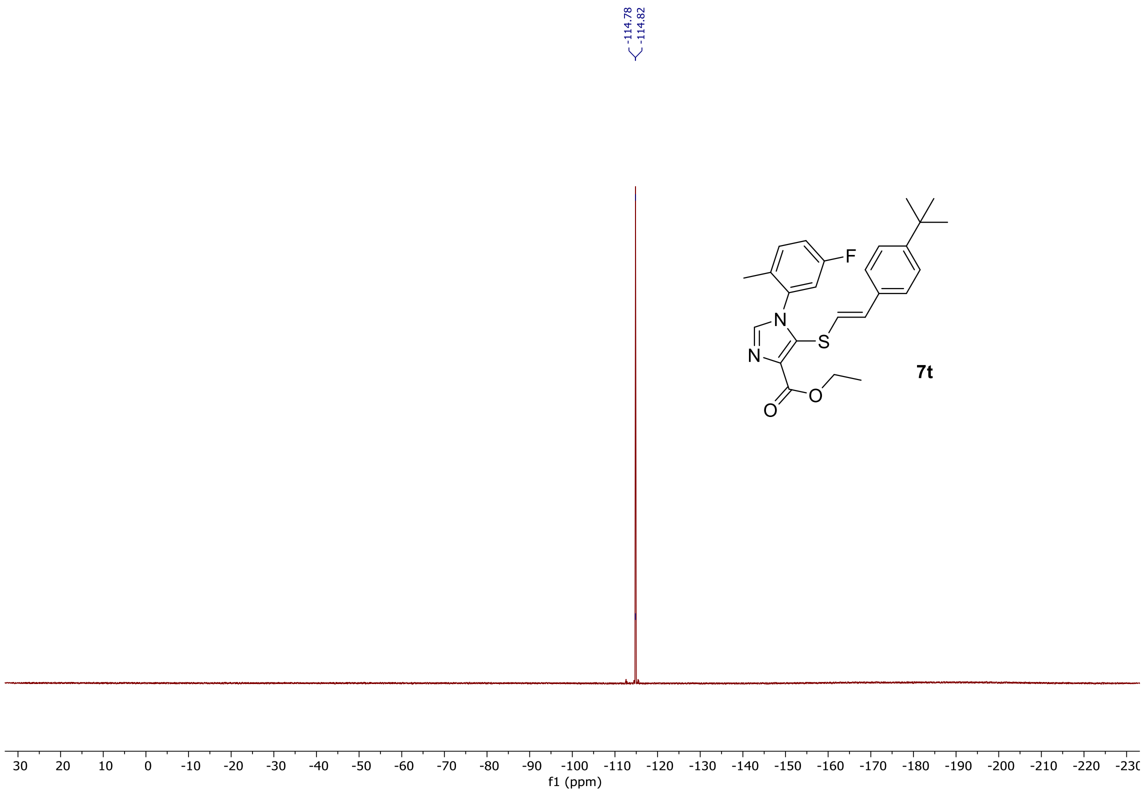

Ethyl 1-(4-methoxyphenyl)-5-(styrylthio)-1H-imidazole-4-carboxylate (7u)

^{1}H RMN (300 MHz, $CDCl_3$, 300K)

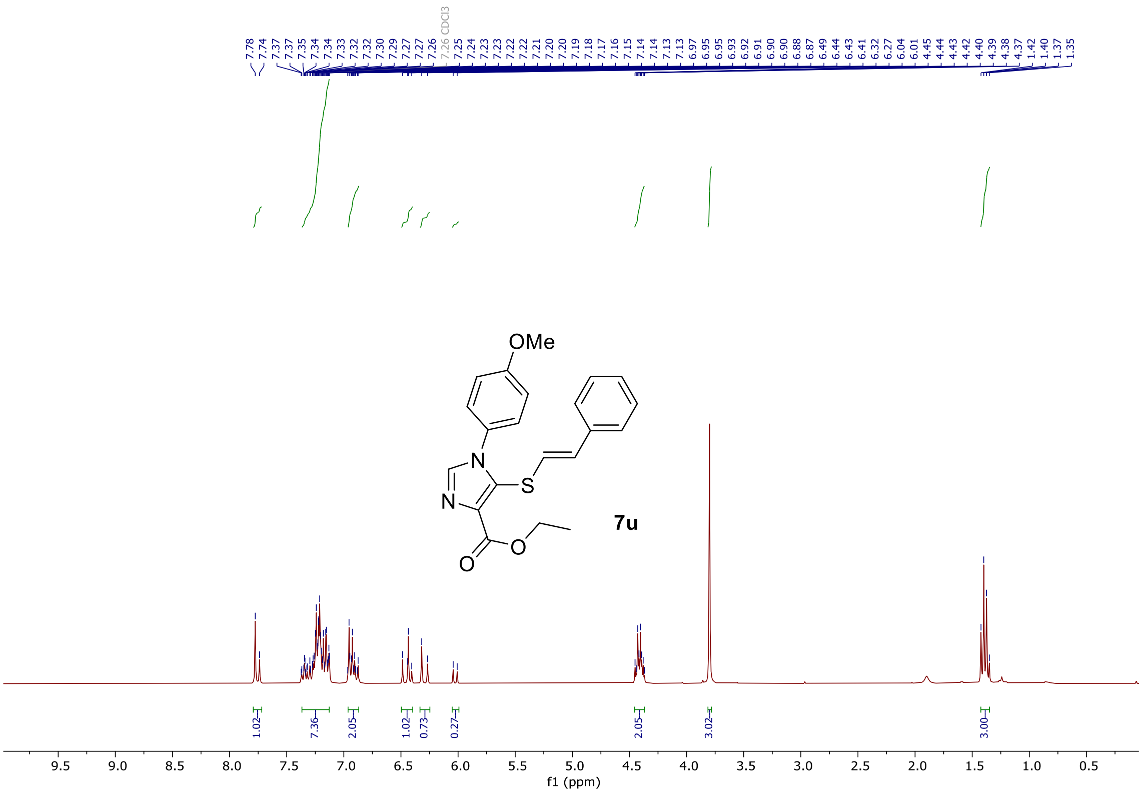

^{13}C{^{1}H} NMR (75 MHz, $CDCl_3$, 300K)

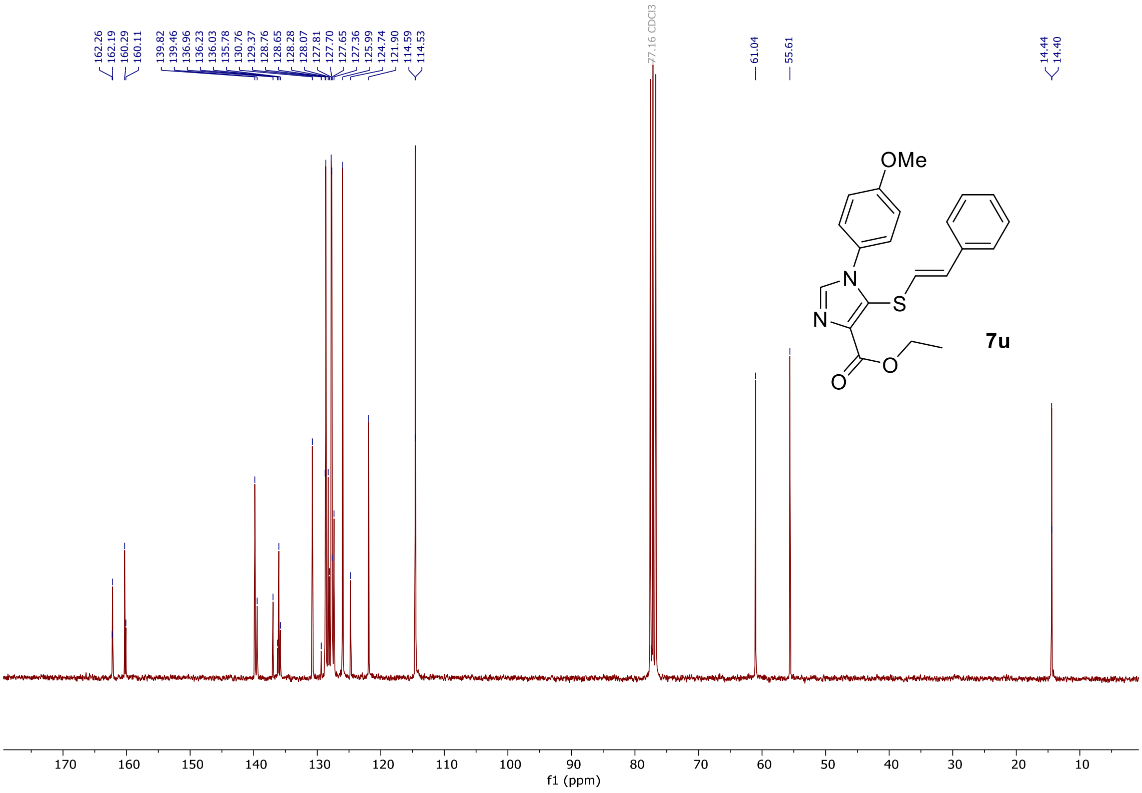

(*E*)-Ethyl 1-(4-methoxyphenyl)-5-((4-methylstyryl)thio)-1H-imidazole-4-carboxylate (7v)

^{1}H RMN (300 MHz, $CDCl_3$, 300K)

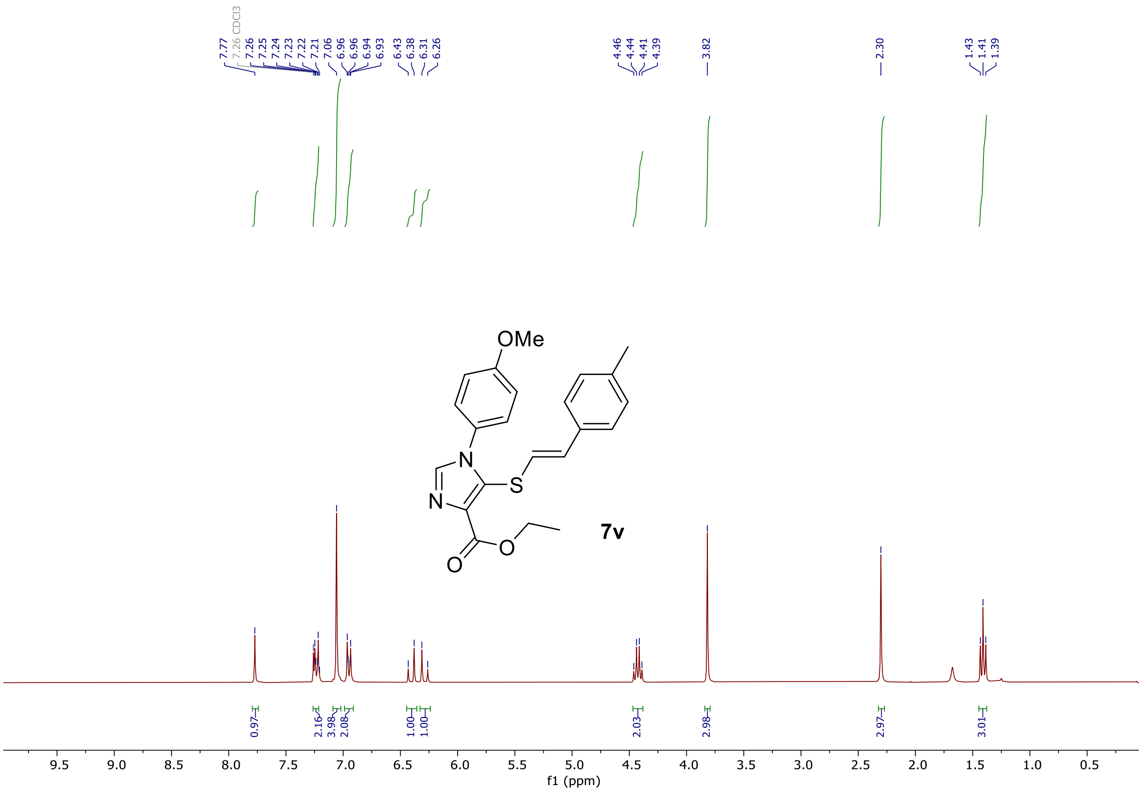

^{13}C{^{1}H} NMR (75 MHz, $CDCl_3$, 300K)

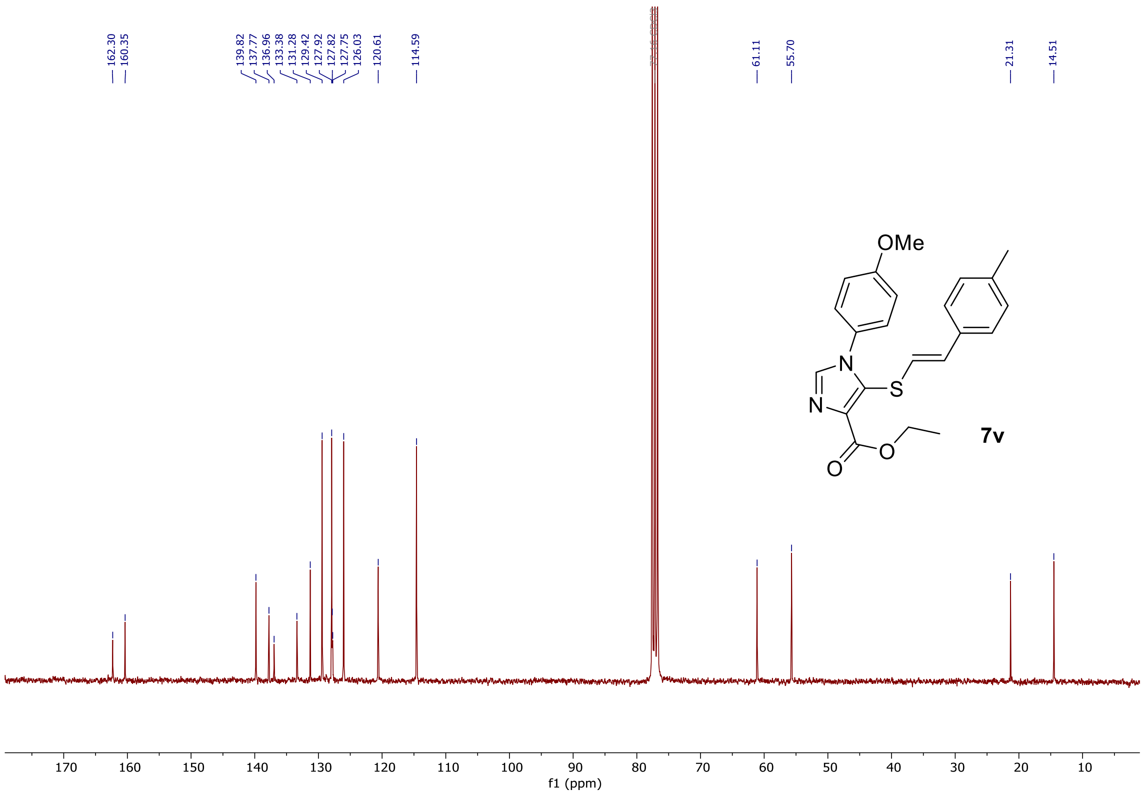

(*Z*)-Ethyl 1-(4-methoxyphenyl)-5-((4-methylstyryl)thio)-1H-imidazole-4-carboxylate (7v')

^{1}H RMN (300 MHz, $CDCl_3$, 300K)

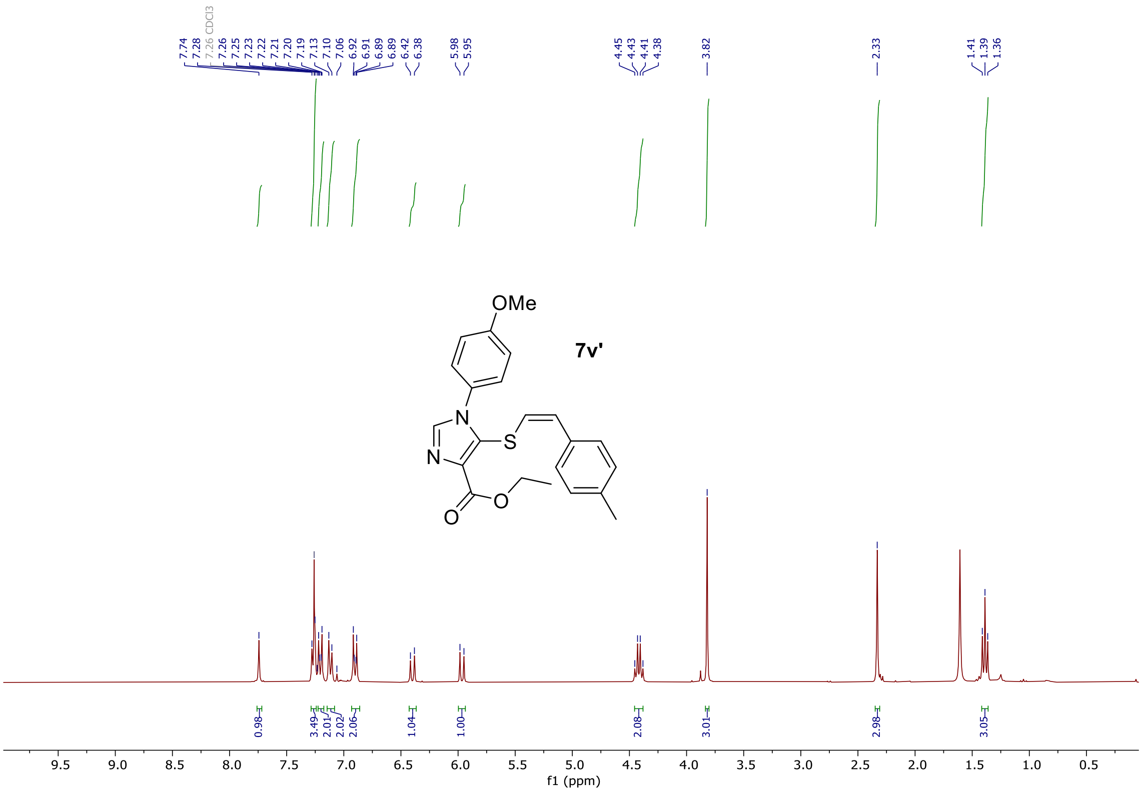

^{13}C{^{1}H} NMR (75 MHz, $CDCl_3$, 300K)

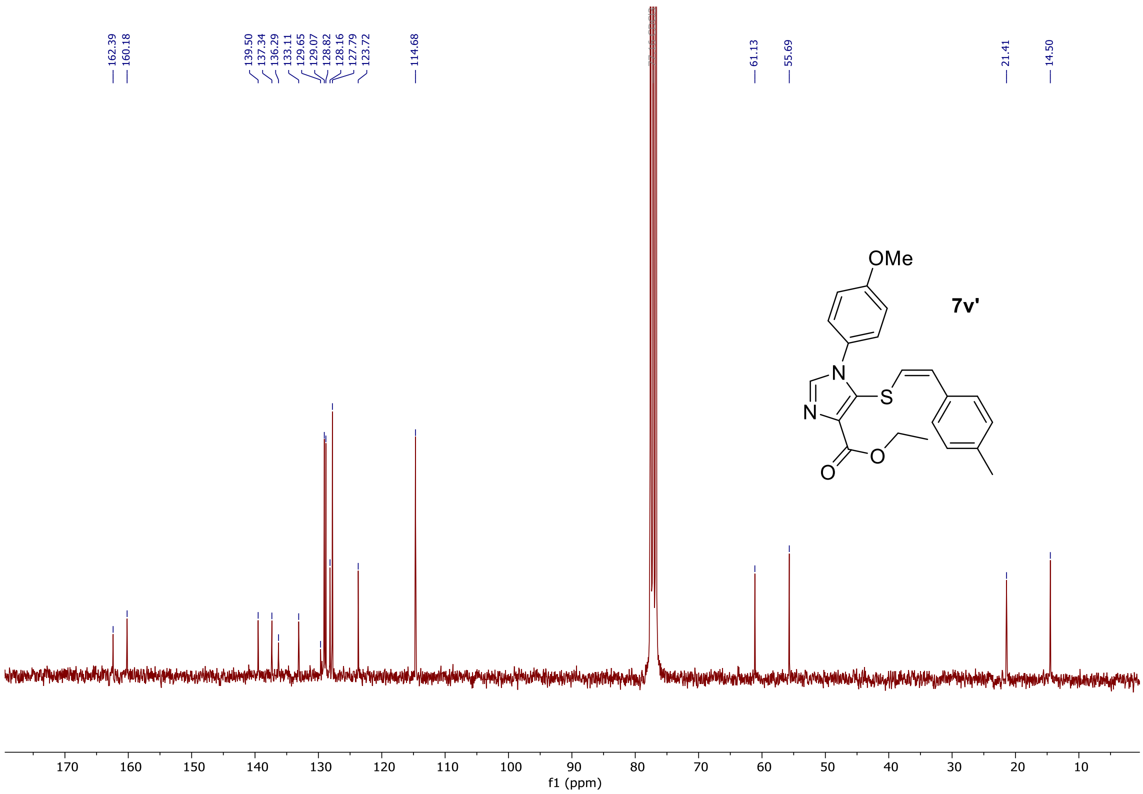

Ethyl 5-(styrylthio)-1-(4-(trifluoromethyl)phenyl)-1H-imidazole-4-carboxylate (7w)

^{1}H RMN (300 MHz, $CDCl_3$, 300K)

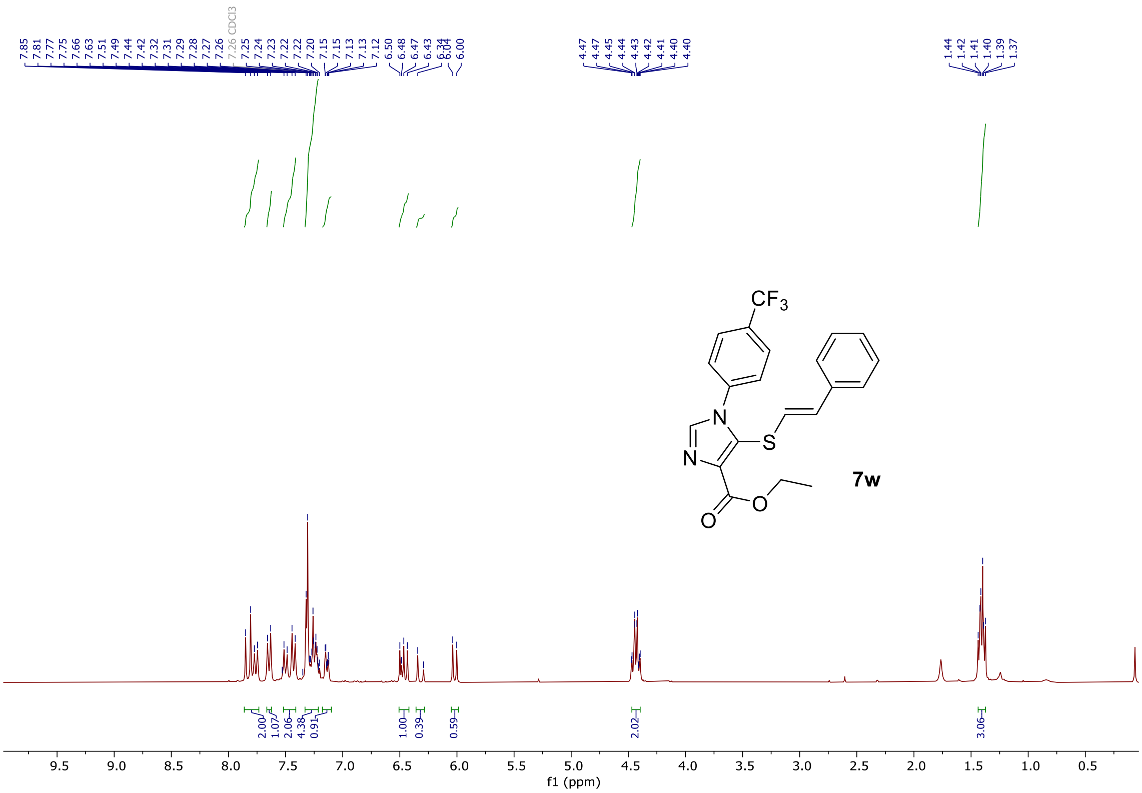

^{13}C{^{1}H} NMR (75 MHz, $CDCl_3$, 300K)

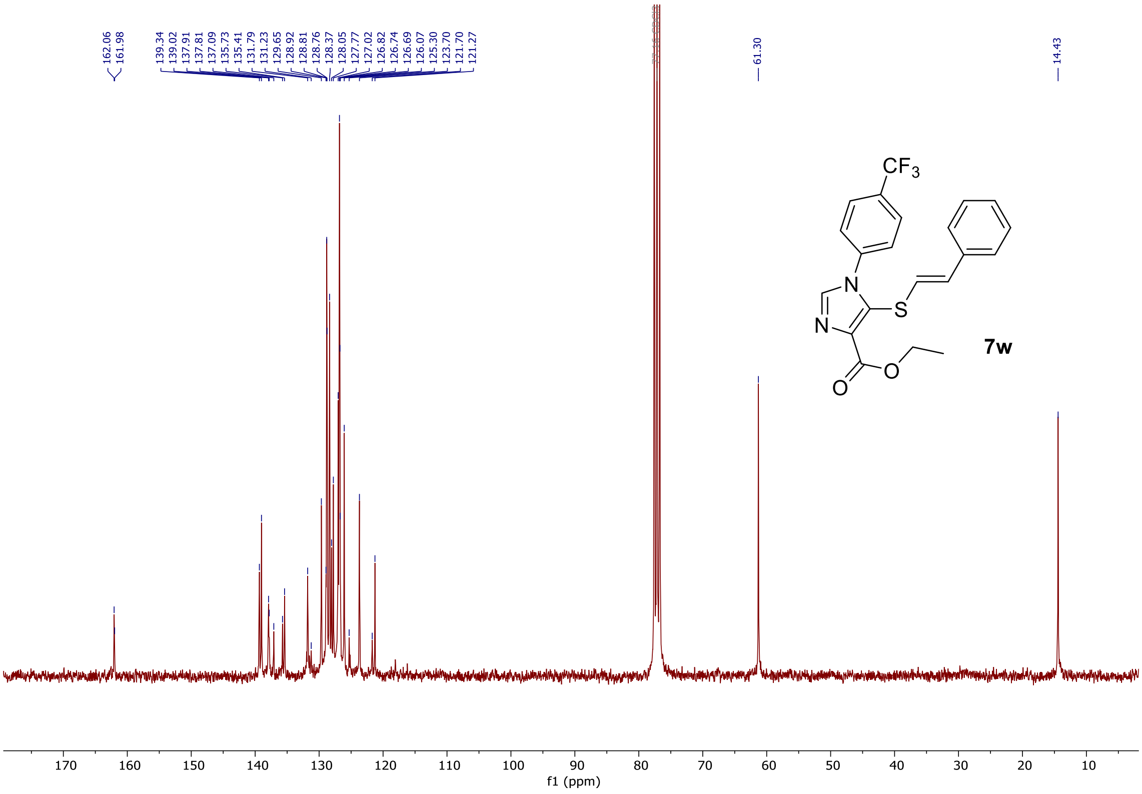

^{19}F RMN (282 MHz, $CDCl_3$, 300K)

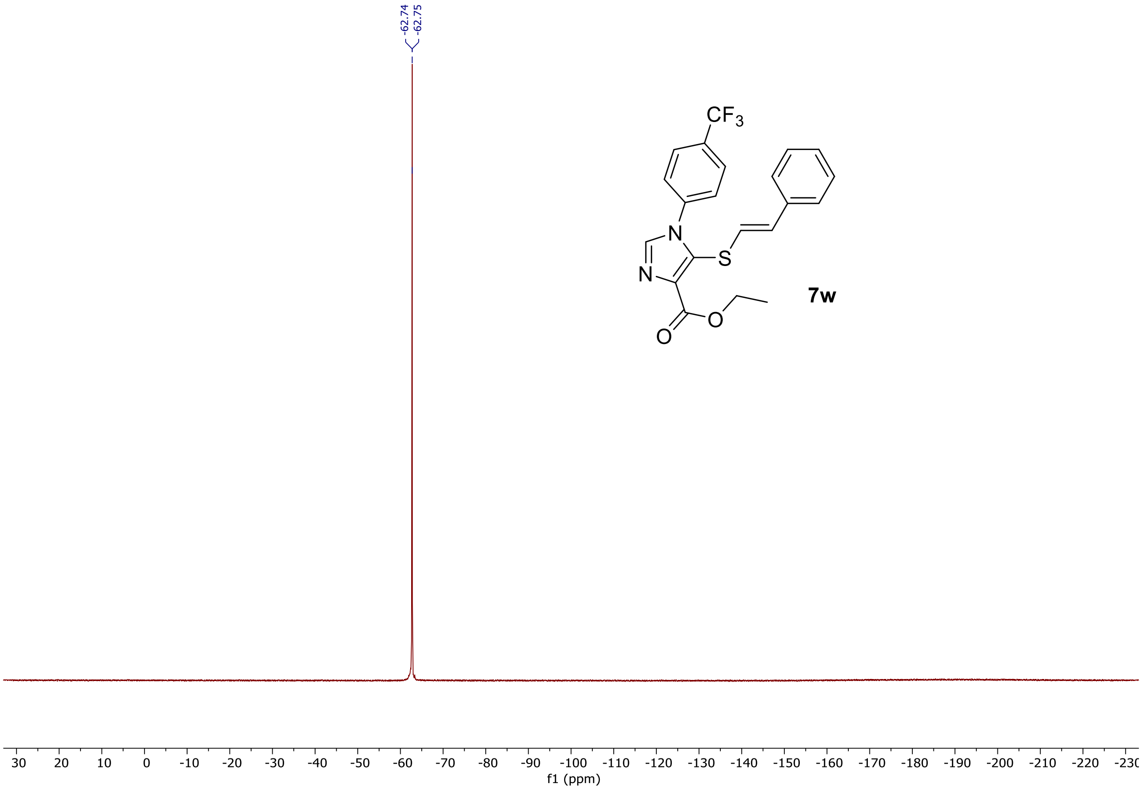

Ethyl 5-((2-(naphthalen-2-yl)vinyl)thio)-1-(4-(trifluoromethyl)phenyl)-1H-imidazole-4-carboxylate (7x)

^{1}H RMN (300 MHz, $CDCl_3$, 300K)

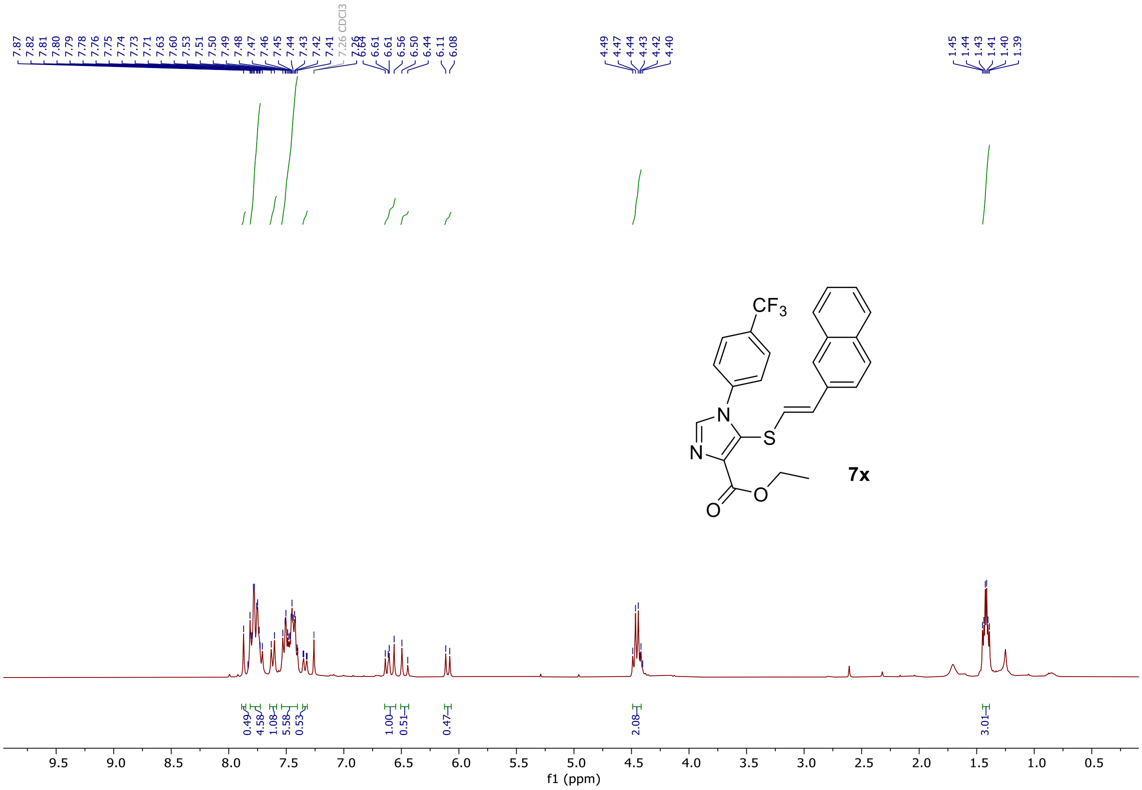

$^{13}C\{^{1}H\}$ NMR (75 MHz, $CDCl_3$, 300K)

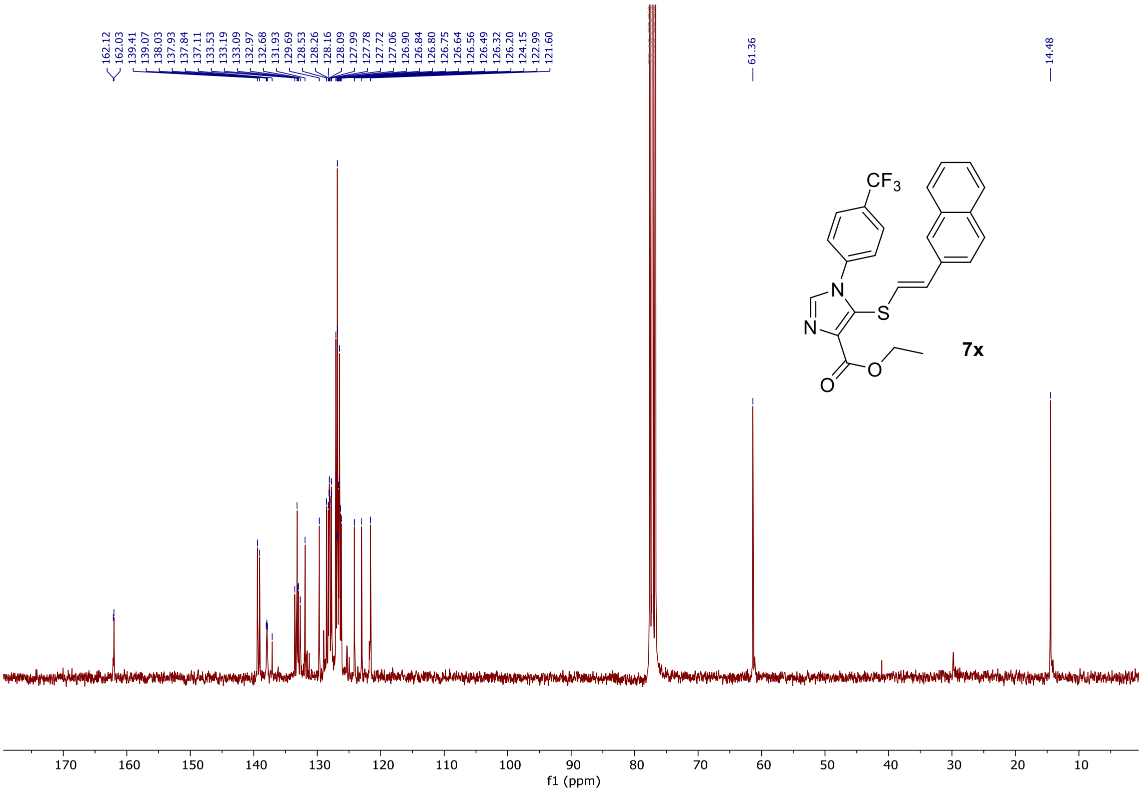

^{19}F RMN (282 MHz, $CDCl_3$, 300K)

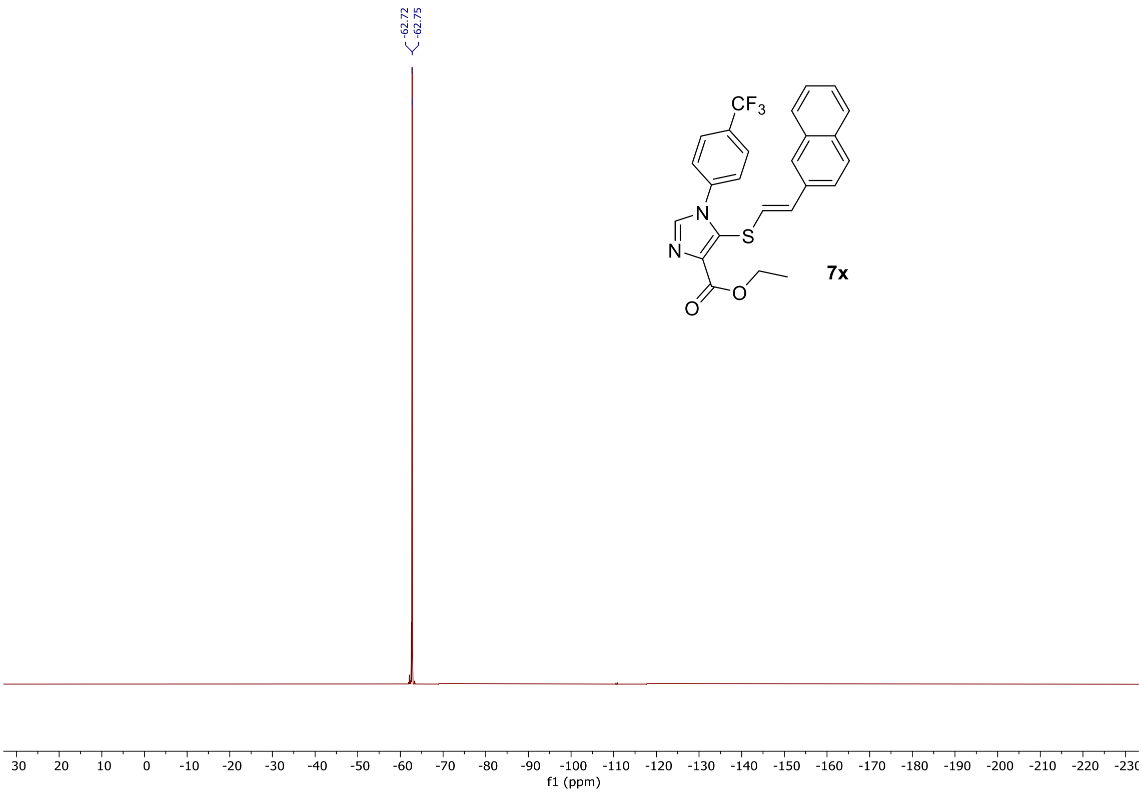

(*E*)-Ethyl 1-(4-fluorophenyl)-5-(styrylthio)-1H-imidazole-4-carboxylate (7y)

^{1}H RMN (300 MHz, $CDCl_3$, 300K)

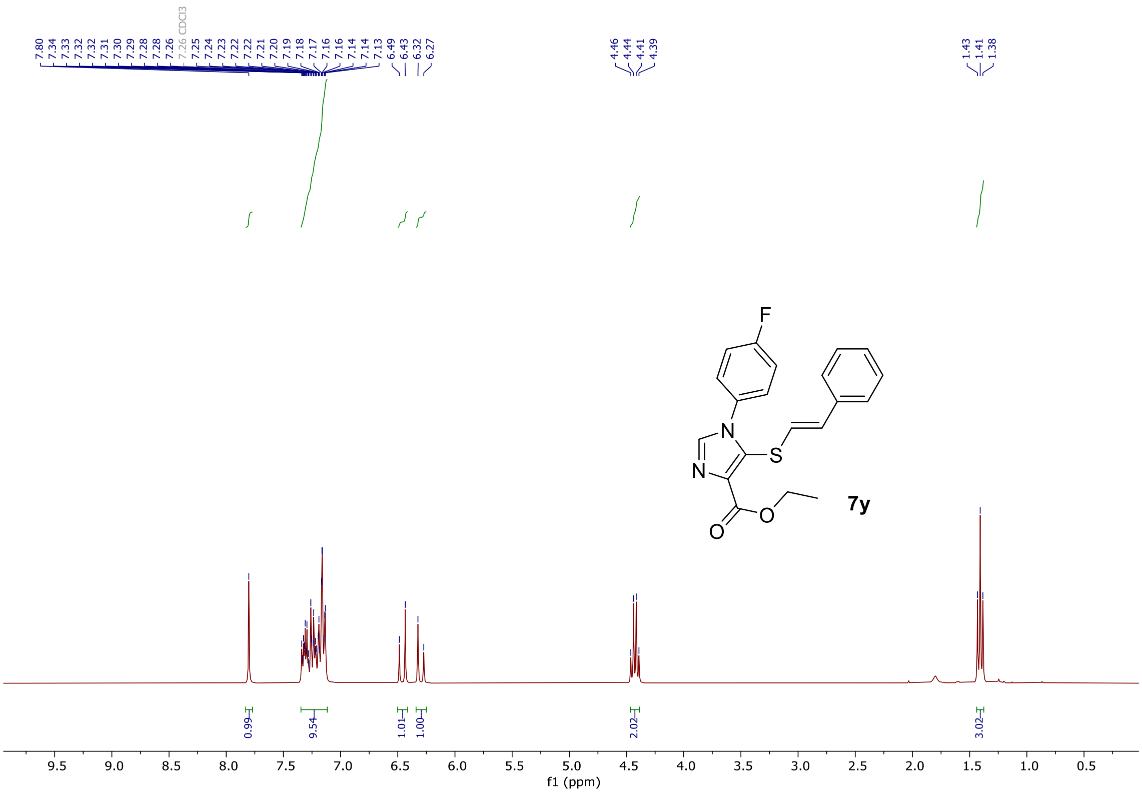

^{13}C{^{1}H} NMR (75 MHz, $CDCl_3$, 300K)

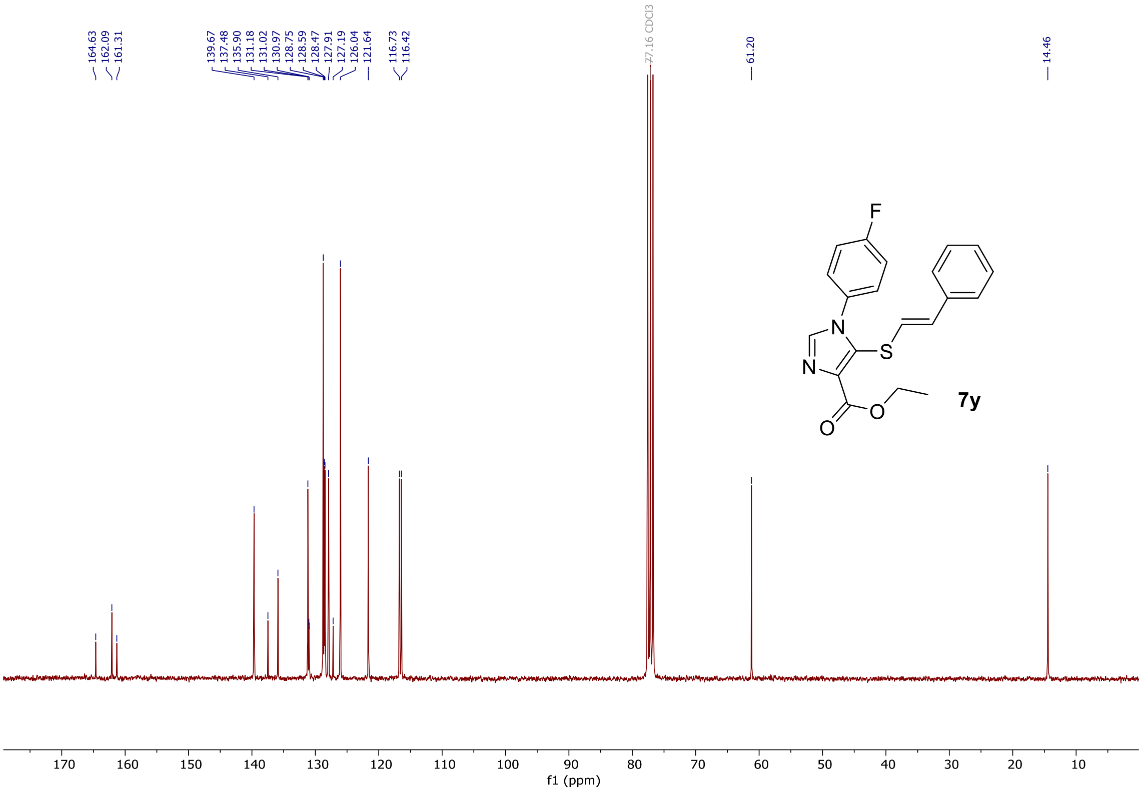

^{19}F RMN (282 MHz, $CDCl_3$, 300K)

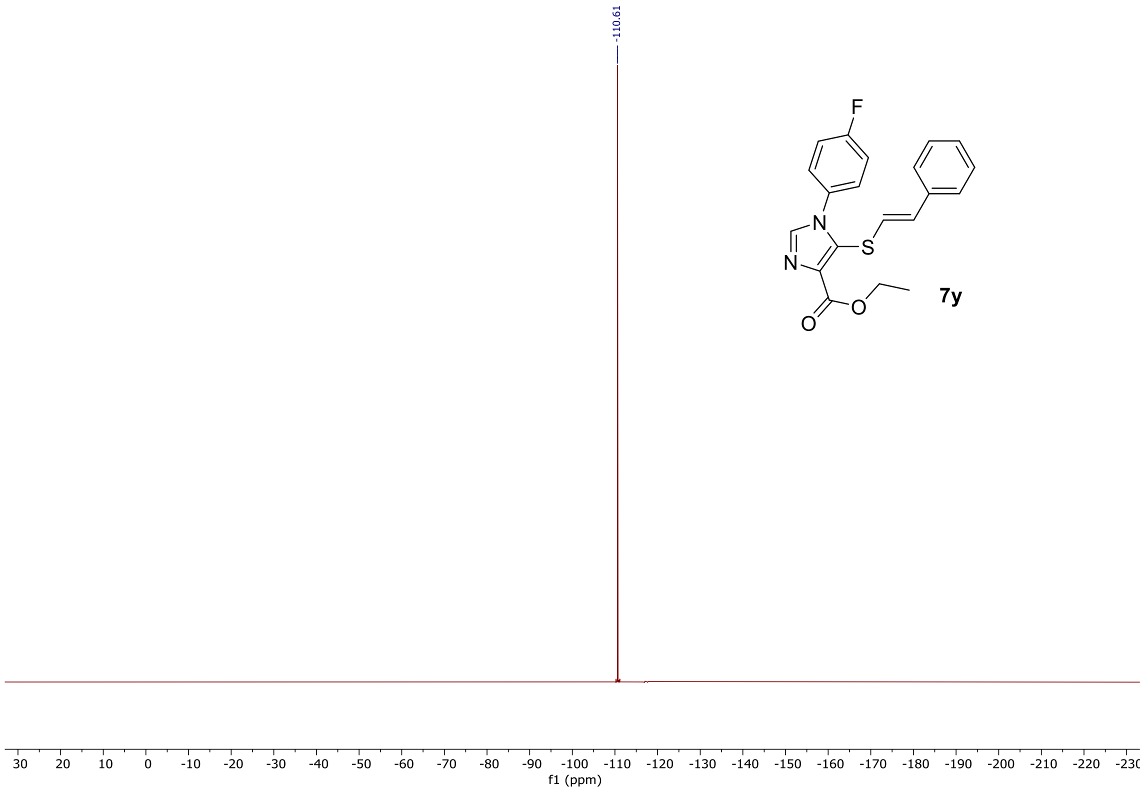

(*Z*)-Ethyl 1-(4-fluorophenyl)-5-(styrylthio)-1H-imidazole-4-carboxylate (7y')

^{1}H RMN (300 MHz, $CDCl_3$, 300K)

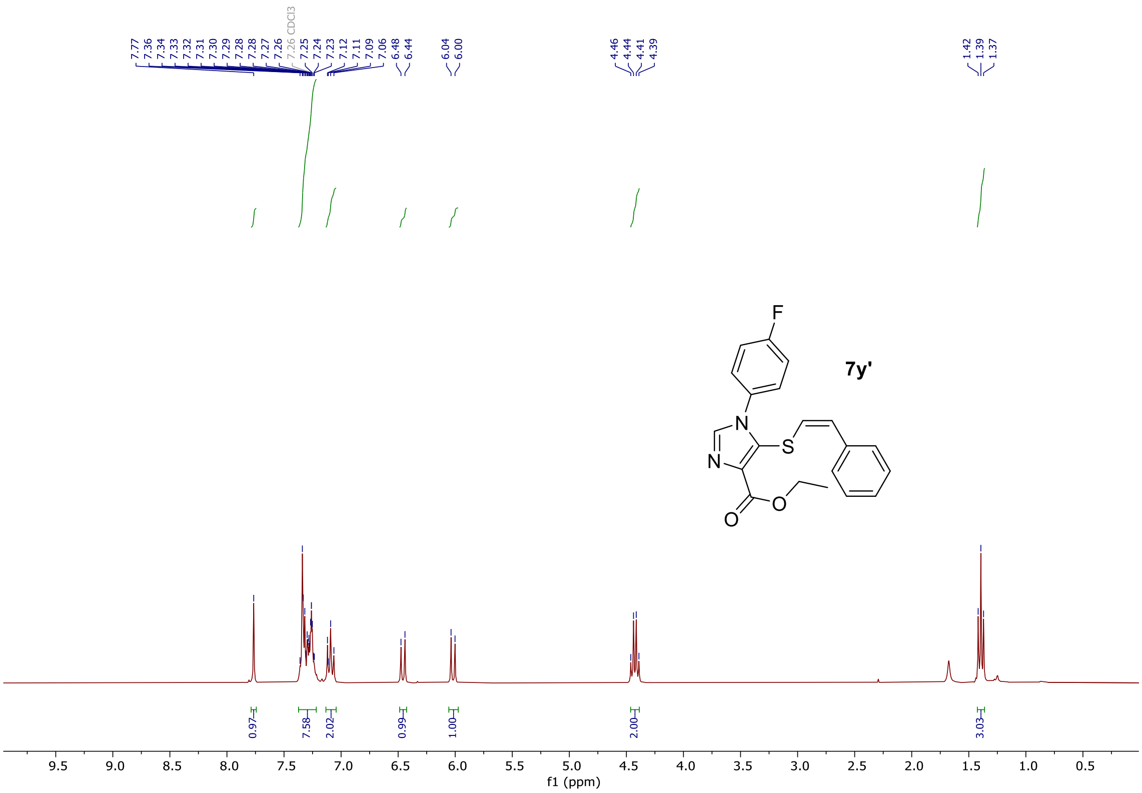

^{13}C{^{1}H} NMR (75 MHz, $CDCl_3$, 300K)

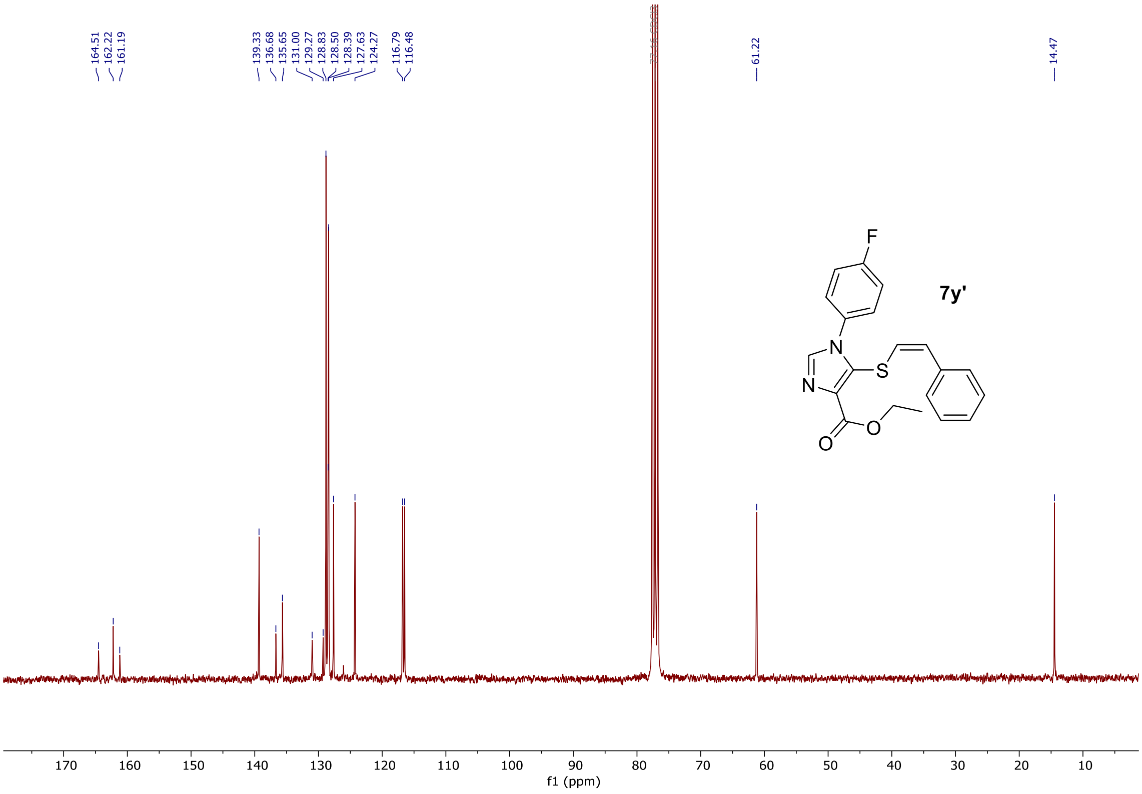

^{19}F RMN (282 MHz, $CDCl_3$, 300K)

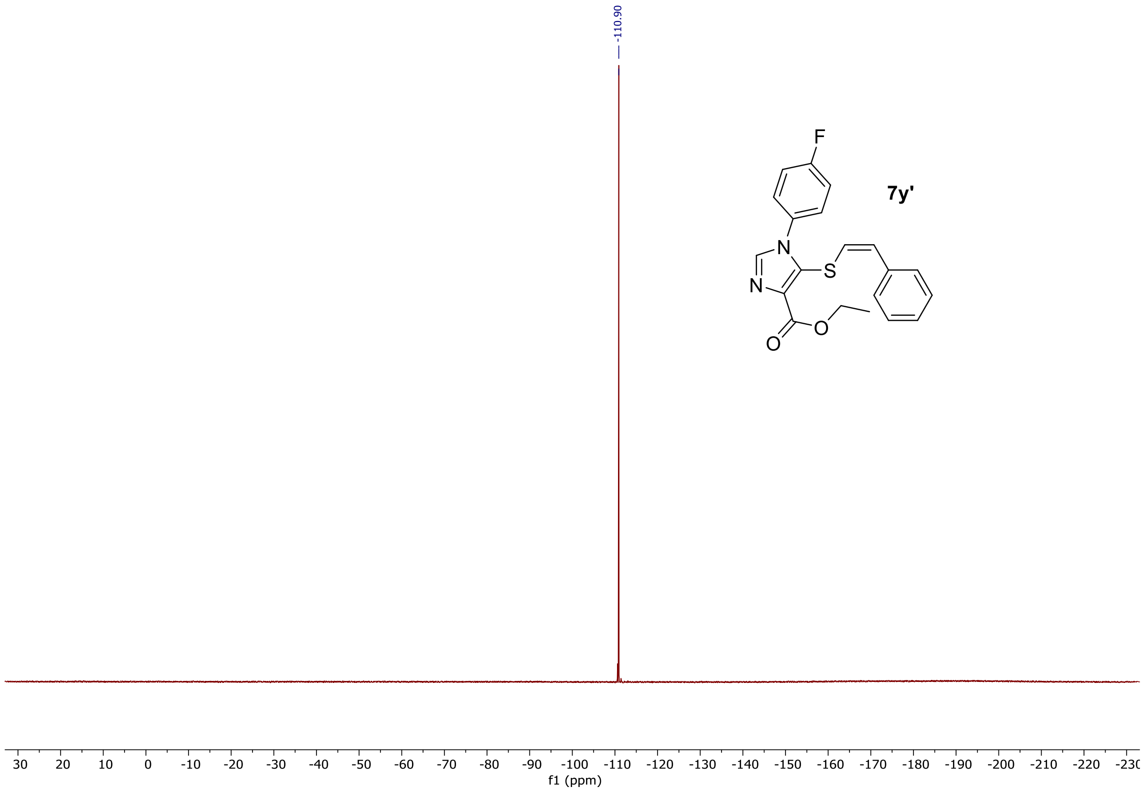

(*E*)-Ethyl 1-(4-fluorophenyl)-5-((2-(naphthalen-2-yl)vinyl)thio)-1H-imidazole-4-carboxylate (7z)

^{1}H RMN (300 MHz, $CDCl_3$, 300K)

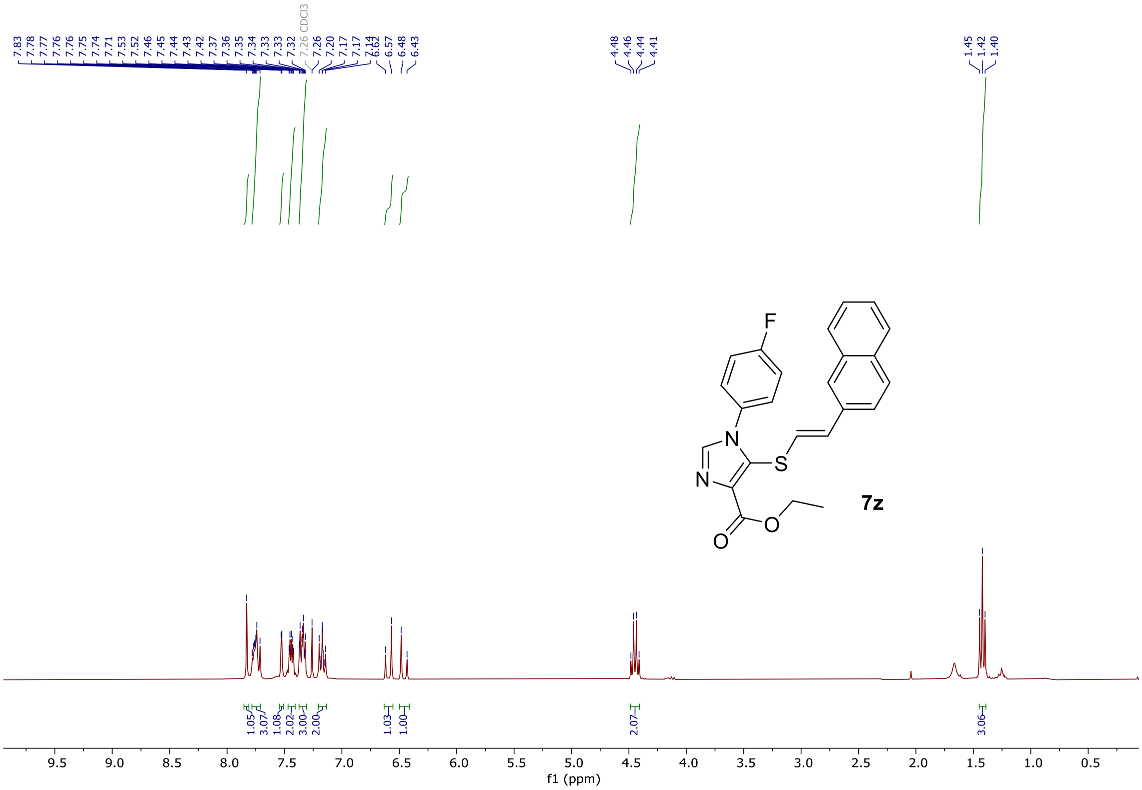

$^{13}C\{^{1}H\}$ NMR (75 MHz, $CDCl_3$, 300K)

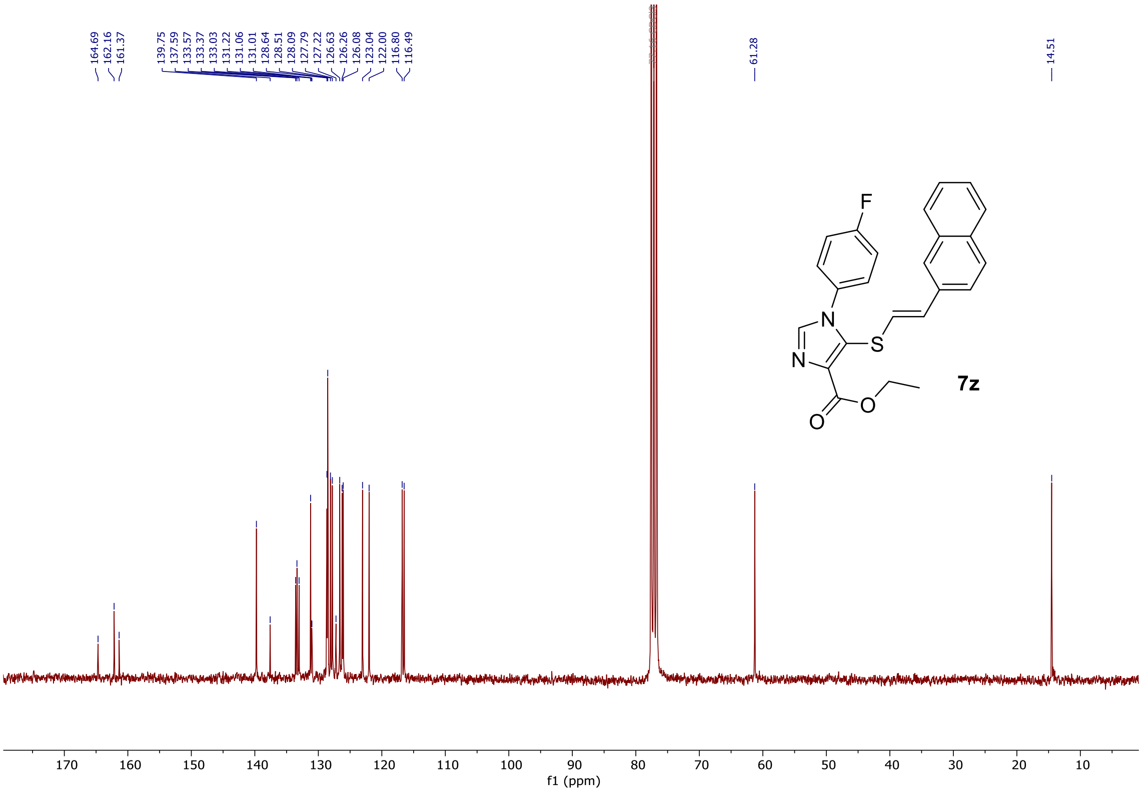

^{19}F RMN (282 MHz, $CDCl_3$, 300K)

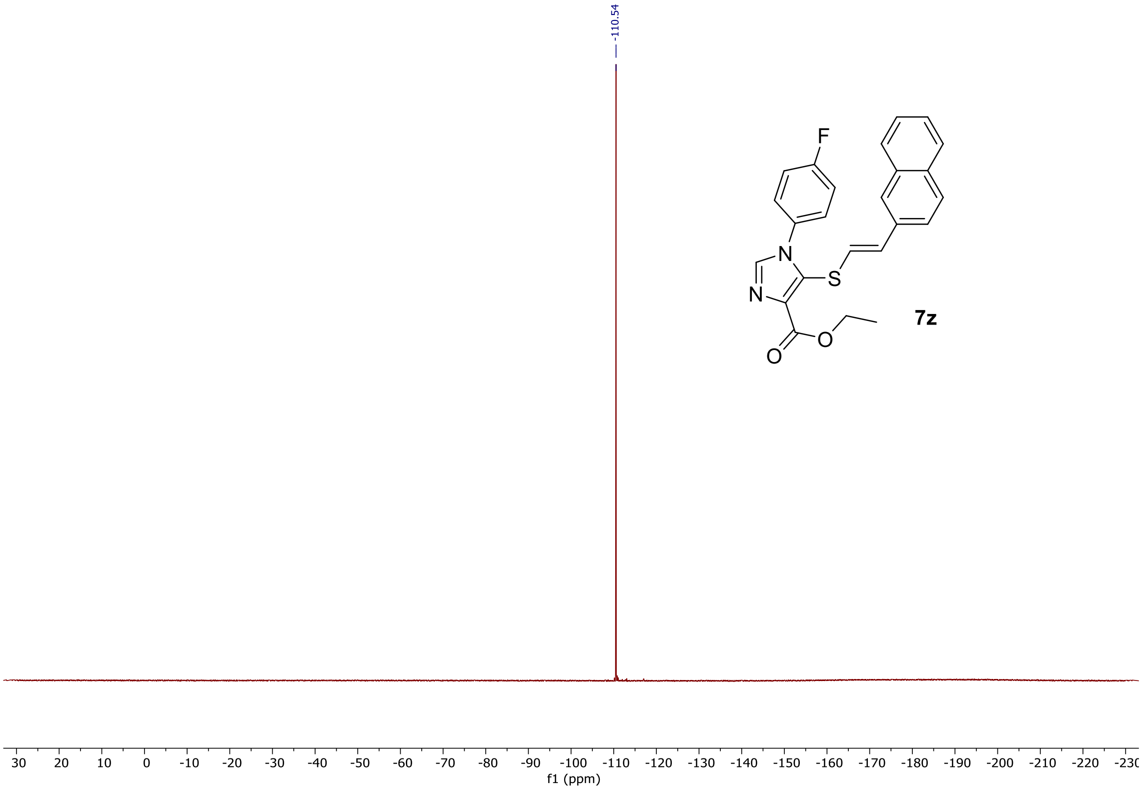

(*Z*)-Ethyl 1-(4-fluorophenyl)-5-((2-(naphthalen-2-yl)vinyl)thio)-1H-imidazole-4-carboxylate (7z')

^{1}H RMN (300 MHz, $CDCl_3$, 300K)

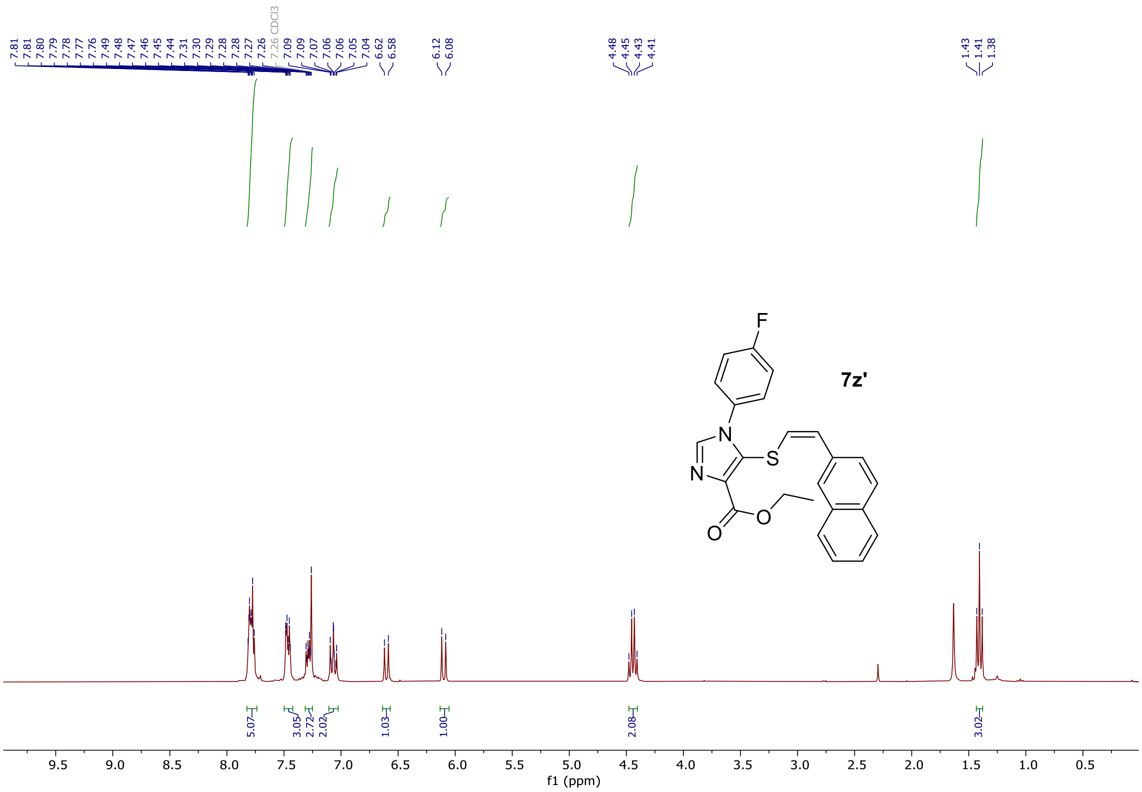

$^{13}C\{^1H\}$ NMR (75 MHz, $CDCl_3$, 300K)

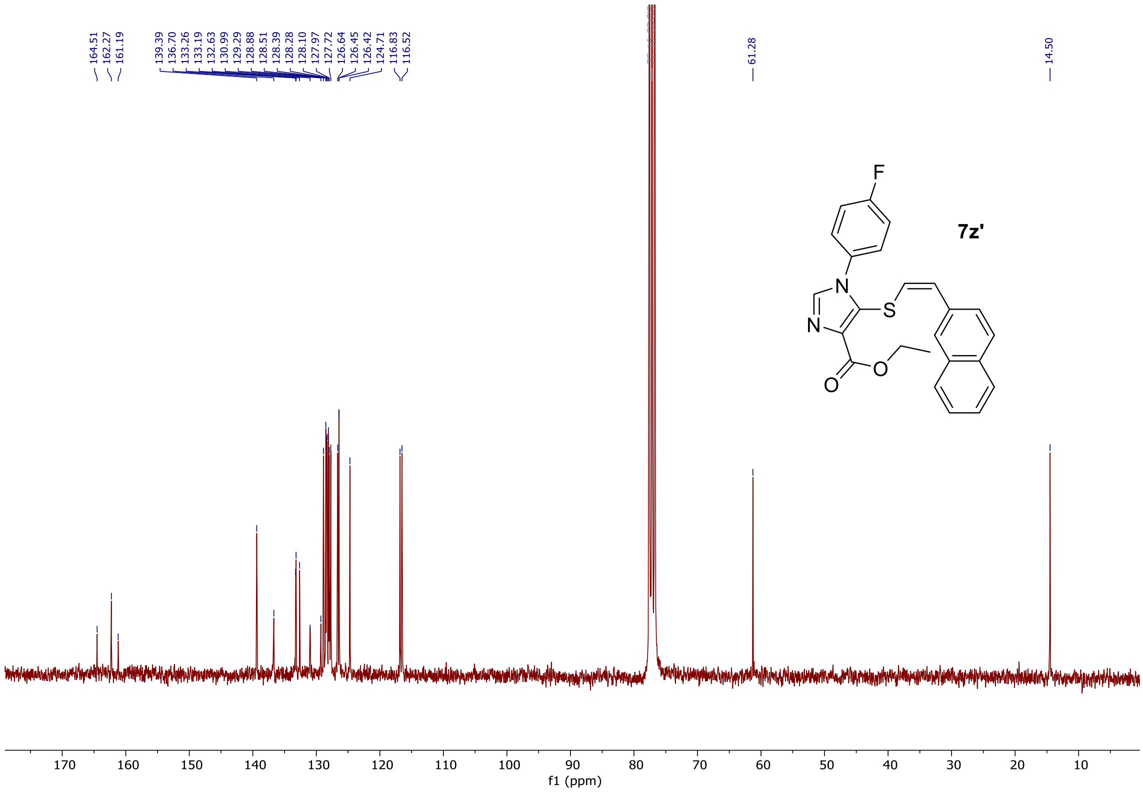

^{19}F RMN (282 MHz, $CDCl_3$, 300K)

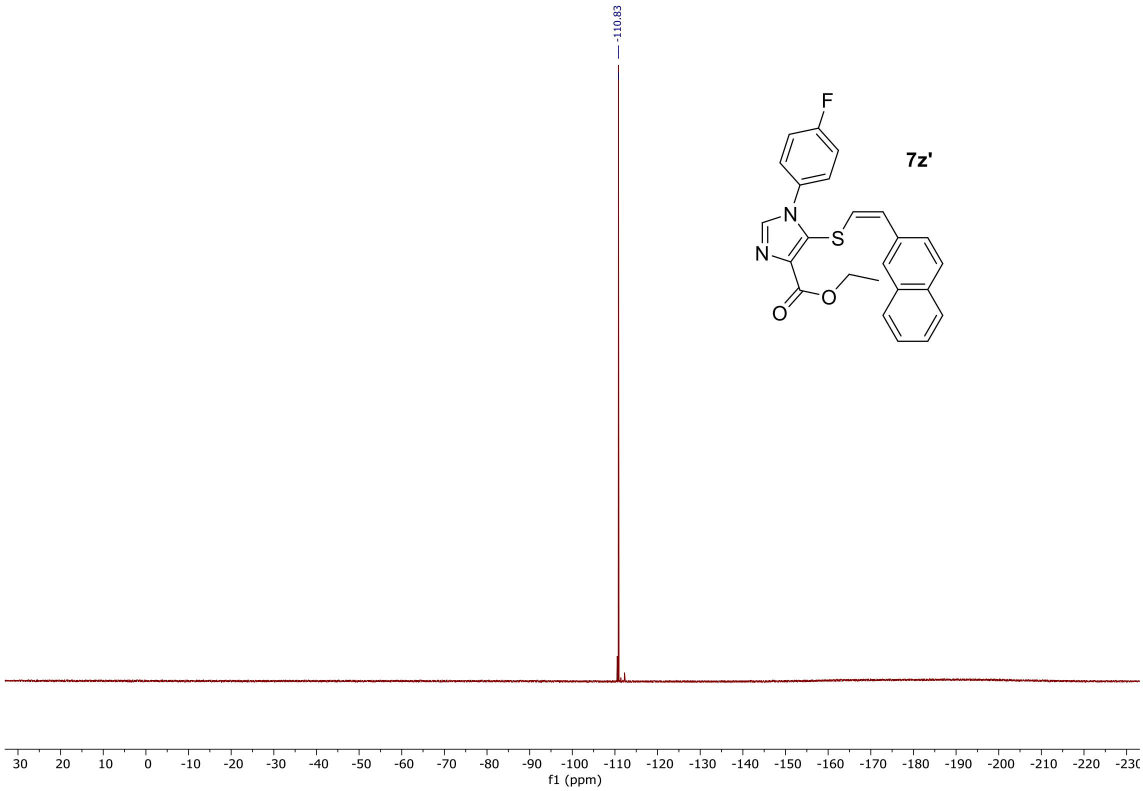

Ethyl 5-((4-((((3aS,5S,6R,6aS)-5-((R)-2,2-dimethyl-1,3-dioxolan-4-yl)-2,2-dimethyltetrahydrofuro[2,3-d][1,3]dioxol-6-yl)oxy)carbonyl)styryl)thio)-1-phenyl-1H-imidazole-4-carboxylate (7aa)

^{1}H RMN (300 MHz, $CDCl_3$, 300K)

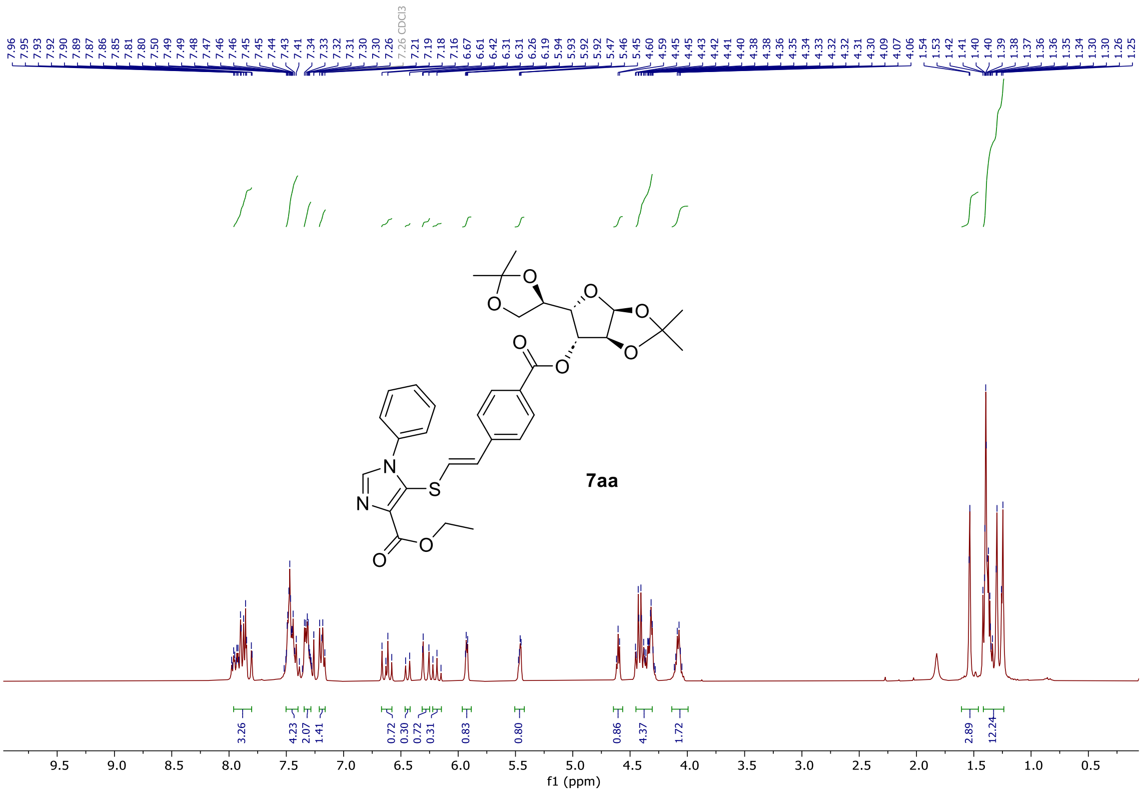

$^{13}C\{^{1}H\}$ NMR (75 MHz, $CDCl_3$, 300K)

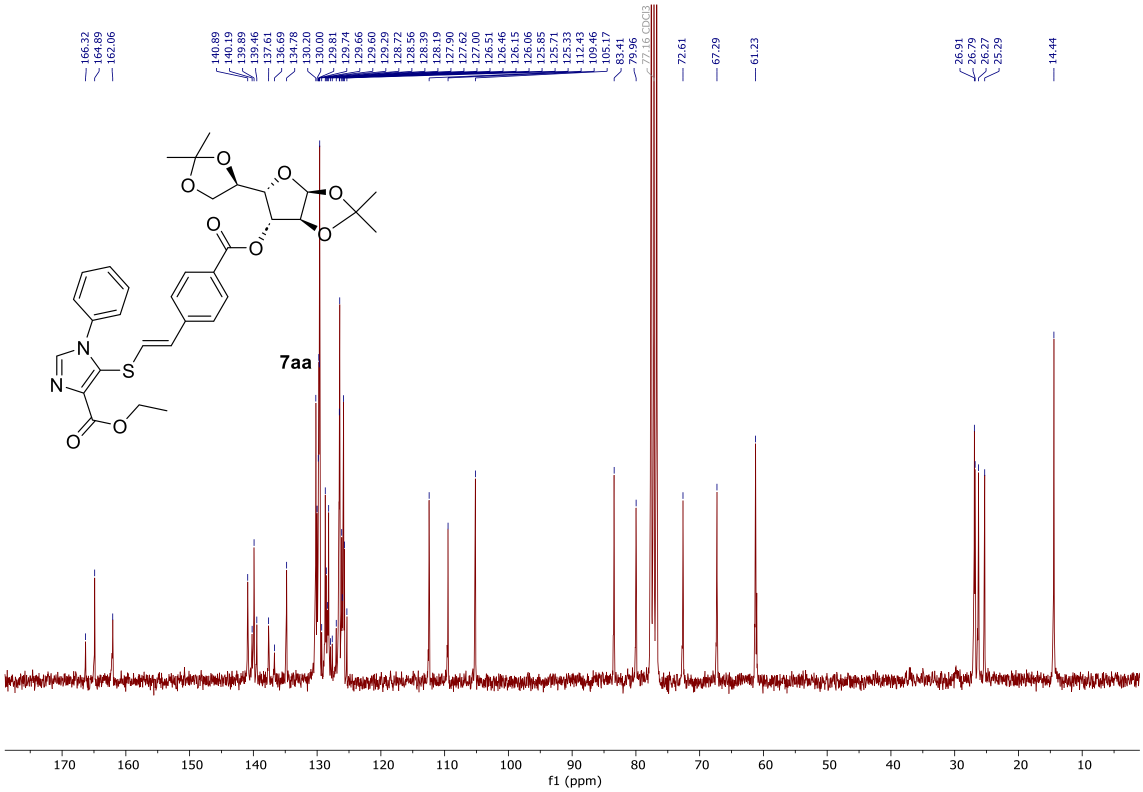

(*E*)-Ethyl 5-((4-((((1S,5R)-6,6-dimethylbicyclo[3.1.1]hept-2-en-2-yl)methoxy)carbonyl)styryl)thio)-1-phenyl-1H-imidazole-4-carboxylate (7ab)

^{1}H RMN (300 MHz, $CDCl_3$, 300K)

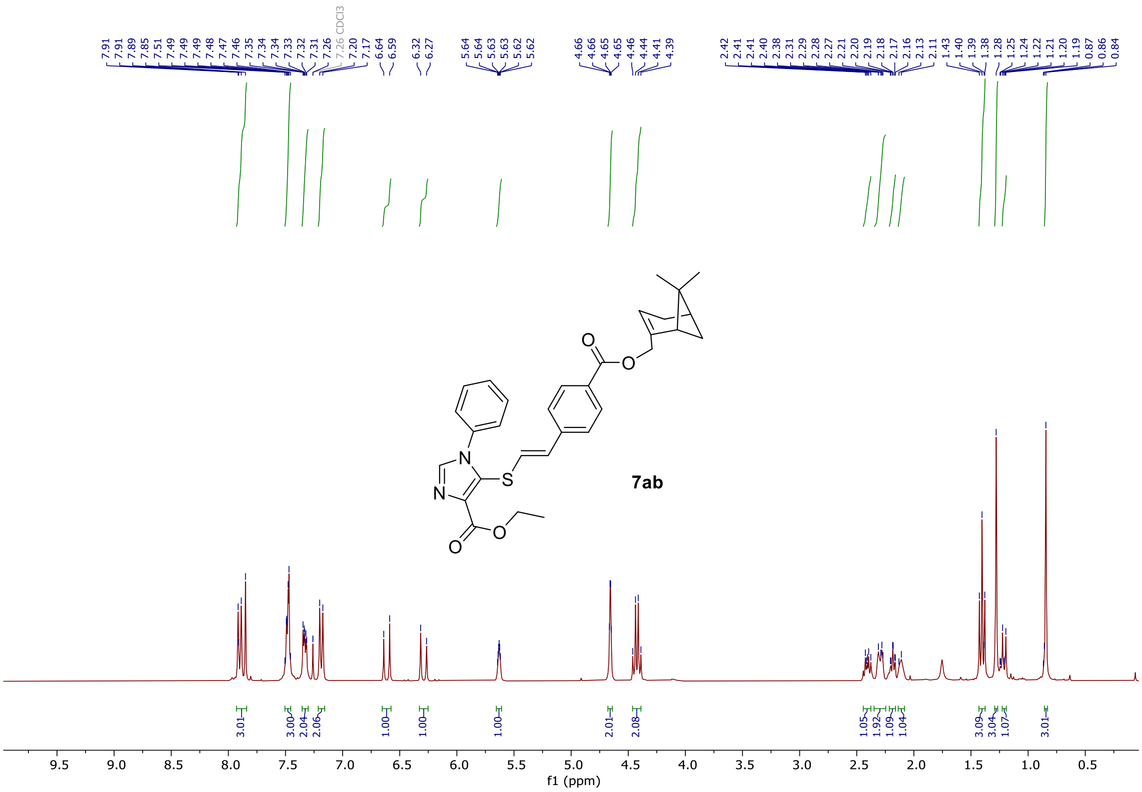

$^{13}C\{^{1}H\}$ NMR (75 MHz, $CDCl_3$, 300K)

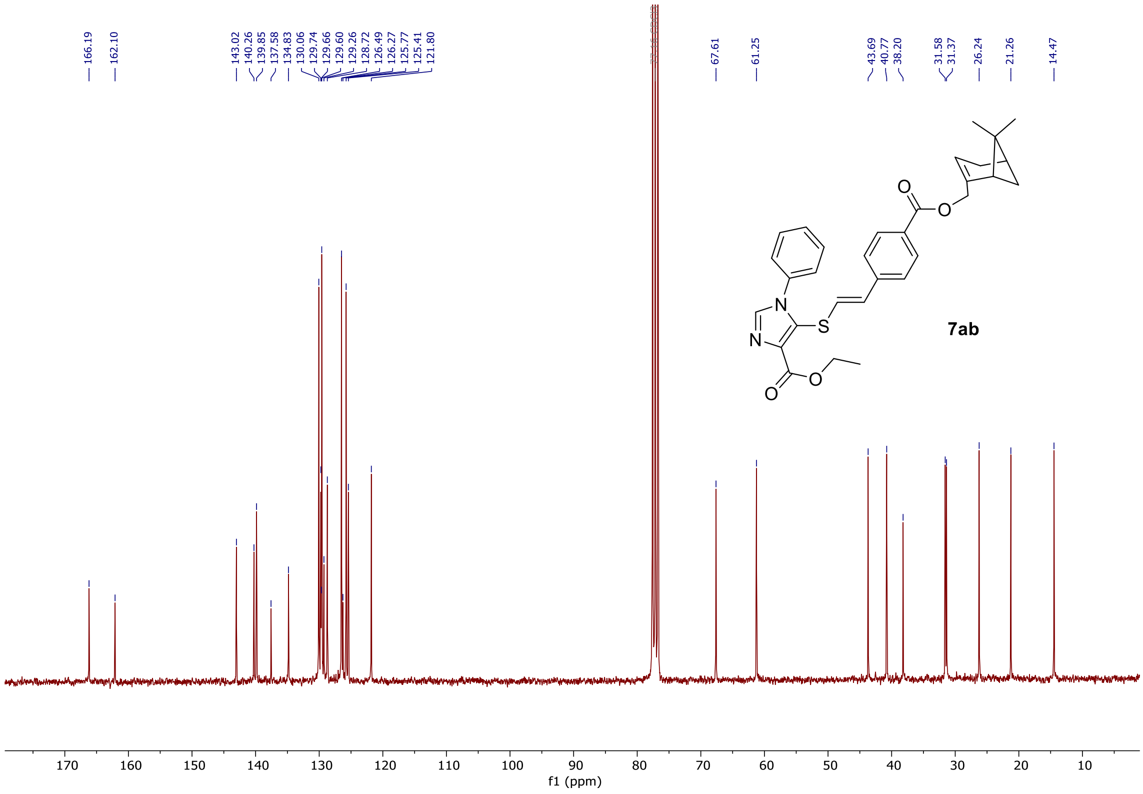

(*Z*)-Ethyl 5-((4-((((1S,5R)-6,6-dimethylbicyclo[3.1.1]hept-2-en-2-yl)methoxy)carbonyl)styryl)thio)-1-phenyl-1H-imidazole-4-carboxylate (7ab')

^{1}H RMN (300 MHz, $CDCl_3$, 300K)

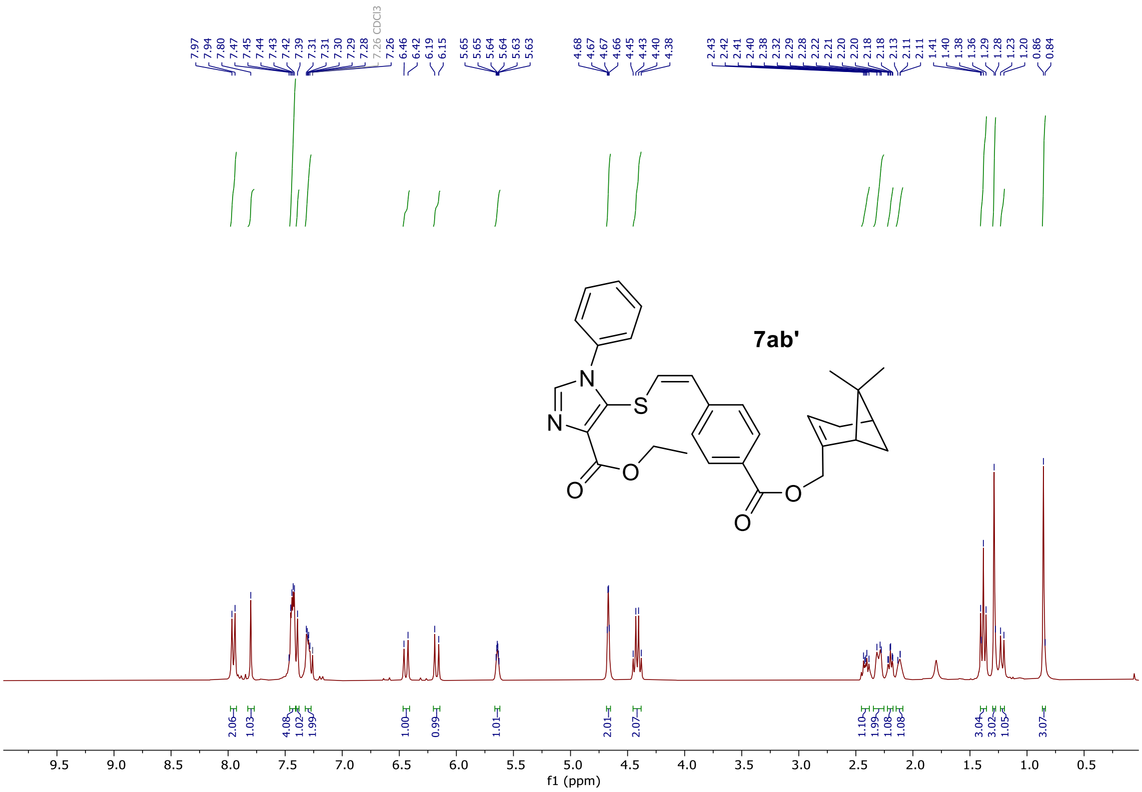

$^{13}C\{^1H\}$ NMR (75 MHz, $CDCl_3$, 300K)

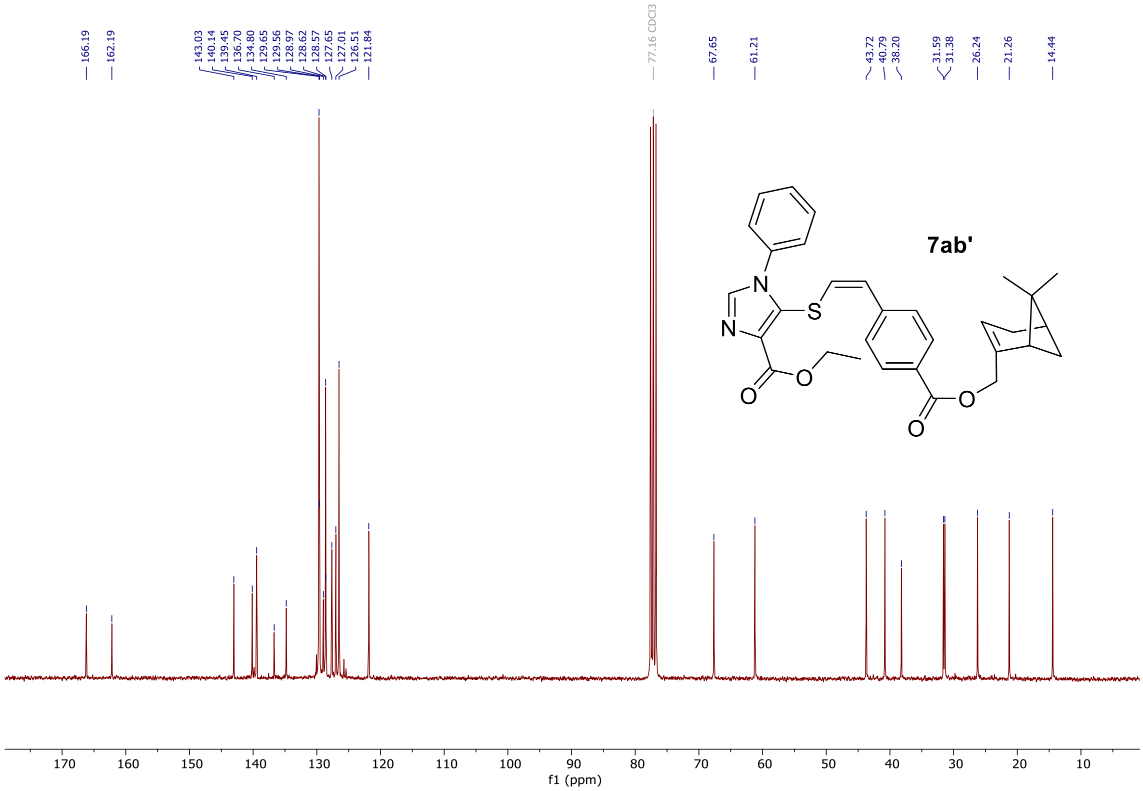

(*E*)-Ethyl 5-((4-((((1S,2R,5R)-2-isopropyl-5-methylcyclohexyl)oxy)carbonyl)styryl)thio)-1-phenyl-1H-imidazole-4-carboxylate (7ac)

^{1}H RMN (300 MHz, $CDCl_3$, 300K)

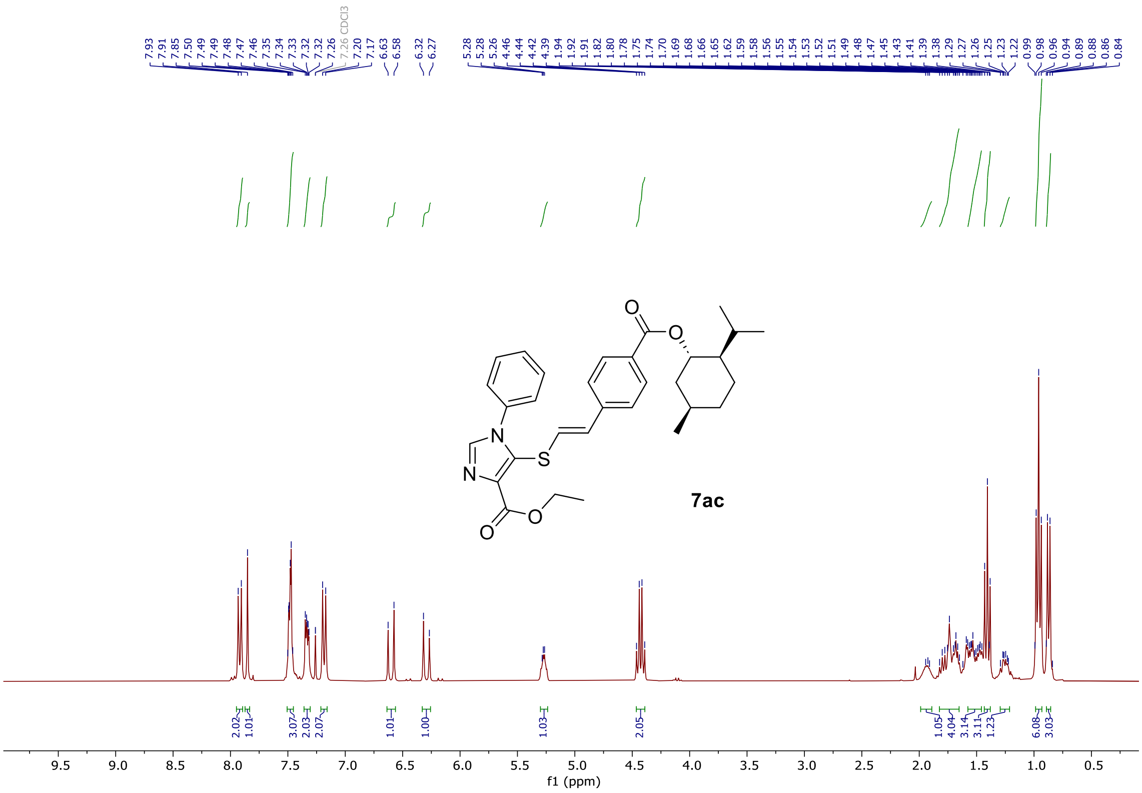

$^{13}C\{^1H\}$ NMR (75 MHz, $CDCl_3$, 300K)

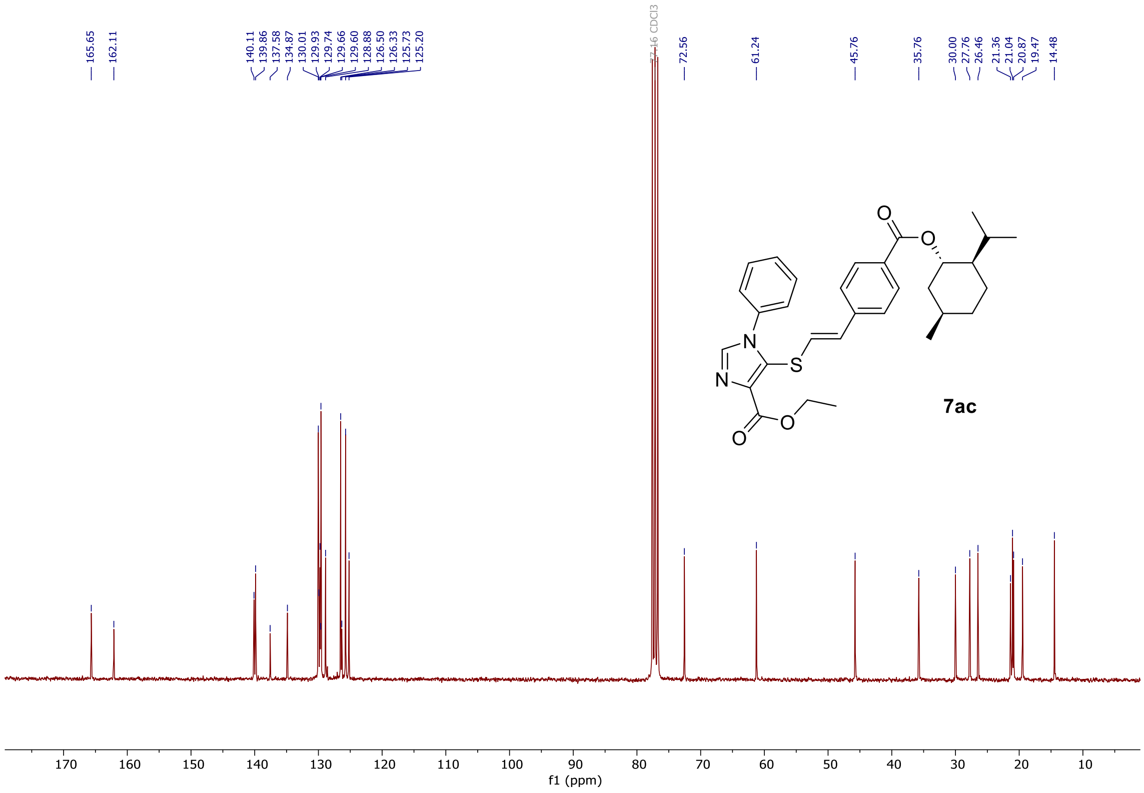

(*Z*)-Ethyl 5-((4-((((1S,2R,5R)-2-isopropyl-5-methylcyclohexyl)oxy)carbonyl)styryl)thio)-1-phenyl-1H-imidazole-4-carboxylate (7ac')

^{1}H RMN (300 MHz, $CDCl_3$, 300K)

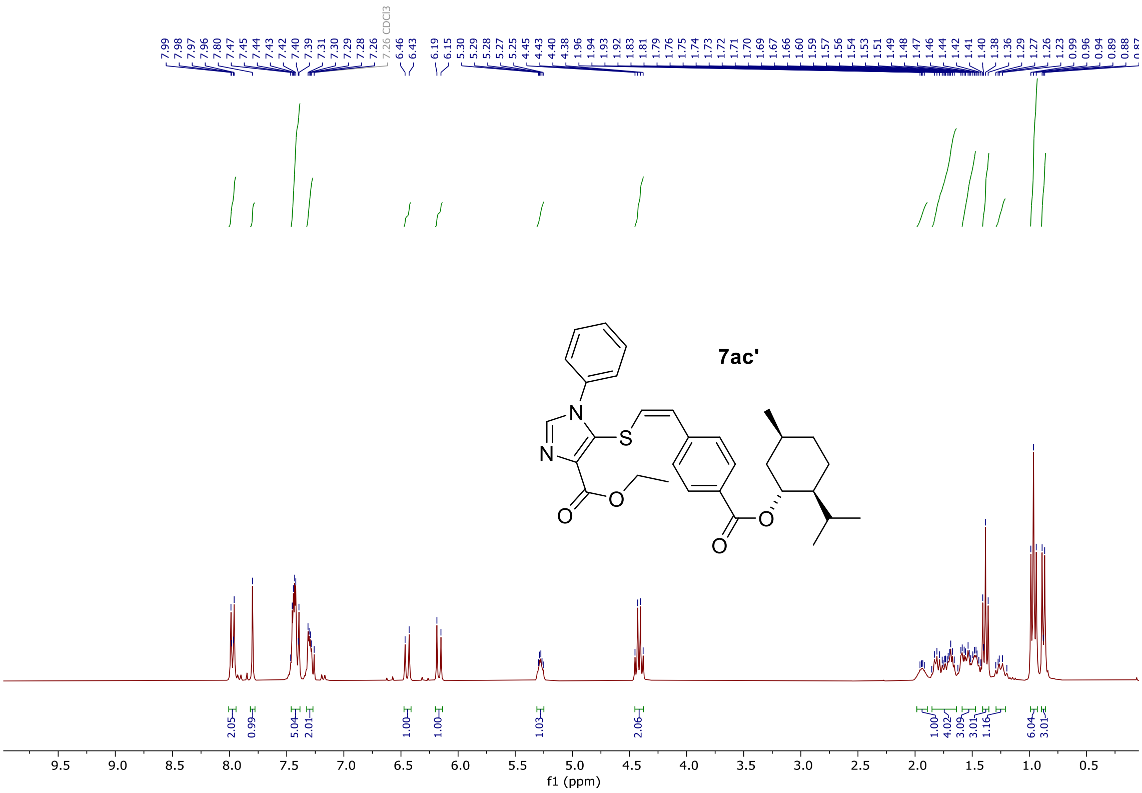

^{13}C{^{1}H} NMR (75 MHz, $CDCl_3$, 300K)

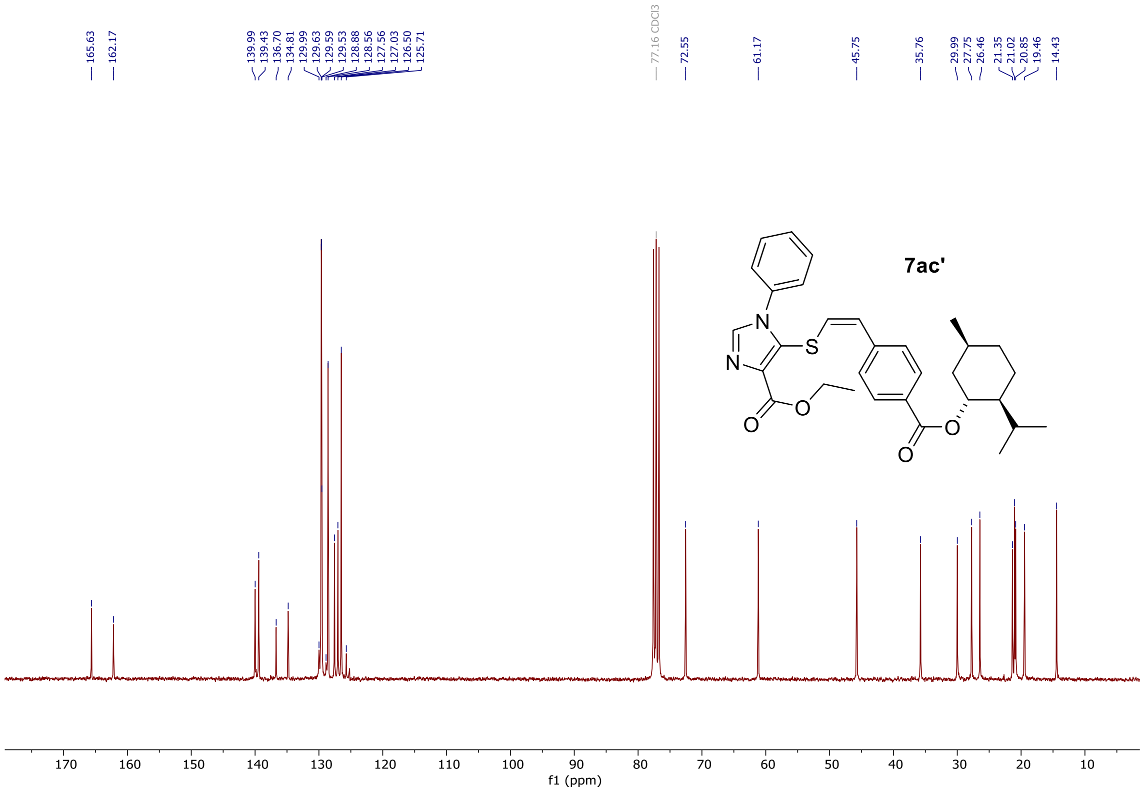

Ethyl 5-((4-(4-chlorophenyl)buta-1,3-dien-1-yl)thio)-1-phenyl-1H-imidazole-4-carboxylate (8a)

^{1}H RMN (300 MHz, $CDCl_3$, 300K)

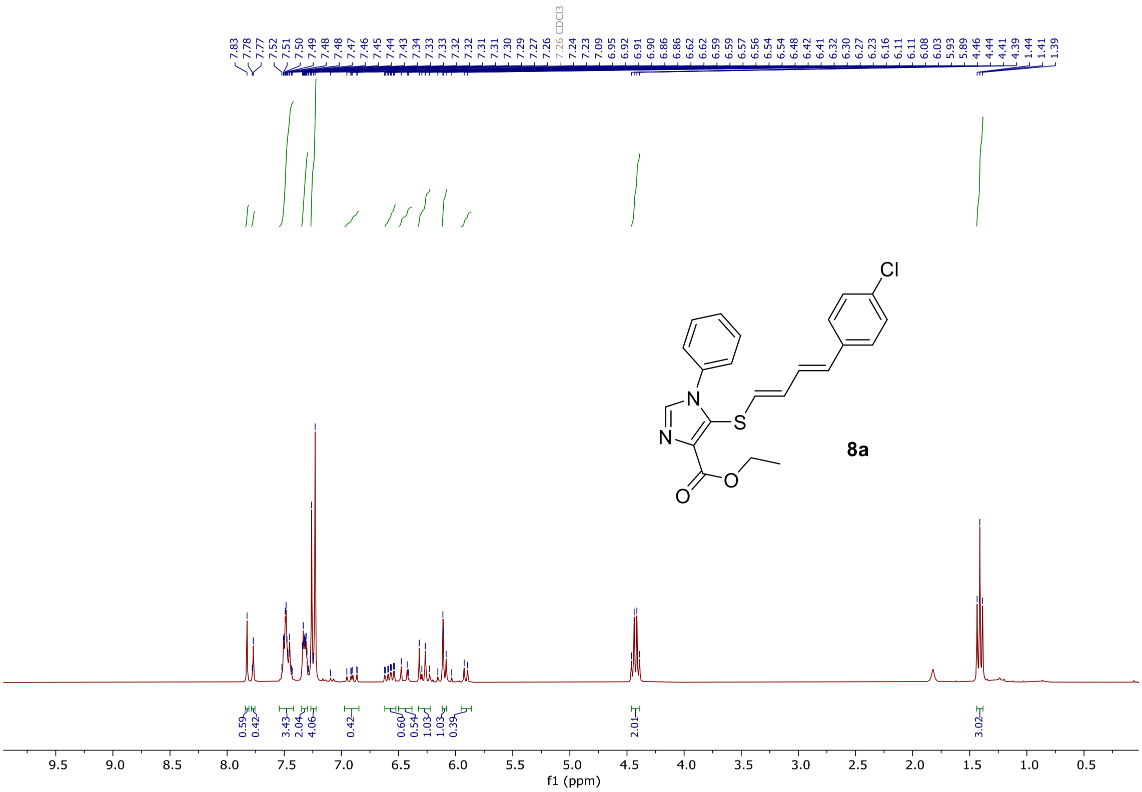

^{13}C{^{1}H} NMR (75 MHz, $CDCl_3$, 300K)

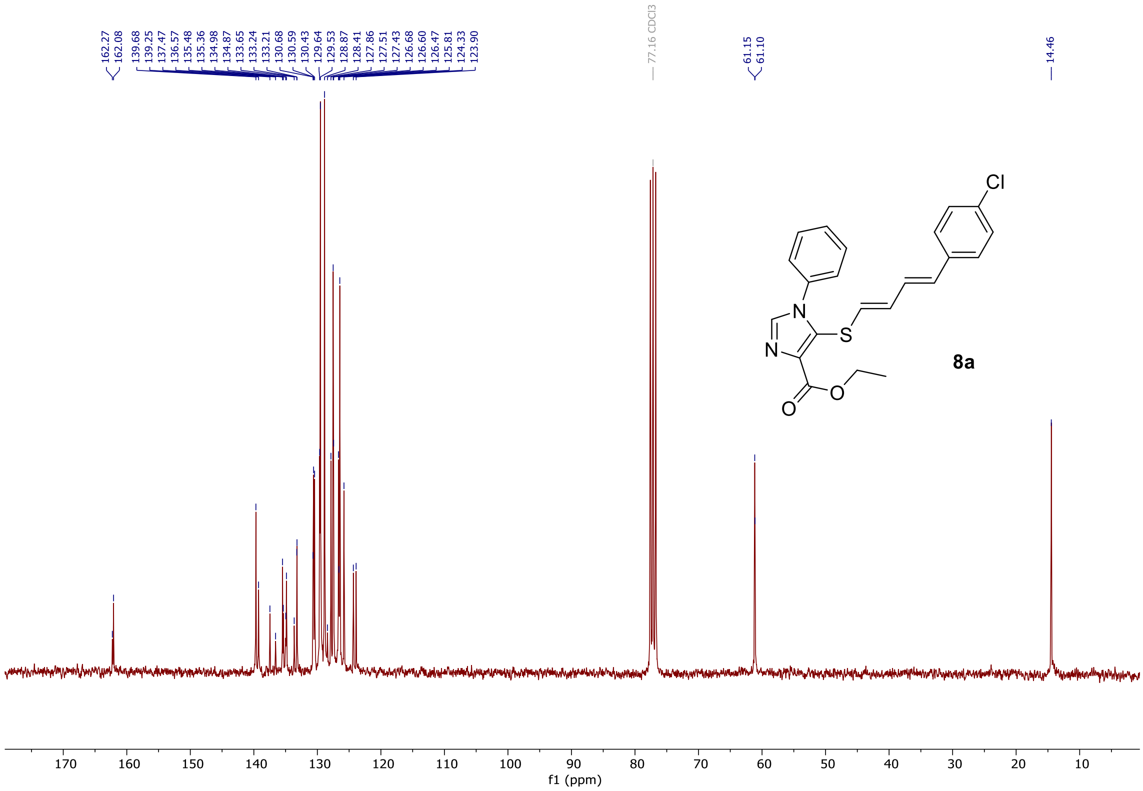

Ethyl 1-phenyl-5-(phenylthio)-1H-imidazole-4-carboxylate (9a)

^{1}**H RMN** (300 MHz, $CDCl_3$, 300K)

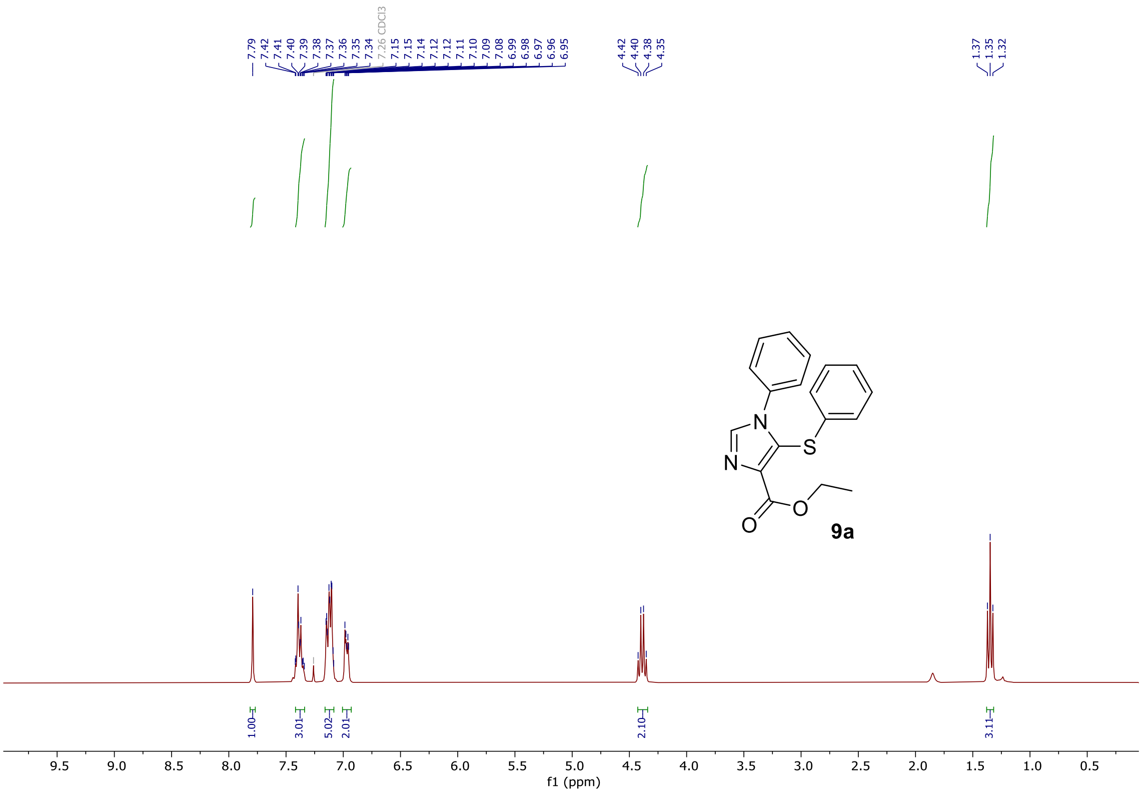

^{13}**C{^{1}H} NMR** (75 MHz, $CDCl_3$, 300K)

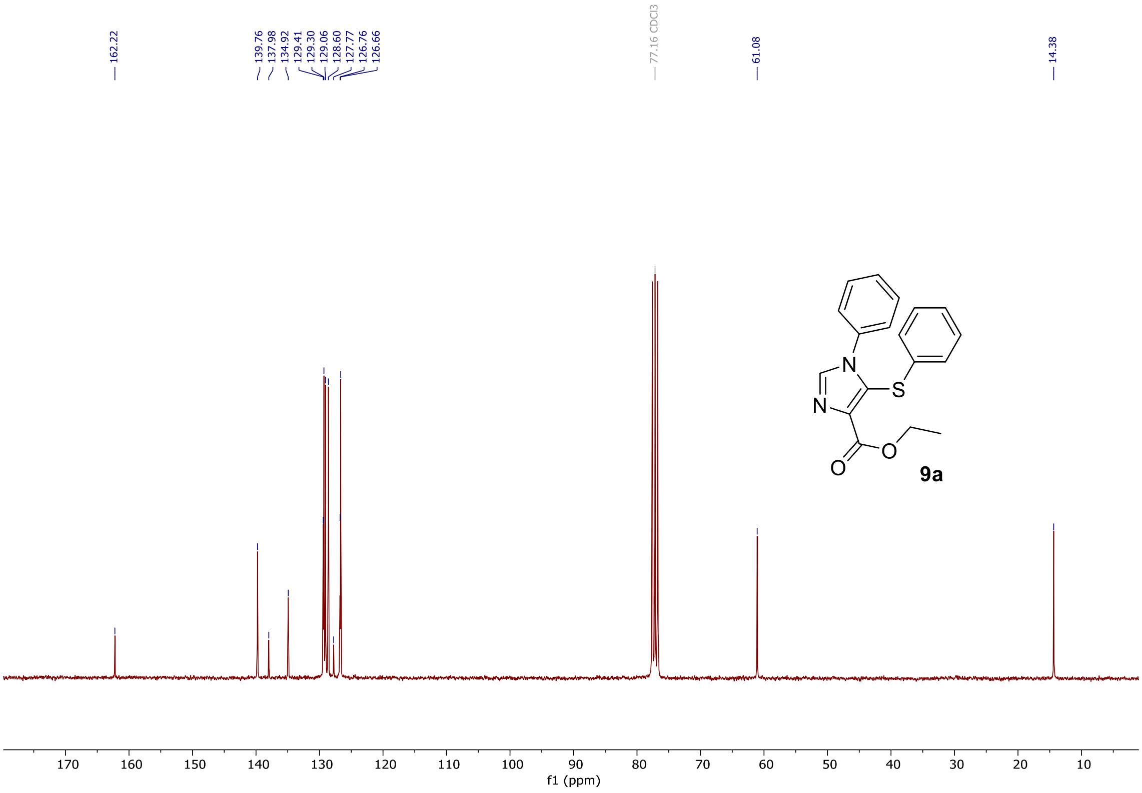

Ethyl 5-((4-(tert-butyl)phenyl)thio)-1-phenyl-1H-imidazole-4-carboxylate (9b)

^{1}H RMN (300 MHz, $CDCl_3$, 300K)

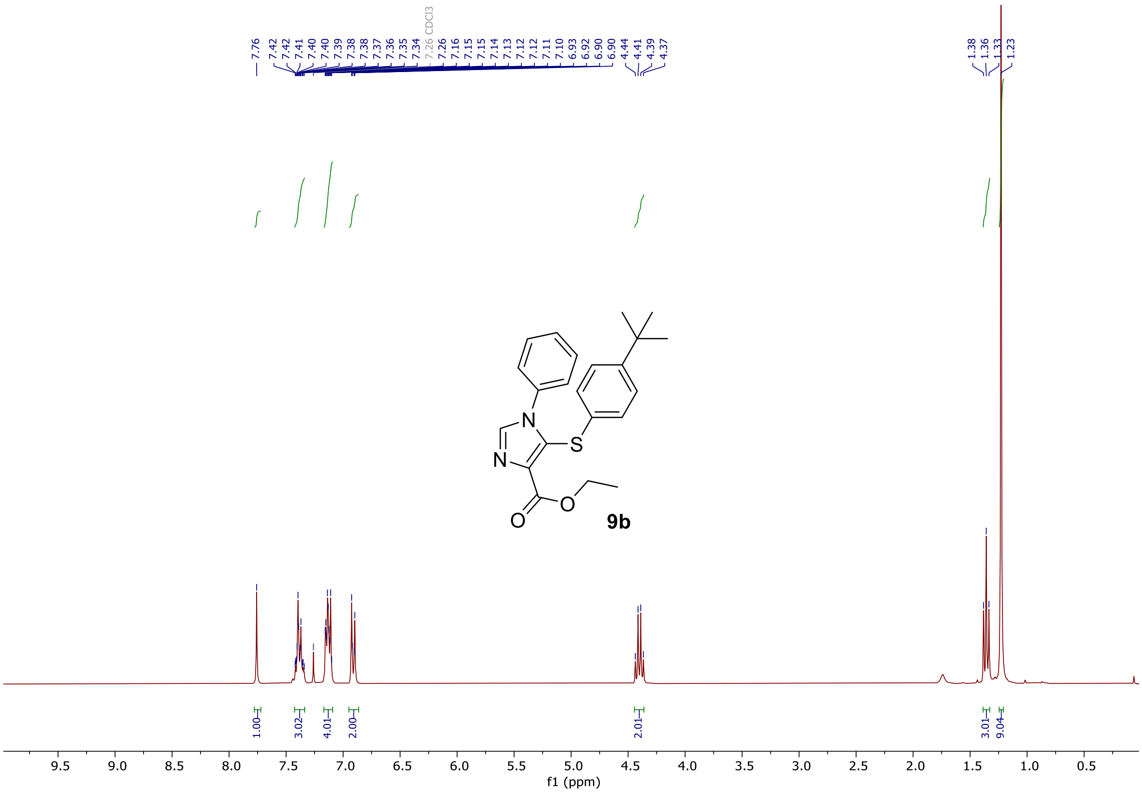

^{13}C{^{1}H} NMR (75 MHz, $CDCl_3$, 300K)

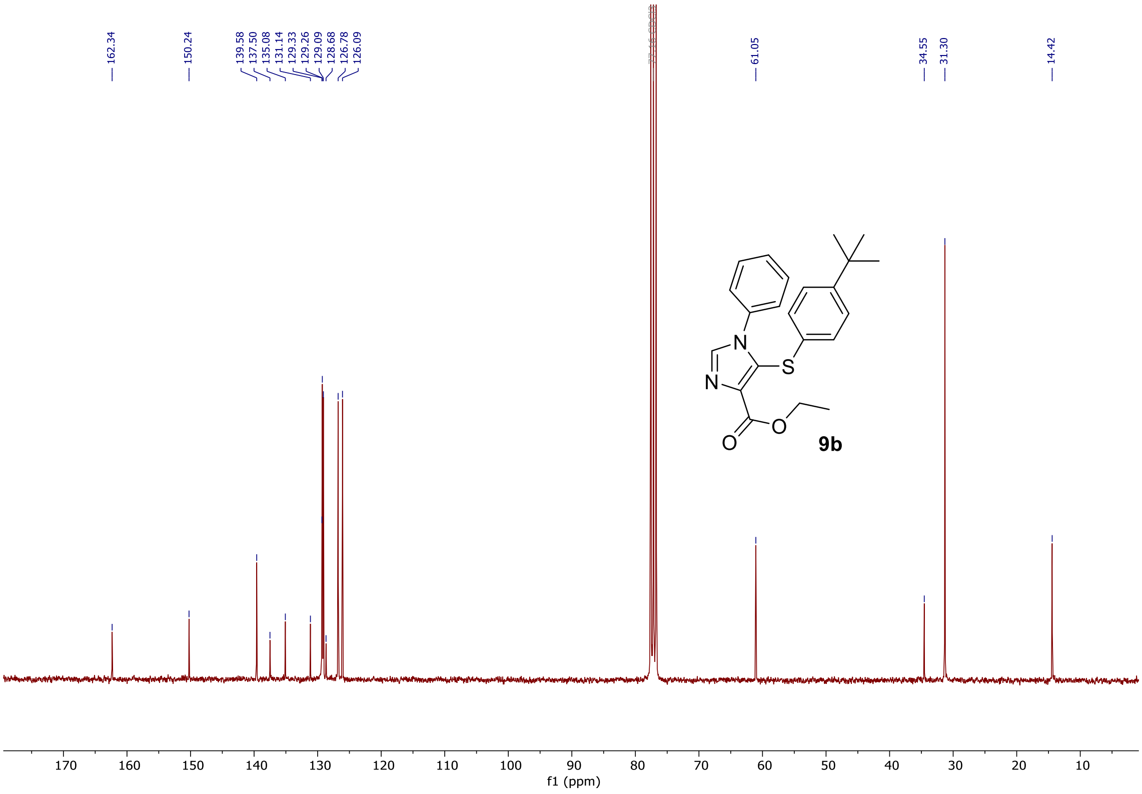

Ethyl 1-phenyl-5-((4-(trifluoromethyl)phenyl)thio)-1H-imidazole-4-carboxylate (9c)

^{1}H RMN (300 MHz, $CDCl_3$, 300K)

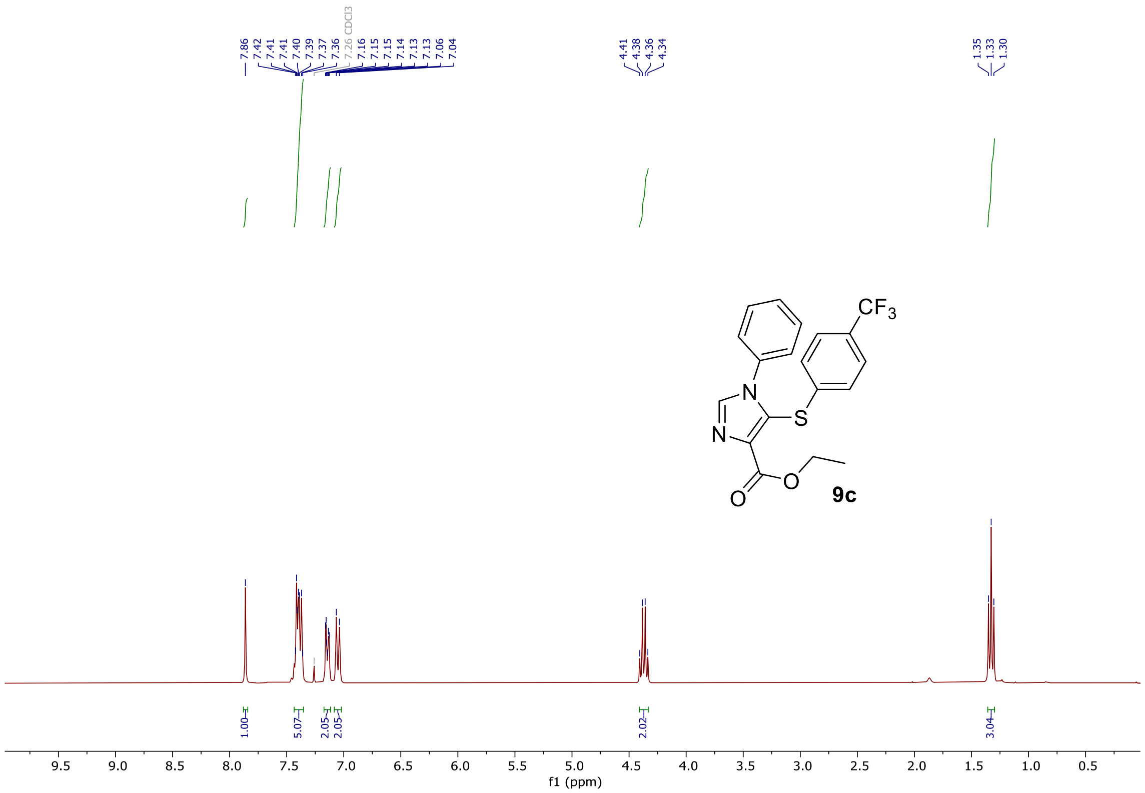

^{13}C{^{1}H} NMR (75 MHz, $CDCl_3$, 300K)

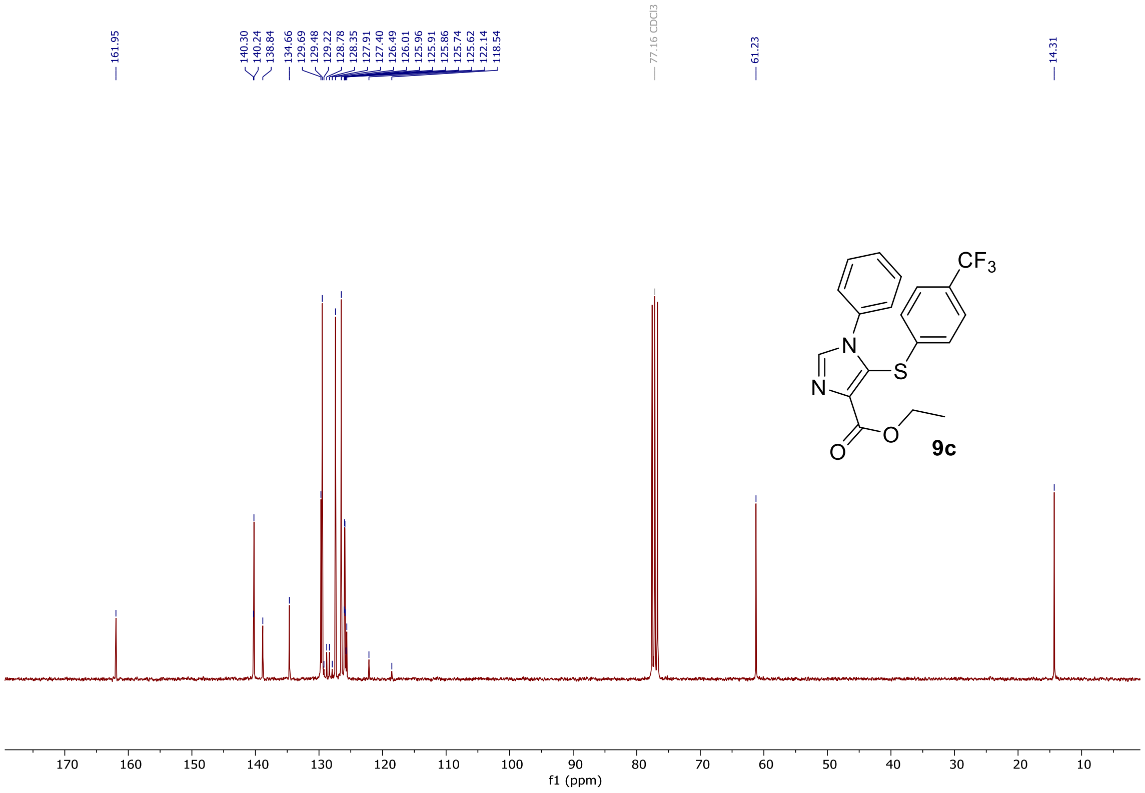

^{19}F RMN (282 MHz, $CDCl_3$, 300K)

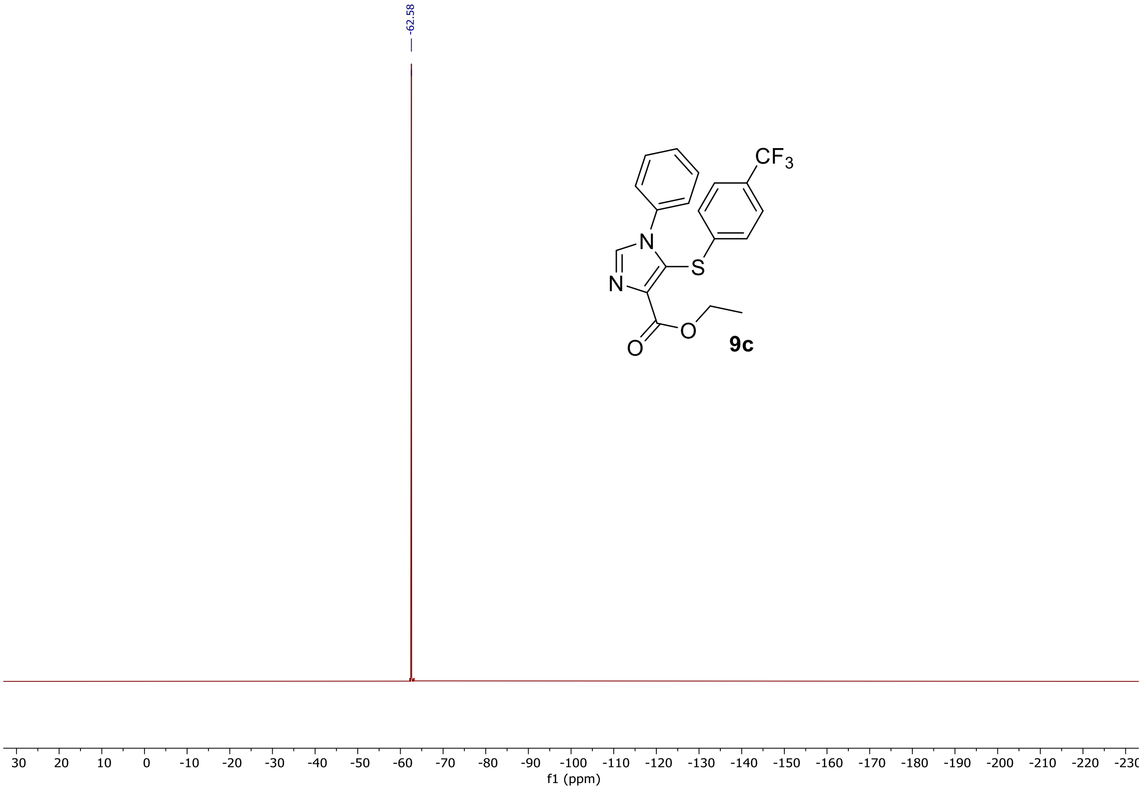

Ethyl 5-((4-ethylphenyl)thio)-1-phenyl-1H-imidazole-4-carboxylate (9d)

^{1}H RMN (300 MHz, $CDCl_3$, 300K)

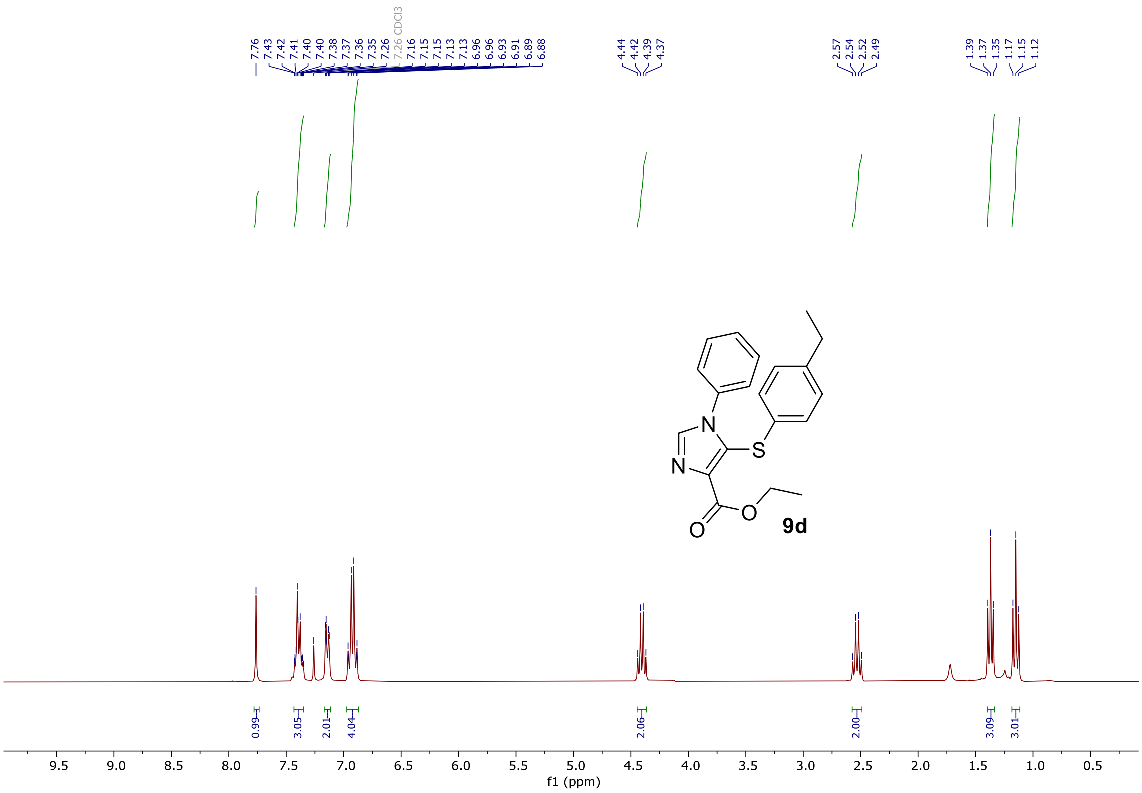

^{13}C{^{1}H} NMR (75 MHz, $CDCl_3$, 300K)

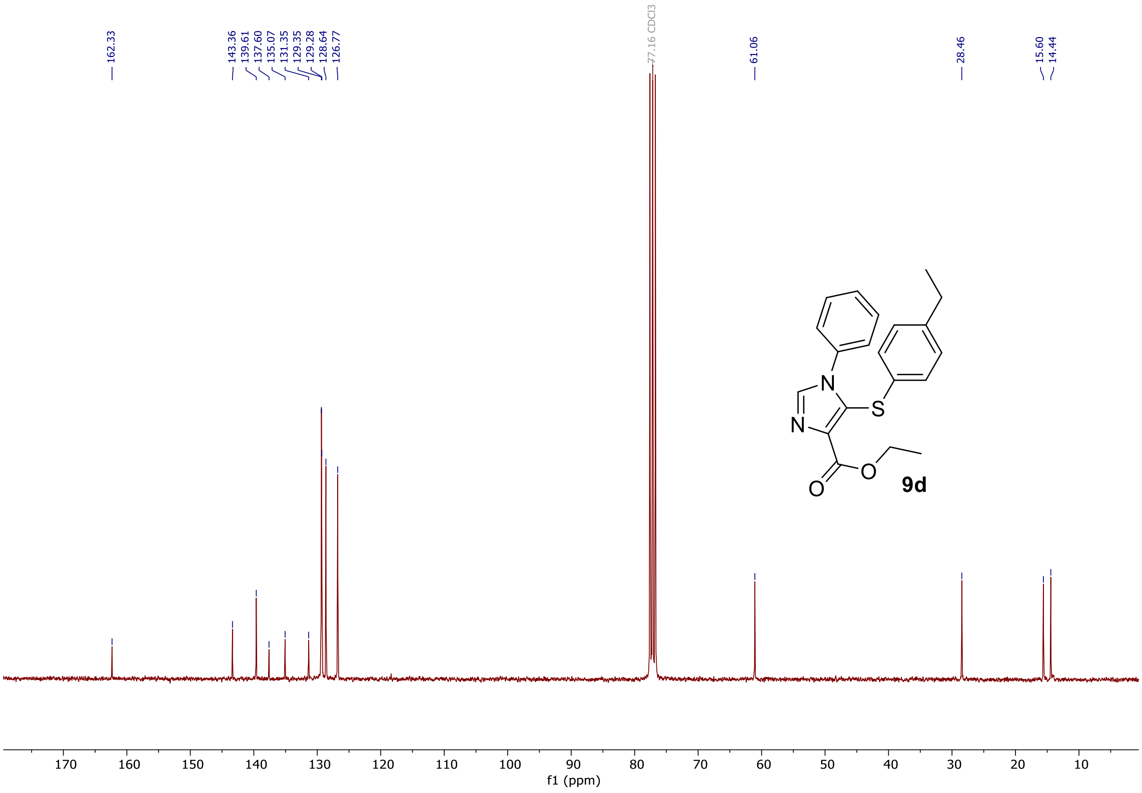

Ethyl 1-phenyl-5-(pyridin-2-ylthio)-1H-imidazole-4-carboxylate (9e)

^{1}H RMN (300 MHz, $CDCl_3$, 300K)

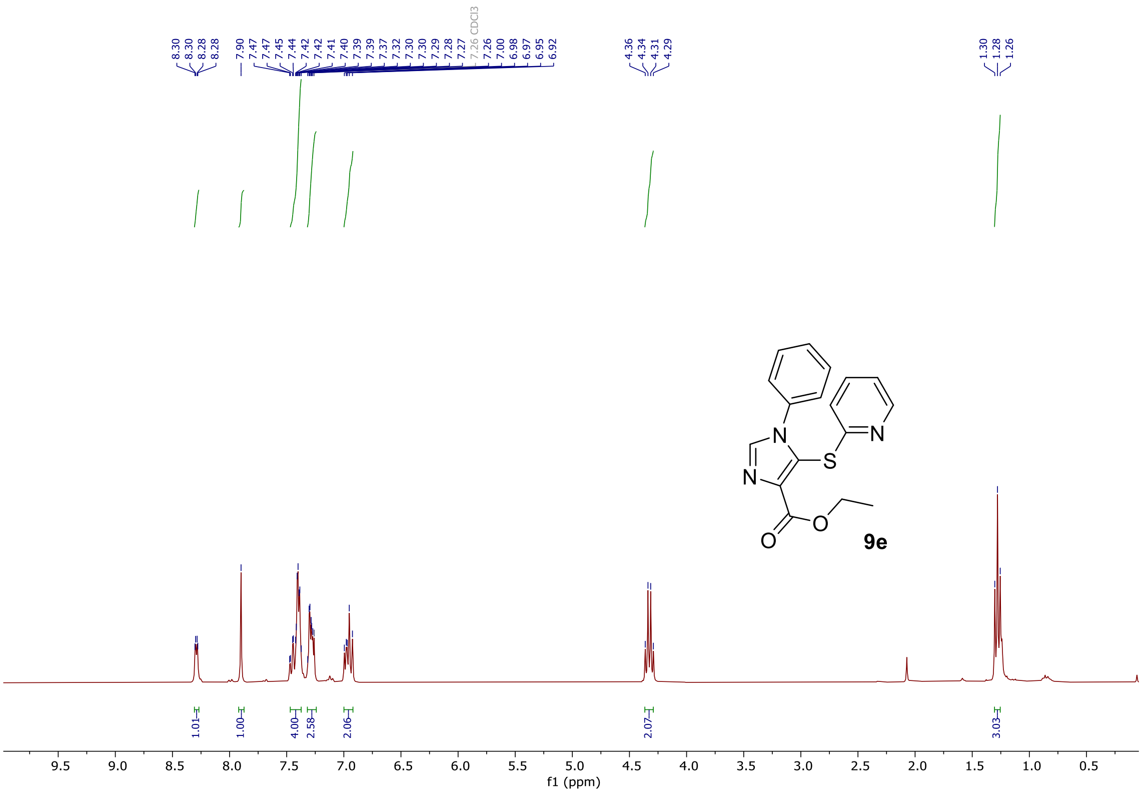

^{13}C{^{1}H} NMR (75 MHz, $CDCl_3$, 300K)

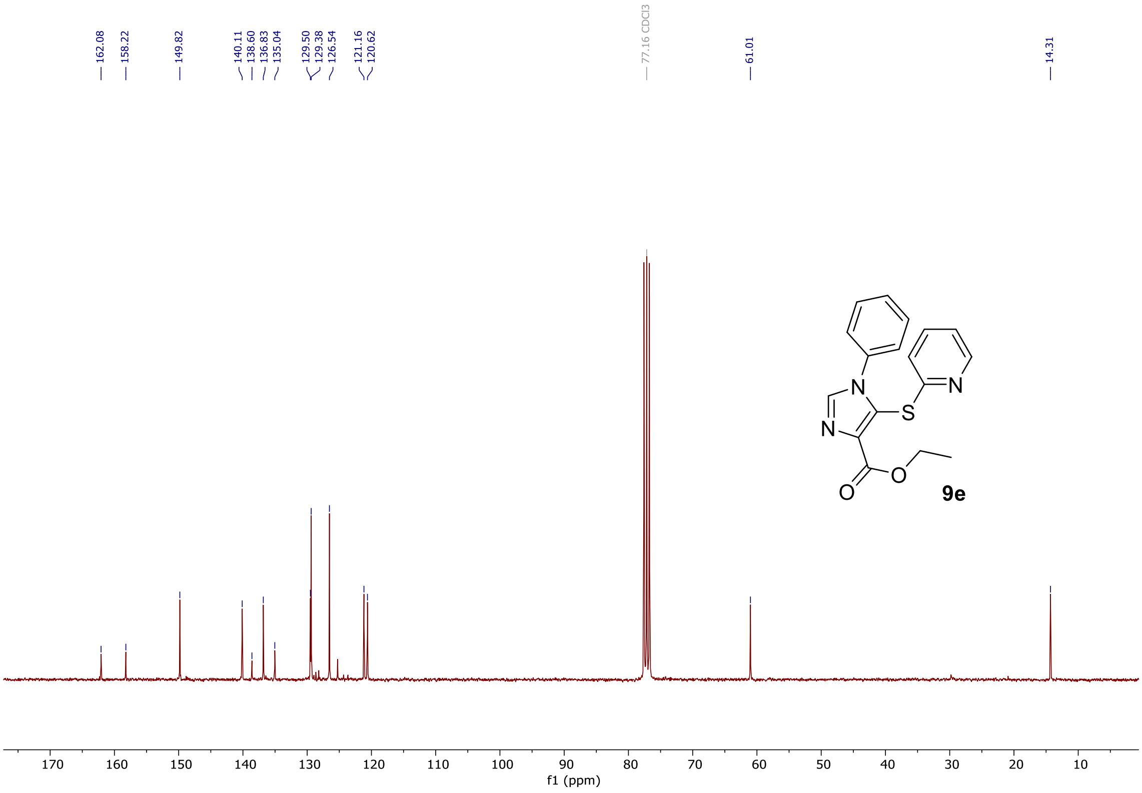

Ethyl 5-((3-formylphenyl)thio)-1-phenyl-1H-imidazole-4-carboxylate (9f)

^{1}H RMN (300 MHz, $CDCl_3$, 300K)

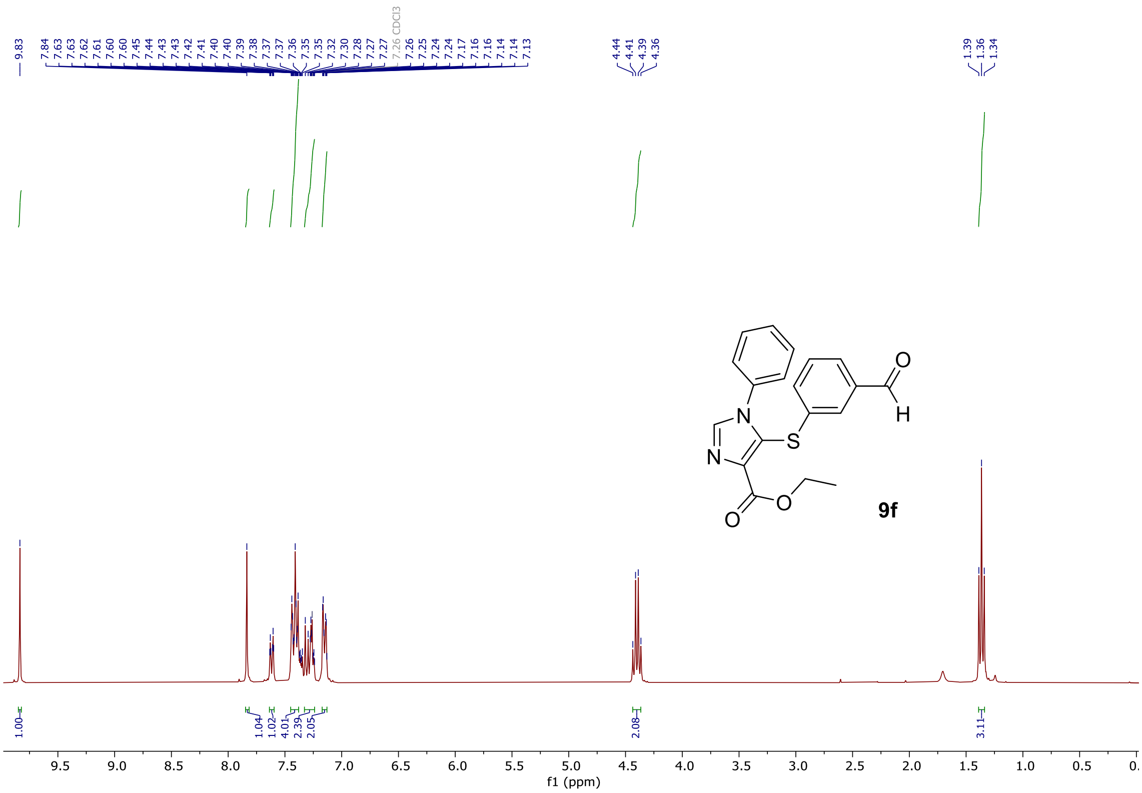

^{13}C{^{1}H} NMR (75 MHz, $CDCl_3$, 300K)

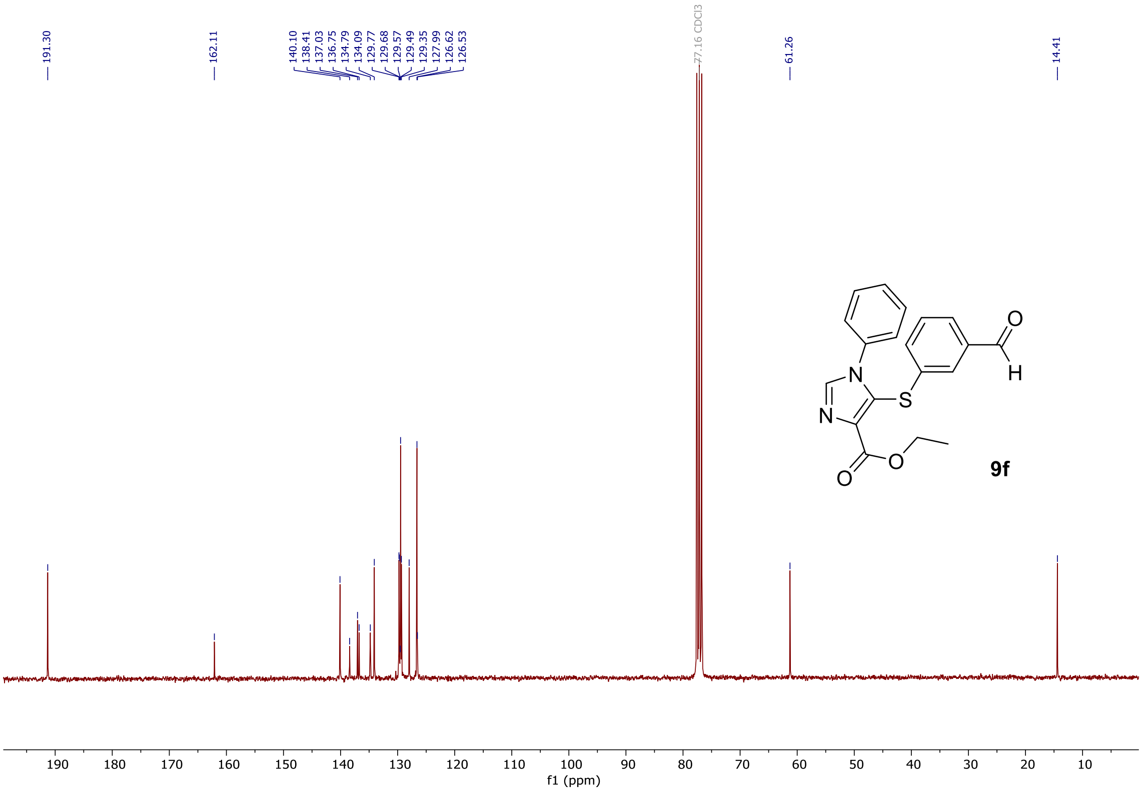

Ethyl 1-phenyl-5-(thiophen-2-ylthio)-1H-imidazole-4-carboxylate (9g)

^{1}H RMN (300 MHz, $CDCl_3$, 300K)

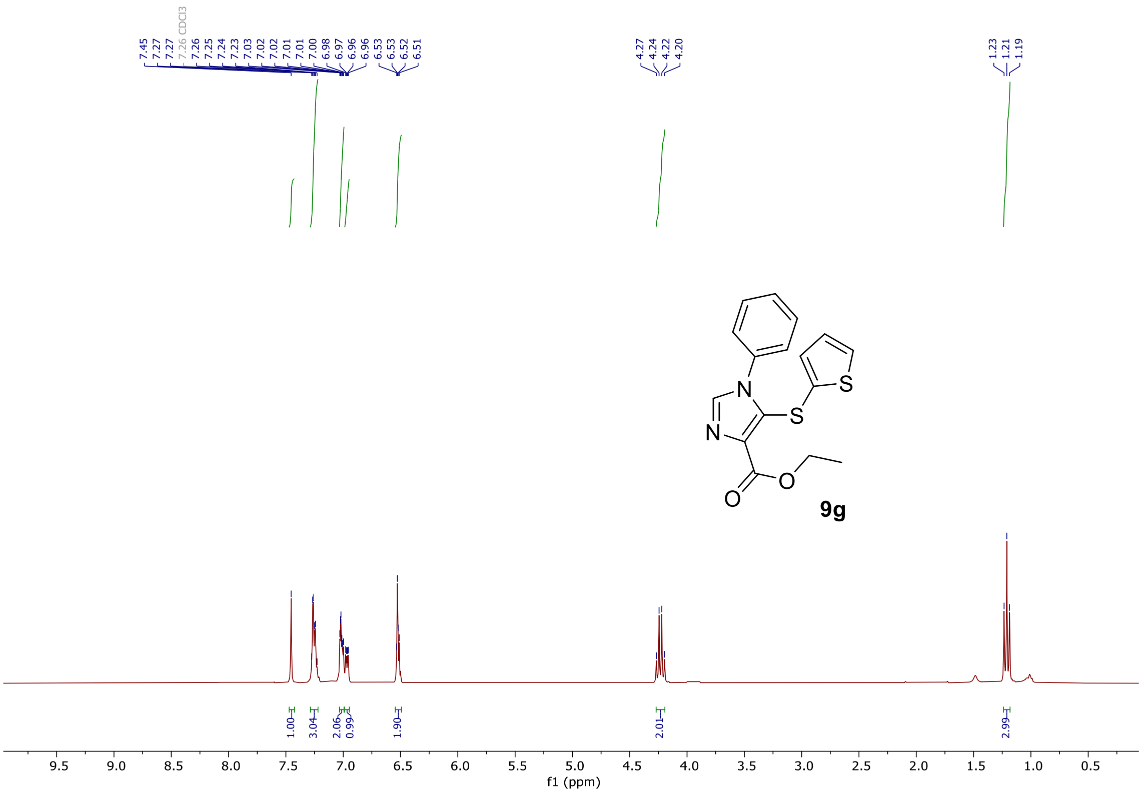

^{13}C{^{1}H} NMR (75 MHz, $CDCl_3$, 300K)

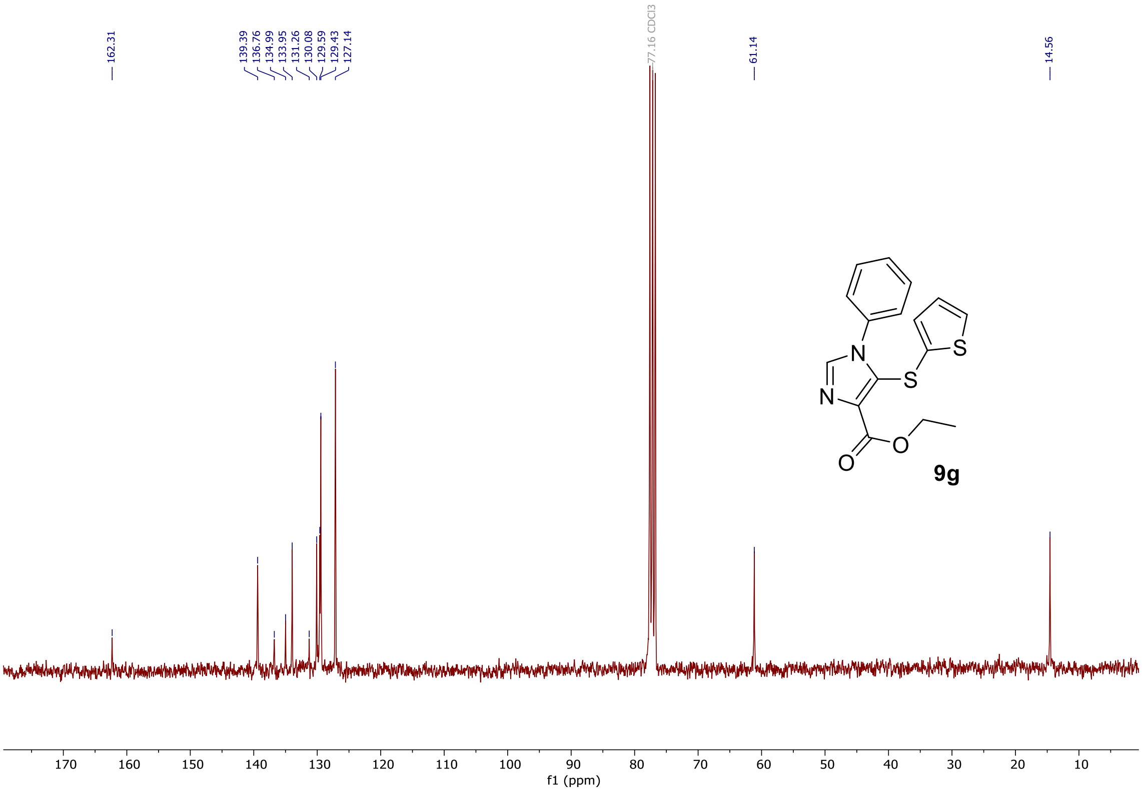

Supplement: Supplementary file 1 [file gg6c00004_si_001.pdf]
